# Next-Generation Sequencing in the Assessment of the Transcriptomic Landscape of DNA Damage Repair Genes in Abdominal Aortic Aneurysm, Chronic Venous Disease and Lower Extremity Artery Disease

**DOI:** 10.3390/ijms24010551

**Published:** 2022-12-29

**Authors:** Karol P. Ruszel, Daniel P. Zalewski, Andrzej Stępniewski, Dariusz Gałkowski, Jacek Bogucki, Marcin Feldo, Bartosz J. Płachno, Janusz Kocki, Anna Bogucka-Kocka

**Affiliations:** 1 Department of Clinical Genetics, Chair of Medical Genetics, Medical University of Lublin, 11 Radziwiłłowska St., 20-080 Lublin, Poland; 2 Chair and Department of Biology and Genetics, Medical University of Lublin, 4a Chodźki St., 20-093 Lublin, Poland; 3 Ecotech Complex Analytical and Programme Centre for Advanced Environmentally Friendly Technologies, University of Marie Curie-Skłodowska, 39 Głęboka St., 20-612 Lublin, Poland; 4 Department of Pathology and Laboratory Medicine, Rutgers-Robert Wood Johnson Medical School, One Robert Wood Johnson Place, New Brunswick, NJ 08903, USA; 5 Chair and Department of Organic Chemistry, Medical University of Lublin, 4a Chodźki St., 20-093 Lublin, Poland; 6 Chair and Department of Vascular Surgery and Angiology, Medical University of Lublin, 11 Staszica St., 20-081 Lublin, Poland; 7 Department of Plant Cytology and Embryology, Institute of Botany, Faculty of Biology, Jagiellonian University in Kraków, 9 Gronostajowa St., 30-387 Kraków, Poland

**Keywords:** DNA repair, lower extremity arterial disease, chronic venous disease, abdominal aortic aneurysm, gene expression, biomarker, next-generation sequencing

## Abstract

Vascular diseases are one of the most common causes of death and morbidity. Lower extremity artery disease (LEAD), abdominal aortic aneurysm (AAA) and chronic venous disease (CVD) belong to this group of conditions and exhibit various presentations and courses; thus, there is an urgent need for revealing new biomarkers for monitoring and potential treatment. Next-generation sequencing of mRNA allows rapid and detailed transcriptome analysis, allowing us to pinpoint the most pronounced differences between the mRNA expression profiles of vascular disease patients. Comparison of expression data of 519 DNA-repair-related genes obtained from mRNA next-generation sequencing revealed significant transcriptomic marks characterizing AAA, CVD and LEAD. Statistical, gene set enrichment analysis (GSEA), gene ontology (GO) and literature analyses were applied and highlighted many DNA repair and accompanying processes, such as cohesin functions, oxidative stress, homologous recombination, ubiquitin turnover, chromatin remodelling and DNA double-strand break repair. Surprisingly, obtained data suggest the contribution of genes engaged in the regulatory function of DNA repair as a key component that could be used to distinguish between analyzed conditions. DNA repair–related genes depicted in the presented study as dysregulated in AAA, CVD and LEAD could be utilized in the design of new biomarkers or therapies associated with these diseases.

## 1. Introduction

### 1.1. Cardiovascular Diseases

The global burden of disease-related mortality is shaped mainly by the prevalence of noncommunicable diseases like cancer, diabetes mellitus, cardiovascular and chronic respiratory diseases. Within this set of conditions, those related to the cardiovascular system are one of the most common causes of death and morbidity. Moreover, they will exhibit a constant trend toward being more prevalent in future decades [1,2,3].

Peripheral arterial disease (PAD), along with coronary heart disease and stroke, is a pronounced cause of demise and debilitation due to atherosclerotic complications [4]. Lower extremities arterial disease (LEAD) is the most common manifestation of PAD, characterised by chronic degenerative changes in the lower limbs (like ulceration or gangrene), intermittent claudication and pain [5]. Symptoms emerge due to formation of atheromas at different aortic vessel locations. The overall global prevalence of LEAD is estimated at 5.56% of individuals aged 25 years and older and exceeds 10% in people older than 70 years [6].

Abdominal aortic aneurysm (AAA) is most commonly defined by the maximum diameter of the abdominal aorta in excess of 3 cm in either anterior–posterior or transverse planes or, alternatively, as a focal dilation at least 1.5 times larger than the diameter of the normal adjacent arterial segment [7]. The prevalence of AAA ranges between 4% and 8% in the general population of men aged 65–80 years [8]. Typical risk factors for the development of AAA include age over 60, tobacco smoking and use, male gender, Caucasian race and family history of AAA, while aneurysm growth and rupture risk appear to be associated with persistent tobacco use, female gender and chronic pulmonary disease [7].

The main characteristics of chronic venous disease (CVD) include chronic morphological and functional abnormalities of the venous system caused by venous hypertension, venous valve incompetency and reflux in veins of the lower limbs, leading to venous wall remodelling and vascular inflammation. The clinical presentation varies from telangiectasia and varicose veins to much more severe complications like leg edema, skin changes and ulcers [9,10,11,12].

### 1.2. Reactive Oxygen Species and DNA Repair in Cardiovascular Diseases

It is widely accepted that oxidative stress and resulting DNA damage is present in various cardiovascular conditions. In atherosclerosis, DNA damage and repair is an important element of disease onset and progression [13,14,15]. It was revealed that impaired base excision repair of oxidative DNA damage in vascular smooth muscle cells triggers atherosclerosis-related processes [16], and alterations of the expression levels of certain DNA damage genes are seen throughout the course of the disease, being present in early plaques and almost universal in advanced ones [17]. The importance of base excision repair (BER) was shown in carotid atherosclerosis, where enhanced transcriptional activity of BER genes may contribute to nuclear DNA stability [18]. Enzymes involved in oxidative DNA damage repair (like OGG1) as well as other components of DNA repair pathways (like PARP1 or ATM kinase) were shown to have an important role in both diseases reviewed in [19]. DNA repair machinery may have a somewhat ambiguous role in acute kidney disease following an ischemia-reperfusion injury event; on the one hand, it is benevolent to DNA integrity, but on the other, it could fail the completion of the DNA repair process [20].

BER could be utilized in therapies reducing ischemic injury, where enhanced mitochondrial DNA repair could have a benevolent effect on the state of the human heart muscle and cerebral vasculature in stroke models [20,21].

The presented research is a part of a larger project regarding the application of next-generation sequencing in the identification of the transcriptomic signatures of three vascular diseases: LEAD [22], CVD [23] and AAA [24]. Our previous studies focused on the principal dissimilarities and resemblances between patients with the mentioned conditions, leading to an indication of gene expression patterns being either specific or common for studied diseases [25]. This approach, utilizing application of strictly defined criteria for gene choice, allowed us to define only those genes with a very high impact on the conditions studied. Due to this fact, other genes with potential relevance in pathology may have been omitted. This prompted us to change our approach and focus on genes contributing to more specified biological processes. Our attention was attracted to DNA damage repair and metabolism. The results obtained revealed biological phenomena related to not only direct DNA repair, but also more general processes connected to regulation of DNA repair by phosphorylation or ubiquitination/deubiquitination, reactive oxygen species and chromatin remodeling, replication and transcription. Current work adds new knowledge to the area of DNA repair in vascular diseases which may provide new insights into their biomarker composition as well as into new therapeutic approaches utilizing DNA damage metabolism genes.

## 2. Results

### 2.1. Study Group Attributes

The study group consisted of seven patients with AAA, seven patients with CVD, eight patients with LEAD, and seven controls with no cardiovascular conditions. The clinical characteristics of the participants are presented in Table 1. Inclusion and exclusion criteria as well as a detailed demographical and clinical description of each study group and RNA sequencing quality data were provided in [22,23,24]. Although the statistical analysis revealed no significant differences in smoking habits and sex, there were significant (*p* < 0.05) dissimilarities in age and body mass index (BMI) (Table 1). While LEAD and AAA share common conspicuous risk factors, such as older age and higher BMI, the control group was devoid of any signs of vascular pathology due to the inclusion of younger, non–smoking subjects with a lower BMI. The influence of age, sex, BMI and smoking status on obtained results was further examined in the text.

### 2.2. The Differential Expression Analysis of DNA Damage Metabolism Genes in AAA, CVD, LEAD and Control Subjects

In order to select the set of genes engaged in DNA damage metabolism, the HUGO Gene Nomenclature Committee database (https://www.genenames.org/) (accessed on 15 June 2021) was queried with the term “DNA repair”, yielding 449 hits. To provide further details, the obtained gene list was compared with the Human DNA Repair Genes (https://www.mdanderson.org/documents/Labs/Wood-Laboratory/human-dna-repair-genes.html) (accessed on 17 June 2021), and all genes not presented in the HUGO set were added. Moreover, the gene list was supplemented with the remaining ALKB homologs (ALKBH1-8 and FTO) due to their established role in nucleic acid modification and repair [26]. After confronting gene names with names and synonyms indicated by Human Gene Database GeneCards v5.0 (https://www.genecards.org) (accessed on 18 June 2021), the final list of genes submitted to further analysis had 519 positions (Table S1).

DESeq2 and UVE-PLS (uninformative variable elimination by partial least squares) methods were used to assess the differential expression analysis of the set of 519 selected genes in pairwise comparisons between studied groups (LEAD vs. control, AAA vs. control, CVD vs. control, LEAD vs. AAA, LEAD vs. CVD and AAA vs. CVD). To supplement pairwise analysis, pooled group comparisons were also conducted. In the first instance, the transcriptomic landscape of genes in a pooled group including LEAD, AAA and CVD subjects was compared with gene expression in control group, to reveal a general scheme of DNA damage metabolism gene expression in vascular pathogenesis. The second association was performed between gene expression in a pooled group containing LEAD and AAA subjects, representing artery-related diseases and CVD patients, to pinpoint differences in DNA repair-associated pathological processes ongoing in arteries compared with venous systems.

The quality of the results was assessed and presented as Cook’s distances of genes expression across all samples (Figure S1) with accompanying MA plots and histograms of *p* values generated for each comparison (Figures S2–S9).

**Figure 1:**
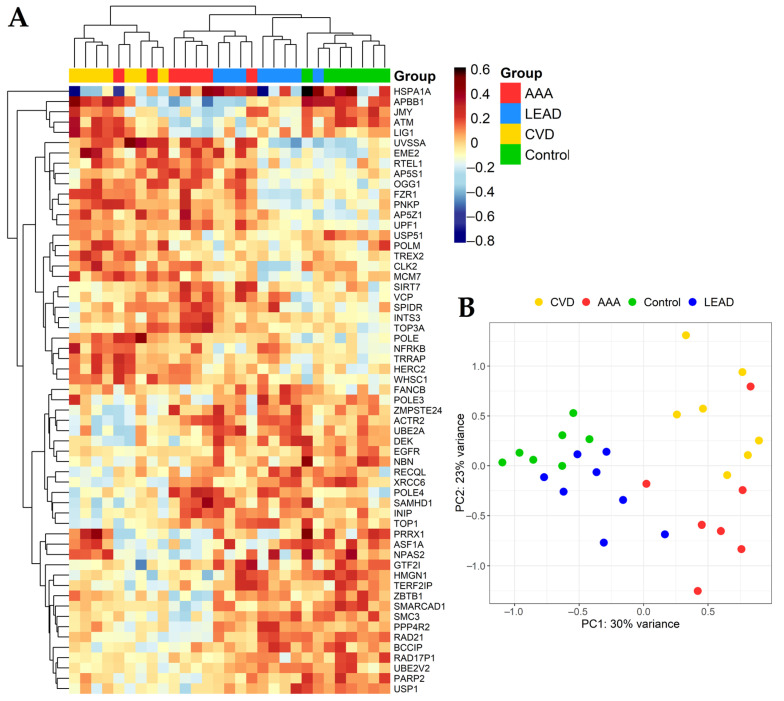
Differentiation in expression of genes selected from performed comparisons. (**A**) Heatmap with clustering of Euclidean distances using the complete method. (**B**) Principal component analysis (PCA) plot. LEAD—lower extremities arterial disease, AAA—abdominal aortic aneurysm, CVD—chronic venous disease, Control—healthy persons.

Distinct thresholds were set for further analysis; in the DESeq2 method, only genes with *p* < 0.05 (after adjustment using the Benjamini–Hochberg false discovery rate) and fold change > 1.2 (for upregulated genes) or fold change < 0.8 (for downregulated genes) were selected, while genes indicated by UVE-PLS as informative, with a minimum reliable score equal to 5, were considered. Both methods gave separate sets of genes in each of the comparisons (supplementary Tables S2 and S3). Genes universal for both sets were subjected to further analysis (59 genes in total). The differential expression parameters of these genes are presented in Table 2. Expression patterns of selected genes in the studied samples are presented on a heatmap with hierarchical clustering, as well as on a principal component analysis plot (Figure 1). Receiver operating characteristics (ROC) analysis was used to assess the specificity and sensitivity of selected genes as potential biomarkers for analyzed diseases. The results of the ROC analysis are presented in Table 2 (ROC-AUC values) and Table S4 (all results). Graphical representation of the relationships between sensitivity and specificity calculated for each gene (ROC plots) is presented in Figures S10–S17. Representative electropherograms of total RNA and corresponding libraries of randomly selected samples are presented in Figure S18.

Graphical representation of the results of UVE-PLS analysis is presented in Figures S19 and S20. Relationships between the categorical characteristics of study subjects (gender, smoking status) and expression of genes selected from comparisons, along with correlation analysis between characteristics (age and BMI) of studied groups and expression of genes selected from all comparisons performed, are presented on Table S6 and Table S7, respectively.

**Figure 2:**
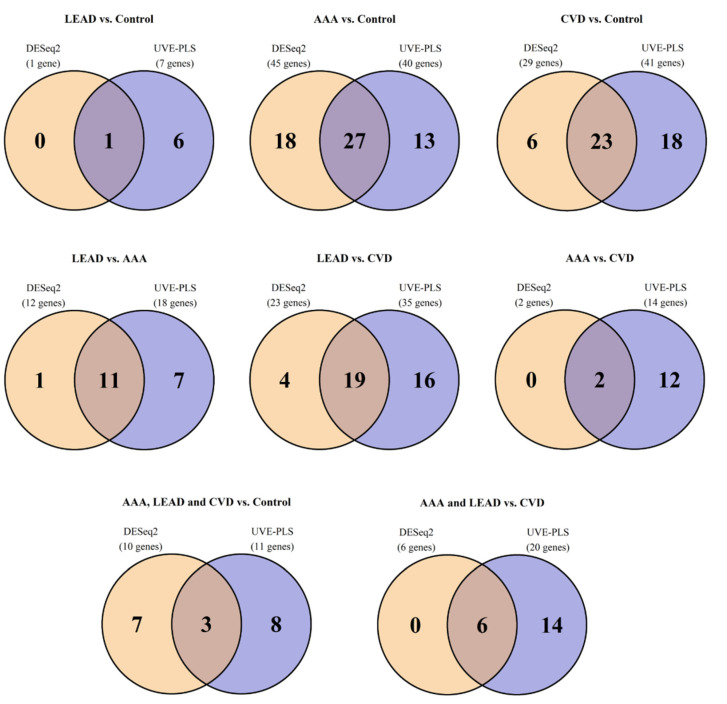
Relations of the quantity of differentially expressed genes in the pairwise and pooled group comparisons. Gene sets were obtained by DESeq2 and UVE–PLS methods with unified selection criteria applied (*p* value adjusted by Benjamini—Hochberg false discovery rate < 0.05 and fold change > 1.2 (for upregulated genes) or <0.8 (for downregulated genes), as well as UVE-PLS reliability score ≥ 5). The numbers in overlapping fields in each Venn diagram represent the number of genes shared by both DESeq2 and UVE-PLS gene sets.

Sets of genes selected from performed comparisons are shown on a Venn Diagram to identify genes being unique or common for more than one comparison (Figure 2, Table S5).

**Figure 3:**
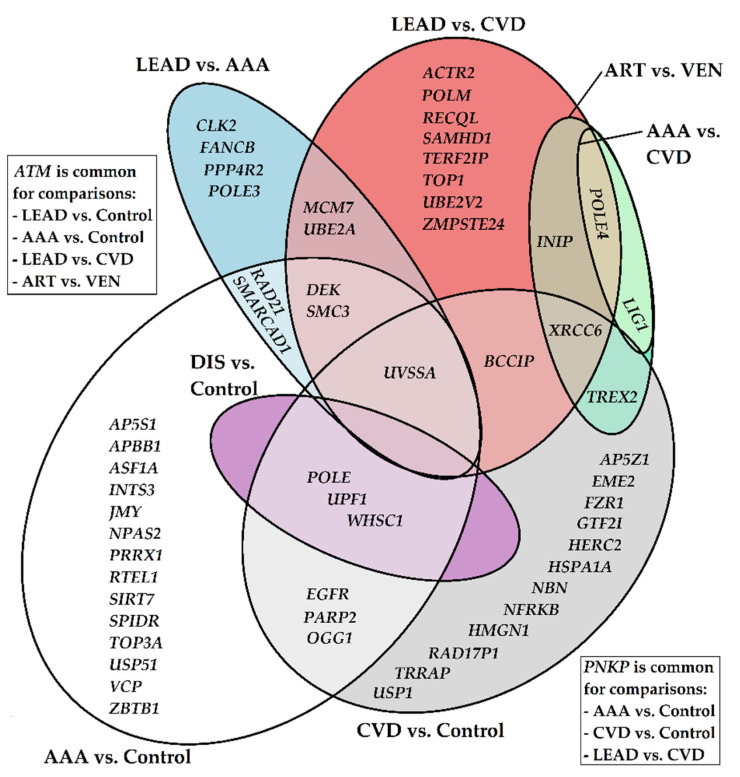
Graphical representation of unique and common genes for gene sets selected from performed comparisons. AAA—abdominal aortic aneurysm, CVD—chronic venous disease, LEAD—lower extremity arterial disease, DIS—pooled group including AAA, CVD and LEAD patients, ART—pooled group of patients with diseases affecting arteries (AAA and LEAD), VEN—patients with disease affecting veins (CVD). Empty fields represent lack of common genes in given comparison(s).

Analysis revealed differentially expressed genes arranged in sets which are characteristic for more than two comparisons studied as well as unique ones for given comparisons (Figure 3). Throughout all comparisons, the most frequently occurring, differentially expressed genes were *ATM* and *UVSSA* (common for four comparisons). Correspondingly, there were groups of genes with less pronounced differential expression, common for three comparisons (gene names, and the most frequently occurring synonyms in brackets): *PNKP, DEK, POLE, SMC3, UPF1, WHSC1 (NDS2), XRCC6 (Ku60), POLE4,* and for two comparisons *EGFR, RAD21, OGG1, PARP2, SMARCAD1, BCCIP, TREX2, MCM7, UBE2A, INIP (SSBIP1), LIG1.*

Additionally, a large group of differentially expressed genes that are unique for performed comparisons was found as a result of analysis. Out of them, fourteen (*AP5S1, APBB1 (FE65), ASF1A, INTS3, JMY, NPAS2, PRRX1, RTEL1, SIRT7, SPIDR, TOP3A, USP51, VCP (p97), ZBTB1*) were differentiating the AAA and control groups; twelve (*AP5Z1, EME2, FZR1, GTF2I (TFII–I), HERC2, HSPA1A, NBN (NBS1), NFRKB, HMGN1, RAD17P1, USP1, TRRAP*) were exclusively characteristic of the CVD and control group comparison; and eight (*ACTR2 (ARP2), POLM, RECQL, SAMHD1, TERF2IP (RAP1), TOP1, UBE2V2 (MMS2), ZMPSTE24 (STE24)*) had a dissimilar pattern of expression between the LEAD and CVD groups, while only four (*CLK2, FANCB, PPP4R2, POLE3*) distinguished LEAD from AAA patients. Their gene products are mainly related to responsibility for various DNA repair-related and genetic information maintenance processes, such as non-homologous end joining (NHEJ), double-strand breaks (DSBs) repair, transcription, DNA replication and chromosome maintenance (Table S5). Additionally, analysis of the literature cited in the current publication has revealed a secondary, more general set of ascribed functions, such as ubiquitination and deubiquitination, kinase activity, circadian rhythm regulation, or histone modification and chromatin remodeling. Some of those genes have already been associated with various processes important in vascular physiology. Additional information regarding presented genes, including brief descriptions of related terms and processes, is placed in the Discussion section below and in Table S5.

### 2.3. Functional Analysis of Selected Genes

Functional analysis was performed for selected genes using two approaches: overrepresentation analysis and gene set enrichment analysis (GSEA). Overrepresentation analysis was performed to functionally annotate upregulated and downregulated gene sets selected from every comparison using the Database for Annotation, Visualization and Integrated Discovery (DAVID) v2021 [27,28] and the following categories for annotation: gene ontology (biological process, molecular function, cellular compartment), KEGG (Kyoto Encyclopedia of Genes and Genomes) and reactome. Functional terms with *p* value < 0.05 were taken into account (supplementary Figure S2).

The results showed that every comparison has been enriched with common terms like “DNA repair”, “DNA metabolic process”, “cellular response to DNA damage stimulus” and “cellular response to stress”, among others associated with nucleic acid metabolism. Differences in more specific terms were noticed between comparisons. Downregulated genes in the AAA vs. control group were primarily associated with “regulation of DNA metabolic process” (*p* = 7.9 × 10^−10^), “double-strand break repair” (*p* = 2.1 × 10^−8^), “regulation of response to DNA damage stimulus” (*p* = 3.3 × 10^−8^), “regulation of DNA repair” (*p* = 2.1 × 10^−7^) but also “chromosome organization” (*p* = 2.3 × 10^−8^), while upregulated genes were connected to “double-strand break repair” (*p* = 4.9 × 10^−13^), “regulation of double-strand break repair” (*p* = 7.9 × 10^−7^) and terms related to homologous recombination, such as “HDR through Homologous Recombination (HRR)” (*p* = 1.9 × 10^−5^) and the regulation of the DNA repair process, such as “regulation of DNA metabolic process” (*p* = 1 × 10^−5^) and “regulation of response to DNA damage stimulus” (*p* = 2.3 × 10^−5^). Downregulated genes in the CVD vs. control group were annotated to terms “double-strand break repair” (*p* = 3.4 × 10^−3^) and also terms connected to regulation, such as “regulation of DNA repair” (*p* = 3.4 × 10^−3^) and “regulation of DNA metabolic process” (*p* = 1.4 × 10^−3^), whereas upregulated genes represented terms like “double-strand break repair” (*p* = 1.7 × 10^−5^), “nucleotide excision repair” (*p* = 4.2 × 10^−6^) and various terms connected to “regulation of DNA repair” (*p* = 2.2 × 10^−4^). Downregulated genes in the LEAD vs. AAA group were primarily associated with “nucleoplasm” (*p* = 4.4 × 10^−2^), “double-strand break repair” (*p* = 0.0014) and “response to radiation” (*p* = 4.9 × 10^−2^), while upregulated ones, apart from terms connected to “double-strand break repair” (*p* = 1.9 × 10^−6^) and “regulation of double-strand break repair” (*p* = 0.001), were also associated with terms regarding “chromosome organization” (*p* = 3.2 × 10^−5^), “histone modification” (*p* = 6.2 × 10^−4^), and “chromatin remodeling” (*p* = 0.003). Upregulated genes in the LEAD vs. CVD group were ascribed to terms like “double-strand break repair” (*p* = 3.2 × 10^−10^), “DNA recombination” (*p* = 1.8 × 10^−6^) and “double-strand break repair via homologous recombination” (*p* = 1.5 × 10^−4^). Moreover, terms associated with various DNA repair regulatory processes, such as “regulation of DNA metabolic process” (*p* = 2.7 × 10^−5^) or “regulation of double-strand break repair” (*p* = 9.7 × 10^−5^) were present. Characteristic associations of downregulated genes from this comparison were, for example, “double-strand break repair via nonhomologous end joining” (*p* = 7.3 × 10^−5^) and “non-recombinational repair” (*p* = 2.55 × 10^−4^). Single genes from the LEAD vs. control and AAA vs. CVD comparisons were associated with terms like “histone mRNA catabolic process”, *ATM*; “Nucleotide Excision Repair”, “Base excision repair” and “Mismatch Repair”, *LIG1*; and “Nucleotide Excision Repair”, “HDR through Homologous Recombination (HRR) or Single Strand Annealing (SSA)”, “DNA Double-Strand Break Repair”, *POLE4*.

**Figure 4:**
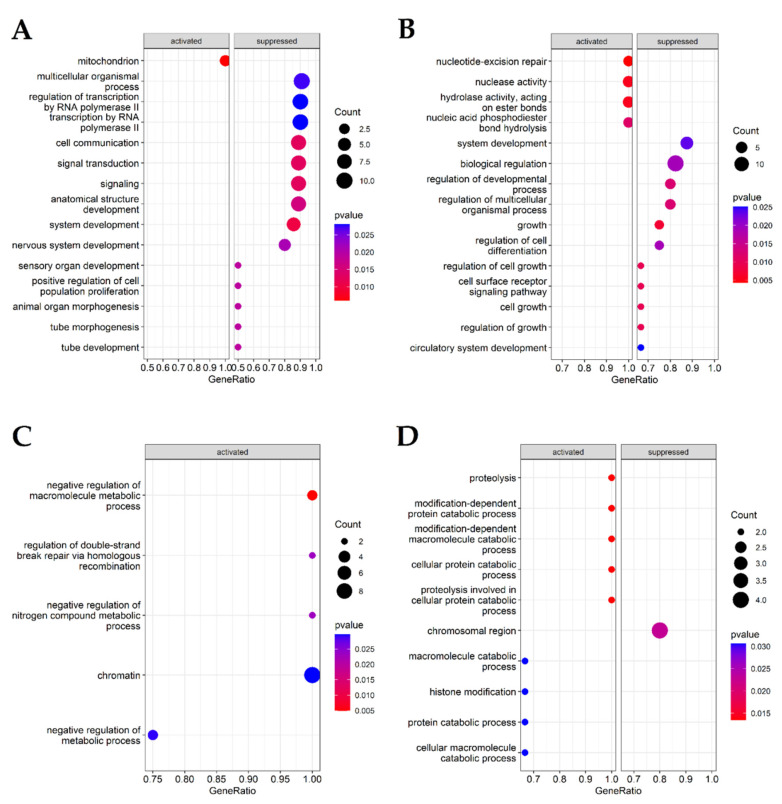
Results of gene set enrichment analysis (GSEA) analysis for genes selected from such comparisons, where more than ten genes were found to be indicative and differentially expressed. Up to the top 15 functional terms with the lowest *p* value are presented. (**A**)—AAA vs. control; (**B**)—CVD vs. control; (**C**)—LEAD vs. AAA; (**D**)—LEAD vs. CVD. AAA—abdominal aortic aneurysm, CVD—chronic venous disease, LEAD—lower extremity arterial disease.

Gene set enrichment analysis (GSEA) was performed for gene ontology categories in R environment using package clusterProfiler v 4.4.4. (https://bioconductor.org/packages/release/bioc/html/clusterProfiler.html) (accessed on 12 July 2022) [29,30]. In order to obtain reliable data output, analysis was conducted for fold change-ordered gene sets selected from only those comparisons where more than ten genes were selected, namely AAA vs. control, CVD vs. control, LEAD vs. AAA and LEAD vs. CVD. Functional terms with *p* below 0.05 were harvested (supplementary Figure S3) and up to the top 15 functional terms with the lowest *p* value are presented on Figure 4.

In AAA vs. control, GO categories associated with transcription and regulation of transcription were downregulated 1.86-fold, whereas GO categories connected to “DNA–templated DNA replication” and “mitochondrion” were upregulated 1.64- and 1.68-fold, respectively. CVD vs. control categories describing nucleotide-excision repair were upregulated 1.77–1.98-fold, while those indicating various cell growth-related processes and their regulation were downregulated 1.91- to 1.98-fold. There were only upregulated GO categories in LEAD vs. AAA, including “regulation of double-strand break repair via homologous recombination” and “chromatin” with fold changes of 1.68 and 1.64, respectively. In LEAD vs. CVD, a cluster of more general categories emerged, such as “histone modification” (which increased 1.6-fold) or “proteolysis”; various references to catabolic processes and regulation were upregulated 1.81-fold.

### 2.4. Relationships between Gene Expression Patterns and Characteristics of the Study Groups of Patients from Performed Comparisons

Age, BMI, gender and smoking status (never and former smokers vs. current smokers) are all risk factors for cardiovascular diseases; thus, it is difficult to find a characteristics-matched group of control subjects. Due to differences in those traits between control and patient groups, statistical analysis regarding those traits was performed. Spearman rank correlation test was used in the analysis of continuous variables (age, BMI), while a two-sided Mann–Whitney *U* test was used in the analysis of categorical variables (gender and smoking status). A statistical significance of *p* < 0.05, corrected by the Benjamini–Hochberg false discovery rate, was set as a selection criterion for both tests. Genes selected from the correlation analysis are presented in Table 3 (the entire correlation results are provided in Table S6). *PRRX1, USP51, VCP* were correlated, albeit moderately, with both age and BMI in the AAA vs. control group. Apart from that, single genes were also moderately correlated either with BMI or age in different comparisons. Therefore, the differential character of their expression patterns may be determined to some extent by differences in age and BMI between studied groups (Table 1). There were only few statistically significant relationships regarding categorical characteristics. *APBB1* (*p* = 0.036) expression levels were correlated with smoking status and *POLM* (*p* = 0.015) and *XRCC6* (*p* = 0.023) with gender (Table S7).

## 3. Discussion

DNA has the most important biological function; it bears the information of a blueprint of the living entity. Despite that, this crucial biological polymer is under constant threat of damage through the action of a plethora of chemical and physical factors.

It was earlier established that some DNA repair-related genes and their protein products are also engaged in various vascular-related processes, such as vascular endothelial growth factor A (VEGFA) function (*RAD21*) [31], muscle stem cell activation (*DEK*) [32], atherosclerosis (*MORF4L2* [33], *FZR1* [34]), neointimal formation (*SIRT7*) [35] and foam cell formation (*USP10*) [36], and have been associated with cardiovascular risk factors (*TRRAP* [37], *NPAS2* [38]). This prompted the present research focusing on similarities and differences in expression of 519 genes known to be related to DNA repair and other interconnected processes (Table S1). Out of them, 59 genes were identified as differentially expressed in at least one disease (Table 2). The transcriptional landscape of mentioned genes was assessed in PBMCs obtained from AAA, CVD and LEAD patients as well as from healthy volunteers included in the control group. Both pairwise and pooled groups comparisons were analyzed (AAA vs. control; CVD vs. control; LEAD vs. control; AAA vs. CVD; LEAD vs. AAA; LEAD vs. CVD and LEAD; AAA and CVD vs. control; LEAD and AAA vs. CVD). From each of those, genes resulting from DESeq2 (with Benjamini–Hochberg FDR *p* < 0.05) and indicated by UVE-PLS to be informative were selected. Relationships between the expression of selected genes and age, BMI, gender and smoking habits were also assessed.

All additional comments and information regarding genes described in the main text, along with an introductory description of DNA damage metabolism pathways and extended discussion, were placed in supplementary text section. Analysis pointed especially to genes contributing to reactive oxygen species’ response and DNA double-strand break repair as the most prominent in the mentioned diseases.

### 3.1. Gene Ontology (GO) and Gene Set Enrichment Analysis (GSEA) Reveals Terms and Categories Associated with Biological Process Regulation, DNA Repair Regulation, Double-Strand Break Repair and Homologous Recombination, Differentiating AAA, CVD and LEAD

Downregulated genes in the AAA vs. control group were primarily associated with various terms related to the regulation of DNA metabolism and repair, but also to transcription and the regulation of transcription, which may indicate decreased DNA repair activities associated with the transcription of genes required for DNA repair. On the other hand, terms describing the regulation of double-strand break repair, nucleotide-excision repair and various terms related to homologous recombination were overrepresented, leading to assumption that DSBs may be pronounced in AAA.

In CVD vs. control, genes ascribed to term “double-strand break repair” and “regulation of DNA repair” form two distinct groups, both upregulated (*TRRAP, PNKP, OGG1, NFRKB* for “regulation of DNA repair” and *AP5Z1, TRRAP, EME2, PNKP, OGG1, ARID2* for “double-strand break repair”) and downregulated (*USP1, EGFR, GTF2I* for “regulation of DNA repair” and *XRCC6, PARP2, RAD21, NBN* for “double-strand break repair”) for each term, different in composition. This could be indicative of different modes of regulation of DNA repair present in CVD patients. Downregulated genes in the LEAD vs. AAA group were primarily associated with the replication of DNA, and “regulation of DNA metabolic process”. The term “double-strand break repair”, similarly to CVD, had ascribed both upregulated (*PPP4R2, RAD21, SMARCAD1, DEK, FANCB*) and downregulated (*MCM7, PNKP*) groups of genes. This again points to DSBs and regulation of their repair as prominent features in those two diseases. Upregulation of terms connected to histone modification and chromatin may reveal chromatin remodeling processes distinguishing LEAD and AAA. While upregulated genes in LEAD vs. CVD were ascribed to terms like double-strand break repair and homologous recombination, there was significant downregulation of genes responsible for repair of DSBs via the NHEJ pathway, indicating that HR is the main pathway of DSBs’ repair in LEAD, in comparison with CVD.

Gene set enrichment analysis (GSEA) was performed for comparisons where more than ten genes were shown as differentiating, namely: AAA vs. control, CVD vs. control, Lead vs. AAA and LEAD vs. CVD. Results are shown in Figure 4, and in supplementary Figure S3. In AAA vs. control group, pathways concerning various aspects of RNA and transcription and “positive regulation of cell population proliferation” were suppressed; whereas, telomere and mitochondrion processes were activated, which may indicate a decrease in metabolic activity caused by senescence. In CVD vs. control, the nucleotide excision repair pathway with accompanying enzymatic activities was activated, whereas general pathways were suppressed, suggesting elevated NER pathway activity affecting wide-scale regulation of biological processes. In the case of LEAD vs. AAA comparison, GSEA found terms ascribed to activated pathways, including “chromatin” and “regulation of double-strand break repair via homologous recombination”, which may indicate that those pathways distinguish LEAD and AAA. In the LEAD vs. CVD comparison, more general pathways were revealed, such as activated ones connected to proteolysis, and suppressed ones, such as the “chromosomal region”. This may be indicative of more general differences present in LEAD and CVD which require in-depth analysis. Such analysis is provided in the discussion below and in the supplementary text.

### 3.2. Expression of DNA Double Strand Break Repair Genes Is Altered in Vascular Diseases

Due to their detrimental effects and toxicity, double-strand breaks require rapid detection, safeguarding and prompt repair. This forces the mutual action of many proteins. The repair process is not only directed at enzymatic activities processing damaged DNA; it is also highly regulated by accessory proteins [39,40].

Three gene-encoding products possessing various enzymatic activities needed in HR were shown to be differentially expressed and characteristic for multiple comparisons, namely *PNKP, POLE, UPF1* (Table S5).

Protein products of those genes contribute to the processing of free DNA ends (*PNKP*) [41], the sensing and resection of DNA in DSBs (*POLE*) [42,43] and the possessing DNA/RNA helicase function in the resection of DNA in DSBs (*UPF1*) [44]. REQL is a helicase preventing replication fork collapse during replication stress, which is needed for genomic stability [45,46]. Contrary to *PNKP, POLE* and *UPF1*, *REQL* has been found as upregulated only in LEAD vs. CVD.

The second group of genes involved in DSB repair is composed of gene-encoding proteins engaged mainly in DNA binding, with less consistent expression changes through the compared groups. It includes *XRCC6 (Ku70)*, *EGFR*, *INIP (SSBIP1)* and *INTS3*. Proteins encoded by these genes play a critical role in efficient HR–dependent repair of DSBs and ATM-dependent signaling pathways [47,48].

It is tempting to assume that revealed dependencies in expression changes could be indicative of diverse regulation of the same process in various diseases, creating the possibility of targeted therapies and diagnosis.

The third group of genes consists of genes responsible for the regulation of the DSB repair process. It includes *PARP2, SMARCAD1, ARID2 (Baf200)* (in a set of genes common for at least two comparisons) and *AP5S1, APBB1 (FE65), ASF1A, SIRT7, SPIDR, USP51* and *VCP (p97)* (unique for the AAA vs. control group comparison) and *SAMHD1* (exclusive to the LEAD vs. CVD comparison).

The AAA vs. control group comparison was the richest in uniquely differentially expressed genes (eighteen positions). Among them, eight genes were ascribed to regulation of the DSBs repair process, on the basis of cited literature (*AP5S1, APBB1, ASF1A, SIRT7, SPIDR, USP51, VCP*).

An increase in the activity of the SIRT7 gene points to increased DSB repair [49], suggesting an alleviating action in AAA patients; meanwhile, a decrease in said gene expression may be responsible for impairing strand-break repair and inducing senescence in patients’ cells, potentially aggravating the course of AAA.

Elevated expression of *SPIDR* may indicate fine tuning and regulation of homologous recombination [50,51] in AAA patients.

DNA DSBs repair by homologous recombination is facilitated by SAMHD1 through promotion of DNA ends resection [52]. Its expression is upregulated in LEAD vs. CVD patients, again suggesting not only distinct regulation of homologous recombination, but also inclusion of inflammation [53] in LEAD and CVD.

There were other genes present in sets common for at least two comparisons (*BCCIP*, *LIG1*) and unique for only one comparison (*RTEL1*, *TOP3A*, *EME2*, *FANCB*) contributing to homologous recombination [54,55,56,57,58,59,60,61].

The composition of transcriptional patterns points to homologous repair as the main DSBs repair mechanism predominant in AAA, but occurring also in both LEAD and CVD. 

To further support engagement of homologous repair of DSB in the presented diseases, non-homologous end joining-related gene (*POLM, ASF1A, ERCC6L2, USP51*) transcription was examined [62,63,64,65,66].

All NHEJ-related gene transcription was decreased, suggesting a shift to HR as the main mechanism of DNA DSBs in AAA patients.

Interestingly, genes identified by the current analysis were engaged more in different modes of DSBs repair regulation than in specific enzymatic activities. This allows us to speculate that the regulation of basic processes of DNA repair could be characteristic of given vascular disease, reflecting different onset, progression and specific presentations, while the main enzymatic activities proceeding HR remain unaltered. This could be a prerequisite for targeted treatment and/or discriminative diagnosis. Counterintuitive though may it seem, elevated activity of HR may promote excess DNA damage due to a rise in free DNA end resection which thus causes an increase in DNA damage intermediates known to cause DNA damage when unrepaired [67,68]. This is in line with the fact that intensified DNA damage may aggravate presentations of vascular diseases.

### 3.3. Specific Gene Expression Changes May Be Indicative for Altered Oxidative DNA Damage Responses in AAA, CVD and LEAD

Oxidative stress is both the crucial hallmark of cardiovascular diseases and their causative factor [69]. The MRGX protein was suggested to possess a role in defense against reactive oxygen species (ROS) [70]. MRGX, along with *ERCC5 (XPG)* gene, encodes protein taking part in NER and HR [71,72]. Downregulation of MRGX may indicate impaired response to elevated oxidative stress, especially in AAA and CVD patients. Analysis of transcriptional patterns of genes presented in this research is supported by the findings of other groups that oxidative stress is the prominent feature of many vascular diseases, including those assessed in the current paper.

### 3.4. Analysis Reveals Known Genes Being Involved in Vascular Diseases’ Initiation and Progression

Cardiovascular diseases have long been described as ‘multifactorial’ [73], involving a plethora of genetic and environmental factors contributing to the overall disease outcome.

Gene contribution revealed in our studies and according to the literature analysed varies from the regulation of a vascular-specific processes to commitment to a specific risk factor or cardiovascular system defects. The transcription pattern is also variable, ranging from four comparison occurrences (*ATM and UVSSA*) to exclusiveness to only one comparison.

Protein products of those genes take part in signal transduction from vascular endothelial growth factor (VEGF) receptors (RAD21, [39]), muscle stem cell activation (DEK [40]), the promotion of plaque instability through accelerated senescence (MORF4L2 [41]) and the regulation of inflammation and proliferation of cells (TREX2 [74]). The OGG glycosylase is the main glycosylase; it removes the most abundant DNA lesion resulting from oxidation, 8–oxoG, possessing not only enzymatic functions, but also contributing to cellular signaling and epigenetic-like pathways [75,76,77]. It has a dual action; on the one hand, it is an important modulatory factor restricting inflammatory responses and apoptosis in macrophages preventing atherosclerosis [78], while on the other it has the ability to upregulate pro-inflammatory gene expression [77]. Thus, elevated expression of *OGG1* in AAA and CVD may have ambiguous effects: it may be benevolent through alleviation of DNA damage or promote inflammation through its ability to bind to DNA and upregulate pro-inflammatory gene expression [77].

Gene sets correlated with both vascular-related processes/terms and DNA repair, unique for one comparison, only had the following composition. In AAA patients (AAA vs. control group) there were six genes with differential expression: *NPAS2 (MOP4), PRRX1, RTEL1, SIRT7, USP10, VCP (p97)*. In the LEAD vs. CVD group, there were three such genes: *ACTR2 (ARP2), SAMHD1, TERF2IP (RAP1), HERC2.* In CVD patients (CVD vs. control group), there were two characteristic genes: *HSPA1A, TRRAP;* only one appeared in the LEAD vs. AAA comparison (*CLK2*).

Interestingly, besides correlation with known vascular diseases and DNA repair, some genes point to the circadian oscillator being an important element of those conditions: *NPAS2* [79], *HERC2* [80] and *CLK2* [81]. This may suggest some interesting therapeutic opportunities, such as application of chronotherapy, in vascular diseases. This approach takes into account the daily oscillation of various metabolic processes governing uptake, transport and activation of medicines in order to apply different treatment regimens for the best outcome [80].

- AAA vs. control group: *NPAS2* (*MOP4*), *PRRX1*, *RTEL1*, *SIRT7*, *USP10*, *VCP* (p97)

Pathological vasculogenesis and arteriolar remodeling are hallmarks of different vascular diseases; both PPRX and RTEL1 are known to be engaged in those processes [82,83], and their altered expression may influence AAA presentation in patients.

The *SIRT7* gene product shapes VSMCs proliferation and migration in neointimal formation following vascular injury [84], while USP10 dysregulation may affect VSMC-derived foam cells formation also in AAA [85,86]. Moreover, elevated levels of VCP expression may facilitate the stability of the angiotensin type 1 receptor and enhance Ang II blood pressure elevating effects in AAA patients [87,88].

- LEAD vs. CVD group: *ACTR2* (*ARP2*), *SAMHD1*, *TERF2IP* (*RAP1*)

These genes are associated with various processes and correlated with various vasculopathies. They contribute to pathological cell motility, enhancing neointima formation after vascular injury (ACTR2 [89]), cerebral vasculopathy in Aicardi–Goutieres syndrome (SAMHD1 [90,91,92]) and cells’ activation, senescence and apoptosis (TERF2IP [93]).

- CVD vs. control group: HSPA1A (HSP70), TRRAP

Decreased expression of *HSPA1A* may be an element of compromised tolerance for stresses present in the course of CVD, compromising defense mechanisms including DNA repair [94]. The TRRAP protein is engaged in the regulation of lipid metabolism through stimulation of lipid droplet formation [37], and positively regulates cholesterol metabolism and intake [95].

### 3.5. Chromatin Remodelling Could Be the Process Shaping and Regulating DNA Damage Responses in Vascular Diseases

DNA in eukaryotes tightly interacts with many specialized proteins, such as histones, building a highly organized entity called chromatin. Current NGS analysis did detect some of differentially expressed genes whose products are needed in various chromatin remodeling processes. In group of genes ascribed to more than one comparison, those were *ATM, POLE* and *POLE4, WHSC1 (NDS2), SMARCAD1, ARID2 (Baf200)*.

The ATM kinase is the node factor in a vast biochemical network of reciprocal interactions, signaling the presence of DNA damage and controlling the repair process [96]. Recently, it was discovered that two of subunits (POLE3 and POLE4) are histone H3–H4 chaperones, maintaining chromatin integrity during DNA replication [97]. Another gene connected to posttranslational modifications of histones, encoding histone methyltransferase, is *WHSC1*; meanwhile, HMGN1 binds to 147-bp nucleosome core particle in purely structure-dependent manner [98] and facilitates UV light and ionizing irradiation-induced DNA damage [99,100]. Altered expression of genes encoding proteins belonging to chromatin-remodeling complexes, such as ARID2 [101] ACTR2 [102,103] and NRFKB [104], has also been observed in our transcriptomic data.

Differential expression of genes related to chromatin remodeling clearly indicates the important contribution of this process in the regulation of DNA damage in the presented vascular diseases. What is interesting, and could be assumed, is that similarly to DNA DSBs repair, the regulation of various chromatin–related processes is an important factor to distinguish between vascular diseases. Those differences could have a tremendous impact on disease course and presentation.

### 3.6. Ubiquitination and Deubiquitination Could Be a Prominent Mechanism Regulating Gene Activities in Vascular Diseases

One of potent mechanisms of protein function regulation is ubiquitination, defined as a covalent binding of ubiquitin protein to various other proteins. Current NGS analysis did detect some of differentially expressed genes, whose products are needed in various ubiquitin-related processes. Those genes were *UBE2A, USP10, USP51, ZBTB1, HERC2*, and *SHPRH*.

The *UBE2A (RAD6A)* gene encodes an enzyme whose function is monoubiquitination of PCNA and taking part in DNA damage response to ionizing radiation [105,106]. Upregulation of *UBE2A* was characteristic of LEAD in comparison with AAA and CVD.

USP10 and USP51 are deubiquitinases with increased expression in AAA patients. USP10 deubiquitinates and stabilizes MutS Homolog 2 (MSH2), which is the key DNA mismatch repair protein [107]. USP51 is a histone H2A deubiquitinase whose depletion results in the impairment of DNA damage response [44]. The ZBTB1 protein regulates monoubiquitinatination of proliferating cell nuclear antigen (PCNA) at DNA damage sites [108]. Expression of ZBTB1 was decreased in AAA patients.

The HERC2 protein is an E3 ubiquitin ligase negatively regulating both HR and NER [80,109]. Upregulation of this gene is present in CVD patients, suggesting that ubiquitination alterations may have a characteristic pattern in this disease.

Presented results show a connection between DNA damage responses and ubiquitin metabolism, showing possible different modes of regulation of DNA damage. Moreover, these seem to be specific for presented diseases, having potential value in both specific diagnosis and treatment.

### 3.7. Limitations of the Study and Accompanying Issues

Undoubtedly, the descriptive character of our study raised some limitations and additional questions. We are aware that a bias concerning age, BMI, sex and smoking exists in our data. The expression levels of the presented genes may be affected by those characteristics; thus, differential expression of genes could not be exclusive to the disease status, but their expression levels could be affected by the mentioned factors, and statistically significant differences between these variables could be observed (Table 1). Coherently, obtained results could be disrupted and undeniably, some relationships between picked genes and age and BMI can be found (Table 3). The main reason for their appearance is the groups’ construction. First and foremost, during control group construction, we have adhered to the selection of completely healthy persons with no cardiovascular conditions, which resulted in age, gender and smoking differences. It is clear that the characteristics of groups with different vascular diseases reflect their epidemiology; the disease mainly affects persons with predisposing traits. Consequently, age and BMI are well-known risk factors for all vascular diseases; therefore, selected genes could contribute to these diseases through age- and BMI-associated mechanisms [25]. There were only few correlations regarding categorical characteristics. *APBB1* expression levels were correlated with smoking status, and *POLM* and *XRCC6* with gender (Table S7).

The experimental design was initially focused on NGS analysis of the whole transcriptome and mirnome of patients [23,24,25], with subsequent determination of commonalities and dissimilarities present in the transcriptome of AAA, CVD and LEAD cases [25]. On this basis, our attention later converged on more specific processes such as DNA damage metabolism. PCR validation of our data was extremely difficult due to the fact that we were unable to predict the final number of genes resulting from analysis. That information was crucial for the design of the PCR experiment (e.g., the amount of material needed, the number of particular probes and the amount of master mix to purchase). For the same reasons, no protein expression validation, such as ELISA or Western blot, was conducted. Therefore, in order to recompensate the lack of qPCR validation and protein detection assays, broader and more advanced statistical analysis (DESeq2 with Benjamini–Hochberg false discovery rate correction, confirmed by UVE-PLS and ROC analysis) was applied, which substantially limited potential false positive results.

The number of screened patients could be considered low; however, in analogous research conducted so far, one can observe similar or even lower quantities of screened subjects. Moreover, there are a great variety of approaches to cardiovascular disease assessment. These issues are discussed in detail in the supplementary material in [22]. Interestingly, St. Laurent et al. discovered that most of statistical methods can detect reliable transcripts even when as few as three biological replicates are sequenced using single-molecule based RNAseq [110]. Despite all of the above, we are aware that a higher number of screened patients may be beneficial in our future research for discovering new disease-associated patterns in complicated processes such as DNA damage repair.

The typical subjectively chosen threshold for changes in gene expression is about 2–3 fold. St. Laurent et al. shed some light on this issue and drew the conclusion that even though a plethora of differentially expressed RNAs exhibit fold changes less than two, they still retain viable and biologically relevant information. This happens even at very low fold changes, and exclusion of those mRNAs may cause a loss of data and a needless drain on the discovery process [110]. Moreover, additive effects affecting the physiological response may be present in genes with low fold change in expression. Additionally, many of the indicated genes have regulatory function in DNA repair, so may exhibit big biologically relevant effects even with small expression changes. This is why it was finally decided that the terminal gene set for all analyses would contain genes with fold changes ranging from 0.8 (downregulated) to 1.2 (upregulated).

We are aware that the presented studies mainly have an initial and descriptive character, and we do not imply that the presented results and interpretations are definitive. Conclusions deduced from established gene sets need to be confirmed in the future by more detailed experiments. The explanatory character of transcriptomic landscape of DNA damage metabolism in vascular diseases and its role in pathology should be elucidated in detail in further studies. Presented results could be reassessed by other techniques like qPCR, flow cytometry, Western blot, gene expression modification in model systems by means of genetic engineering, and experiments with animal models and human cells or artificial tissues. These could be accompanied by techniques utilized by traditional DNA repair research, such as comet assays, micronuclei indexes, DNA modifications detection and oxidative stress assessment. Moreover, subsequent validation studies should encompass much larger and character-balanced populations. Similarly to preceding publications, we wished to share the findings of the current research to give the opportunity to start a discussion within the scientific community, to propose new, explorative paths for other research groups [25] and to broaden knowledge about DNA repair in the context of vascular diseases.

## 4. Materials and Methods

### 4.1. Study Groups Participants Characteristics

The study group consisted of four assemblages: LEAD (8 patients), AAA (7 patients), CVD (7 patients) and control group of healthy volunteers (7 persons). All subjects were assessed by a vascular surgeon in Independent Public Clinical Hospital No. 1 in Lublin. All persons gave informed and signed consent. All mentioned procedures were performed in accordance with the Declaration of Helsinki. The Ethics Committee of Medical University of Lublin approved the study design (resolution No. KE–0254/341/2015). The clinical characteristics regarding all participants are presented in Table 1. A detailed description of clinical features, as well as inclusion procedures and exclusion criteria were provided in previous papers [32,33,34].

### 4.2. Gene Expression Datasets Generation

Next-generation sequencing was the method of choice for the assessment of transcriptomic profiles of PBMCs from study participants. An entire detailed methodology with accompanying data regarding sequencing data quality was provided in previous papers [22,23,24].

Briefly, blood samples were subjected to PBMCs isolation using density gradient centrifugation with Gradisol L reagent (Aqua–Med, Łódź, Poland). Concisely, total RNA specimens (for transcriptome analysis) were isolated from PBMCs samples of vascular diseases patients and randomly selected controls using TRI Reagent Solution (Applied Biosystems, Foster City, CA, USA). The quality of total RNA samples was assessed using the Agilent 2100 Bioanalyzer instrument with the Agilent RNA 6000 Pico Kit for total RNA samples and the Agilent DNA 1000 Kit for libraries ([22] and supplementary Figure S20). Subsequently, total RNA preparations were submitted to ribosomal RNA removal with use of RiboMinus Eukaryote System v2 (Ambion, Austin, TX, USA). Transcriptome libraries were prepared using the Ion Total RNA-Seq Kit v2, Magnetic Bead Cleanup Module kit, Ion Xpress RNA-Seq Barcode 01-16 Kit and sequenced on Ion 540 chips (all Life Technologies, Carlsbad, CA, USA) using the Ion S5 XL System (Thermo Fisher Scientific, Waltham, MA, USA). Sequencing reaction of libraries was performed with Ion 540 chips (Life Technologies) using an Ion S5 XL System (Thermo Fisher Scientific, Waltham, MA, USA). An alignment of raw sequences of the hg19 version of the human genome was made with the help of Torrent Suite Software v5.0.4. and the Ion Torrent RNASeq Analysis plugin v.5.0.3.0 (Thermo Fisher Scientific). Raw sequences of transcriptomic libraries were aligned to 55,765 known genes, their splicing variants and the putative open reading frames of hg19 human genome.

### 4.3. Data Analysis Methods and Tools

R environment (R version 4.1.0, https://www.r-project.org) (accessed on 18 May 2021) and appropriate packages according to the provided reference manuals were used for statistical analyses and data visualizations. Kruskal–Wallis rank sum test (kruskal.test function in R) was applied to assess the statistical significance of differences in age and BMI between the LEAD, AAA, CVD and control groups. A two-sided Fisher’s exact test (fisher.test function in R) was applied to assess the statistical significance of differences in gender and smoking status between the studied groups.

Differential expression analysis was performed on data originating from biological replicates. DESeq2 and UVE-PLS (uninformative variable elimination by partial least squares) [111,112] methods were used, and the latter works especially well with partial least squares as a multivariate regression technique fitted for dealing with large sets of noisy and correlated variables as well as with rather small numbers of samples [113].

DESeq2 analysis was carried out using a DESeq2 1.26.0 package [111] (https://bioconductor.org/packages/release/bioc/html/DESeq2.html) (accessed on 18 May 2021) on expression data devoid of genes with mean number of reads lower than one. Differentially expressed genes were considered as statistically significant when the criterion of a *p* value below 0.05 after Benjamini–Hochberg false discovery rate correction was met.

A plsVarSel 0.9.6 package [114] (https://cran.r-project.org/web/packages/plsVarSel/index.html) (accessed on 18 May 2021) was used to perform UVE-PLS analysis. The initial data was transformed by regularized log normalization (rlog function in the DESeq2 package). 1000 iterations, a 0.75 ratio for splitting into trained and tested data subsets and a minimum reliable score equal to 5 cutoff threshold were the values used in UVE-PLS analysis. The number of PLS (partial least squares) components for UVE-PLS analysis (Table A1 in Appendix A) was established by inspecting the estimated root mean squared error of prediction (RMSEP) determined by PLS regression with leave-one-out (LOO) cross-validation (Figures S19 and S20).

The pROC package 1.16.2 [115] (https://cran.r-project.org/web/packages/pROC/index.html) (accessed on 19 May 2021) was used to conduct the ROC analysis.

Boxplots were generated using the ggplot2 3.3.0 package (https://ggplot2.tidyverse.org) (accessed on 29 August 2021).

The statistical significance of categorical characteristics (gender and smoking habits) was assessed with a two-sided Mann–Whitney *U* test implemented in wilcox.test function in R. Statistical significance of continuous clinical features (age and BMI) was performed using a Spearman rank correlation test implemented in the Hmisc package 4.4–0. (https://cran.r-project.org/web/packages/Hmisc/index.html) (accessed on 29 August 2021).

Overrepresentation functional analysis was performed by the Database for Annotation, Visualization and Integrated Discovery (DAVID) (https://david.ncifcrf.gov/home.jsp (accessed on 11 July 2022)) [27,28]. The list of genes resulting from each of comparisons was uploaded, and analysis was run on given settings: Select Identifier—OFFICIAL_GENE_SYMBOL; Select species—Homo sapiens; List Type—Gene List; Annotation Summary Results: No defaults; Gene_Ontology: GOTERM_BP_ALL, GOTERM_CC_ALL, GOTERM_MF_ALL; Pathways: KEGG_PATHWAY, REACTOME_PATHWAY. Selected annotations were combined by functional annotation clustering and placed in supplementary Figure S2.

Gene set enrichment analysis (GSEA) was performed in R environment with package clusterProfiler v4.4.4. (https://bioconductor.org/packages/release/bioc/html/clusterProfiler.html) (accessed on 12 July 2022) [29,30]. Analysis was performed for comparisons where more than 10 genes were selected, namely AAA vs. control, CVD vs. control, Lead vs. AAA and LEAD vs. CVD. Results are shown in supplementary Figure S3.

### 4.4. Advantages of Peripheral Blood Mononuclear Cells’ (PBMCs) Utilization for DNA Damage Metabolism and Vascular Conditions Assessment

Blood mononuclear cells were widely used in the assessment of not only various atherosclerotic risk factors like hypertension [116], type 2 diabetes [117], and hyperlipidemia [118], but also plaque vulnerability itself [119]. The contribution of PBMCs to vascular pathology is now widely accepted [120]. DNA damage is present both in the vascular bed in circulating cells and cells present in the lesions of vascular walls [15]. Exemplary patients with coronary artery disease possess a higher micronuclei index (a marker of genetic instability) than healthy controls, which correlates with disease severity [121]. PBMCs have been confirmed to be a good source of biological material exhibiting evidence of DNA damage in coronary artery disease. Moreover, DNA damage is confirmed in early atherosclerotic lesions and becomes overabundant in advanced plaques [16]. PBMCs encompass biological material which is easily accessible and appropriate for routine clinical practice without using expensive, labor- and time-consuming methods like cell sorting and flow cytometry. One of the goals of this study was to determine markers that could be applied in an easy test for the diagnosis and prediction of vascular diseases. Careful selection of possible tissues favored the use of blood circulating cells as an excellent biological material [22].

## 5. Conclusions

The presented research sheds light on DNA damage repair in patients with three vascular diseases: AAA, CVD and LEAD. In-depth data analysis revealed gene sets that could be ascribed to numerous DNA repair processes, pointing especially to the prominent homologous repair presence in all probed diseases. Data also indicate a significant crosstalk between diverse pathways interrelated with DNA repair, encompassing chromatin remodeling, cohesin metabolism and function and ubiquitin turnover, and oxidative stress presence and management. These characteristics show significant commonalities in processes contributing to the presented vascular diseases. Striking differences appear when the analysis takes into account the regulatory component present in the examined diseases. Data suggest that gene-encoding proteins responsible for control and fine–tuning of mentioned processes exhibit the most interesting dissimilarities in expression. This could be the key that describes and distinguishes one condition from the other, and may explain the relative richness of their clinical presentations and courses. This is also a prerequisite for the creation of new diagnostic methods and therapies utilizing DNA repair as the target of intervention.

## Figures and Tables

**Table 1:** Clinical attributes of the study subjects.

Characteristic	AAA (*n* = 7)	CVD (*n* = 7)	LEAD (*n* = 8)	Control (*n* = 7)	*p* ^1^
Age	66.3 ± 4.03 ^2^ 59–71 ^3^	41.3 ± 4.03 ^2^ 35–47 ^3^	62 ± 7.82 ^2^ 48–71 ^3^	33.6 ± 9.64 ^2^ 24–54 ^3^	6.290 × 10^−5 4^
Body mass index (BMI)	27.23 ± 2.76 ^2^ 23.66–30.85 ^3^	23.36 ± 1.94 ^2^ 20.94–25.83 ^3^	28.25 ± 2.07 ^2^ 25.5–31.2 ^3^	21.73 ± 3.30 ^2^ 19.33–29.00 ^3^	0.448 ^5^
Gender: males/females	6 (85.7%)/ 1 (14.3%)	3 (42.9%)/ 4 (57.1%)	6 (75%)/ 2 (25%)	5 (71.4%)/ 2 (28.6%)	1.564 × 10^−3 4^
Smoking: never and former/current	5 (71.4%)/ 2 (28.6%)	5 (71.4%)/ 2 (28.6%)	6 (75%)/ 2 (25%)	7 (100%)/ 0 (0%)	0.535 ^5^

^1^ Statistical significance of differences between lower extremity arterial disease (LEAD), abdominal aortic aneurysm (AAA) and chronic venous disease (CVD) groups, ^2^ mean ± SD, ^3^ range, ^4^ *p* value calculated using Kruskal–Wallis rank sum test, ^5^ *p* value calculated using two-sided Fisher’s exact test.

**Table 2:** Differential expression parameters of genes selected from the pairwise and pooled group comparisons. The table presents *p* (FDR with Benjamini–Hochberg correction) and fold change values received from DESeq2 analysis, PLS coefficients received from UVE-PLS analysis, and areas under ROC curves (ROC–AUC) received from ROC analysis. Genes were ordered within each comparison according to increasing *p* value.

No.	Gene Symbol ^1^	Gene Name ^1^	*p*	Fold Change	PLS Coefficient	ROC–AUC
LEAD vs. Control group						
1.	*ATM*	ATM serine/threonine kinase	3.969 × 10^−2^	0.762	−5.042 × 10^−1^	1.000
**AAA vs. Control group**						
1.	*RAD21*	RAD21 cohesin complex component	4.926 × 10^−7^	0.757	−7.573 × 10^−2^	1.000
2.	*UPF1*	UPF1 RNA helicase and ATPase	7.004 × 10^−5^	1.286	7.567 × 10^−2^	1.000
3.	*SMARCAD1*	SWI/SNF-related, matrix-associated actin-dependent regulator of chromatin, subfamily a, containing DEAD/H box 1	2.260 × 10^−4^	0.725	−8.092 × 10^−2^	0.959
4.	*WHSC1*	Nuclear receptor-binding SET Domain protein 2	4.903 × 10^−4^	1.300	7.990 × 10^−2^	1.000
5.	*TOP3A*	DNA topoisomerase III alpha	2.659 × 10^−3^	1.320	6.685 × 10^−2^	0.918
6.	*INTS3*	Integrator complex subunit 3	3.802 × 10^−3^	1.227	5.369 × 10^−2^	0.918
7.	*PNKP*	Polynucleotide kinase 3’-phosphatase	3.955 × 10^−3^	1.311	8.177 × 10^−2^	0.959
8.	*PRRX1*	Paired related homeobox 1	3.955 × 10^−3^	0.208	−1.010 × 10^−1^	0.918
9.	*UVSSA*	UV stimulated scaffold protein A	3.955 × 10^−3^	1.482	1.246 × 10^−1^	0.980
10.	*ZBTB1*	Zinc finger and BTB domain containing 1	3.955 × 10^−3^	0.778	−5.801 × 10^−2^	0.918
11.	*PARP2*	Poly(ADP-ribose) polymerase 2	4.303 × 10^−3^	0.692	−5.700 × 10^−2^	0.918
12.	*SMC3*	Structural maintenance of chromosomes 3	4.303 × 10^−3^	0.795	−5.300 × 10^−2^	0.898
13.	*OGG1*	8-oxoguanine DNA glycosylase	8.026 × 10^−3^	1.314	7.994 × 10^−2^	0.918
14.	*ATM*	ATM serine/threonine kinase	8.031× 10^−3^	0.761	−6.130 × 10^−2^	0.918
15.	*EGFR*	Epidermal growth factor receptor	8.031× 10^−3^	0.250	−4.767 × 10^−2^	0.959
16.	*ASF1A*	Anti-silencing function 1A histone chaperone	8.587 × 10^−3^	0.681	−1.029 × 10^−1^	0.959
17.	*RTEL1*	Regulator of telomere elongation helicase 1	9.642 × 10^−3^	1.805	6.994 × 10^−2^	0.939
18.	*APBB1*	Amyloid beta precursor protein-binding family B member 1	1.132 × 10^−2^	0.638	−9.918 × 10^−2^	0.878
19.	*NPAS2*	Neuronal PAS domain protein 2	1.145 × 10^−2^	0.384	−6.987 × 10^−2^	1.000
20.	*DEK*	DEK proto-oncogene	1.163 × 10^−2^	0.794	−6.876 × 10^−2^	0.980
21.	*POLE*	DNA polymerase epsilon, catalytic subunit	1.656 × 10^−2^	1.235	6.854× 10^−2^	0.959
22.	*SIRT7*	Sirtuin 7	1.656 × 10^−2^	1.236	6.185× 10^−2^	0.918
23.	*USP51*	Ubiquitin specific peptidase 51	1.656 × 10^−2^	0.541	−5.382 × 10^−2^	0.959
24.	*AP5S1*	Adaptor-related protein complex 5 subunit sigma 1	2.422 × 10^−2^	1.350	5.630 × 10^−2^	0.959
25.	*SPIDR*	Scaffold protein involved in DNA repair	2.655 × 10^−2^	1.237	4.797 × 10^−2^	0.898
26.	*JMY*	Junction-mediating and regulatory protein, p53 cofactor	2.874 × 10^−2^	0.767	−5.160 × 10^−2^	0.898
27.	*VCP*	Valosin-containing protein	3.095 × 10^−2^	1.209	5.048 × 10^−2^	0.898
**CVD vs. Control group**						
1.	*POLE*	DNA polymerase epsilon, catalytic subunit	2.750 × 10^−4^	1.389	−1.007 × 10^−1^	1.000
2.	*UVSSA*	UV stimulated scaffold protein A	5.268 × 10^−4^	1.608	−1.571 × 10^−1^	1.000
3.	*PNKP*	Polynucleotide kinase 3’–phosphatase	3.628 × 10^−3^	1.324	−9.409 × 10^−2^	0.980
4.	*RAD17P1*	RAD17 pseudogene 1	3.628 × 10^−3^	0.241	6.482 × 10^−2^	0.980
5.	*WHSC1*	Nuclear receptor-binding SET domain protein 2	3.628 × 10^−3^	1.267	−7.529 × 10^−2^	0.980
6.	*XRCC6*	X-ray repair cross complementing 6	3.894 × 10^−3^	0.773	7.724 × 10^−2^	1.000
7.	*TRRAP*	Transformation/transcription domain-associated protein	3.937 × 10^−3^	1.247	−8.331 × 10^−2^	1.000
8.	*AP5Z1*	Adaptor-related protein complex 5 subunit zeta 1	4.601 × 10^−3^	1.319	−1.079 × 10^−1^	0.980
9.	*EME2*	Essential meiotic structure–specific endonuclease subunit 2	4.601 × 10^−3^	1.445	−1.024 × 10^−1^	0.939
10.	*UPF1*	UPF1 RNA helicase and ATPase	4.601 × 10^−3^	1.211	−8.287 × 10^−2^	1.000
11.	*NBN*	Nibrin	7.122 × 10^−3^	0.767	6.012 × 10^−2^	0.980
12.	*USP1*	Ubiquitin-specific peptidase 1	8.767 × 10^−3^	0.786	6.183 × 10^−2^	1.000
13.	*GTF2I*	General transcription factor IIi	1.106 × 10^−2^	0.748	1.004 × 10^−1^	0.980
14.	*HERC2*	HECT and RLD domain containing E3 ubiquitin protein ligase 2	1.291 × 10^−2^	1.229	−5.896 × 10^−2^	0.959
15.	*TREX2*	Three prime repair exonuclease 2	1.291 × 10^−2^	2.039	−5.716 × 10^−2^	0.959
16.	*HMGN1*	High-mobility group nucleosome-binding domain 1	1.303 × 10^−2^	0.760	1.011 × 10^−1^	1.000
17.	*EGFR*	Epidermal growth factor receptor	1.396 × 10^−2^	0.244	4.115 × 10^−2^	0.918
18.	*OGG1*	8-oxoguanine DNA glycosylase	2.368 × 10^−2^	1.282	−7.221 × 10^−2^	0.898
19.	*PARP2*	Poly(ADP-ribose) polymerase 2	2.490 × 10^−2^	0.725	5.284 × 10^−2^	0.918
20.	*NFRKB*	Nuclear factor related to kappaB-binding protein	2.618 × 10^−2^	1.249	−9.573 × 10^−2^	0.980
21.	*BCCIP*	BRCA2 and CDKN1A interacting protein	3.014 × 10^−2^	0.788	6.583 × 10^−2^	0.939
22.	*FZR1*	Fizzy and cell division cycle 20 related 1	3.418 × 10^−2^	1.250	−9.201 × 10^−2^	0.980
23.	*HSPA1A*	Heat shock protein family A (Hsp70) member 1A	3.418 × 10^−2^	0.533	1.376 × 10^−1^	0.898
**LEAD vs. AAA**						
1.	*PPP4R2*	Protein phosphatase 4 regulatory subunit 2	2.732 × 10^−6^	1.335	1.229 × 10^−1^	1.000
2.	*RAD21*	RAD21 cohesin complex component	9.034 × 10^−6^	1.272	1.049× 10^−1^	1.000
3.	*SMC3*	Structural maintenance of chromosomes 3	7.431 × 10^−4^	1.294	1.195 × 10^−1^	0.964
4.	*POLE3*	DNA polymerase epsilon 3, accessory subunit	5.547 × 10^−3^	1.326	1.340 × 10^−1^	0.946
5.	*MCM7*	Minichromosome maintenance complex component 7	9.869 × 10^−3^	0.786	−1.065 × 10^−1^	0.911
6.	*CLK2*	CDC like kinase 2	1.350 × 10^−2^	0.774	−1.063 × 10^−1^	0.946
7.	*DEK*	DEK proto–oncogene	1.350 × 10^−2^	1.274	1.133 × 10^−1^	0.929
8.	*SMARCAD1*	SWI/SNF–related, matrix–associated actin–dependent regulator of chromatin, subfamily a, containing DEAD/H box 1	1.350 × 10^−2^	1.265	1.110 × 10^−1^	0.964
9.	*UVSSA*	UV stimulated scaffold protein A	1.350 × 10^−2^	0.703	−1.890× 10^−1^	0.875
10.	*FANCB*	FA complementation group B	2.563 × 10^−2^	1.598	9.226 × 10^−2^	0.929
11.	*UBE2A*	Ubiquitin conjugating enzyme E2 A	3.975 × 10^−2^	1.291	1.428 × 10^−1^	0.911
**LEAD vs. CVD**						
1.	*UVSSA*	UV-stimulated scaffold protein A	2.430 × 10^−3^	0.648	−1.885 × 10^−1^	0.893
2.	*MCM7*	Minichromosome maintenance complex component 7	5.487 × 10^−3^	0.771	−9.060 × 10^−2^	0.964
3.	*ACTR2*	Actin-related protein 2	7.278 × 10^−3^	1.290	6.713 × 10^−2^	0.946
4.	*POLM*	DNA polymerase mu	7.278 × 10^−3^	0.774	−8.552 × 10^−2^	0.946
5.	*PNKP*	Polynucleotide kinase 3′–phosphatase	1.780 × 10^−2^	0.793	−6.935 × 10^−2^	0.946
6.	*UBE2A*	Ubiquitin conjugating enzyme E2 A	1.780 × 10^−2^	1.327	6.779 × 10^−2^	0.893
7.	*DEK*	DEK proto–oncogene	1.795 × 10^−2^	1.256	5.554 × 10^−2^	0.875
8.	*INIP*	INTS3 and NABP interacting protein	1.795 × 10^−2^	1.283	6674 × 10^−2^	0.929
9.	*XRCC6*	X–ray repair cross complementing 6	1.795 × 10^−2^	1.239	7.014 × 10^−2^	0.911
10.	*UBE2V2*	Ubiquitin conjugating enzyme E2 V2	1.820 × 10^−2^	1.250	7.772 × 10^−2^	1.000
11.	*BCCIP*	BRCA2 and CDKN1A interacting protein	1.992 × 10^−2^	1.271	8.439 × 10^−2^	0.964
12.	*ATM*	ATM serine/threonine kinase	2.223 × 10^−2^	0.785	−6.956 × 10^−2^	0.911
13.	*SMC3*	Structural maintenance of chromosomes 3	2.226 × 10^−2^	1.204	7.135 × 10^−2^	0.982
14.	*RECQL*	RecQ like helicase	2.592 × 10^−2^	1.246	5.847 × 10^−2^	0.857
15.	*TOP1*	DNA topoisomerase I	2.677 × 10^−2^	1.219	6.918 × 10^−2^	0.893
16.	*ZMPSTE24*	Zinc metallopeptidase STE24	2.677 × 10^−2^	1.314	5.716 × 10^−2^	0.929
17.	*POLE4*	DNA polymerase epsilon 4, accessory subunit	3.143 × 10^−2^	1.297	6.662 × 10^−2^	0.929
18.	*TERF2IP*	TERF2 interacting protein	4.007 × 10^−2^	1.231	8.871 × 10^−2^	0.946
19.	*SAMHD1*	SAM and HD domain containing deoxynucleoside triphosphate triphosphohydrolase 1	4.624 × 10^−2^	1.270	6.332 × 10^−2^	0.893
**AAA vs. CVD**						
1.	*LIG1*	DNA ligase 1	1.405 × 10^−2^	0.734	−2.215 × 10^−1^	0.878
2.	*POLE4*	DNA polymerase epsilon 4. accessory subunit	1.405 × 10^−2^	1.381	2.107 × 10^−1^	0.918
**LEAD, AAA and CVD vs. Control group**						
3.	*WHSC1*	Nuclear receptor binding SET domain protein 2	2.248 × 10^−3^	1.245	1.269 × 10^−1^	0.981
4.	*UPF1*	UPF1 RNA helicase and ATPase	3.682 × 10^−3^	1.204	9.414 × 10^−2^	0.955
5.	*POLE*	DNA polymerase epsilon, catalytic subunit	3.016 × 10^−2^	1.242	9.073 × 10^−2^	0.909
**LEAD and AAA vs. CVD**						
1.	*ATM*	ATM serine/threonine kinase	2.116 × 10^−2^	0.791	−1.108 × 10^−1^	0.867
2.	*INIP*	INTS3 and NABP interacting protein	2.116 × 10^−2^	1.282	1.131 × 10^−1^	0.914
3.	*LIG1*	DNA ligase 1	2.116 × 10^−2^	0.764	−1.165 × 10^−1^	0.895
4.	*POLE4*	DNA polymerase epsilon, 4 accessory subunit	2.116 × 10^−2^	1.334	1.197 × 10^−1^	0.924
5.	*TREX2*	Three prime repair exonuclease 2	2.116 × 10^−2^	0.569	−1.122 × 10^−1^	0.914
6.	*XRCC6*	X–ray repair cross complementing 6	2.116 × 10^−2^	1.222	8.096 × 10^−2^	0.914

AAA—abdominal aortic aneurysm. CVD—chronic venous disease. LEAD—lower extremity arterial disease, ROC—receiver operating characteristics, UVE-PLS—uninformative variable elimination by partial least squares. ^1^ Gene name and symbol according to HUGO Gene Nomenclature Committee.

**Table 3:** Correlation analysis between characteristics of studied groups: age and body mass index (BMI) and expression of genes selected from comparisons performed. The table presents genes correlated with statistical significance *p* < 0.05 (adjusted by Benjamini–Hochberg FDR).

**Comparison**	**Gene Symbol**	**R**	***p***
Age			
AAA vs. control	*APBB1*	−0.590	7.116 × 10^−3^
	*NPAS2*	−0.456	4.393 × 10^−2^
	*PRRX1*	−0.510	2.300 × 10^−2^
	*SIRT7*	0.499	2.630 × 10^−2^
	*TOP3A*	0.579	8.596 × 10^−3^
	*USP51*	−0.672	1.536 × 10^−3^
	*VCP*	0.619	4.216 × 10^−3^
	*JMY*	−0.490	2.959 × 10^−2^
LEAD vs. control, AAA vs. control, LEAD vs. CVD, ART vs. CVD	*ATM*	−0.653	2.262 × 10^−3^
AAA vs. CVD, ART vs. CVD	*LIG1*	−0.461	4.177 × 10^−2^
LEAD vs. CVD, AAA vs. CVD, ART vs. CVD	*POLE4*	0.580	8.546 × 10^−3^
AAA vs. control, CVD vs. control, Disease vs. control	*UPF1*	0.558	1.200 × 10^−2^
	*WHSC1*	0.479	3.370 × 10^−2^
BMI			
AAA vs. control	*PRRX1*	−0.519	2.061 × 10^−2^
	*USP51*	−0.533	1.726 × 10^−2^
	*VCP*	0.467	3.826 × 10^−2^
LEAD vs. CVD	*RECQL*	0.450	4.744 × 10^−2^
LEAD vs. CVD	*POLM*	−0.469	3.729 × 10^−2^

AAA—abdominal aortic aneurysm, CVD—chronic venous disease, LEAD—lower extremity arterial disease, BMI—body mass index, R—correlation coefficient.

## Data Availability

https://doi.org/10.6084/m9.figshare.19518010.v1.

## Supplementary Materials

The following supporting information can be downloaded at: https://www.mdpi.com/article/10.3390/ijms24010551/s1.

## Abbreviations

8–oxoG	8–oxo–7:8–dihydroguanine
AAA	Abdominal aortic aneurysm
BAF200	BAF Nuclear Assembly Factor 1
BER	Base Excision Repair
BMI	Body mass index
CVD	Chronic Venous Disease
DSBs	Double-strand breaks
FDR	False Discovery Rate
HUGO	Human Genome Organization
LEAD	Lower Extremities Arterial Disease
MA plot	Bland–Altman plot for visual representation of genomic data
NER	Nucleotide excision repair
NGS	Next-Generation Sequencing
NHEJ	Non–Homologous End Joining
p53	Tumor Protein P53
PAD	Peripheral Arterial Disease
PBMCs	Peripheral blood mononuclear cells
ROC	Receiver Operating Characteristics
ROS	Reactive oxygen species
Swi/Snf complex	SWItch/Sucrose Non–Fermentable complex
UV	Ultraviolet
UVE–PLS	Uninformative Variable Elimination by Partial Least Squares
VEGFA	Vascular Endothelial Growth Factor A
VSMCs	Vascular Smooth Muscle Cells

## Appendix A

**Table A1:** General results of differential gene expression analysis performed using DESeq2 and UVE-PLS methods obtained for comparisons.

Comparison	DESeq2	UVE–PLS		Number of Genes Common for Sets of Genes Selected from DESeq2 (*p* < 0.05) and from UVE–PLS as Informative
	Number of Differentially Expressed Genes with *p* < 0.05 and Fold Change > 1.2 (for Upregulated Genes) or Fold Change < 0.8 (for Downregulated Genes)	Number of PLS Components/Iterations	Number of Informative Genes (Default Cutoff Threshold of Reliability Score)	
LEAD vs. control	1	4/1000	7	1
AAA vs. control	45	2/1000	40	27
CVD vs. control	29	3/1000	41	23
LEAD vs. AAA	12	3/1000	18	11
LEAD vs. CVD	23	2/1000	35	19
AAA vs. CVD	2	2/1000	14	2
LEAD, AAA and CVD vs. control	10	3/1000	11	3
LEAD and AAA vs. CVD	6	4/1000	20	6

*p*—statistical significance after correction by Benjamini–Hochberg false discovery rate, AAA—abdominal aortic aneurysm, CVD—chronic venous disease, LEAD—lower extremity artery disease, UVE–PLS—uninformative variable elimination by partial least squares.
