# Supplementary material for: Next-Generation Sequencing in the Assessment of the Transcriptomic Landscape of DNA Damage Repair Genes in Abdominal Aortic Aneurysm, Chronic Venous Disease and Lower Extremity Artery Disease
Source: Int J Mol Sci. 2022 Dec 29;24(1):551. doi: 10.3390/ijms24010551 (PMC9820637; doi:10.3390/ijms24010551)
Supplement: Supplementary file 1 [file ijms-24-00551-s001.zip › ijms-1778386-supplementary v3.pdf]

SUPPLEMENTARY MATERIAL

Supplementary figures

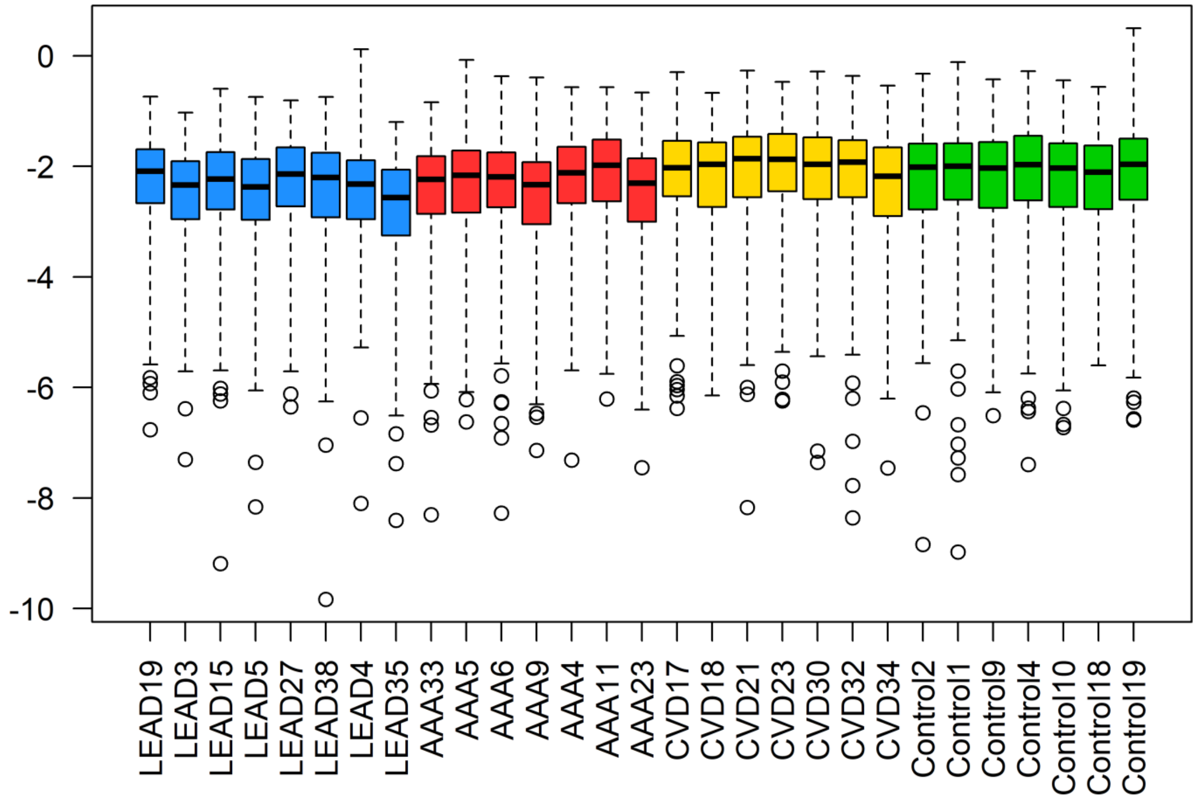

Figure S1. Boxplot presenting Cook's distances of genes across samples. Whiskers define range between minimum and maximum value of Cook's distance, boxes range between 25% and 75% quartile, horizontal lines inside boxes mark median value. LEAD - lower extremities arterial disease, AAA - abdominal aortic aneurysm, CVD - chronic venous disease.

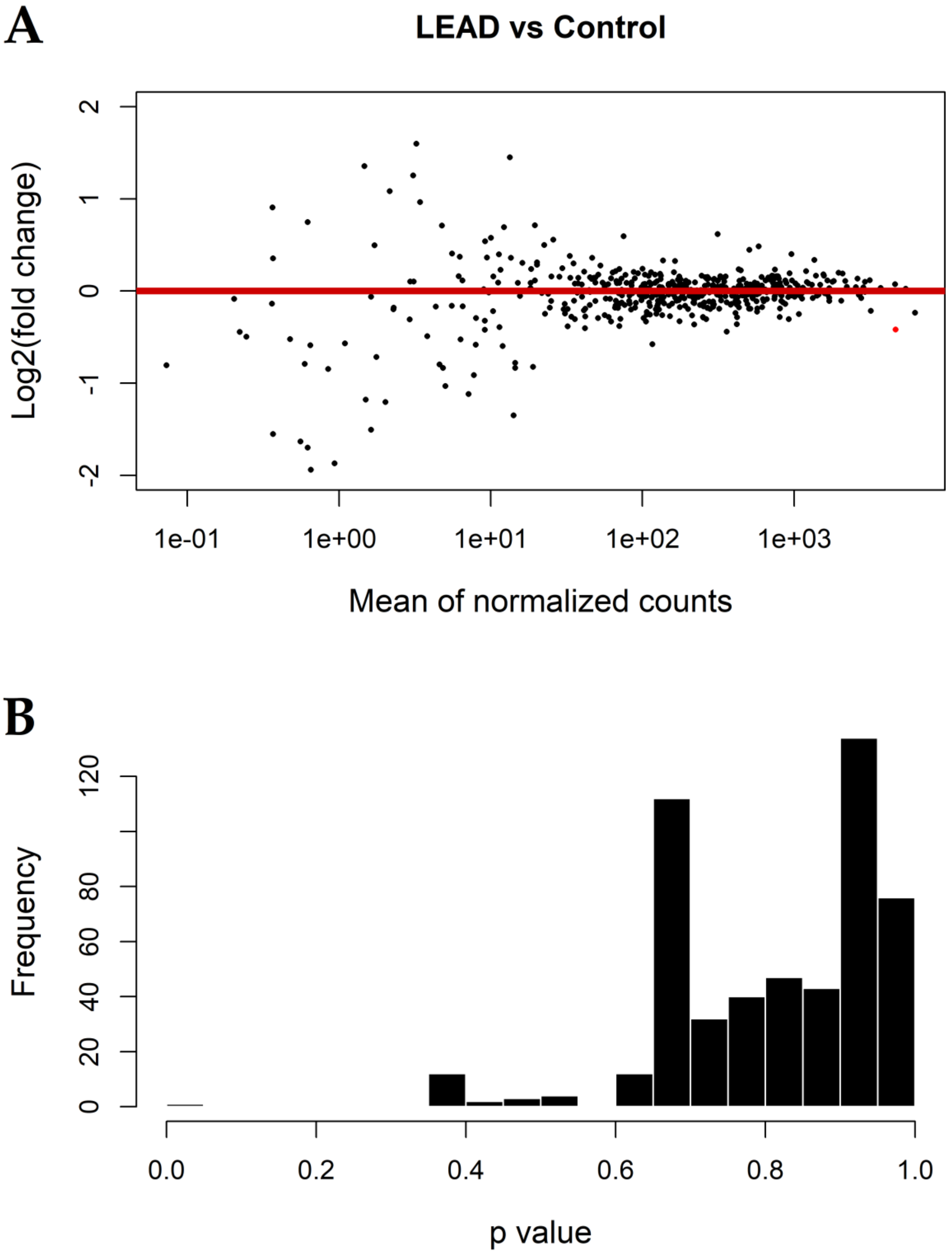

Figure S2. Quality control of results obtained from differential gene expression analysis performed by DESeq2 package between group of 8 LEAD subjects and the group of 7 healthy controls. (**A**) MA plot showing relation between log2 of fold changes of differentially expressed genes and averages of normalized counts. Genes with *p* value < 0.05 are marked as red points. (**B**) Histogram presenting distribution of *p* values.

A

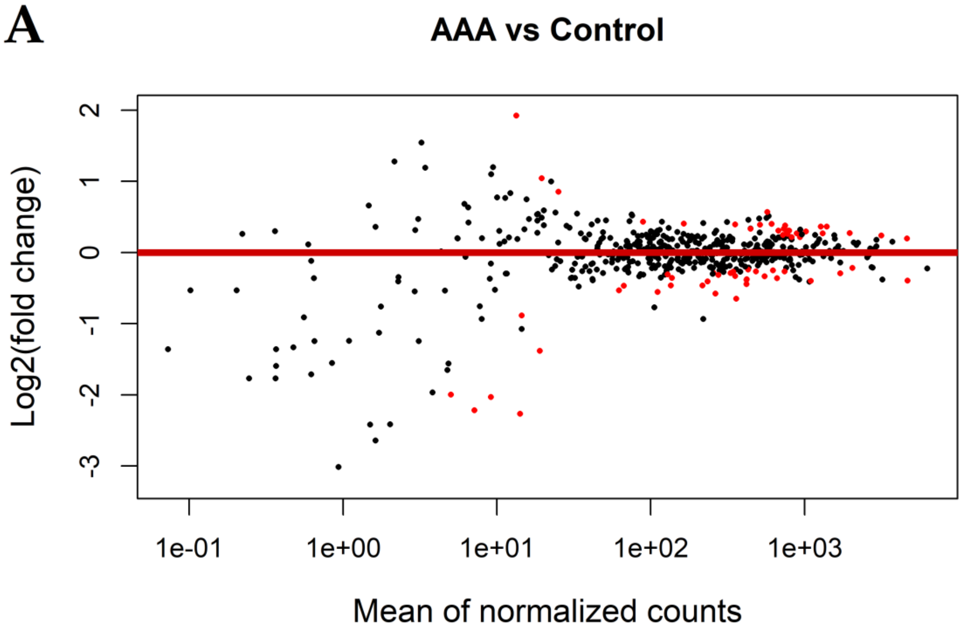

B

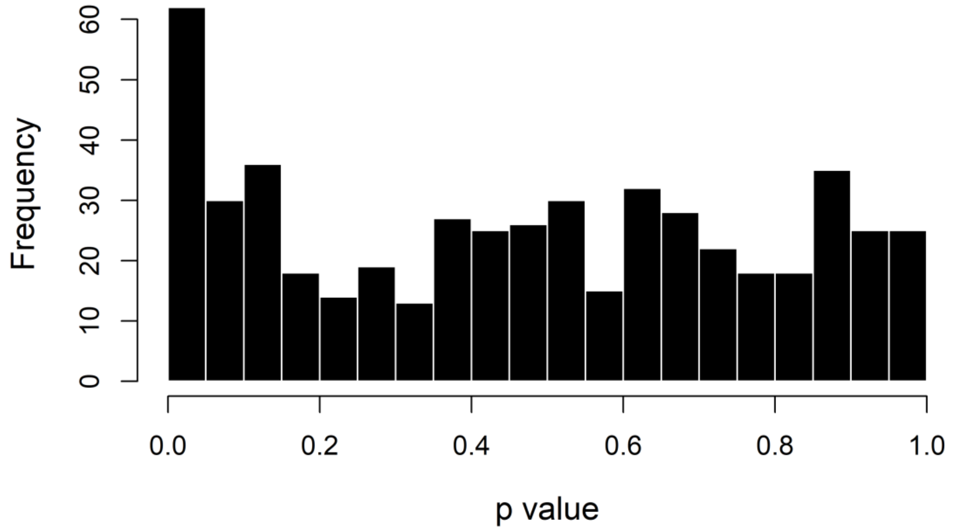

Figure S3. Quality control of results obtained from differential gene expression analysis performed by DESeq2 package between group of 7 AAA subjects and the group of 7 healthy controls. (**A**) MA plot showing relation between log2 of fold changes of differentially expressed genes and averages of normalized counts. Genes with p value < 0.05 are marked as red points. (**B**) Histogram presenting distribution of p values.

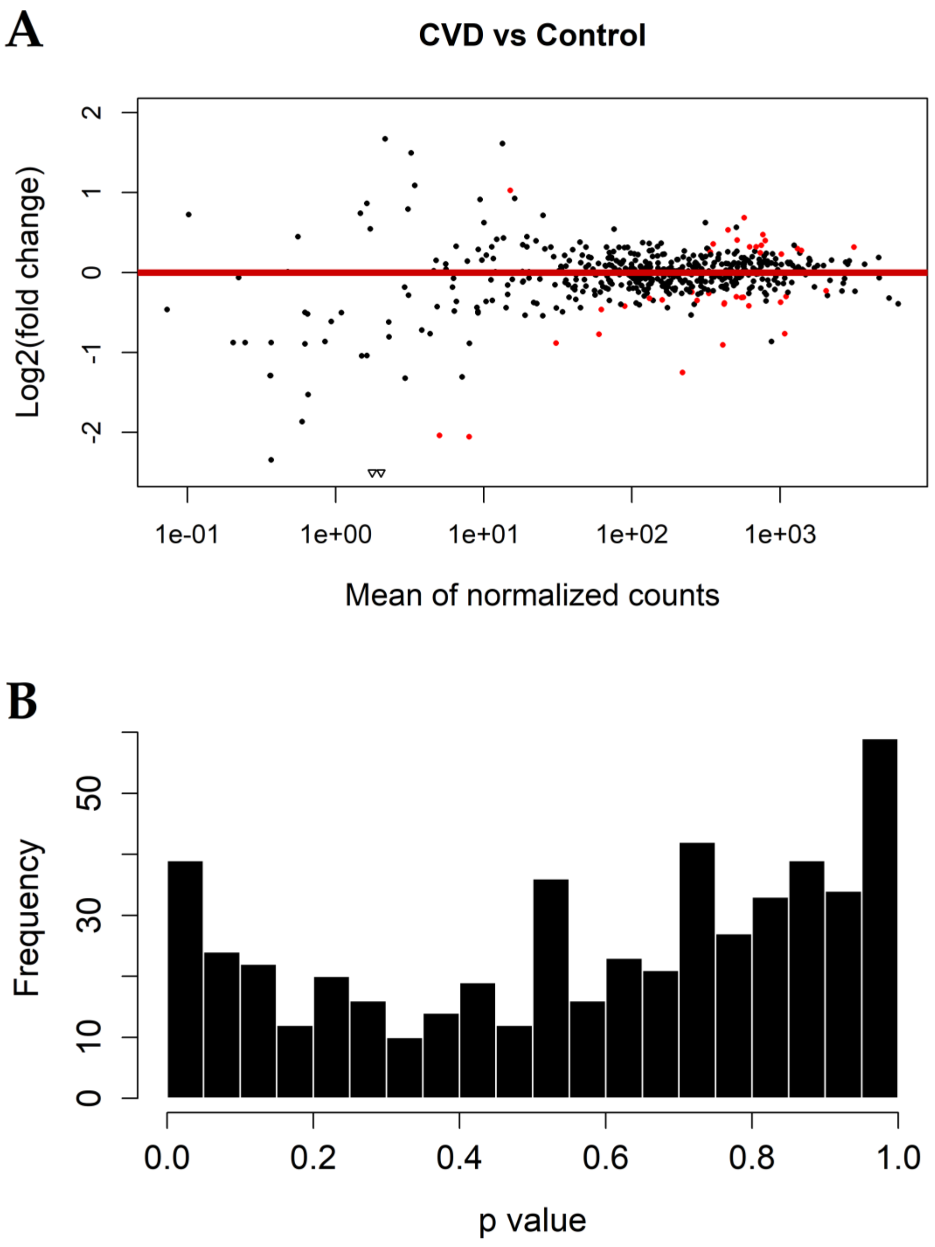

Figure S4. Quality control of results obtained from differential gene expression analysis performed by DESeq2 package between group of 7 CVD subjects and the group of 7 healthy controls. (**A**) MA plot showing relation between log2 of fold changes of differentially expressed genes and averages of normalized counts. Genes with *p* value < 0.05 are marked as red points. (**B**) Histogram presenting distribution of *p* values.

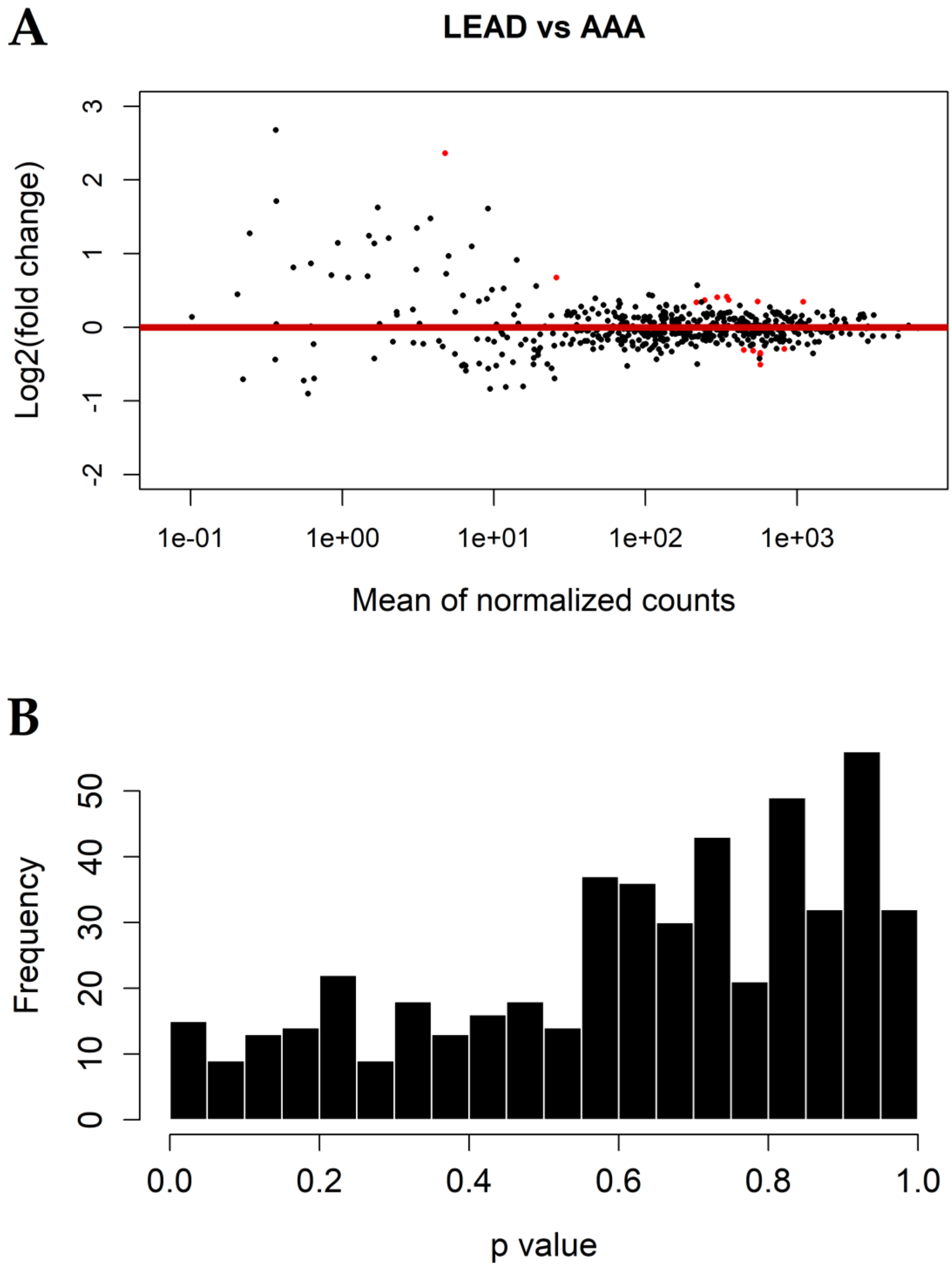

Figure S5. Quality control of results obtained from differential gene expression analysis performed by DESeq2 package between group of 8 LEAD subjects and the group of 7 AAA subjects. (**A**) MA plot showing relation between log2 of fold changes of differentially expressed genes and averages of normalized counts. Genes with *p* value < 0.05 are marked as red points. (**B**) Histogram presenting distribution of *p* values.

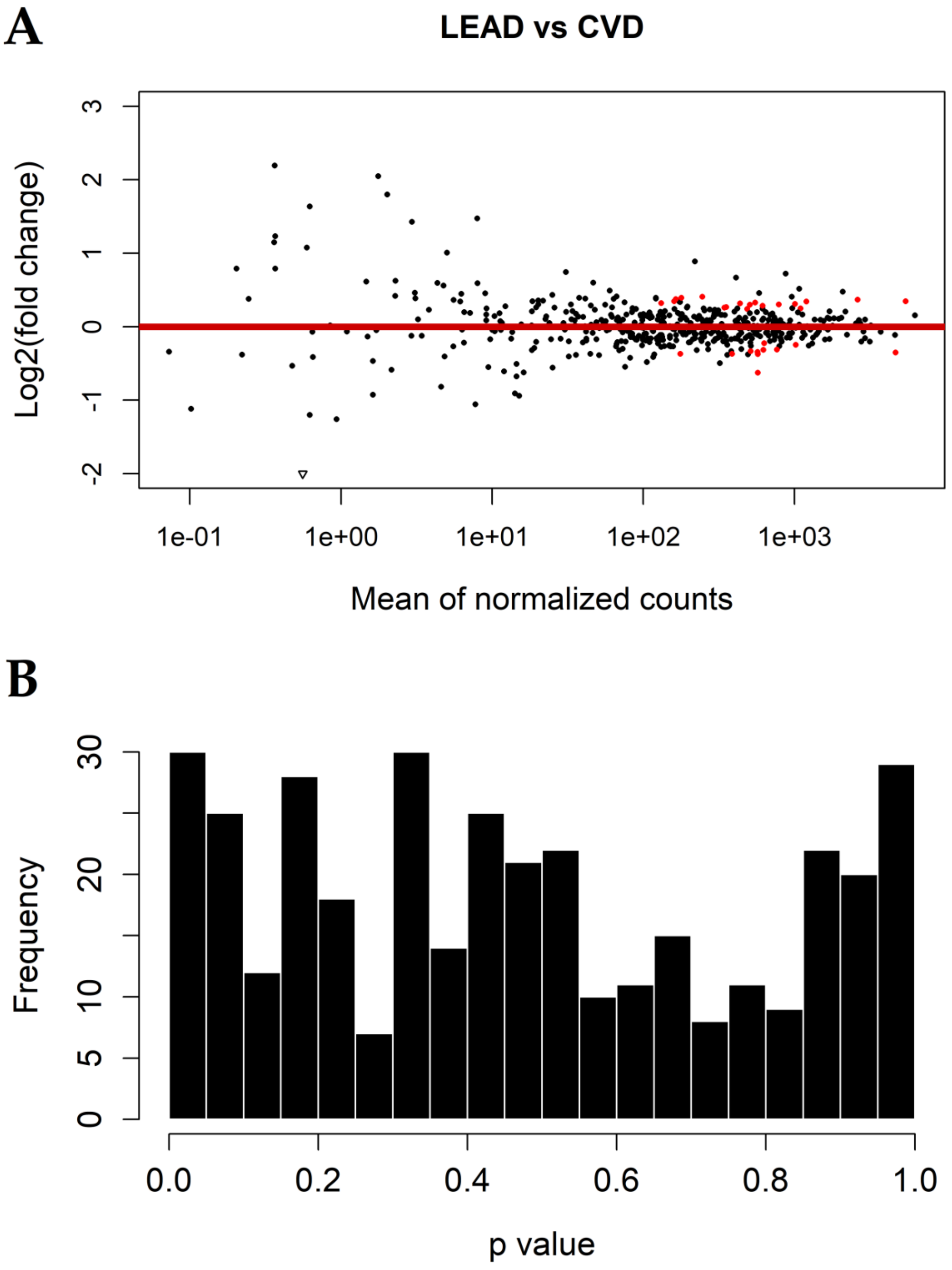

Figure S6. Quality control of results obtained from differential gene expression analysis performed by DESeq2 package between group of 8 LEAD subjects and the group of 7 CVD subjects. (**A**) MA plot showing relation between log2 of fold changes of differentially expressed genes and averages of normalized counts. Genes with p value < 0.05 are marked as red points. (**B**) Histogram presenting distribution of p values.

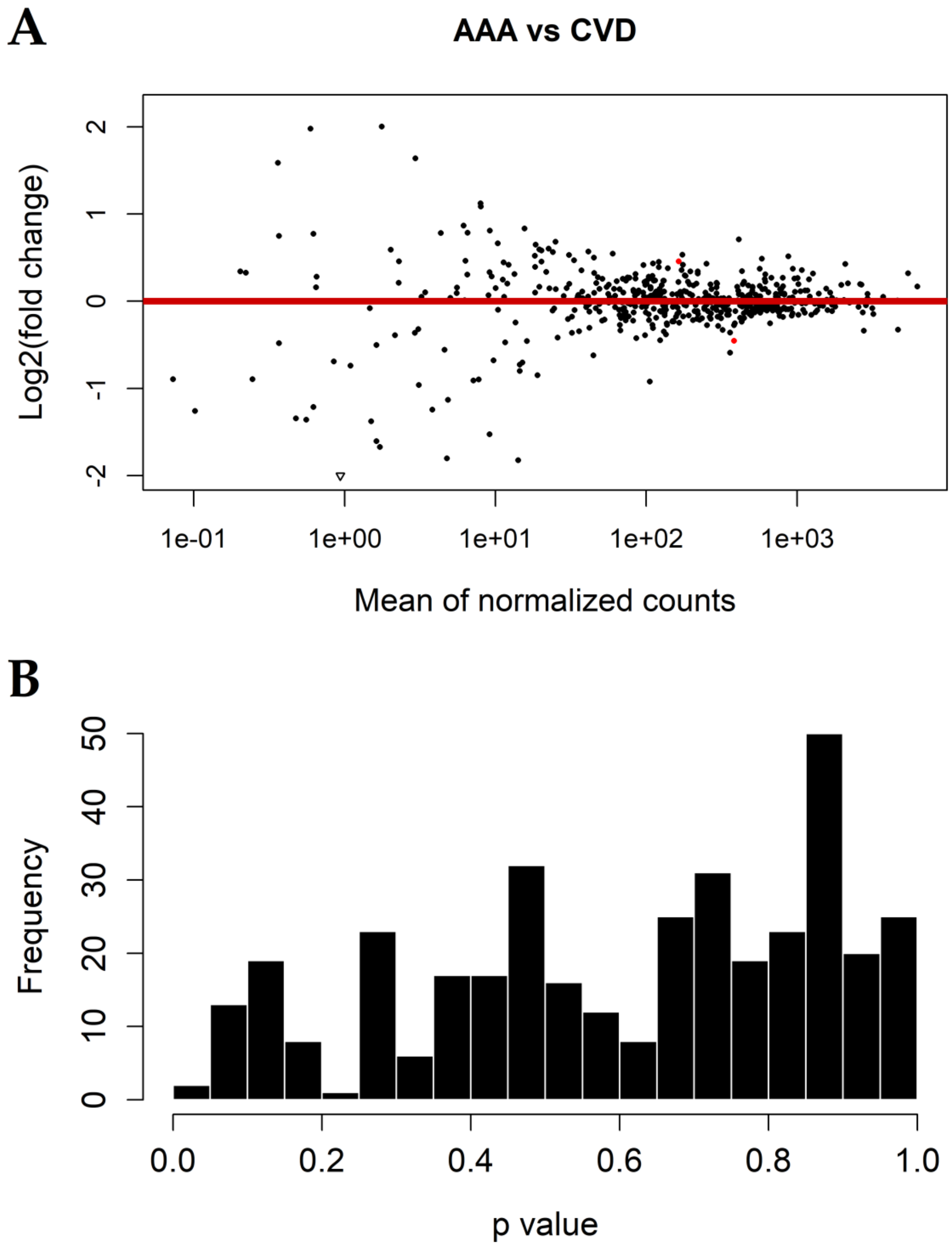

Figure S7. Quality control of results obtained from differential gene expression analysis performed by DESeq2 package between group of 7 AAA subjects and the group of 7 CVD subjects. (**A**) MA plot showing relation between log2 of fold changes of differentially expressed genes and averages of normalized counts. Genes with *p* value < 0.05 are marked as red points. (**B**) Histogram presenting distribution of *p* values.

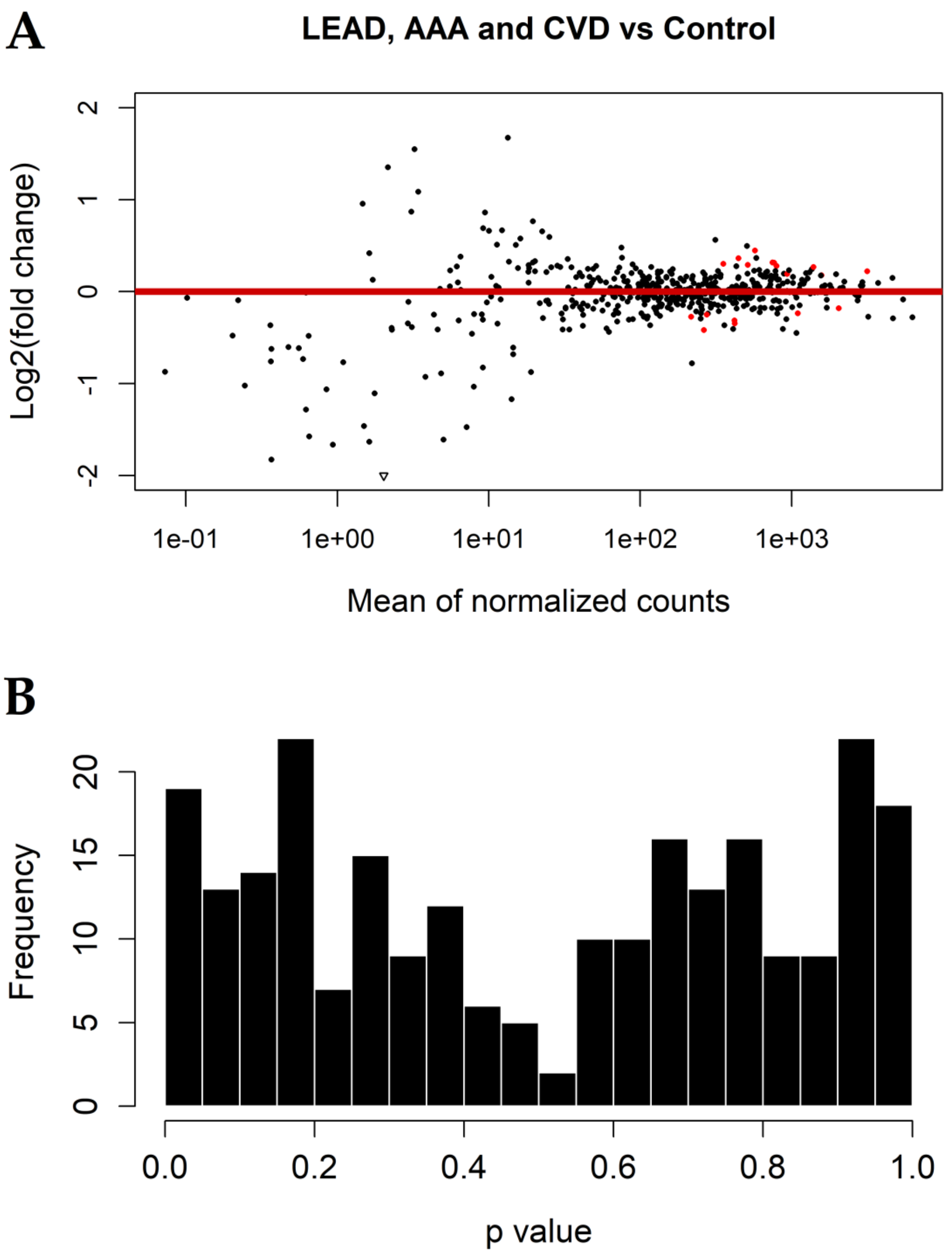

Figure S8. Quality control of results obtained from differential gene expression analysis performed by DESeq2 package between pooled groups of patients (8 LEAD, 7 AAA and 7 CVD subjects) and 7 healthy controls. (**A**) MA plot showing relation between log2 of fold changes of differentially expressed genes and averages of normalized counts. Genes with p value < 0.05 are marked as red points. (**B**) Histogram presenting distribution of p values.

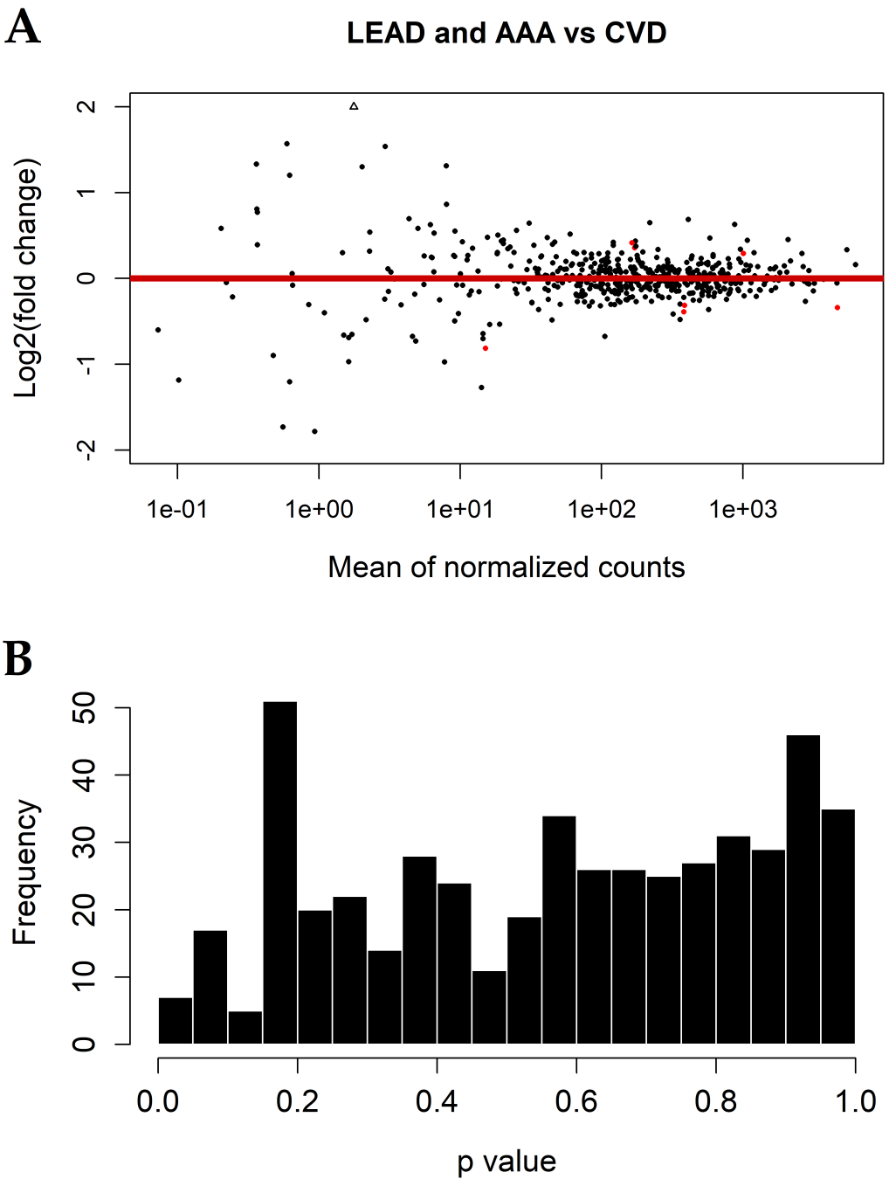

Figure S9. Quality control of results obtained from differential gene expression analysis performed by DESeq2 package between pooled groups of patients with arterial diseases (8 LEAD and 7 AAA subjects) and 7 CVD patients. (**A**) MA plot showing relation between log2 of fold changes of differentially expressed genes and averages of normalized counts. Genes with *p* value < 0.05 are marked as red points. (**B**) Histogram presenting distribution of *p* values.

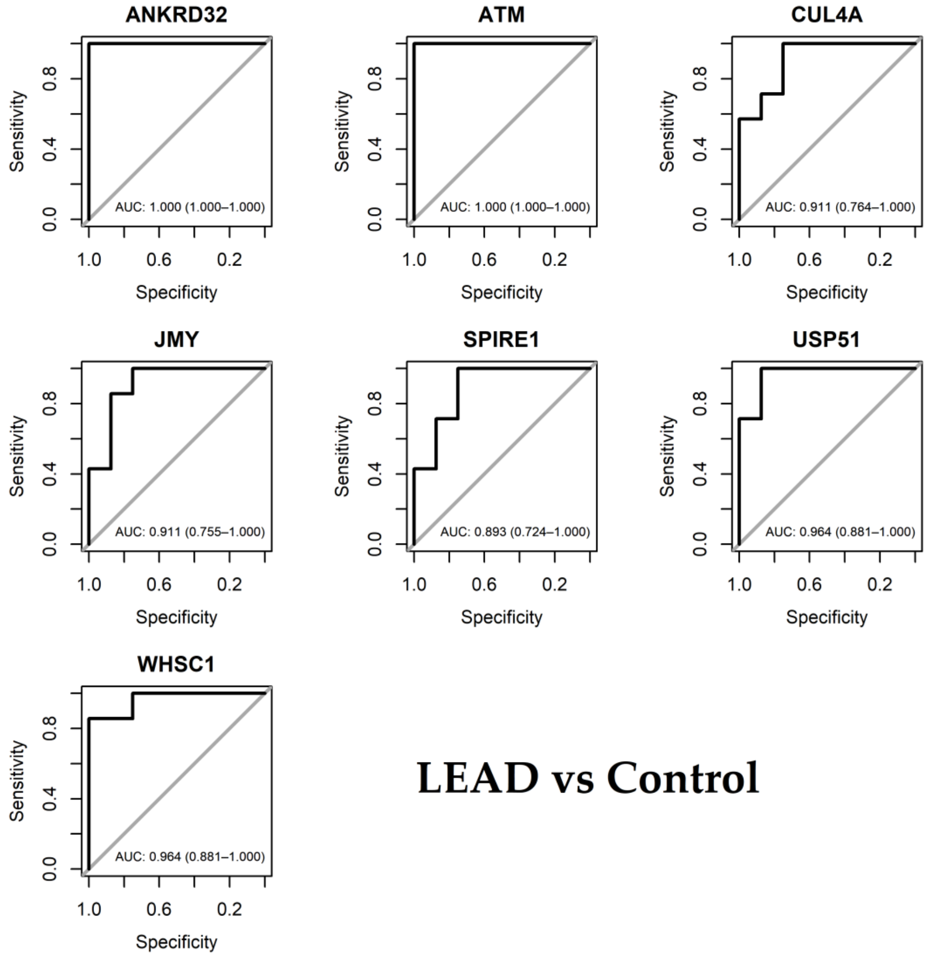

Figure S10. Results of Receiver Operating Characteristics (ROC) analysis performed for genes selected from pairwise comparison between LEAD and Control groups.

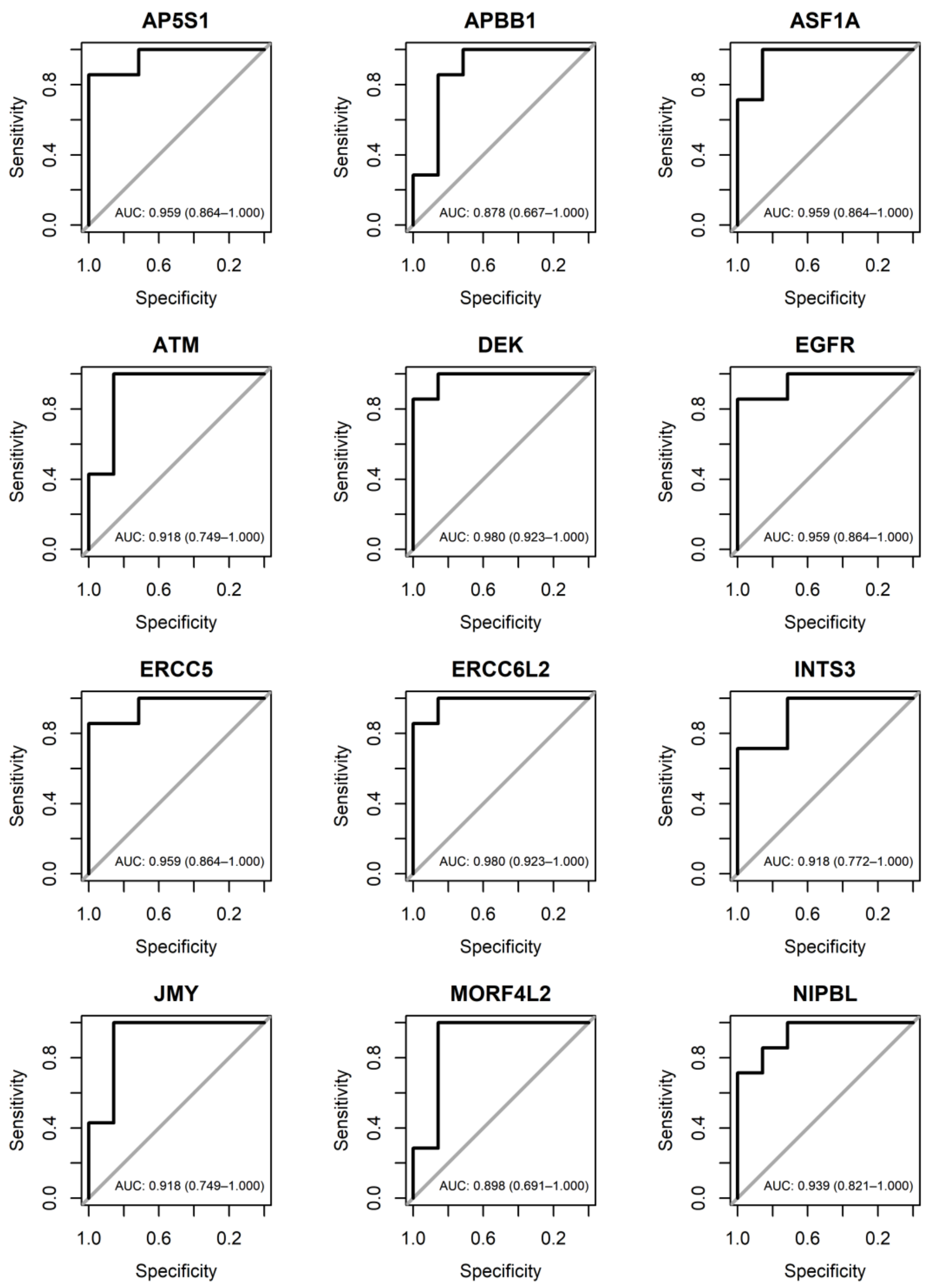

Figure S11. Results of Receiver Operating Characteristics (ROC) analysis performed for genes selected from pairwise comparison between AAA and Control groups.

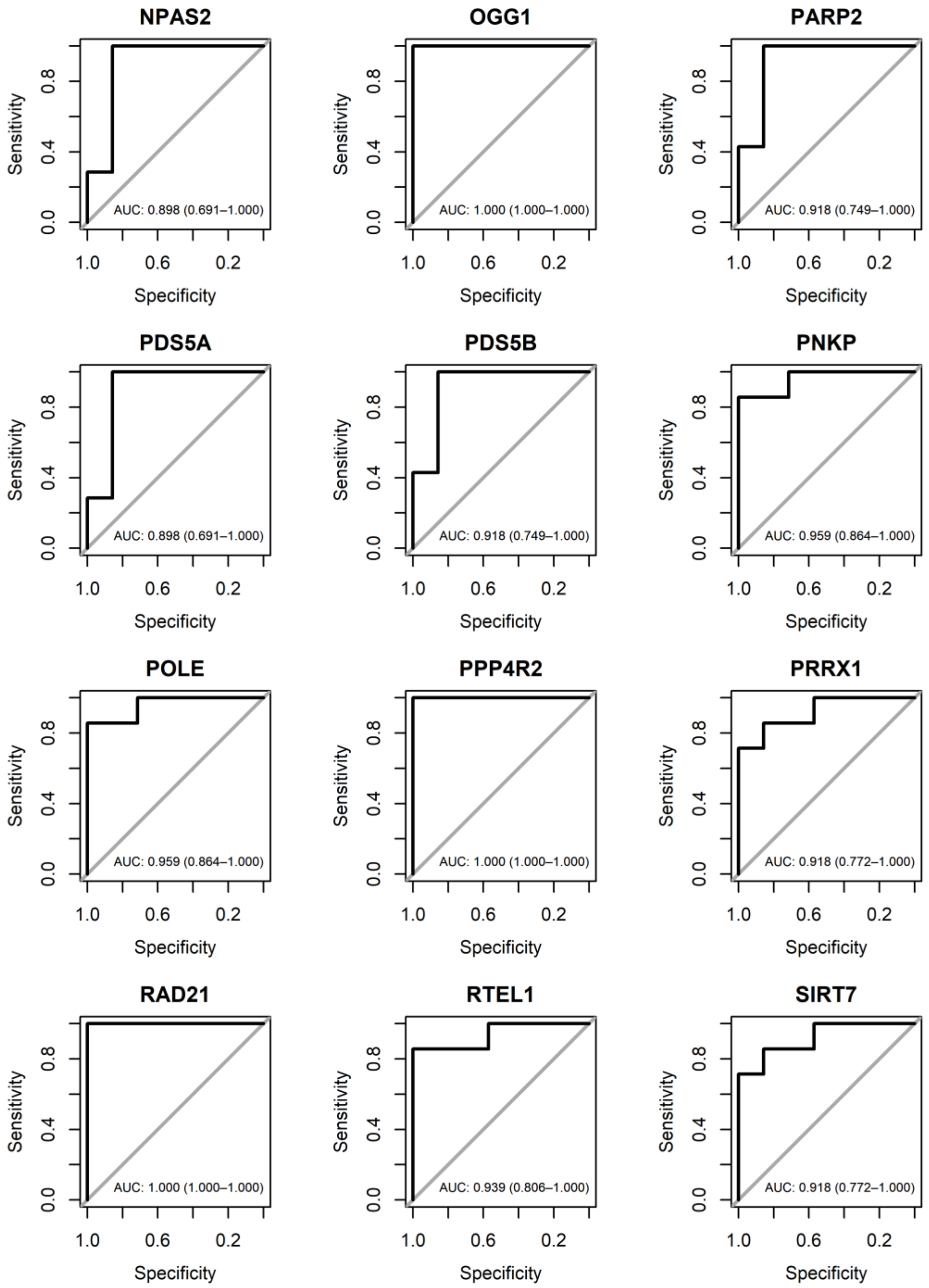

Figure S11. Continued

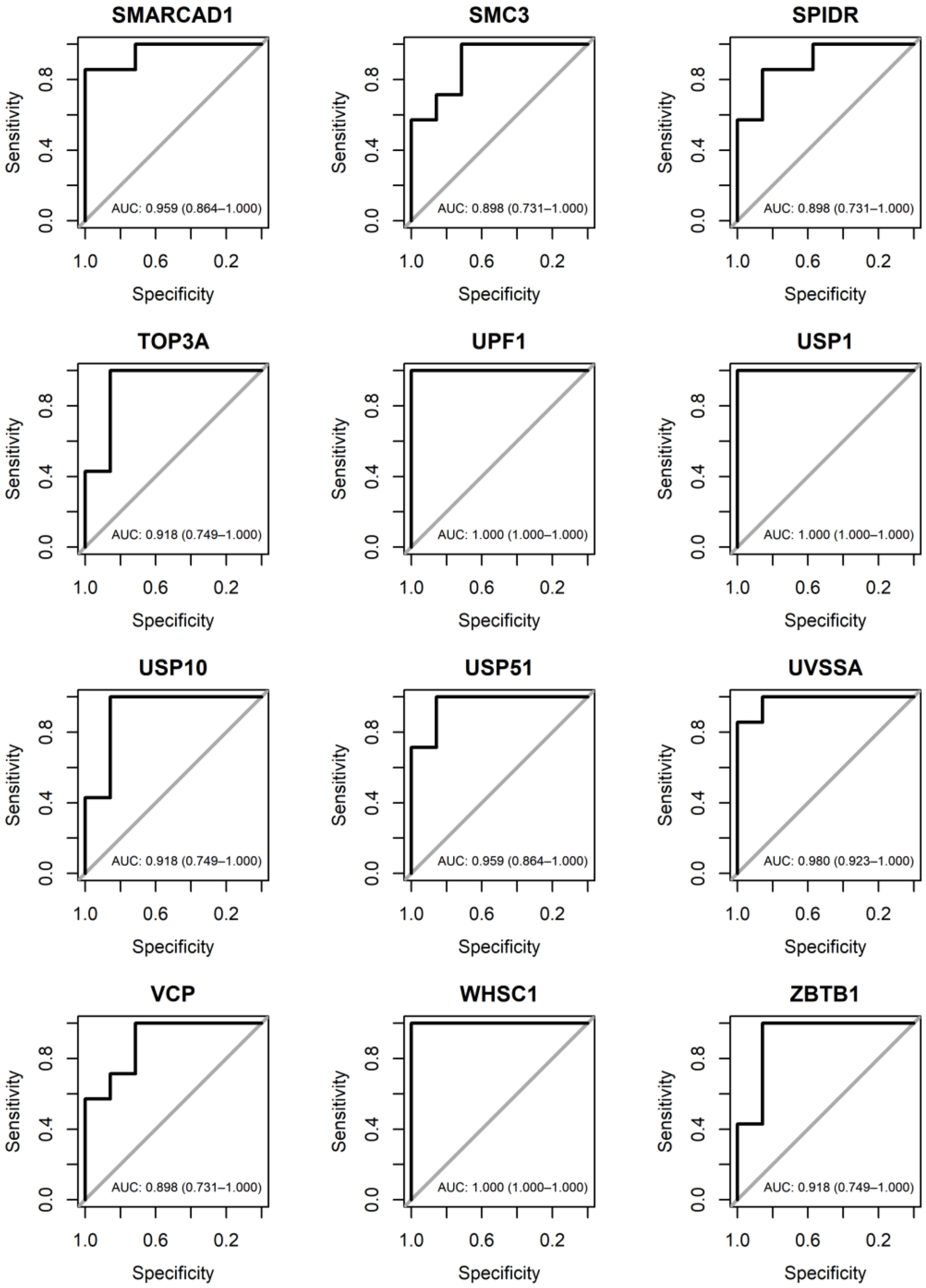

Figure S11. Continued

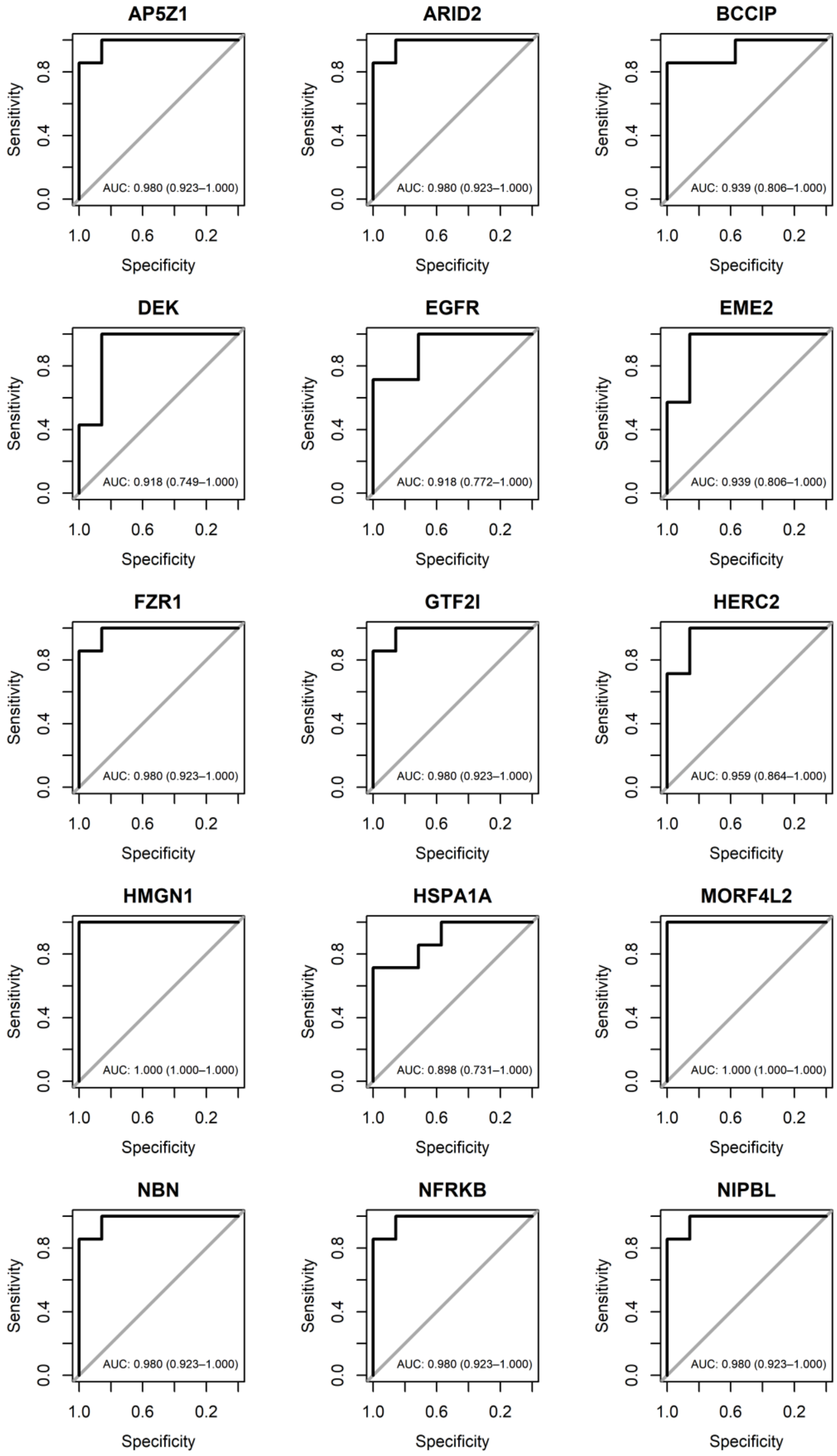

Figure S12. Results of Receiver Operating Characteristics (ROC) analysis performed for genes selected from pairwise comparison between CVD and Control groups.

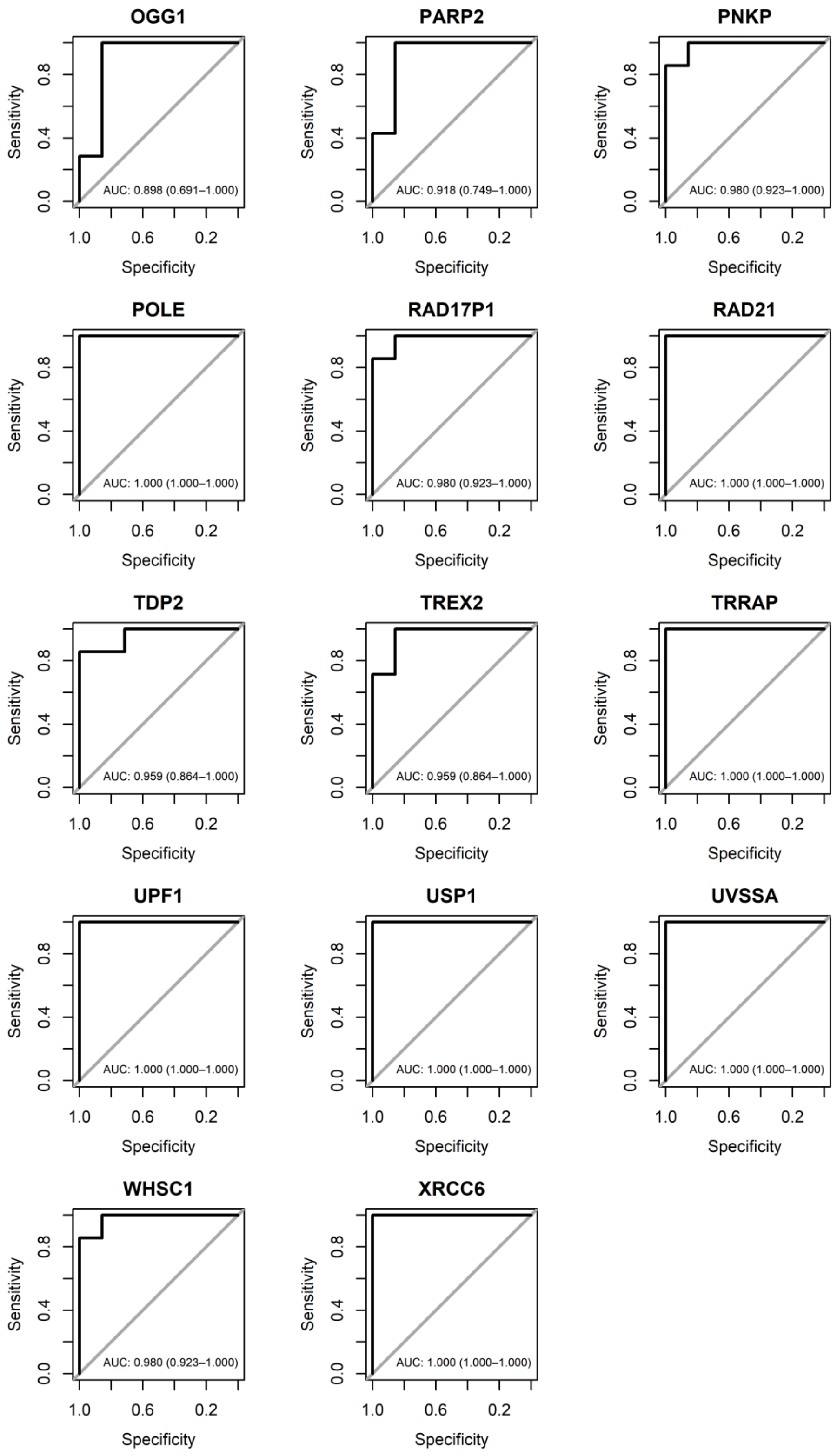

Figure S12. Continued

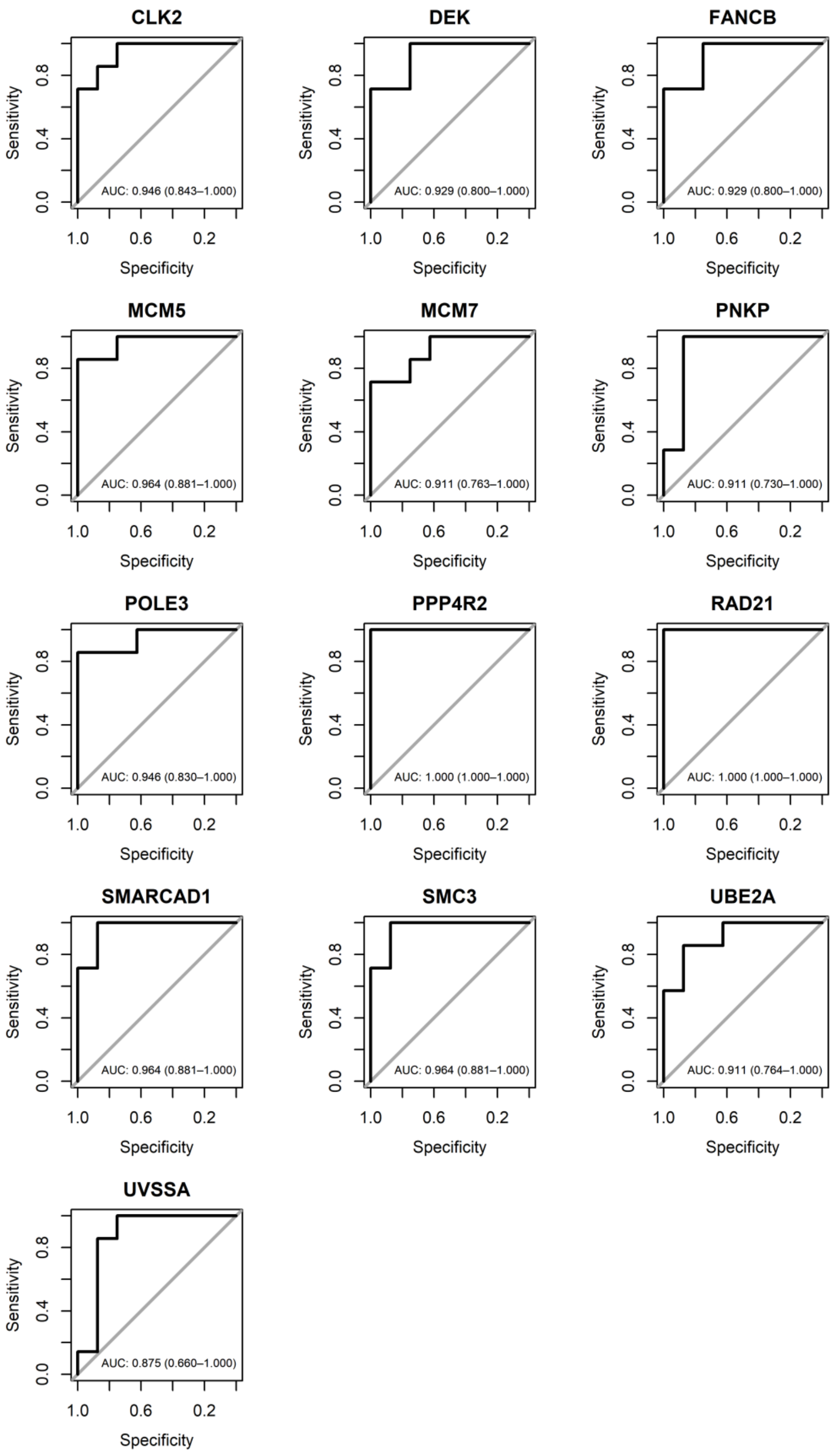

Figure S13. Results of Receiver Operating Characteristics (ROC) analysis performed for genes selected from pairwise comparison between LEAD and AAA groups.

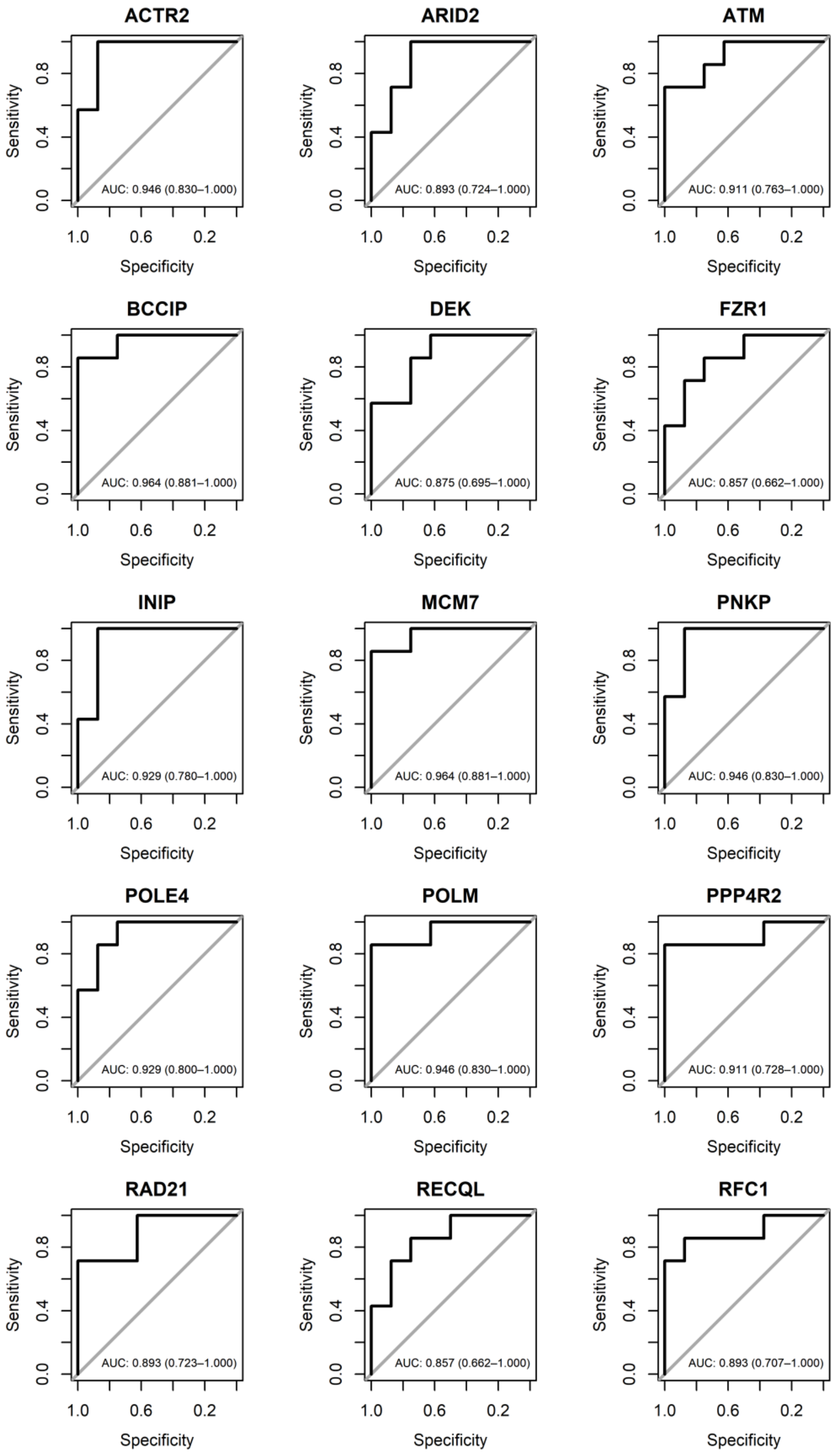

Figure S14. Results of Receiver Operating Characteristics (ROC) analysis performed for genes selected from pairwise comparison between LEAD and CVD groups.

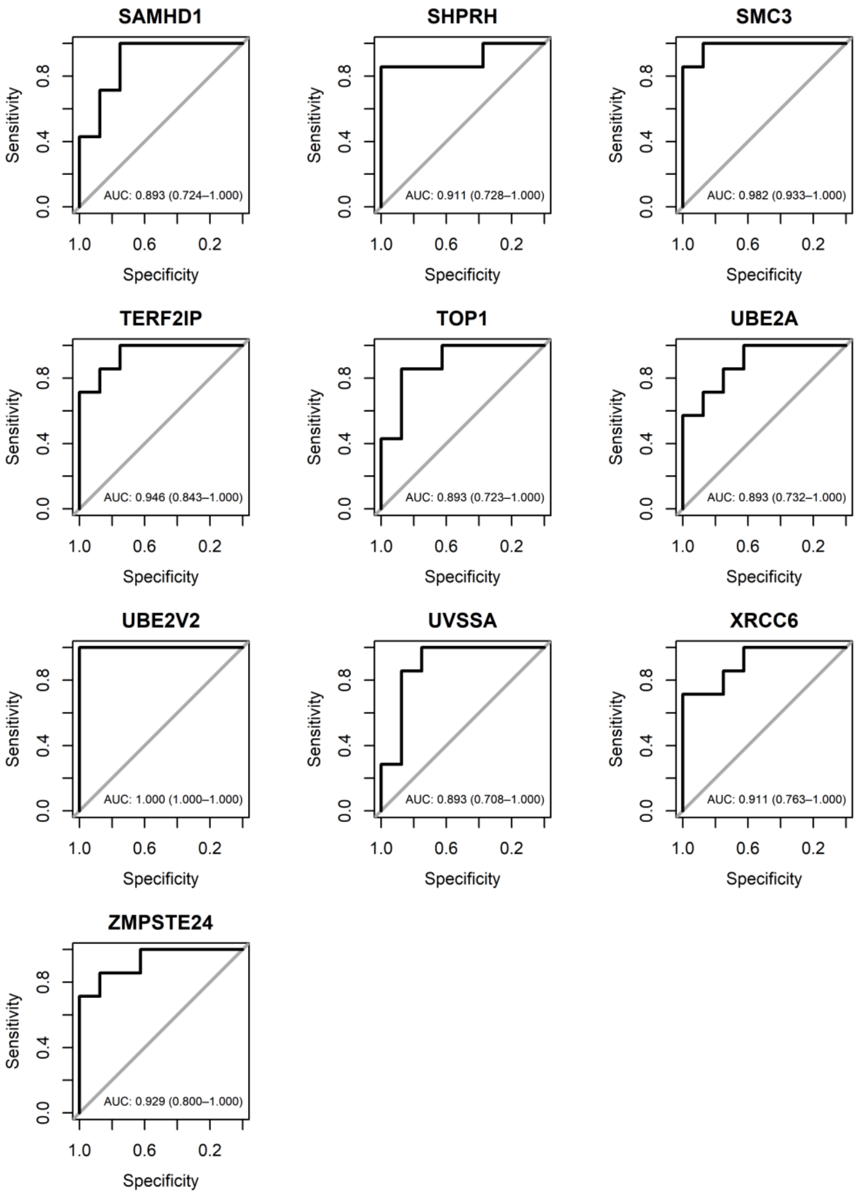

Figure S14. Continued

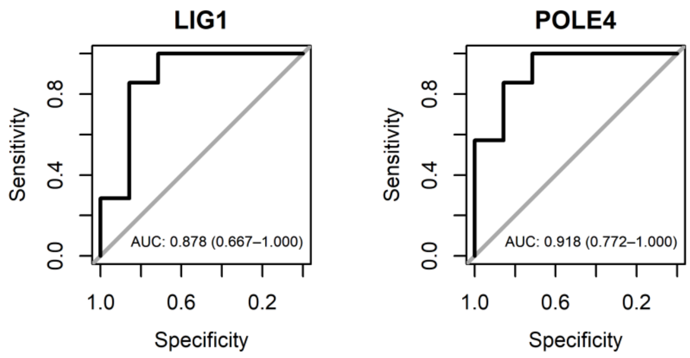

Figure S15. Results of Receiver Operating Characteristics (ROC) analysis performed for genes selected from pairwise comparison between AAA and CVD groups.

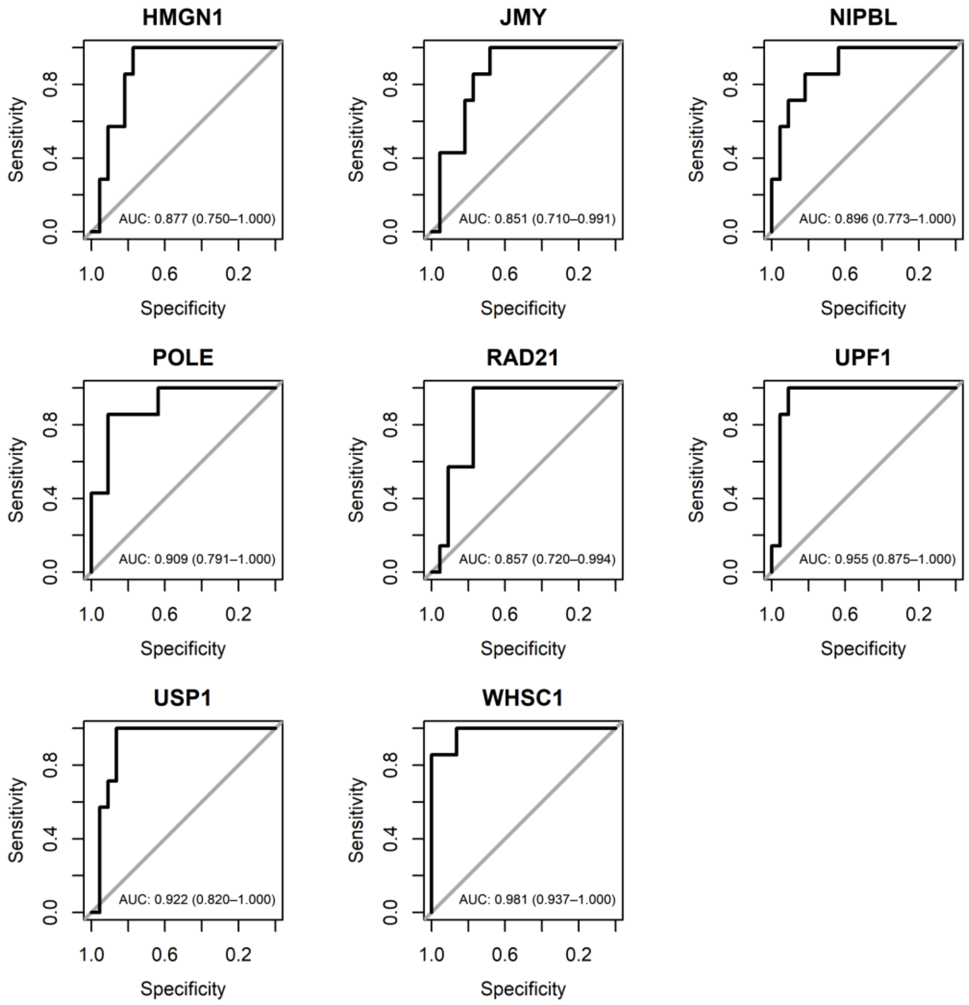

Figure S16. Results of Receiver Operating Characteristics (ROC) analysis performed for genes selected from comparison between pooled groups of patients (LEAD, AAA and CVD subjects) and control groups.

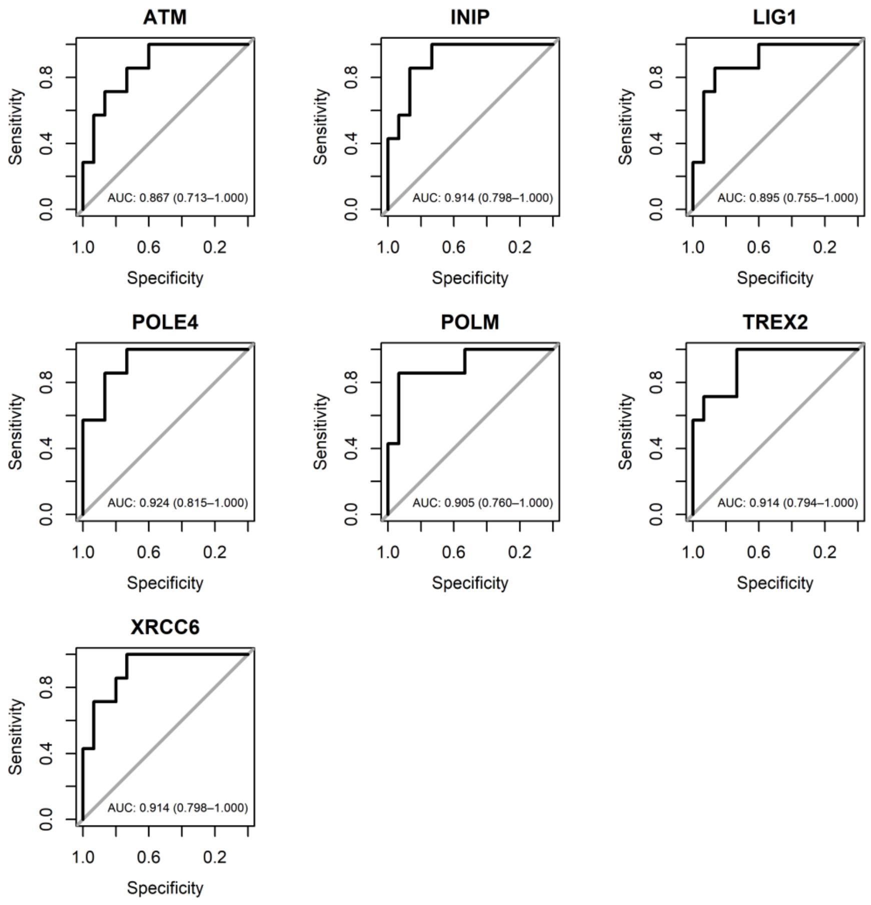

Figure S17. Results of Receiver Operating Characteristics (ROC) analysis performed for genes selected from comparison between pooled groups of patients with arterial diseases (LEAD and AAA) and CVD patients groups.

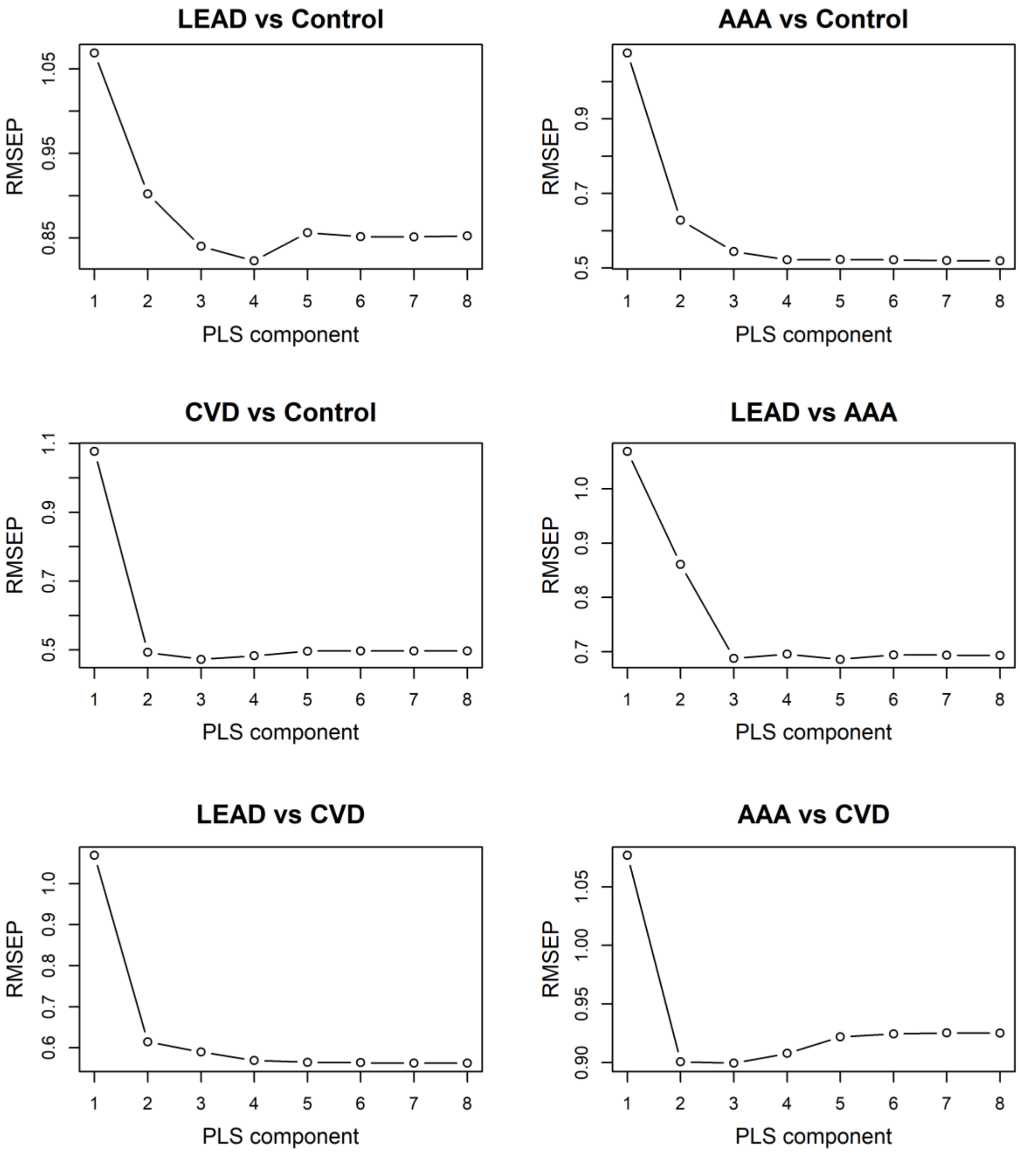

Figure S18. Plots presenting the arrangement of prediction error and Partial Least Squares (PLS) components generated in UVE-PLS differential expression analysis of gene expression data for pairwise comparisons. **LEAD vs Control:** 8 LEAD subjects versus 7 healthy controls, **AAA vs Control:** 7 AAA subjects versus 7 healthy controls, **CVD vs Control:** 7 CVD subjects versus 7 healthy controls, **LEAD vs AAA:** 8 LEAD subjects versus 7 AAA subjects, **LEAD vs CVD:** 8 LEAD subjects versus 7 CVD subjects **AAA vs CVD:** 7 AAA subjects versus 7 CVD subjects. RMSEP - Root Mean Squared Error of Prediction

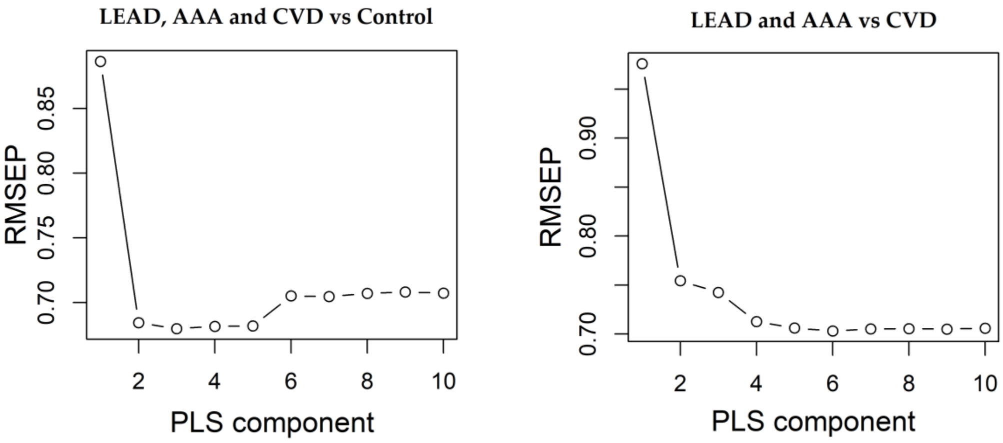

Figure S19. Plots presenting the arrangement of prediction error and Partial Least Squares (PLS) components generated in UVE-PLS differential expression analysis of gene expression data for comparisons with pooled groups. **LEAD, AAA and CVD vs Control**: pooled group of 8 LEAD, 7 AAA and 7 CVD subjects versus 7 healthy controls, **LEAD and AAA vs CVD:** pooled group of 8 LEAD and 7 AAA subjects versus 7 CVD patients. RMSEP - Root Mean Squared Error of Prediction

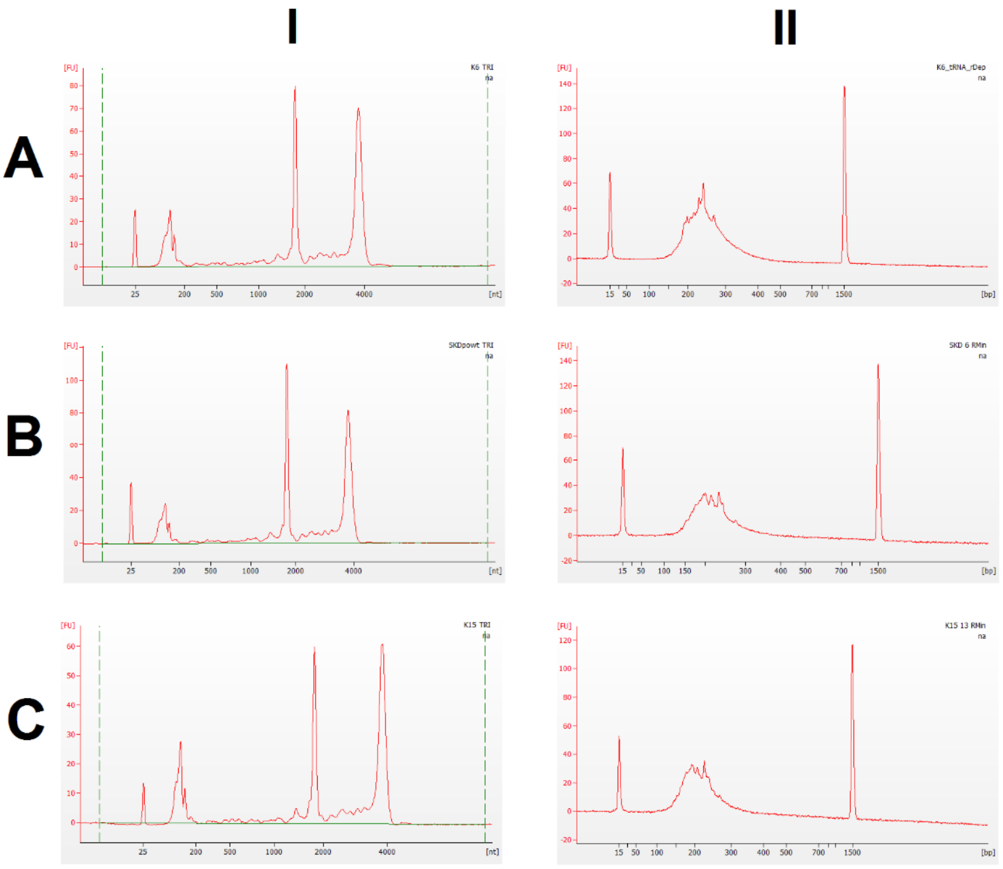

Figure S20: Representative electrophoregrams of total RNA (IA, IB and IC) and corresponding libraries (IIA, IIB and IIC) of three randomly selected samples. Evaluations were performed using the Agilent 2100 Bioanalyzer instrument with the Agilent RNA 6000 Pico Kit (for total RNA samples) and Agilent DNA 1000 Kit (for libraries) [Bogucka–Kocka, A.; Zalewski, D.P.; Ruszel, K.P.; Stępniewski, A.; Gałkowski, D.; Bogucki, J.; Komsta, Ł.; Kołodziej, P.; Zubilewicz, T.; Feldo, M.; Kocki, J. Dysregulation of MicroRNA Regulatory Network in Lower Extremities Arterial Disease. *Front. Genet*. **2019**, *10*, 1200. https://doi.org/10.3389/fgene.2019.01200].

Supplementary tables

Table S1. The list of genes submitted to the study.

Approved gene symbol	Approved gene name	HGNC ID	Location
ABL1	ABL proto-oncogene 1, non-receptor tyrosine kinase	HGNC:76	9q34.12
ACTL6A	actin like 6A	HGNC:24124	3q26.33
ACTR2	actin related protein 2	HGNC:169	2p14
ACTR5	actin related protein 5	HGNC:14671	20q11.23
ACTR8	actin related protein 8	HGNC:14672	3p21.1
ADPRS	ADP-ribosylserine hydrolase	HGNC:21304	1p34.3
ALKBH1	alkB homolog 1, histone H2A dioxygenase	HGNC:17911	14q24.3
ALKBH2	alkB homolog 2, alpha-ketoglutarate dependent dioxygenase	HGNC:32487	12q24.11
ALKBH3	alkB homolog 3, alpha-ketoglutarate dependent dioxygenase	HGNC:30141	11p11.2
ALKBH4	alkB homolog 4, lysine demethylase	HGNC:21900	7q22.1
ALKBH5	alkB homolog 5, RNA demethylase	HGNC:25996	17p11.2
ALKBH6	alkB homolog 6	HGNC:28243	19q13.12
ALKBH7	alkB homolog 7	HGNC:21306	19p13.3
ALKBH8	alkB homolog 8, tRNA methyltransferase	HGNC:25189	11q22.3
ANKLE1	ankyrin repeat and LEM domain containing 1	HGNC:26812	19p13.11
AP5S1	adaptor related protein complex 5 subunit sigma 1	HGNC:15875	20p13
AP5Z1	adaptor related protein complex 5 subunit zeta 1	HGNC:22197	7p22.1
APBB1	amyloid beta precursor protein binding family B member 1	HGNC:581	11p15.4
APEX1	apurinic/apyrimidinic endodeoxyribonuclease 1	HGNC:587	14q11.2
APEX2	apurinic/apyrimidinic endodeoxyribonuclease 2	HGNC:17889	Xp11.21
APLF	aprataxin and PNKP like factor	HGNC:28724	2p13.3
APTX	aprataxin	HGNC:15984	9p21.1
ARID2	AT-rich interaction domain 2	HGNC:18037	12q12
ASCC1	activating signal cointegrator 1 complex subunit 1	HGNC:24268	10q22.1
ASCC2	activating signal cointegrator 1 complex subunit 2	HGNC:24103	22q12.2
ASCC3	activating signal cointegrator 1 complex subunit 3	HGNC:18697	6q16.3
ASF1A	anti-silencing function 1A histone chaperone	HGNC:20995	6q22.31
ATM	ATM serine/threonine kinase	HGNC:795	11q22.3
ATR	ATR serine/threonine kinase	HGNC:882	3q23
ATRIP	ATR interacting protein	HGNC:33499	3p21.31
ATRX	ATRX chromatin remodeler	HGNC:886	Xq21.1
AUNIP	aurora kinase A and ninein interacting protein	HGNC:28363	1p36.11
AXIN2	axin 2	HGNC:904	17q24.1
BABAM1	BRISC and BRCA1 A complex member 1	HGNC:25008	19p13.11
BABAM2	BRISC and BRCA1 A complex member 2	HGNC:1106	2p23.2
BACH1	BTB domain and CNC homolog 1	HGNC:935	21q21.3
BARD1	BRCA1 associated RING domain 1	HGNC:952	2q35
BCCIP	BRCA2 and CDKN1A interacting protein	HGNC:978	10q26.2
BLM	BLM RecQ like helicase	HGNC:1058	15q26.1
BRCA1	BRCA1 DNA repair associated	HGNC:1100	17q21.31
BRCA2	BRCA2 DNA repair associated	HGNC:1101	13q13.1
BRCC3	BRCA1/BRCA2-containing complex subunit 3	HGNC:24185	Xq28
BRIP1	BRCA1 interacting helicase 1	HGNC:20473	17q23.2
BRME1	break repair meiotic recombinase recruitment factor 1	HGNC:28153	19p13.12
C14orf39	chromosome 14 open reading frame 39	HGNC:19849	14q23.1
CBX8	chromobox 8	HGNC:15962	17q25.3
CCNH	cyclin H	HGNC:1594	5q14.3
CDC14B	cell division cycle 14B	HGNC:1719	9q22.32-q22.33
CDC45	cell division cycle 45	HGNC:1739	22q11.21
CDC5L	cell division cycle 5 like	HGNC:1743	6p21.1
CDC7	cell division cycle 7	HGNC:1745	1p22.1
CDCA5	cell division cycle associated 5	HGNC:14626	11q13.1
CDK2	cyclin dependent kinase 2	HGNC:1771	12q13.2
CDK7	cyclin dependent kinase 7	HGNC:1778	5q13.2
CDK9	cyclin dependent kinase 9	HGNC:1780	9q34.11
CDKN2D	cyclin dependent kinase inhibitor 2D	HGNC:1790	19p13.2

CEBPG	CCAAT enhancer binding protein gamma	HGNC:1837	19q13.11
CENPS	centromere protein S	HGNC:23163	1p36.22
CENPX	centromere protein X	HGNC:11422	17q25.3
CEP164	centrosomal protein 164	HGNC:29182	11q23.3
CETN1	centrin 1	HGNC:1866	18p11.32
CETN2	centrin 2	HGNC:1867	Xq28
CGAS	cyclic GMP-AMP synthase	HGNC:21367	6q13
CHAF1A	chromatin assembly factor 1 subunit A	HGNC:1910	19p13.3
CHAF1B	chromatin assembly factor 1 subunit B	HGNC:1911	21q22.12-q22.13
CHCHD4	coiled-coil-helix-coiled-coil-helix domain containing 4	HGNC:26467	3p25.1
CHD1L	chromodomain helicase DNA binding protein 1 like	HGNC:1916	1q21.1
CHEK1	checkpoint kinase 1	HGNC:1925	11q24.2
CHEK2	checkpoint kinase 2	HGNC:16627	22q12.1
CHRNA4	cholinergic receptor nicotinic alpha 4 subunit	HGNC:1958	20q13.33
CINP	cyclin dependent kinase 2 interacting protein	HGNC:23789	14q32.31
CLK2	CDC like kinase 2	HGNC:2069	1q22
CLSPN	claspin	HGNC:19715	1p34.3
COMMD1	copper metabolism domain containing 1	HGNC:23024	2p15
CUL4A	cullin 4A	HGNC:2554	13q34
CUL4B	cullin 4B	HGNC:2555	Xq24
CYREN	cell cycle regulator of NHEJ	HGNC:22432	7q33
DCLRE1A	DNA cross-link repair 1A	HGNC:17660	10q25.3
DCLRE1B	DNA cross-link repair 1B	HGNC:17641	1p13.2
DCLRE1C	DNA cross-link repair 1C	HGNC:17642	10p13
DDB1	damage specific DNA binding protein 1	HGNC:2717	11q12.2
DDB2	damage specific DNA binding protein 2	HGNC:2718	11p11.2
DDX1	DEAD-box helicase 1	HGNC:2734	2p24.3
DDX11	DEAD/H-box helicase 11	HGNC:2736	12p11.21
DEK	DEK proto-oncogene	HGNC:2768	6p22.3
DHX9	DExH-box helicase 9	HGNC:2750	1q25.3
DMAP1	DNA methyltransferase 1 associated protein 1	HGNC:18291	1p34.1
DMC1	DNA meiotic recombinase 1	HGNC:2927	22q13.1
DNA2	DNA replication helicase/nuclease 2	HGNC:2939	10q21.3
DNPH1	2'-deoxynucleoside 5'-phosphate N-hydrolase 1	HGNC:21218	6p21.1
DNTT	DNA nucleotidylexotransferase	HGNC:2983	10q24.1
DOT1L	DOT1 like histone lysine methyltransferase	HGNC:24948	19p13.3
DTL	denticleless E3 ubiquitin protein ligase homolog	HGNC:30288	1q32.3
DTX3L	deltex E3 ubiquitin ligase 3L	HGNC:30323	3q21.1
DUT	deoxyuridine triphosphatase	HGNC:3078	15q21.1
EEPD1	endonuclease/exonuclease/phosphatase family domain containing 1	HGNC:22223	7p14.2
EGFR	epidermal growth factor receptor	HGNC:3236	7p11.2
EID3	EP300 interacting inhibitor of differentiation 3	HGNC:32961	12q23.3
EME1	essential meiotic structure-specific endonuclease 1	HGNC:24965	17q21.33
EME2	essential meiotic structure-specific endonuclease subunit 2	HGNC:27289	16p13.3
EMSY	EMSY transcriptional repressor, BRCA2 interacting	HGNC:18071	11q13.5
ENDOV	endonuclease V	HGNC:26640	17q25.3
EPC2	enhancer of polycomb homolog 2	HGNC:24543	2q23.1
ERCC1	ERCC excision repair 1, endonuclease non-catalytic subunit	HGNC:3433	19q13.32
ERCC2	ERCC excision repair 2, TFIIH core complex helicase subunit	HGNC:3434	19q13.32
ERCC3	ERCC excision repair 3, TFIIH core complex helicase subunit	HGNC:3435	2q14.3
ERCC4	ERCC excision repair 4, endonuclease catalytic subunit	HGNC:3436	16p13.12
ERCC5	ERCC excision repair 5, endonuclease	HGNC:3437	13q33.1
ERCC6	ERCC excision repair 6, chromatin remodeling factor	HGNC:3438	10q11.23
ERCC6L2	ERCC excision repair 6 like 2	HGNC:26922	9q22.32
ERCC8	ERCC excision repair 8, CSA ubiquitin ligase complex subunit	HGNC:3439	5q12.1
ESCO2	establishment of sister chromatid cohesion N-acetyltransferase 2	HGNC:27230	8p21.1
ETAA1	ETAA1 activator of ATR kinase	HGNC:24648	2p14
EXD2	exonuclease 3'-5' domain containing 2	HGNC:20217	14q24.1
EXO1	exonuclease 1	HGNC:3511	1q43
EXO5	exonuclease 5	HGNC:26115	1p34.2

EYA1	EYA transcriptional coactivator and phosphatase 1	HGNC:3519	8q13.3
EYA2	EYA transcriptional coactivator and phosphatase 2	HGNC:3520	20q13.12
EYA3	EYA transcriptional coactivator and phosphatase 3	HGNC:3521	1p35.3
EYA4	EYA transcriptional coactivator and phosphatase 4	HGNC:3522	6q23.2
FAM111A	FAM111 trypsin like peptidase A	HGNC:24725	11q12.1
FAM168A	family with sequence similarity 168 member A	HGNC:28999	11q13.4
FAN1	FANCD2 and FANCI associated nuclease 1	HGNC:29170	15q13.3
FANCA	FA complementation group A	HGNC:3582	16q24.3
FANCB	FA complementation group B	HGNC:3583	Xp22.2
FANCC	FA complementation group C	HGNC:3584	9q22.32
FANCD2	FA complementation group D2	HGNC:3585	3p25.3
FANCD2OS	FANCD2 opposite strand	HGNC:28623	3p25.3
FANCD2P2	FANCD2 pseudogene 2	HGNC:44488	3p25.2
FANCE	FA complementation group E	HGNC:3586	6p21.31
FANCF	FA complementation group F	HGNC:3587	11p14.3
FANCG	FA complementation group G	HGNC:3588	9p13.3
FANCI	FA complementation group I	HGNC:25568	15q26.1
FANCL	FA complementation group L	HGNC:20748	2p16.1
FANCM	FA complementation group M	HGNC:23168	14q21.2
FBH1	F-box DNA helicase 1	HGNC:13620	10p15.1
FBXO6	F-box protein 6	HGNC:13585	1p36.22
FEN1	flap structure-specific endonuclease 1	HGNC:3650	11q12.2
FGF10	fibroblast growth factor 10	HGNC:3666	5p12
FIGNL1	fidgetin like 1	HGNC:13286	7p12.2
FMN2	formin 2	HGNC:14074	1q43
FOXM1	forkhead box M1	HGNC:3818	12p13.33
FTO	FTO alpha-ketoglutarate dependent dioxygenase	HGNC:24678	16q12.2
FUS	FUS RNA binding protein	HGNC:4010	16p11.2
FZR1	fizzy and cell division cycle 20 related 1	HGNC:24824	19p13.3
GEN1	GEN1 Holliday junction 5' flap endonuclease	HGNC:26881	2p24.2
GGN	gametogenetin	HGNC:18869	19q13.2
GINS2	GINS complex subunit 2	HGNC:24575	16q24.1
GINS4	GINS complex subunit 4	HGNC:28226	8p11.21
GTF2E2	general transcription factor IIE subunit 2	HGNC:4651	8p12
GTF2H1	general transcription factor IIH subunit 1	HGNC:4655	11p15.1
GTF2H2	general transcription factor IIH subunit 2	HGNC:4656	5q13.2
GTF2H3	general transcription factor IIH subunit 3	HGNC:4657	12q24.31
GTF2H4	general transcription factor IIH subunit 4	HGNC:4658	6p21.33
GTF2H5	general transcription factor IIH subunit 5	HGNC:21157	6q25.3
GTF2I	general transcription factor IIi	HGNC:4659	7q11.23
H2AW	H2A.W histone	HGNC:20507	1q42.13
H2AX	H2A.X variant histone	HGNC:4739	11q23.3
HDAC10	histone deacetylase 10	HGNC:18128	22q13.33
HDAC9	histone deacetylase 9	HGNC:14065	7p21.1
HELB	DNA helicase B	HGNC:17196	12q14.3
HELQ	helicase, POLQ like	HGNC:18536	4q21.23
HERC2	HECT and RLD domain containing E3 ubiquitin protein ligase 2	HGNC:4868	15q13.1
HFM1	helicase for meiosis 1	HGNC:20193	1p22.2
HINFP	histone H4 transcription factor	HGNC:17850	11q23.3
HLTF	helicase like transcription factor	HGNC:11099	3q24
HMCES	5-hydroxymethylcytosine binding, ES cell specific	HGNC:24446	3q21.3
HMGA1	high mobility group AT-hook 1	HGNC:5010	6p21.31
HMGA2	high mobility group AT-hook 2	HGNC:5009	12q14.3
HMGB1	high mobility group box 1	HGNC:4983	13q12.3
HMGN1	high mobility group nucleosome binding domain 1	HGNC:4984	21q22.2
HPF1	histone PARylation factor 1	HGNC:26051	4q33
HROB	homologous recombination factor with OB-fold	HGNC:28460	17q21.31
HSF1	heat shock transcription factor 1	HGNC:5224	8q24.3
HSF2BP	heat shock transcription factor 2 binding protein	HGNC:5226	21q22.3
HSPA1A	heat shock protein family A (Hsp70) member 1A	HGNC:5232	6p21.33
HUS1	HUS1 checkpoint clamp component	HGNC:5309	7p12.3

HUS1B	HUS1 checkpoint clamp component B	HGNC:16485	6p25.3
HUWE1	HECT, UBA and WWE domain containing E3 ubiquitin protein ligase 1	HGNC:30892	Xp11.22
IER3	immediate early response 3	HGNC:5392	6p21.33
IFFO1	intermediate filament family orphan 1	HGNC:24970	12p13.31
INIP	INTS3 and NABP interacting protein	HGNC:24994	9q32
INO80	INO80 complex ATPase subunit	HGNC:26956	15q15.1
INO80B	INO80 complex subunit B	HGNC:13324	2p13.1
INO80C	INO80 complex subunit C	HGNC:26994	18q12.2
INO80D	INO80 complex subunit D	HGNC:25997	2q33.3
INTS3	integrator complex subunit 3	HGNC:26153	1q21.3
JMY	junction mediating and regulatory protein, p53 cofactor	HGNC:28916	5q14.1
KASH5	KASH domain containing 5	HGNC:26520	19q13.33
KAT5	lysine acetyltransferase 5	HGNC:5275	11q13.1
KAT7	lysine acetyltransferase 7	HGNC:17016	17q21.33
KDM1A	lysine demethylase 1A	HGNC:29079	1p36.12
KDM2A	lysine demethylase 2A	HGNC:13606	11q13.2
KDM4D	lysine demethylase 4D	HGNC:25498	11q21
KHDC3L	KH domain containing 3 like, subcortical maternal complex member	HGNC:33699	6q13
KIF22	kinesin family member 22	HGNC:6391	16p11.2
KIN	Kin17 DNA and RNA binding protein	HGNC:6327	10p14
KLHL15	kelch like family member 15	HGNC:29347	Xp22.11
KMT5B	lysine methyltransferase 5B	HGNC:24283	11q13.2
KMT5C	lysine methyltransferase 5C	HGNC:28405	19q13.42
LIG1	DNA ligase 1	HGNC:6598	19q13.33
LIG3	DNA ligase 3	HGNC:6600	17q12
LIG4	DNA ligase 4	HGNC:6601	13q33.3
MAD2L2	mitotic arrest deficient 2 like 2	HGNC:6764	1p36.22
MARF1	meiosis regulator and mRNA stability factor 1	HGNC:29562	16p13.11
MBD4	methyl-CpG binding domain 4, DNA glycosylase	HGNC:6919	3q21.3
MC1R	melanocortin 1 receptor	HGNC:6929	16q24.3
MCM2	minichromosome maintenance complex component 2	HGNC:6944	3q21.3
MCM3	minichromosome maintenance complex component 3	HGNC:6945	6p12.2
MCM4	minichromosome maintenance complex component 4	HGNC:6947	8q11.21
MCM5	minichromosome maintenance complex component 5	HGNC:6948	22q12.3
MCM6	minichromosome maintenance complex component 6	HGNC:6949	2q21.3
MCM7	minichromosome maintenance complex component 7	HGNC:6950	7q22.1
MCM8	minichromosome maintenance 8 homologous recombination repair factor	HGNC:16147	20p12.3
MCM9	minichromosome maintenance 9 homologous recombination repair factor	HGNC:21484	6q22.31
MCMDC2	minichromosome maintenance domain containing 2	HGNC:26368	8q13.1
MCRS1	microspherule protein 1	HGNC:6960	12q13.12
MDC1	mediator of DNA damage checkpoint 1	HGNC:21163	6p21.33
MEIOB	meiosis specific with OB-fold	HGNC:28569	16p13.3
MEIOC	meiosis specific with coiled-coil domain	HGNC:26670	17q21.31
MGME1	mitochondrial genome maintenance exonuclease 1	HGNC:16205	20p11.23
MGMT	O-6-methylguanine-DNA methyltransferase	HGNC:7059	10q26.3
MLH1	mutL homolog 1	HGNC:7127	3p22.2
MLH3	mutL homolog 3	HGNC:7128	14q24.3
MMS19	MMS19 homolog, cytosolic iron-sulfur assembly component	HGNC:13824	10q24.1
MMS22L	MMS22 like, DNA repair protein	HGNC:21475	6q16.1
MNAT1	MNAT1 component of CDK activating kinase	HGNC:7181	14q23.1
MORF4L1	mortality factor 4 like 1	HGNC:16989	15q25.1
MORF4L2	mortality factor 4 like 2	HGNC:16849	Xq22.2
MPG	N-methylpurine DNA glycosylase	HGNC:7211	16p13.3
MPLKIP	M-phase specific PLK1 interacting protein	HGNC:16002	7p14.1
MRE11	MRE11 homolog, double strand break repair nuclease	HGNC:7230	11q21
MRNIP	MRN complex interacting protein	HGNC:30817	5q35.3
MSH2	mutS homolog 2	HGNC:7325	2p21-p16.3

MSH3	mutS homolog 3	HGNC:7326	5q14.1
MSH4	mutS homolog 4	HGNC:7327	1p31.1
MSH5	mutS homolog 5	HGNC:7328	6p21.33
MSH6	mutS homolog 6	HGNC:7329	2p16.3
MTA1	metastasis associated 1	HGNC:7410	14q32.33
MUS81	MUS81 structure-specific endonuclease subunit	HGNC:29814	11q13
MUTYH	mutY DNA glycosylase	HGNC:7527	1p34.1
NABP1	nucleic acid binding protein 1	HGNC:26232	2q32.3
NABP2	nucleic acid binding protein 2	HGNC:28412	12q13.3
NBN	nibrin	HGNC:7652	8q21.3
NEIL1	nei like DNA glycosylase 1	HGNC:18448	15q24.2
NEIL2	nei like DNA glycosylase 2	HGNC:18956	8p23.1
NEIL3	nei like DNA glycosylase 3	HGNC:24573	4q34.3
NFRKB	nuclear factor related to kappaB binding protein	HGNC:7802	11q24.3
NHEJ1	non-homologous end joining factor 1	HGNC:25737	2q35
NIPBL	NIPBL cohesin loading factor	HGNC:28862	5p13.2
NONO	non-POU domain containing octamer binding	HGNC:7871	Xq13.1
NOP53	NOP53 ribosome biogenesis factor	HGNC:4333	19q13.33
NPAS2	neuronal PAS domain protein 2	HGNC:7895	2q11.2
NPM1	nucleophosmin 1	HGNC:7910	5q35.1
NSD2	nuclear receptor binding SET domain protein 2	HGNC:12766	4p16.3
NSMCE1	NSE1 homolog, SMC5-SMC6 complex component	HGNC:29897	16p12.1
NSMCE2	NSE2 (MMS21) homolog, SMC5-SMC6 complex SUMO ligase	HGNC:26513	8q24.13
NSMCE3	NSE3 homolog, SMC5-SMC6 complex component	HGNC:7677	15q13.1
NSMCE4A	NSE4 homolog A, SMC5-SMC6 complex component	HGNC:25935	10q26.13
NTHL1	nth like DNA glycosylase 1	HGNC:8028	16p13.3
NUCKS1	nuclear casein kinase and cyclin dependent kinase substrate 1	HGNC:29923	1q32.1
NUDT1	nudix hydrolase 1	HGNC:8048	7p22.3
NUDT15	nudix hydrolase 15	HGNC:23063	13q14.2
NUDT16L1	nudix hydrolase 16 like 1	HGNC:28154	16p13.3
NUDT18	nudix hydrolase 18	HGNC:26194	8p21.3
OGG1	8-oxoguanine DNA glycosylase	HGNC:8125	3p25.3
OOEP	oocyte expressed protein	HGNC:21382	6q13
OTUB1	OTU deubiquitinase, ubiquitin aldehyde binding 1	HGNC:23077	11q13.1
OTUB2	OTU deubiquitinase, ubiquitin aldehyde binding 2	HGNC:20351	14q32.12
PALB2	partner and localizer of BRCA2	HGNC:26144	16p12.2
PARG	poly(ADP-ribose) glycohydrolase	HGNC:8605	10q11.23
PARK7	Parkinsonism associated deglycase	HGNC:16369	1p36.23
PARP1	poly(ADP-ribose) polymerase 1	HGNC:270	1q42.12
PARP10	poly(ADP-ribose) polymerase family member 10	HGNC:25895	8q24.3
PARP2	poly(ADP-ribose) polymerase 2	HGNC:272	14q11.2
PARP3	poly(ADP-ribose) polymerase family member 3	HGNC:273	3p21.2
PARP9	poly(ADP-ribose) polymerase family member 9	HGNC:24118	3q21.1
PARPBP	PARP1 binding protein	HGNC:26074	12q23.2
PAXIP1	PAX interacting protein 1	HGNC:8624	7q36.2
PAXX	PAXX non-homologous end joining factor	HGNC:27849	9q34.3
PCLAF	PCNA clamp associated factor	HGNC:28961	15q22.31
PCNA	proliferating cell nuclear antigen	HGNC:8729	20p12.3
PDS5A	PDS5 cohesin associated factor A	HGNC:29088	4p14
PDS5B	PDS5 cohesin associated factor B	HGNC:20418	13q13.1
PER1	period circadian regulator 1	HGNC:8845	17p13.1
PIF1	PIF1 5'-to-3' DNA helicase	HGNC:26220	15q22.31
PML	PML nuclear body scaffold	HGNC:9113	15q24.1
PMS1	PMS1 homolog 1, mismatch repair system component	HGNC:9121	2q32.2
PMS2	PMS1 homolog 2, mismatch repair system component	HGNC:9122	7p22.1
PMS2P3	PMS1 homolog 2, mismatch repair system component pseudogene 3	HGNC:9128	7q11.23
PNKP	polynucleotide kinase 3'-phosphatase	HGNC:9154	19q13.33
PNP	purine nucleoside phosphorylase	HGNC:7892	14q11.2
POLA1	DNA polymerase alpha 1, catalytic subunit	HGNC:9173	Xp22.11-p21.3
POLB	DNA polymerase beta	HGNC:9174	8p11.21

POLD1	DNA polymerase delta 1, catalytic subunit	HGNC:9175	19q13.3
POLD2	DNA polymerase delta 2, accessory subunit	HGNC:9176	7p13
POLD3	DNA polymerase delta 3, accessory subunit	HGNC:20932	11q13.4
POLD4	DNA polymerase delta 4, accessory subunit	HGNC:14106	11q13.2
POLDIP2	DNA polymerase delta interacting protein 2	HGNC:23781	17q11.2
POLE	DNA polymerase epsilon, catalytic subunit	HGNC:9177	12q24.33
POLE2	DNA polymerase epsilon 2, accessory subunit	HGNC:9178	14q21.3
POLE3	DNA polymerase epsilon 3, accessory subunit	HGNC:13546	9q32
POLE4	DNA polymerase epsilon 4, accessory subunit	HGNC:18755	2p12
POLG	DNA polymerase gamma, catalytic subunit	HGNC:9179	15q26.1
POLG2	DNA polymerase gamma 2, accessory subunit	HGNC:9180	17q23.3
POLH	DNA polymerase eta	HGNC:9181	6p21.1
POLI	DNA polymerase iota	HGNC:9182	18q21.2
POLK	DNA polymerase kappa	HGNC:9183	5q13.3
POLL	DNA polymerase lambda	HGNC:9184	10q24.32
POLM	DNA polymerase mu	HGNC:9185	7p13
POLN	DNA polymerase nu	HGNC:18870	4p16.3
POLQ	DNA polymerase theta	HGNC:9186	3q13.33
POLR2I	RNA polymerase II subunit I	HGNC:9196	19q13.12
PPP4C	protein phosphatase 4 catalytic subunit	HGNC:9319	16p11.2
PPP4R2	protein phosphatase 4 regulatory subunit 2	HGNC:18296	3p13
PRDM9	PR/SET domain 9	HGNC:13994	5p14.2
PRIMPOL	primase and DNA directed polymerase	HGNC:26575	4q35.1
PRKCG	protein kinase C gamma	HGNC:9402	19q13.42
PRKDC	protein kinase, DNA-activated, catalytic subunit	HGNC:9413	8q11.21
PRMT6	protein arginine methyltransferase 6	HGNC:18241	1p13.3
PRPF19	pre-mRNA processing factor 19	HGNC:17896	11q12.2
PRRX1	paired related homeobox 1	HGNC:9142	1q24.2
PSMD14	proteasome 26S subunit, non-ATPase 14	HGNC:16889	2q24.2
PSME4	proteasome activator subunit 4	HGNC:20635	2p16.2
PTTG1	PTTG1 regulator of sister chromatid separation, securin	HGNC:9690	5q33.3
PWWP3A	PWWP domain containing 3A, DNA repair factor	HGNC:29641	19p13.3
RAD1	RAD1 checkpoint DNA exonuclease	HGNC:9806	5p13.2
RAD17	RAD17 checkpoint clamp loader component	HGNC:9807	5q13.2
RAD17P1	RAD17 pseudogene 1	HGNC:9808	7p21.1
RAD17P2	RAD17 pseudogene 2	HGNC:9809	13q14.2
RAD18	RAD18 E3 ubiquitin protein ligase	HGNC:18278	3p25.3
RAD21	RAD21 cohesin complex component	HGNC:9811	8q24.11
RAD21-AS1	RAD21 antisense RNA 1	HGNC:32158	8q24.11
RAD21L1	RAD21 cohesin complex component like 1	HGNC:16271	20p13
RAD23A	RAD23 homolog A, nucleotide excision repair protein	HGNC:9812	19p13.13
RAD23B	RAD23 homolog B, nucleotide excision repair protein	HGNC:9813	9q31.2
RAD23BP1	RAD23B pseudogene 1	HGNC:44509	3p24.3
RAD23BP2	RAD23B pseudogene 2	HGNC:44510	7q21.11
RAD23BP3	RAD23B pseudogene 3	HGNC:9814	21q21.1
RAD50	RAD50 double strand break repair protein	HGNC:9816	5q31.1
RAD51	RAD51 recombinase	HGNC:9817	15q15.1
RAD51AP1	RAD51 associated protein 1	HGNC:16956	12p13.32
RAD51AP2	RAD51 associated protein 2	HGNC:34417	2p24.2
RAD51-AS1	RAD51 antisense RNA 1	HGNC:48621	15q15.1
RAD51B	RAD51 paralog B	HGNC:9822	14q24.1
RAD51C	RAD51 paralog C	HGNC:9820	17q22
RAD51D	RAD51 paralog D	HGNC:9823	17q12
RAD51L3-RFFL	Unassigned		
RAD52	RAD52 homolog, DNA repair protein	HGNC:9824	12p13.33
RAD54B	RAD54 homolog B	HGNC:17228	8q22.1
RAD54L	RAD54 like	HGNC:9826	1p34.1
RAD54L2	RAD54 like 2	HGNC:29123	3p21.2
RAD9A	RAD9 checkpoint clamp component A	HGNC:9827	11q13.2
RAD9B	RAD9 checkpoint clamp component B	HGNC:21700	12q24.11
RADX	RPA1 related single stranded DNA binding protein, X-linked	HGNC:25486	Xq22.3

RBBP8	RB binding protein 8, endonuclease	HGNC:9891	18q11.2
RBX1	ring-box 1	HGNC:9928	22q13.2
RDM1	RAD52 motif containing 1	HGNC:19950	17q12
REC8	REC8 meiotic recombination protein	HGNC:16879	14q12
RECQL	RecQ like helicase	HGNC:9948	12p12.1
RECQL4	RecQ like helicase 4	HGNC:9949	8q24.3
RECQL5	RecQ like helicase 5	HGNC:9950	17q25
REV1	REV1 DNA directed polymerase	HGNC:14060	2q11.2
REV3L	REV3 like, DNA directed polymerase zeta catalytic subunit	HGNC:9968	6q21
REXO4	REX4 homolog, 3'-5' exonuclease	HGNC:12820	9q34.2
RFC1	replication factor C subunit 1	HGNC:9969	4p14
RFC2	replication factor C subunit 2	HGNC:9970	7q11.23
RFC3	replication factor C subunit 3	HGNC:9971	13q13.2
RFC4	replication factor C subunit 4	HGNC:9972	3q27.3
RFC5	replication factor C subunit 5	HGNC:9973	12q24.23
RFWD3	ring finger and WD repeat domain 3	HGNC:25539	16q23.1
RHNO1	RAD9-HUS1-RAD1 interacting nuclear orphan 1	HGNC:28206	12p13.33
RIF1	replication timing regulatory factor 1	HGNC:23207	2q23.3
RMI1	RecQ mediated genome instability 1	HGNC:25764	9q21.32
RMI2	RecQ mediated genome instability 2	HGNC:28349	16p13.13
RNASEH2A	ribonuclease H2 subunit A	HGNC:18518	19p13.13
RNF111	ring finger protein 111	HGNC:17384	15q22.1-q22.2
RNF138	ring finger protein 138	HGNC:17765	18q12.1
RNF168	ring finger protein 168	HGNC:26661	3q29
RNF169	ring finger protein 169	HGNC:26961	11q13.4
RNF4	ring finger protein 4	HGNC:10067	4p16.3
RNF8	ring finger protein 8	HGNC:10071	6p21.2
RPA1	replication protein A1	HGNC:10289	17p13.3
RPA2	replication protein A2	HGNC:10290	1p35.3
RPA3	replication protein A3	HGNC:10291	7p21.3
RPA4	replication protein A4	HGNC:30305	Xq21.33
RPAIN	RPA interacting protein	HGNC:28641	17p13.2
RPS3	ribosomal protein S3	HGNC:10420	11q13.4
RRM2B	ribonucleotide reductase regulatory TP53 inducible subunit M2B	HGNC:17296	8q22.3
RTEL1	regulator of telomere elongation helicase 1	HGNC:15888	20q13.33
RUVBL1	RuvB like AAA ATPase 1	HGNC:10474	3q21.3
RUVBL2	RuvB like AAA ATPase 2	HGNC:10475	19q13.33
SAMHD1	SAM and HD domain containing deoxynucleoside triphosphate triphosphohydrolase 1	HGNC:15925	20q11.23
SEM1	SEM1 26S proteasome subunit	HGNC:10845	7q21.3
SEM1/C7orf76	Unassigned		
SETD2	SET domain containing 2, histone lysine methyltransferase	HGNC:18420	3p21.31
SETMAR	SET domain and mariner transposase fusion gene	HGNC:10762	3p26.1
SETX	senataxin	HGNC:445	9q34.13
SFPQ	splicing factor proline and glutamine rich	HGNC:10774	1p34.3
SFR1	SWI5 dependent homologous recombination repair protein 1	HGNC:29574	10q25.1
SHPRH	SNF2 histone linker PHD RING helicase	HGNC:19336	6q24.3
SIRT1	sirtuin 1	HGNC:14929	10q21.3
SIRT6	sirtuin 6	HGNC:14934	19p13.3
SIRT7	sirtuin 7	HGNC:14935	17q25.3
SLF1	SMC5-SMC6 complex localization factor 1	HGNC:25408	5q15
SLF2	SMC5-SMC6 complex localization factor 2	HGNC:17814	10q24.31
SLX1A	SLX1 homolog A, structure-specific endonuclease subunit	HGNC:20922	16p11.2
SLX1B	SLX1 homolog B, structure-specific endonuclease subunit	HGNC:28748	16p11.2
SLX4	SLX4 structure-specific endonuclease subunit	HGNC:23845	16p13.3
SMARCAD1	SWI/SNF-related, matrix-associated actin-dependent regulator of chromatin, subfamily a, containing DEAD/H box 1	HGNC:18398	4q22.3
SMARCAL1	SWI/SNF related, matrix associated, actin dependent regulator of chromatin, subfamily a like 1	HGNC:11102	2q35
SMC1A	structural maintenance of chromosomes 1A	HGNC:11111	Xp11.22

SMC2	structural maintenance of chromosomes 2	HGNC:14011	9q31.1
SMC3	structural maintenance of chromosomes 3	HGNC:2468	10q25.2
SMC4	structural maintenance of chromosomes 4	HGNC:14013	3q25.33
SMC5	structural maintenance of chromosomes 5	HGNC:20465	9q21.12
SMC6	structural maintenance of chromosomes 6	HGNC:20466	2p24.2
SMCHD1	structural maintenance of chromosomes flexible hinge domain containing 1	HGNC:29090	18p11.32
SMG1	SMG1 nonsense mediated mRNA decay associated PI3K related kinase	HGNC:30045	16p12.3
SMUG1	single-strand-selective monofunctional uracil-DNA glycosylase 1	HGNC:17148	12q13.13
SPATA22	spermatogenesis associated 22	HGNC:30705	17p13.2
SPIDR	scaffold protein involved in DNA repair	HGNC:28971	8q11.21
SPIRE1	spire type actin nucleation factor 1	HGNC:30622	18p11.21
SPIRE2	spire type actin nucleation factor 2	HGNC:30623	16q24.3
SPO11	SPO11 initiator of meiotic double stranded breaks	HGNC:11250	20q13.31
SPRTN	SprT-like N-terminal domain	HGNC:25356	1q42.2
SSRP1	structure specific recognition protein 1	HGNC:11327	11q12.1
STUB1	STIP1 homology and U-box containing protein 1	HGNC:11427	16p13.3
SUV39H1	SUV39H1 histone lysine methyltransferase	HGNC:11479	Xp11.23
SWI5	SWI5 homologous recombination repair protein	HGNC:31412	9q34.11
SWSAP1	SWIM-type zinc finger 7 associated protein 1	HGNC:26638	19p13.2
SYCP1	synaptonemal complex protein 1	HGNC:11487	1p13.2
SYCP3	synaptonemal complex protein 3	HGNC:18130	12q23.2
TAOK1	TAO kinase 1	HGNC:29259	17q11.2
TAOK3	TAO kinase 3	HGNC:18133	12q24.23
TDG	thymine DNA glycosylase	HGNC:11700	12q23.3
TDP1	tyrosyl-DNA phosphodiesterase 1	HGNC:18884	14q32.11
TDP2	tyrosyl-DNA phosphodiesterase 2	HGNC:17768	6p22.3
TERF2IP	TERF2 interacting protein	HGNC:19246	16q23.1
TEX12	testis expressed 12	HGNC:11734	11q23.1
TEX15	testis expressed 15, meiosis and synapsis associated	HGNC:11738	8p12
TEX264	testis expressed 264, ER-phagy receptor	HGNC:30247	3p21.2
TFPT	TCF3 fusion partner	HGNC:13630	19q13.42
TICRR	TOPBP1 interacting checkpoint and replication regulator	HGNC:28704	15q26.1
TIGAR	TP53 induced glycolysis regulatory phosphatase	HGNC:1185	12p13.32
TIMELESS	timeless circadian regulator	HGNC:11813	12q13.3
TMEM161A	transmembrane protein 161A	HGNC:26020	19p13.11
TNKS1BP1	tankyrase 1 binding protein 1	HGNC:19081	11q12.1
TNP1	transition protein 1	HGNC:11951	2q35
TONSL	tonsoku like, DNA repair protein	HGNC:7801	8q24.3
TOP1	DNA topoisomerase I	HGNC:11986	20q12
TOP2A	DNA topoisomerase II alpha	HGNC:11989	17q21.2
TOP3A	DNA topoisomerase III alpha	HGNC:11992	17p11.2
TOPBP1	DNA topoisomerase II binding protein 1	HGNC:17008	3q22.1
TP53	tumor protein p53	HGNC:11998	17p13.1
TP53BP1	tumor protein p53 binding protein 1	HGNC:11999	15q15.3
TRAIP	TRAF interacting protein	HGNC:30764	3p21.31
TREX1	three prime repair exonuclease 1	HGNC:12269	3p21.31
TREX2	three prime repair exonuclease 2	HGNC:12270	Xq28
TRIM28	tripartite motif containing 28	HGNC:16384	19q13.43
TRIP12	thyroid hormone receptor interactor 12	HGNC:12306	2q36.3
TRIP13	thyroid hormone receptor interactor 13	HGNC:12307	5p15.33
TRPC2	transient receptor potential cation channel subfamily C member 2 (pseudogene)	HGNC:12334	11p15.4
TRRAP	transformation/transcription domain associated protein	HGNC:12347	7q22.1
TTC5	tetratricopeptide repeat domain 5	HGNC:19274	14q11.2
TWIST1	twist family bHLH transcription factor 1	HGNC:12428	7p21.1
UBE2A	ubiquitin conjugating enzyme E2 A	HGNC:12472	Xq24
UBE2B	ubiquitin conjugating enzyme E2 B	HGNC:12473	5q31.1
UBE2D3	ubiquitin conjugating enzyme E2 D3	HGNC:12476	4q24
UBE2N	ubiquitin conjugating enzyme E2 N	HGNC:12492	12q22

UBE2T	ubiquitin conjugating enzyme E2 T	HGNC:25009	1q32.1
UBE2V1	ubiquitin conjugating enzyme E2 V1	HGNC:12494	20q13.13
UBE2V2	ubiquitin conjugating enzyme E2 V2	HGNC:12495	8q11.21
UBE2W	ubiquitin conjugating enzyme E2 W	HGNC:25616	8q21.11
UBQLN4	ubiquilin 4	HGNC:1237	1q22
UBR5	ubiquitin protein ligase E3 component n-recognin 5	HGNC:16806	8q22.3
UCHL5	ubiquitin C-terminal hydrolase L5	HGNC:19678	1q31.2
UFL1	UFM1 specific ligase 1	HGNC:23039	6q16.1
UHRF1	ubiquitin like with PHD and ring finger domains 1	HGNC:12556	19p13.3
UIMC1	ubiquitin interaction motif containing 1	HGNC:30298	5q35.2
UNG	uracil DNA glycosylase	HGNC:12572	12q24.11
UPF1	UPF1 RNA helicase and ATPase	HGNC:9962	19p13.11
USP1	ubiquitin specific peptidase 1	HGNC:12607	1p31.3
USP10	ubiquitin specific peptidase 10	HGNC:12608	16q24.1
USP28	ubiquitin specific peptidase 28	HGNC:12625	11q23.2
USP3	ubiquitin specific peptidase 3	HGNC:12626	15q22.31
USP45	ubiquitin specific peptidase 45	HGNC:20080	6q16.2
USP47	ubiquitin specific peptidase 47	HGNC:20076	11p15.3
USP51	ubiquitin specific peptidase 51	HGNC:23086	Xp11.21
USP7	ubiquitin specific peptidase 7	HGNC:12630	16p13.2
UVRAG	UV radiation resistance associated	HGNC:12640	11q13.5
UVSSA	UV stimulated scaffold protein A	HGNC:29304	4p16.3
VCP	valosin containing protein	HGNC:12666	9p13.3
VCPIP1	valosin containing protein interacting protein 1	HGNC:30897	8q13.1
WAS	WASP actin nucleation promoting factor	HGNC:12731	Xp11.23
WDHD1	WD repeat and HMG-box DNA binding protein 1	HGNC:23170	14q22.2-q22.3
WDR48	WD repeat domain 48	HGNC:30914	3p22.2
WDR70	WD repeat domain 70	HGNC:25495	5p13.2
WRAP53	WD repeat containing antisense to TP53	HGNC:25522	17p13.1
WRN	WRN RecQ like helicase	HGNC:12791	8p12
WRNIP1	WRN helicase interacting protein 1	HGNC:20876	6p25.2
XAB2	XPA binding protein 2	HGNC:14089	19p13.2
XPA	XPA, DNA damage recognition and repair factor	HGNC:12814	9q22.33
XPC	XPC complex subunit,<br>DNA damage recognition and repair factor	HGNC:12816	3p25.1
XRCC1	X-ray repair cross complementing 1	HGNC:12828	19q13.31
XRCC2	X-ray repair cross complementing 2	HGNC:12829	7q36.1
XRCC3	X-ray repair cross complementing 3	HGNC:12830	14q32.33
XRCC4	X-ray repair cross complementing 4	HGNC:12831	5q14.2
XRCC5	X-ray repair cross complementing 5	HGNC:12833	2q35
XRCC6	X-ray repair cross complementing 6	HGNC:4055	22q13.2
XRN2	5'-3' exoribonuclease 2	HGNC:12836	20p11.22
YY1	YY1 transcription factor	HGNC:12856	14q32.2
ZBTB1	zinc finger and BTB domain containing 1	HGNC:20259	14q23.3
ZBTB7A	zinc finger and BTB domain containing 7A	HGNC:18078	19p13.3
ZCWPW1	zinc finger CW-type and PWWP domain containing 1	HGNC:23486	7q22.1
ZFYVE26	zinc finger FYVE-type containing 26	HGNC:20761	14q24.1
ZMPSTE24	zinc metallopeptidase STE24	HGNC:12877	1p34.2
ZRANB3	zinc finger RANBP2-type containing 3	HGNC:25249	2q21.3
ZSWIM7	zinc finger SWIM-type containing 7	HGNC:26993	17p12

Gene symbols, gene names, genomic location and gene identificators of HUGO Gene Nomenclature Committee database (HGNC IDs) were collected from HUGO Gene Nomenclature Committee database (https://www.genenames.org/). [1] NCBI Entrez Gene ID numer (https://www.ncbi.nlm.nih.gov/gene/100529207)

Table S2. Differential expression parameters of genes indicated by DESeq2 method with *p* values (FDR with Benjamini-Hochberg correction) below 0.05. Genes were ordered within each comparison according to increasing *p* value.

Gene	log2FoldChange	*pvalue*	*padj*	Fold change
**PAIRWISE COMPARISONS**				
**LEAD vs Control**				
ATM	-0,41723	9,10 × 10^{-5}	4.711 × 10^{-2}	0,748859
**AAA vs Control**				
	log2FoldChange	pvalue	padj	Fold change
RAD21	-0,40105	9,51 × 10^{-10}	4.926 × 10^{-7}	0,757307
UPF1	0,362531	2,70 × 10^{-7}	7.004 × 10^{-5}	1,285679
SMARCAD1	-0,46414	1,31 × 10^{-6}	2.260 × 10^{-4}	0,724905
WHSC1	0,378448	3,79 × 10^{-6}	4.903 × 10^{-4}	1,299942
TOP3A	0,400012	2,57 × 10^{-5}	2.659 × 10^{-3}	1,319519
INTS3	0,294797	4,40 × 10^{-5}	3.802 × 10^{-3}	1,226713
HMGN1	-0,44373	8,40 × 10^{-5}	3.955 × 10^{-3}	0,735229
PNKP	0,390179	6,08 × 10^{-5}	3.955 × 10^{-3}	1,310556
PRRX1	-2,26462	6,23 × 10^{-5}	3.955 × 10^{-3}	0,208104
UVSSA	0,567737	8,00 × 10^{-5}	3.955 × 10^{-3}	1,482197
ZBTB1	-0,36226	7,64 × 10^{-5}	3.955 × 10^{-3}	0,777945
HFM1	-2,21519	0,000125	4.303 × 10^{-3}	0,215359
PARP2	-0,53077	0,00011	4.303 × 10^{-3}	0,692187
RNF138	-0,57782	0,00012	4.303 × 10^{-3}	0,669974
SMC3	-0,33176	0,000101	4.303 × 10^{-3}	0,794567
NBN	-0,3754	0,000201	6.504 × 10^{-3}	0,77089
OGG1	0,393617	0,000263	8.026 × 10^{-3}	1,313683
ATM	-0,39405	0,000341	8.031× 10^{-3}	0,760993
EGFR	-1,99835	0,000341	8.031× 10^{-3}	0,250285
FMN2	-2,03292	0,000315	8.031× 10^{-3}	0,24436
MORF4L2	-0,28806	0,00033	8.031× 10^{-3}	0,819004
NIPBL	-0,21621	0,000283	8.031× 10^{-3}	0,860826
PPP4R2	-0,2712	0,000373	8.402 × 10^{-3}	0,828631
ASF1A	-0,5542	0,000406	8.587 × 10^{-3}	0,681036
ERCC5	-0,28927	0,000431	8.587 × 10^{-3}	0,818317
USP10	0,24772	0,000423	8.587 × 10^{-3}	1,187329
ERCC1	0,331665	0,000476	9.127 × 10^{-3}	1,258465
RTEL1	0,851731	0,000539	9.642 × 10^{-3}	1,804665
TRPC2	1,924183	0,00054	9.642 × 10^{-3}	3,79522
APBB1	-0,64794	0,000656	1.132 × 10^{-2}	0,638189
NPAS2	-1,38219	0,000708	1.145 × 10^{-2}	0,383636
USP1	-0,31497	0,000696	1.145 × 10^{-2}	0,803868
DEK	-0,33298	0,000741	1.163 × 10^{-2}	0,793897
PDS5A	-0,29446	0,000771	1.174 × 10^{-2}	0,815377
CUL4B	-0,26509	0,001048	1.507 × 10^{-2}	0,83215
MSH2	-0,46288	0,001046	1.507 × 10^{-2}	0,725538

POLE	0,304591	0,001233	1.656×10^{-2}	1,235069
SIRT7	0,305894	0,001247	1.656×10^{-2}	1,236185
USP51	-0,88518	0,001216	1.656×10^{-2}	0,541419
ETAA1	-0,46611	0,001486	1.876×10^{-2}	0,723916
POLE4	0,404786	0,001458	1.876×10^{-2}	1,323892
PDS5B	-0,25063	0,001534	1.891×10^{-2}	0,840531
HERC2	0,267121	0,001607	1.891×10^{-2}	1,203404
RAD54L2	0,21683	0,001576	1.891×10^{-2}	1,162177
PIF1	1,042317	0,001788	2.058×10^{-2}	2,059533
FBXO18	0,261635	0,002109	2.374×10^{-2}	1,198837
AP5S1	0,433489	0,002198	2.422×10^{-2}	1,350496
CDK9	0,269785	0,002375	2.562×10^{-2}	1,205628
AP5Z1	0,307846	0,002534	2.655×10^{-2}	1,237858
SPIDR	0,307129	0,002564	2.655×10^{-2}	1,237243
UBE2B	-0,40644	0,002735	2.777×10^{-2}	0,754482
TRRAP	0,236905	0,00286	2.849×10^{-2}	1,178462
JMY	-0,38347	0,002941	2.874×10^{-2}	0,766589
PMS1	-0,35777	0,003177	3.047×10^{-2}	0,78037
VCP	0,274332	0,003287	3.095×10^{-2}	1,209434
USP47	-0,2679	0,003474	3.213×10^{-2}	0,83053
ERCC6L2	-0,24055	0,003674	3.338×10^{-2}	0,846425
CUL4A	0,21448	0,004216	3.665×10^{-2}	1,160286
RAD1	-0,30741	0,004246	3.665×10^{-2}	0,808093
WAS	0,363713	0,004123	3.665×10^{-2}	1,286733
HUWE1	0,195543	0,004719	4.007×10^{-2}	1,145155
IFFO1	0,332808	0,005258	4.393×10^{-2}	1,259462
**CVD vs Control**				
	log2FoldChange	pvalue	padj	Fold change
POLE	0,474205	$5{,}31 \times 10^{-7}$	2.750×10^{-4}	1,389152
UVSSA	0,685032	$2{,}03 \times 10^{-6}$	5.268×10^{-4}	1,607737
RAD21	-0,30128	$5{,}06 \times 10^{-6}$	8.728×10^{-4}	0,81153
PNKP	0,404384	$3{,}67 \times 10^{-5}$	3.628×10^{-3}	1,323524
RAD17P1	-2,05537	$4{,}20 \times 10^{-5}$	3.628×10^{-3}	0,240587
WHSC1	0,341185	$3{,}54 \times 10^{-5}$	3.628×10^{-3}	1,266797
XRCC6	-0,37127	$5{,}26 \times 10^{-5}$	3.894×10^{-3}	0,773104
TRRAP	0,318968	$6{,}08 \times 10^{-5}$	3.937×10^{-3}	1,247437
AP5Z1	0,399989	$9{,}24 \times 10^{-5}$	4.601×10^{-3}	1,319498
EME2	0,531217	$9{,}77 \times 10^{-5}$	4.601×10^{-3}	1,445148
UPF1	0,276538	$9{,}67 \times 10^{-5}$	4.601×10^{-3}	1,211284
NIPBL	-0,22902	0,000132	5.686×10^{-3}	0,853213
NBN	-0,38221	0,000179	7.122×10^{-3}	0,767261
USP1	-0,34824	0,000237	8.767×10^{-3}	0,785544
DTX3L	-0,76664	0,000307	1.061×10^{-2}	0,587784
GTF2I	-0,41902	0,000344	1.106×10^{-2}	0,747935
RAD23BP1	-0,773	0,000363	1.106×10^{-2}	0,585199

HERC2	0,296997	0,000474	1.291×10^{-2}	1,228584
TREX2	1,027979	0,000455	1.291×10^{-2}	2,039166
HMGN1	-0,39545	0,000503	1.303×10^{-2}	0,760251
EGFR	-2,03662	0,000566	1.396×10^{-2}	0,243735
OGG1	0,358079	0,001006	2.368×10^{-2}	1,281718
PARP2	-0,46311	0,001106	2.490×10^{-2}	0,72542
NFRKB	0,321013	0,001213	2.618×10^{-2}	1,249208
MORF4L2	-0,26296	0,001301	2.696×10^{-2}	0,833374
UBE2V2	-0,3237	0,001488	2.964×10^{-2}	0,799018
BCCIP	-0,34408	0,001688	3.014×10^{-2}	0,787812
DEK	-0,31284	0,001661	3.014×10^{-2}	0,805053
MMS19	0,245314	0,001582	3.014×10^{-2}	1,185351
IER3	-1,25156	0,001832	3.163×10^{-2}	0,419993
FZR1	0,322427	0,002094	3.418×10^{-2}	1,250432
HSPA1A	-0,90694	0,002176	3.418×10^{-2}	0,533316
POLL	0,256203	0,002178	3.418×10^{-2}	1,194331
GTF2H3	-0,42134	0,002264	3.449×10^{-2}	0,746729
ARID2	0,230381	0,00248	3.669×10^{-2}	1,173144
SLX1B	-0,88237	0,003207	4.489×10^{-2}	0,542476
TP53	-0,31147	0,003122	4.489×10^{-2}	0,805823
TDP2	-0,24488	0,003556	4.847×10^{-2}	0,843884
TERF2IP	-0,30493	0,003735	4.960×10^{-2}	0,809483
**LEAD vs AAA**				
	log2FoldChange	pvalue	padj	Fold change
PPP4R2	0,416809	$5{,}50 \times 10^{-9}$	2.732×10^{-6}	1,334971
RAD21	0,346806	$3{,}64 \times 10^{-8}$	9.034×10^{-6}	1,271742
SMC3	0,371609	$4{,}49 \times 10^{-6}$	7.431×10^{-4}	1,293795
POLE3	0,406956	$4{,}46 \times 10^{-5}$	5.547×10^{-3}	1,325885
MCM7	-0,34653	$9{,}93 \times 10^{-5}$	9.869×10^{-3}	0,786475
CLK2	-0,37008	0,000203	1.350×10^{-2}	0,77374
DEK	0,349259	0,000227	1.350×10^{-2}	1,273906
SMARCAD1	0,339007	0,000209	1.350×10^{-2}	1,264886
UVSSA	-0,50794	0,000245	1.350×10^{-2}	0,703224
FANCB	0,676715	0,000552	2.563×10^{-2}	1,598496
PNKP	-0,32011	0,000567	2.563×10^{-2}	0,801009
ERCC1	-0,30619	0,00072	2.981×10^{-2}	0,808775
MCM5	-0,29174	0,001107	3.975×10^{-2}	0,816916
TWIST1	2,361488	0,0012	3.975×10^{-2}	5,139001
UBE2A	0,368431	0,001164	3.975×10^{-2}	1,290948
**LEAD vs CVD**				
	log2FoldChange	pvalue	padj	Fold change
UVSSA	-0,62524	$6{,}62 \times 10^{-6}$	2.430×10^{-3}	0,648312
MCM7	-0,37435	$2{,}99 \times 10^{-5}$	5.487×10^{-3}	0,771455
ACTR2	0,36705	$9{,}91 \times 10^{-5}$	7.278×10^{-3}	1,289713
POLM	-0,37014	$7{,}89 \times 10^{-5}$	7.278×10^{-3}	0,773707

RAD21	0,247039	$9,92 \times 10^{-5}$	7.278×10^{-3}	1,186769
PNKP	-0,33431	0,000353	1.780×10^{-2}	0,793161
PPP4R2	0,261183	0,000327	1.780×10^{-2}	1,198461
UBE2A	0,408651	0,000388	1.780×10^{-2}	1,327444
CLK2	-0,34388	0,000605	1.795×10^{-2}	0,787919
DEK	0,329127	0,000572	1.795×10^{-2}	1,256253
INIP	0,359037	0,000636	1.795×10^{-2}	1,28257
POLE	-0,31325	0,000538	1.795×10^{-2}	0,804826
XRCC6	0,309669	0,000469	1.795×10^{-2}	1,239423
ARID2	-0,24735	0,000714	1.820×10^{-2}	0,842444
UBE2V2	0,322271	0,000744	1.820×10^{-2}	1,250297
BCCIP	0,345511	0,000869	1.992×10^{-2}	1,270601
ATM	-0,34965	0,00103	2.223×10^{-2}	0,784773
SMC3	0,268109	0,001109	2.226×10^{-2}	1,204228
RFC1	0,242492	0,001174	2.226×10^{-2}	1,183035
RECQL	0,317271	0,001413	2.592×10^{-2}	1,245971
FZR1	-0,31497	0,001751	2.677×10^{-2}	0,803867
HMGB1	0,301849	0,001734	2.677×10^{-2}	1,232723
TOP1	0,285289	0,001637	2.677×10^{-2}	1,218654
ZMPSTE24	0,394098	0,001728	2.677×10^{-2}	1,314121
INO80B	-0,36855	0,002205	3.143×10^{-2}	0,774559
POLE4	0,375705	0,002227	3.143×10^{-2}	1,297473
SHPRH	-0,22479	0,003057	4.007×10^{-2}	0,855722
TERF2IP	0,300349	0,002957	4.007×10^{-2}	1,231442
SAMHD1	0,345299	0,003654	4.624×10^{-2}	1,270414
XRN2	0,342158	0,003789	4.624×10^{-2}	1,267652
**AAA vs CVD**				
	log2FoldChange	pvalue	padj	Fold change
LIG1	-0,45279	0,000204	4.451×10^{-2}	0,730626
POLE4	0,45659	0,000243	4.451×10^{-2}	1,372294
**POOLED**				
**LEAD, AAA and CVD vs Control**				
	log2FoldChange	pvalue	padj	Fold change
WHSC1	0,315813	$9,10 \times 10^{-6}$	2.248×10^{-3}	1,244713
NBN	-0,34477	$3,27 \times 10^{-5}$	3.682×10^{-3}	0,787433
UPF1	0,267259	$4,47 \times 10^{-5}$	3.682×10^{-3}	1,203519
POLE	0,313128	0,000488	3.016×10^{-2}	1,242398
NIPBL	-0,17931	0,000824	3.676×10^{-2}	0,883128
RNF138	-0,41811	0,000893	3.676×10^{-2}	0,748405
AP5Z1	0,278926	0,002277	4.434×10^{-2}	1,213292
CUL4A	0,192884	0,001759	4.434×10^{-2}	1,143046
EME2	0,363989	0,001974	4.434×10^{-2}	1,286979
HMGN1	-0,3149	0,002158	4.434×10^{-2}	0,803904
OGG1	0,301983	0,001649	4.434×10^{-2}	1,232838
RAD21	-0,23601	0,002451	4.434×10^{-2}	0,849091

TRRAP	0,221523	0,001908	4.434×10^{-2}	1,165964
UVSSA	0,44654	0,002514	4.434×10^{-2}	1,362768
JMY	-0,3125	0,003437	4.778×10^{-2}	0,805248
PNKP	0,289161	0,002922	4.778×10^{-2}	1,22193
POLG	0,176557	0,003528	4.778×10^{-2}	1,130183
SMARCAD1	-0,27313	0,003676	4.778×10^{-2}	0,827522
USP1	-0,25104	0,003375	4.778×10^{-2}	0,840292
**LEAD, AAA vs CVD**				
	log2FoldChange	pvalue	padj	Fold change
ATM	-0,33876	0,000268	2.116×10^{-2}	0,790719
INIP	0,357976	0,000108	2.116×10^{-2}	1,281626
LIG1	-0,38867	0,000215	2.116×10^{-2}	0,763834
POLE4	0,415508	0,000126	2.116×10^{-2}	1,333768
POLM	-0,314	0,000174	2.116×10^{-2}	0,804407
TREX2	-0,81435	0,000298	2.116×10^{-2}	0,568664
XRCC6	0,289186	0,000208	2.116×10^{-2}	1,221951

Table S3. Genes identified as indicative by UVE-PLS method. Genes were ordered alphabetically in each comparison.

No.	Gene	PLS coefficient	No.	Gene	PLS coefficient
**LEAD vs Control[a]**			**LEAD vs AAA**		
1	*ANKRD32*	0.139	1	*ASCC3*	0.065
2	*ATM[b]*	-0.220	2	*CLK2*	-0.106
3	*CUL4A*	0.089	3	*DEK*	0.113
4	*JMY*	-0.158	4	*ETAA1*	0.088
5	*SPIRE1*	0.125	5	*FANCB*	0.092
6	*USP51*	-0.155	6	*MCM5*	-0.099
7	*WHSC1*	0.121	7	*MCM7*	-0.107
**AAA vs Control**			8	*PNKP*	-0.090
1	*AP5S1*	0.056	9	*POLE3*	0.134
2	*APBB1*	-0.099	10	*PPP4R2*	0.123
3	*ASF1A*	-0.103	11	*RAD21*	0.105
4	*ATM*	-0.061	12	*RTEL1*	-0.092
5	*DEK*	-0.069	13	*SMARCAD1*	0.111
6	*EGFR*	-0.048	14	*SMC3*	0.120
7	*ERCC5*	-0.050	15	*TDG*	0.064
8	*ERCC6L2*	-0.040	16	*UBE2A*	0.143
9	*INTS3*	0.054	17	*UBE2V2*	0.046
10	*JMY*	-0.052	18	*UVSSA*	-0.189
11	*MORF4L2*	-0.056	**LEAD vs CVD**		
12	*NIPBL*	-0.039	1	*ACTR2*	0.067
13	*NPAS2*	-0.070	2	*ARID2*	-0.059
14	*NUCKS1*	-0.049	3	*ATM*	-0.070
15	*OGG1*	0.080	4	*BCCIP*	0.084
16	*PARP2*	-0.057	5	*C12orf5*	0.041
17	*PDS5A*	-0.048	6	*DEK*	0.056
18	*PDS5B*	-0.045	7	*DTX3L*	0.105
19	*PNKP*	0.082	8	*FZR1*	-0.053
20	*POLE*	0.069	9	*HERC2*	-0.036
21	*PPP4R2*	-0.048	10	*INIP*	0.067
22	*PRRX1*	-0.101	11	*MC1R*	-0.070
23	*RAD21*	-0.082	12	*MCM7*	-0.091
24	*RAD23BP3*	-0.003	13	*PARP9*	0.112
25	*RTEL1*	0.070	14	*PNKP*	-0.069
26	*SIRT7*	0.062	15	*POLE4*	0.067
27	*SLX4*	0.045	16	*POLM*	-0.086
28	*SMARCAD1*	-0.081	17	*PPP4R2*	0.077
29	*SMC3*	-0.053	18	*RAD21*	0.056
30	*SPIDR*	0.048	19	*RECQL*	0.058
31	*TEX264*	0.033	20	*RFC1*	0.060
32	*TOP3A*	0.067	21	*RFC2*	0.035

33	*UPF1*	0.076
34	*USP1*	-0.051
35	*USP10*	0.038
36	*USP51*	-0.054
37	*UVSSA*	0.125
38	*VCP*	0.050
39	*WHSC1*	0.080
40	*ZBTB1*	-0.058
**CVD vs Control**		
1	*ACTR2*	0.059
2	*AP5Z1*	-0.108
3	*ARID2*	-0.063
4	*BCCIP*	0.066
5	*DEK*	0.067
6	*EGFR*	0.041
7	*EME2*	-0.102
8	*FZR1*	-0.092
9	*GGN*	0.016
10	*GTF2I*	0.100
11	*HERC2*	-0.059
12	*HMGN1*	0.101
13	*HSPA1A*	0.138
14	*HUS1B*	-0.061
15	*HUWE1*	-0.045
16	*MORF4L2*	0.058
17	*NBN*	0.060
18	*NFRKB*	-0.096
19	*NIPBL*	0.038
20	*OGG1*	-0.072
21	*PARP2*	0.053
22	*PNKP*	-0.094
23	*POLE*	-0.101
24	*RAD17P1*	0.065
25	*RAD21*	0.073
26	*RAD51B*	-0.063
27	*SAMHD1*	0.040
28	*SMC2*	0.103
29	*SMC3*	0.085
30	*TDP2*	0.058
31	*TEX264*	-0.060
32	*TOP1*	0.038
33	*TREX2*	-0.057
34	*TRRAP*	-0.083
35	*UBR5*	-0.051
36	*UFL1*	0.042
22	*SAMHD1*	0.063
23	*SHPRH*	-0.039
24	*SMC3*	0.071
25	*TDG*	0.052
26	*TERF2IP*	0.089
27	*TOP1*	0.069
28	*TREX2*	-0.065
29	*TRRAP*	-0.035
30	*UBE2A*	0.068
31	*UBE2V2*	0.078
32	*USP51*	-0.047
33	*UVSSA*	-0.189
34	*XRCC6*	0.070
35	*ZMPSTE24*	0.057
**AAA vs CVD**		
1	*BRCC3*	0.068
2	*CHD1L*	-0.094
3	*ERCC6L2*	-0.060
4	*GLTSCR2*	-0.119
5	*HLTF*	-0.102
6	*INIP*	0.077
7	*LIG1*	-0.093
8	*MB21D1*	0.101
9	*MSH2*	-0.105
10	*PCNA*	0.075
11	*POLE4*	0.087
12	*RFC2*	0.065
13	*SIRT7*	0.072
14	*XRCC6*	0.069
**LEAD. AAA and CVD vs Control**		
1	*ATM*	-0.165
2	*HMGN1*	-0.118
3	*JMY*	-0.132
4	*MORF4L2*	-0.061
5	*NIPBL*	-0.059
6	*PARP2*	-0.079
7	*POLE*	0.091
8	*RAD21*	-0.049
9	*UPF1*	0.094
10	*USP1*	-0.047
11	*WHSC1*	0.127
**LEAD and AAA vs CVD**		
1	*ACTR2*	0.040
2	*ARID2*	-0.058
3	*ATM*	-0.111

37	*UPF1*	-0.083	4	*CHD1L*	-0.111
38	*USP1*	0.062	5	*DTX3L*	0.089
39	*UVSSA*	-0.157	6	*HLTF*	-0.148
40	*WHSC1*	-0.075	7	*INIP*	0.113
41	*XRCC6*	0.077	8	*LIG1*	-0.117
			9	*MB21D1*	0.120
			10	*PARP9*	0.104
			11	*PCNA*	0.058
			12	*POLE4*	0.120
			13	*POLM*	-0.100
			14	*RECQL*	0.048
			15	*RFC2*	0.084
			16	*SAMHD1*	0.048
			17	*TOP1*	0.079
			18	*TREX2*	-0.112
			19	*USP51*	-0.063
			20	*XRCC6*	0.081

AAA - abdominal aortic aneurysm, CVD - chronic venous disease, LEAD - lower extremities arterial disease, UVE-PLS - Uninformative Variable Elimination by Partial Least Squares. [a] genes from DESeq2 analysis; [b] ATM gene common for both DESeq2 and UVE-PLS analysis

Table S4. Results of ROC analysis. Genes were ordered alphabetically in each

comparison.

No.	Gene	Threshold	Specificity	Sensitivity	Accuracy	positive predictive value	negative predictive value	ROC-AUC[1]
**LEAD vs Control[a]**								
1	*ANKRD32*	7,056	1,000	1,000	1,000	1,000	1,000	1,000
2	*ATM*[b]	12,204	1,000	1,000	1,000	1,000	1,000	1,000
3	*CUL4A*	9,884	0,750	1,000	0,867	0,778	1,000	0,911
4	*JMY*	8,703	0,750	1,000	0,867	0,778	1,000	0,911
5	*SPIRE1*	6,202	0,750	1,000	0,867	0,778	1,000	0,893
6	*USP51*	3,814	0,875	1,000	0,933	0,875	1,000	0,964
7	*WHSC1*	9,427	1,000	0,857	0,933	1,000	0,889	0,964
**AAA vs Control**								
1	*AP5S1*	6,407	1,000	0,857	0,929	1,000	0,875	0,959
2	*APBB1*	8,270	0,714	1,000	0,857	0,778	1,000	0,878
3	*ASF1A*	6,690	0,857	1,000	0,929	0,875	1,000	0,959
4	*ATM*	12,213	0,857	1,000	0,929	0,875	1,000	0,918
5	*DEK*	9,070	0,857	1,000	0,929	0,875	1,000	0,980
6	*EGFR*	2,126	1,000	0,857	0,929	1,000	0,875	0,959
7	*ERCC5*	7,988	1,000	0,857	0,929	1,000	0,875	0,959
8	*ERCC6L2*	8,714	0,857	1,000	0,929	0,875	1,000	0,980
9	*INTS3*	10,009	0,714	1,000	0,857	0,778	1,000	0,918
10	*JMY*	8,741	0,857	1,000	0,929	0,875	1,000	0,918
11	*MORF4L2*	8,375	0,857	1,000	0,929	0,875	1,000	0,898
12	*NIPBL*	10,964	0,714	1,000	0,857	0,778	1,000	0,939
13	*NPAS2*	4,055	0,857	1,000	0,929	0,875	1,000	0,898
14	*OGG1*	8,410	1,000	1,000	1,000	1,000	1,000	1,000
15	*PARP2*	5,926	0,857	1,000	0,929	0,875	1,000	0,918
16	*PDS5A*	10,721	0,857	1,000	0,929	0,875	1,000	0,898
17	*PDS5B*	9,252	0,857	1,000	0,929	0,875	1,000	0,918
18	*PNKP*	8,965	1,000	0,857	0,929	1,000	0,875	0,959
19	*POLE*	9,520	1,000	0,857	0,929	1,000	0,875	0,959
20	*PPP4R2*	8,394	1,000	1,000	1,000	1,000	1,000	1,000
21	*PRRX1*	3,686	1,000	0,714	0,857	1,000	0,778	0,918
22	*RAD21*	10,093	1,000	1,000	1,000	1,000	1,000	1,000
23	*RTEL1*	4,636	1,000	0,857	0,929	1,000	0,875	0,939
24	*SIRT7*	9,327	1,000	0,714	0,857	1,000	0,778	0,918
25	*SMARCAD1*	7,728	1,000	0,857	0,929	1,000	0,875	0,959
26	*SMC3*	8,431	0,714	1,000	0,857	0,778	1,000	0,898
27	*SPIDR*	9,597	0,857	0,857	0,857	0,857	0,857	0,898
28	*TOP3A*	9,259	0,857	1,000	0,929	0,875	1,000	0,918
29	*UPF1*	10,408	1,000	1,000	1,000	1,000	1,000	1,000
30	*USP1*	8,148	1,000	1,000	1,000	1,000	1,000	1,000
31	*USP10*	9,479	0,857	1,000	0,929	0,875	1,000	0,918
32	*USP51*	3,811	0,857	1,000	0,929	0,875	1,000	0,959

33	*UVSSA*	9,145	0,857	1,000	0,929	0,875	1,000	0,980
34	*VCP*	10,957	0,714	1,000	0,857	0,778	1,000	0,898
35	*WHSC1*	9,466	1,000	1,000	1,000	1,000	1,000	1,000
36	*ZBTB1*	9,332	0,857	1,000	0,929	0,875	1,000	0,918
**CVD vs Control**								
1	*AP5Z1*	9,638	0,857	1,000	0,929	0,875	1,000	0,980
2	*ARID2*	10,022	0,857	1,000	0,929	0,875	1,000	0,980
3	*BCCIP*	7,310	1,000	0,857	0,929	1,000	0,875	0,939
4	*DEK*	9,071	0,857	1,000	0,929	0,875	1,000	0,918
5	*EGFR*	2,116	0,714	1,000	0,857	0,778	1,000	0,918
6	*EME2*	8,761	0,857	1,000	0,929	0,875	1,000	0,939
7	*FZR1*	9,297	0,857	1,000	0,929	0,875	1,000	0,980
8	*GTF2I*	9,180	0,857	1,000	0,929	0,875	1,000	0,980
9	*HERC2*	10,368	0,857	1,000	0,929	0,875	1,000	0,959
10	*HMGN1*	8,713	1,000	1,000	1,000	1,000	1,000	1,000
11	*HSPA1A*	8,610	1,000	0,714	0,857	1,000	0,778	0,898
12	*MORF4L2*	8,399	1,000	1,000	1,000	1,000	1,000	1,000
13	*NBN*	8,699	0,857	1,000	0,929	0,875	1,000	0,980
14	*NFRKB*	9,399	0,857	1,000	0,929	0,875	1,000	0,980
15	*NIPBL*	10,965	0,857	1,000	0,929	0,875	1,000	0,980
16	*OGG1*	8,405	0,857	1,000	0,929	0,875	1,000	0,898
17	*PARP2*	5,930	0,857	1,000	0,929	0,875	1,000	0,918
18	*PNKP*	9,027	0,857	1,000	0,929	0,875	1,000	0,980
19	*POLE*	9,577	1,000	1,000	1,000	1,000	1,000	1,000
20	*RAD17P1*	2,871	0,857	1,000	0,929	0,875	1,000	0,980
21	*RAD21*	10,138	1,000	1,000	1,000	1,000	1,000	1,000
22	*TDP2*	7,983	1,000	0,857	0,929	1,000	0,875	0,959
23	*TREX2*	3,920	0,857	1,000	0,929	0,875	1,000	0,959
24	*TRRAP*	11,609	1,000	1,000	1,000	1,000	1,000	1,000
25	*UPF1*	10,378	1,000	1,000	1,000	1,000	1,000	1,000
26	*USP1*	8,151	1,000	1,000	1,000	1,000	1,000	1,000
27	*UVSSA*	9,105	1,000	1,000	1,000	1,000	1,000	1,000
28	*WHSC1*	9,491	0,857	1,000	0,929	0,875	1,000	0,980
29	*XRCC6*	9,912	1,000	1,000	1,000	1,000	1,000	1,000
**LEAD vs AAA**								
1	*CLK2*	9,118	0,750	1,000	0,867	0,778	1,000	0,946
2	*DEK*	9,127	0,750	1,000	0,867	0,778	1,000	0,929
3	*FANCB*	4,698	0,750	1,000	0,867	0,778	1,000	0,929
4	*MCM5*	9,679	1,000	0,857	0,933	1,000	0,889	0,964
5	*MCM7*	9,155	1,000	0,714	0,867	1,000	0,800	0,911
6	*PNKP*	9,006	0,875	1,000	0,933	0,875	1,000	0,911
7	*POLE3*	8,195	1,000	0,857	0,933	1,000	0,889	0,946
8	*PPP4R2*	8,417	1,000	1,000	1,000	1,000	1,000	1,000
9	*RAD21*	10,051	1,000	1,000	1,000	1,000	1,000	1,000
10	*SMARCAD1*	7,711	0,875	1,000	0,933	0,875	1,000	0,964

11	*SMC3*	8,480	0,875	1,000	0,933	0,875	1,000	0,964
12	*UBE2A*	7,970	0,875	0,857	0,867	0,857	0,875	0,911
13	*UVSSA*	9,047	0,750	1,000	0,867	0,778	1,000	0,875
**LEAD vs CVD**								
1	*ACTR2*	11,332	0,875	1,000	0,933	0,875	1,000	0,946
2	*ARID2*	9,970	0,750	1,000	0,867	0,778	1,000	0,893
3	*ATM*	12,173	1,000	0,714	0,867	1,000	0,800	0,911
4	*BCCIP*	7,276	1,000	0,857	0,933	1,000	0,889	0,964
5	*DEK*	9,160	0,625	1,000	0,800	0,700	1,000	0,875
6	*FZR1*	9,292	0,750	0,857	0,800	0,750	0,857	0,857
7	*INIP*	7,382	0,875	1,000	0,933	0,875	1,000	0,929
8	*MCM7*	9,164	1,000	0,857	0,933	1,000	0,889	0,964
9	*PNKP*	9,012	0,875	1,000	0,933	0,875	1,000	0,946
10	*POLE4*	7,369	0,750	1,000	0,867	0,778	1,000	0,929
11	*POLM*	8,612	1,000	0,857	0,933	1,000	0,889	0,946
12	*PPP4R2*	8,427	1,000	0,857	0,933	1,000	0,889	0,911
13	*RAD21*	10,057	1,000	0,714	0,867	1,000	0,800	0,893
14	*RECQL*	8,744	0,750	0,857	0,800	0,750	0,857	0,857
15	*RFC1*	8,917	0,875	0,857	0,867	0,857	0,875	0,893
16	*SAMHD1*	12,385	0,750	1,000	0,867	0,778	1,000	0,893
17	*SHPRH*	9,285	1,000	0,857	0,933	1,000	0,889	0,911
18	*SMC3*	8,473	0,875	1,000	0,933	0,875	1,000	0,982
19	*TERF2IP*	8,986	0,750	1,000	0,867	0,778	1,000	0,946
20	*TOP1*	9,217	0,875	0,857	0,867	0,857	0,875	0,893
21	*UBE2A*	8,055	0,625	1,000	0,800	0,700	1,000	0,893
22	*UBE2V2*	6,996	1,000	1,000	1,000	1,000	1,000	1,000
23	*UVSSA*	9,068	0,750	1,000	0,867	0,778	1,000	0,893
24	*XRCC6*	9,859	1,000	0,714	0,867	1,000	0,800	0,911
25	*ZMPSTE24*	7,484	0,875	0,857	0,867	0,857	0,875	0,929
**AAA vs CVD**								
1	*LIG1*	8,459	0,714	1,000	0,857	0,778	1,000	0,878
2	*POLE4*	7,361	0,714	1,000	0,857	0,778	1,000	0,918
**LEAD. AAA and CVD vs Control**								
1	*HMGN1*	8,731	0,773	1,000	0,828	0,583	1,000	0,877
2	*JMY*	8,740	0,682	1,000	0,759	0,500	1,000	0,851
3	*NIPBL*	11,019	0,818	0,857	0,828	0,600	0,947	0,896
4	*POLE*	9,513	0,909	0,857	0,897	0,750	0,952	0,909
5	*RAD21*	10,137	0,773	1,000	0,828	0,583	1,000	0,857
6	*UPF1*	10,363	0,909	1,000	0,931	0,778	1,000	0,955
7	*USP1*	8,150	0,864	1,000	0,897	0,700	1,000	0,922
8	*WHSC1*	9,467	0,864	1,000	0,897	0,700	1,000	0,981
**LEAD and AAA vs CVD**								
1	*ATM*	12,062	0,600	1,000	0,727	0,538	1,000	0,867
2	*INIP*	7,382	0,733	1,000	0,818	0,636	1,000	0,914
3	*LIG1*	8,641	0,867	0,857	0,864	0,750	0,929	0,895

4	*POLE4*	7,361	0,733	1,000	0,818	0,636	1,000	0,924
5	*POLM*	8,626	0,933	0,857	0,909	0,857	0,933	0,905
6	*TREX2*	3,907	0,733	1,000	0,818	0,636	1,000	0,914
7	*XRCC6*	9,916	0,733	1,000	0,818	0,636	1,000	0,914

[1]Area under ROC curve, AAA abdominal aortic aneurysm, CVD - chronic venous disease, LEAD - lower extremities arterial disease. [a] genes from DESeq2 analysis; [b] ATM: gene common for both DESeq2 and UVE-PLS analysis

Table S5. Occurences of indicative genes in pairwise and pooled groups comparisons.

Genes common for at least two comparisons											
**Gene**	**Oc**	**L vs Cg**	**A vs Cg**	**C vs Cg**	**L vs A**	**L vs C**	**A vs C**	**LAC vs Cg**	**LA vs C**	**Related process or term according to analysed literature**	**Literature**
RAD21	**5**	–	**1**	**1**	**1**	**1**	–	**1**	–	cohesin subunit, DSB; **VEGFA regulation**	[1, 2, 3]
ATM	**4**	**1**	**1**	–	–	**1**	–	–	**1**	kinase: node of regulatory functions in DNA repair, DSB, mitosis, regulation of chromatin remodelling, oxidative stress response, a plethora of other metabolic processes regulation	[4, 5, 6, 7]
DEK	**4**	–	**1**	**1**	**1**	**1**	–	–	–	DNA secondary structure specific binding; **promotes muscle stem cell activation**	[8, 9]
PNKP	**4**	–	**1**	**1**	**1**	**1**	–	–	–	DSB, free DNA end processing, oxidative damage, mtDNA damage repair	[10, 11, 12]
UVSSA	**4**	–	**1**	**1**	**1**	**1**	–	–	–	TC NER, scaffold protein, RNApolII interaction	[13, 14, 15]
NIPBL	**3**	–	**1**	**1**	–	–	–	**1**	–	cohesin loading factor	[16, 17]
POLE	**3**	–	**1**	**1**	–	–	–	**1**	–	gynecologic cancers, sensing and processing of DSB, chromatin remodelling, AP sites, TLS polymerase	[18, 19, 20, 21, 22, 23, 24]
PPP4R2	**3**	–	**1**	–	**1**	**1**	–	–	–	dephosphorylation of critical DNA damage response proteins	[25]
SMC3	**3**	–	**1**	–	**1**	**1**	–	–	–	cohesin; chromosome metabolism	[26, 27, 28]
UPF1	**3**	–	**1**	**1**	–	–	–	**1**	–	DSB end resection, DNA/RNA helicase, telomere; nonsense-mediated mRNA decay	[29, 30]
USP1	**3**	–	**1**	**1**	–	–	–	**1**	–	ubiquitin deubiquitinase, ICL repair, HR, DSB	[31, 32, 33]
WHSC1 (NDS2)	**3**	–	**1**	**1**	–	–	–	**1**	–	histone methyltransferase, transcription activator, **congenital heart defects**	[34, 35]
XRCC6 (Ku60)	**3**	–	–	**1**	–	**1**	–	–	**1**	NHEJ, DSB, oxidative stress	[36, 37, 38]
POLE4	**3**	–	–	–	–	**1**	**1**	–	**1**	histone chaperone, chromatin integrity during DNA replication	[39]
EGFR	**2**	–	**1**	**1**	–	–	–	–	–	signal transduction, migration to the nucleus, regulation of DNA repair by both signal transduction and direct binding to DSB ends	[40, 41, 42]
JMY	**2**	–	**1**	–	–	–	–	**1**	–	regulating the p53 response, **cell motility** and invasion; *ACTR2* activation, actin regulation	[43, 44]
MORF4L2 (MRGX)	**2**	–	**1**	**1**	–	–	–	–	–	**unstable atherosclerotic plaque**; oxidative stress	[45, 46]
OGG1	**2**	–	**1**	**1**	–	–	–	–	–	repair 8-oxoG in nuclear and mtDNA, BER, is also transcriptional activator, prevents **inflammation**	[47, 48, 49, 50, 51]
PARP2	**2**	–	**1**	**1**	–	–	–	–	–	BER, DSB, protein activity regulation through ADP-ribosylation	[52, 53, 54, 55]
SMARCAD1	**2**	–	**1**	–	**1**	–	–	–	–	chromatin remodeller; MMR, DSB HR	[56, 57]
ARID2 (Baf200)	**2**	–	–	**1**	–	**1**	–	–	–	chromatin remodelling, DSB, HR; NER	[58, 59]

BCCIP	**2**	–	–	**1**	–	**1**	–	–	–	general response to DNA damage; HR	[60, 61]
FZR1 (CDH1)	**2**	–	–	**1**	–	**1**	–	–	–	cell cycle regulation, G2 DNA-damage-response checkpoint; **atherosclerosis-derived monocytes**	[62, 63]
HMGN1	**2**	–	–	**1**	–	–	–	**1**	–	chromatin remodelling; transcription coupled repair, global genome repair, NER	[64, 65, 66]
TREX2	**2**	–	–	**1**	–	–	–	–	**1**	3′ exonuclease, psoriasis, **inflammation**, skin remodelling	[67, 68, 69]
MCM7	**2**	–	–	–	**1**	**1**	–	–	–	helicase, replication forks; Critical Regulator of the S-Phase Checkpoint	[70]
UBE2A	**2**	–	–	–	**1**	**1**	–	–	–	ubiquitin, potential deamidation regulator, proteins deamidation	[71, 72, 73,]
INIP (SSBIP1)	**2**	–	–	–	–	**1**	–	–	**1**	DSB repair, ssDNA binding	[74, 75,]
POLM	**2**	–	–	–	–	**1**	–	–	**1**	DSB, NHEJ	[76, 77]
LIG1	**2**	–	–	–	–	–	**1**	–	**1**	main replication ligase, ligation of DNA in DNA repair and HR	[78, 79]
**Genes unique for only one comparison**											
Gene	**Oc.**	**L vs Cg**	**A vs Cg**	**C vs Cg**	**L vs A**	**L vs C**	**A vs C**	**LAC vs Cg**	**LA vs C**	**Related process or term**	**Literature**
AP5S1	**1**	–	**1**	–	–	–	–	–	–	DSB, HR; endosomal sorting	[80, 81]
APBB1 (FE65)	**1**	–	**1**	–	–	–	–	–	–	histone remodelling, modulates DSB	[82, 83]
ASF1A	**1**	–	**1**	–	–	–	–	–	–	Histone remodelling; DSB, NHEJ; nucleosome assembly	[84, 85, 86]
ERCC5 (XPG)	**1**	–	**1**	–	–	–	–	–	–	Endonuclease, oxidative DNA damage, multiple non-enzymatic functions in DNA repair	[87, 88, 89]
ERCC6L2	**1**	–	**1**	–	–	–	–	–	–	chromatin remodelling, NHEJ, DNA ligation	[90]
INTS3	**1**	–	**1**	–	–	–	–	–	–	DSB repair, ssDNA binding, scaffold protein	[91, 92]
NPAS2 (MOP4)	**1**	–	**1**	–	–	–	–	–	–	circadian oscillator; **corelated with cardiovascular risk**	[93, 94]
PDS5A	**1**	–	**1**	–	–	–	–	–	–	cohesin interactors, cohesin turnover, HR, replication fork integrity	[95, 96]
PDS5B	**1**	–	**1**	–	–	–	–	–	–	cohesin interactors, cohesin turnover, HR, replication fork integrity	[95, 96]
PRRX1	**1**	–	**1**	–	–	–	–	–	–	transcription factor, involved in DNA damage repair, gene regulation; **vasculogenic processes**	[97, 98, 99, 100, 101]
RTEL1	**1**	–	**1**	–	–	–	–	–	–	helicase, HR; telomere maintenance; **increased in coronary microvascular remodeling**	[102, 103, 104]
SIRT7	**1**	–	**1**	–	–	–	–	–	–	histone remodelling; regulates DSB; **neointimal formation-regulation of the VSMC proliferation and motility**	[105, 106, 107, 108]
SPIDR	**1**	–	**1**	–	–	–	–	–	–	DSB, HR, scaffold protein	[109, 110, 111]
TOP3A	**1**	–	**1**	–	–	–	–	–	–	topoisomerase, DNA structures resoultion, HJ resolution, HR	[112, 113, 114]
USP10	**1**	–	**1**	–	–	–	–	–	–	deubiquitinase, **foam cell formation**; MMR regulation	[115, 116]

USP51	**1**	–	**1**	–	–	–	–	–	–	deubiquitinase, NHEJ HR DSB; histone remodeller	[117, 118]
VCP (p97)	**1**	–	**1**	–	–	–	–	–	–	**angiotensin II signal transduction in VSMCs; Angiotensin II-induced VSMC senescence by promoting** ROS generation, DSB, TOP2-DNA crosslinks, 5′ DNA ends resection	[119, 120, 121, 122]
ZBTB1	**1**	–	**1**	–	–	–	–	–	–	PCNA mono ubiquitination, TLS activation, transcriptional repression	[123]
AP5Z1	**1**	–	–	**1**	–	–	–	–	–	subunit of AP5 complex, dysfunctional mitochondria, spastic paraplegia	[124]
ACTR2 (ARP2)	**1**	–	–	–	–	**1**	–	–	–	nuclear actin regulation, actin-dependent chromatin remodeling, direct role for actin and its partners in DNA repair; **migration of VSMCs neointima formation after vascular injury**	[125, 126, 127]
CLK2	**1**	–	–	–	**1**	–	–	–	–	S-phase checkpoint, mRNA splicing, **modulation of blood coagulation**	[128, 129, 130, 131]
EME2	**1**	–	–	**1**	–	–	–	–	–	regulation of structure-specific endonuclease MUS81 complex, HJ resolvase completes HR process	[132, 133]
GTF2I (TFII-I)	**1**	–	–	**1**	–	–	–	–	–	general DNA damage response, transcriptionally suppresses the DNA damage repair pathways, promotes TLS	[134, 135]
HERC2	**1**	–	–	**1**	–	–	–	–	–	ubiquitin ligase, control of NER in circadian oscillator- manner, DSB	[136, 137, 138]
HSPA1A	**1**	–	–	**1**	–	–	–	–	–	heat schock protein, BER and NER pathways; **anti-inflammatory and antiapoptotic functions**	[139, 140]
NBN (NBS1)	**1**	–	–	**1**	–	–	–	–	–	chromosome break sensor	[141, 142]
NFRKB	**1**	–	–	**1**	–	–	–	–	–	nucleosome remodelling, chromatin remodelling	[143, 144]
RAD17P1	**1**	–	–	**1**	–	–	–	–	–	pseudogene of RAD17, possible regulation of RAD17, thus regulation of RAD17 functions like HR, histone remodeller, ATM activation and DNA end resection, AP sites, TLS	[145, 146]
TDP2	**1**	–	–	**1**	–	–	–	–	–	DNA-protein crosslinks repair	[147, 148]
TRRAP	**1**	–	–	**1**	–	–	–	–	–	scaffold protein in chromatin remodelling complexes, **trigliceride metabolism, APOC3 regulation, hipertriglicerydemia, atherosclerosis**	[149, 150, 151]
FANCB	**1**	–	–	–	**1**	–	–	–	–	DNA interstrand crosslinks, HR	[152, 153]
MCM5	**1**	–	–	–	**1**	–	–	–	–	helicase, replication forks	[70, 71]
POLE3	**1**	–	–	–	**1**	–	–	–	–	histone chaperone, chromatin integrity during DNA Replication	[39]
RECQL	**1**	–	–	–	–	**1**	–	–	–	helicase, DSB; regulation of gene expression	[154, 155,]
RFC1	**1**	–	–	–	–	**1**	–	–	–	PCNA loader/unloader	[156, 157]
SAMHD1	**1**	–	–	–	–	**1**	–	–	–	nucleotide pool regulation, dNTPase activity, DSB; **cerebral large-artery atherosclerosis**	[158, 159, 160, 161]
SHPRH	**1**	–	–	–	–	**1**	–	–	–	ubiquitin ligase, helicase; DNA	[162, 163, 164, 165]

										damage tolerance, nucleosome recognition; rDNA stability	
TERF2IP (RAP1)	**1**	–	–	–	–	**1**	–	–	–	telomere maintenance; **<u>epithelial cells activation and senescence</u>**	[166, 167]
TOP1	**1**	–	–	–	–	**1**	–	–	–	DNA replication, transcription, and recombination, DNA torsional stress management, TFIID repression	[168, 169, 170]
UBE2V2 (MMS2)	**1**	–	–	–	–	**1**	–	–	–	post replication repair, ubiquitin, PCNA polyubiquitination	[171]
ZMPSTE24 (STE24)	**1**	–	–	–	–	**1**	–	–	–	nuclear cytoskeleton, lamin A maturation	[172]

ABBREVIATIONS USED IN TABLE: **A** – abdominal aortic aneurysm; **C** – chronic venous disease; **L** – lower extremities arterial disease; –: gene absent in comparison; **Oc** – Occurence of gene in comparisons given; **L vs Cg** – LEAD vs Control group; **A vs Cg**– AAA vs Control group; **C vs Cg** – CVD vs Control group; **L vs A** – LEAD vs AAA; **L vs C** – LEAD vs CVD; **A vs C** – AAA vs CVD; **LAC vs Cg** – LEAD, AAA and CVD vs Control group; **LA vs C** – LEAD and AAA vs CVD. Decrease and increase in expression of a gene in comparison are denoted by white and grey color of field in the tabel respectively.

OTHER ABBREVIATIONS USED IN TABLE: **8 –oxoG** – 8 –oxo –7,8 –dihydroguanine; **ACTR2** – actin related protein 2; **AP sites** Apurynic/Apyrymidinic sites; **APOC3** – Apolipoprotein C3 **ATM** – Ataxia Telangiectasia Mutated; **BER** – Base Excision Repair; **DSB** – Double Strand Breaks; **HJ** – Holliday Junctions; **HR** – Homologous Recombination; **ICL** – Intrestrand Crosslinks; **MMR** – Mismatch Repair; **mtDNA** – mitochondrial DNA; **MUS81** – MUS81 Structure –Specific Endonuclease Subunit; **NER** – Nucleotide Excision Repair; **NHEJ** – Non – Homologous End Joining; **PCNA** –Proliferating Cells Nuclear Antigen; **RAD17** – RAD17 checkpoint clamp loader component; **rDNA** – ribosomal DNA; **RNApolII** – RNA Polymerase II; **ROS** – Reactive Oxygen Species; **ssDNA** – single stranded DNA; **TC NER** – Transcription Coupled Nucleotide Excision Repair; **TFIID** – Transcription Factor II D; **TLS** – Translesion Synthesis; **TOP2** – Topoisomerase 2; **VEGFA** – Vascular Endothelial Growth Factor A; **VSMC** – Vascular Smooth Muscle Cells; **<u>Terms related to atherosclerosis and cardiovascular system conditions, ascribed to given DNA repair gene, according to literature cited</u>**.

1. Cheng, H.; Zhang, N.; Pati, D. Cohesin subunit RAD21: From biology to disease. *Gene* **2020**, *758*, 144966. https://doi.org/10.1016/j.gene.2020.144966
2. Matsumoto, T.; Mugishima, H. Signal transduction via vascular endothelial growth factor (VEGF) receptors and their roles in atherogenesis. *J. Atheroscler. Thromb.* **2006**, *13*(3), 130–135. https://doi.org/10.5551/jat.13.130
3. Li, D.; Yang, T.; Shao, C.; Cao, Z.; Zhang, H. LncRNA MIAT activates vascular endothelial growth factor A through RAD21 to promote nerve injury repair in acute spinal cord injury. *Mol. Cell. Endocrinol.* **2021**, *528*, 111244. https://doi.org/10.1016/j.mce.2021.111244
4. Boohaker, R. J.; Xu, B. The versatile functions of ATM kinase. *Biomed J.* **2014**, *37*(1), 3–9. https://doi.org/10.4103/2319 –4170.125655
5. Berger, N.D.; Stanley, F.; Moore, S.; Goodarzi, A.A. ATM –dependent pathways of chromatin remodelling and oxidative DNA damage responses. *Philos. Trans. R. Soc. Lond. B Biol. Sci.* **2017**, *372*(1731), 20160283. https://doi.org/10.1098/rstb.2016.0283
6. Turan, V.; Oktay, K. BRCA –related ATM –mediated DNA double –strand break repair and ovarian aging. *Hum Reprod Update.* **2020**, *26*(1), 43–57. https://doi.org/10.1093/humupd/dmz043
7. Li, Z.; Pearlman, A.H.; Hsieh, P. DNA mismatch repair and the DNA damage response. *DNA Repair (Amst).* **2016**, *38*, 94–101. https://doi.org/10.1016/j.dnarep.2015.11.019
8. Brázda, V.; Laister, R.C.; Jagelská, E.B.; Arrowsmith, C. Cruciform structures are a common DNA feature important for regulating biological processes. *BMC Mol Biol.* **2011**, *12*, 33. https://doi.org/10.1186/1471 –2199 –12 –33

9. Yue, L.; Wan, R.; Luan, S.; Zeng, W.; Cheung, T.H. Dek Modulates Global Intron Retention during Muscle Stem Cells Quiescence Exit. *Dev. Cell* **2020**, *53*(6), 661–676.e6. https://doi.org/10.1016/j.devcel.2020.05.006
10. Shen, J.; Gilmore, E.C.; Marshall, C.A.; Haddadin, M.; Reynolds, J.J.; Eyaid, W.; Bodell, A.; Barry, B.; Gleason, D.; Allen, K.; Ganesh, V.S.; Chang, B.S.; Grix, A.; Hill, R.S.; Topcu, M.; Caldecott, K.W.; Barkovich, A.J.; & Walsh, C.A. Mutations in PNKP cause microcephaly, seizures and defects in DNA repair. *Nat. Genet.* **2010**, *42*(3), 245–249. https://doi.org/10.1038/ng.526
11. Jilani, A.; Ramotar, D.; Slack, C.; Ong, C.; Yang, X.M.; Scherer, S.W.; Lasko, D.D. Molecular cloning of the human gene, PNKP, encoding a polynucleotide kinase 3' –phosphatase and evidence for its role in repair of DNA strand breaks caused by oxidative damage. *J. Biol Chem.* **1999**, *274*(34), 24176–24186. https://doi.org/10.1074/jbc.274.34.2417
12. Aceytuno, R.D.; Piett, C.G.; Havali –Shahriari, Z., Edwards, R.A.; Rey, M.; Ye, R.; Javed, F.; Fang, S.; Mani, R.; Weinfeld, M.; Hammel, M.; Tainer, J.A.; Schriemer, D.C.; Lees –Miller, S.P.; Glover, J. Structural and functional characterization of the PNKP –XRCC4 –LigIV DNA repair complex. *Nucleic Acids Res.* **2017**, *45*(10), 6238–6251. https://doi.org/10.1093/nar/gkx275
13. Sarasin A. UVSSA and USP7: new players regulating transcription –coupled nucleotide excision repair in human cells. *Genome Med.* **2012**, *4*(5), 44. https://doi.org/10.1186/gm343
14. Schwertman, P.; Vermeulen, W.; & Marteijn, J.A. UVSSA and USP7, a new couple in transcription –coupled DNA repair. *Chromosoma* **2013**, *122*(4), 275–284. https://doi.org/10.1007/s00412 –013 –0420 –2
15. Nakazawa, Y.; Hara, Y.; Oka, Y.; Komine, O.; van den Heuvel, D.; Guo, C.; Daigaku, Y.; Isono, M.; He, Y.; Shimada, M.; Kato, K.; Jia, N.; Hashimoto, S.; Kotani, Y.; Miyoshi, Y.; Tanaka, M.; Sobue, A.; Mitsutake, N.; Suganami, T.; Masuda, A.; Ohno, K.; Nakada, S.; Mashimo, T.; Yamanaka, K.; Luijsterburg, M.S.; Ogi, T. Ubiquitination of DNA Damage –Stalled RNAPII Promotes Transcription –Coupled Repair. *Cell* **2020**, *180*(6), 1228–1244.e24. https://doi.org/10.1016/j.cell.2020.02.010
16. Garcia, P.; Fernandez –Hernandez, R.; Cuadrado, A.; Coca, I.; Gomez, A.; Maqueda, M.; Latorre –Pellicer, A.; Puisac, B.; Ramos, F.J.; Sandoval, J.; Esteller, M.; Mosquera, J.L.; Rodriguez, J.; Pié, J.; Losada, A.; Queralt, E. Disruption of NIPBL/Scc2 in Cornelia de Lange Syndrome provokes cohesin genome –wide redistribution with an impact in the transcriptome. *Nat. Commun.* **2021**, *12*(1), 4551. https://doi.org/10.1038/s41467 –021 –24808 –z
17. Schierding, W.; Horsfield, J.A.; & O'Sullivan, J.M. Low tolerance for transcriptional variation at cohesin genes is accompanied by functional links to disease –relevant pathways. *J. Med. Genet.* **2021**, *58*(8), 534–542. https://doi.org/10.1136/jmedgenet –2020 –107095
18. Conlon, N.; Da Cruz Paula, A.; Ashley, C.W.; Segura, S.; De Brot, L.; da Silva, E.M.; Soslow, R.A.; Weigelt, B.; & DeLair, D.F. Endometrial Carcinomas with a "Serous" Component in Young Women Are Enriched for DNA Mismatch Repair Deficiency,

Lynch Syndrome, and POLE Exonuclease Domain Mutations. *Am J Surg Pathol* . **2020**, *44*(5), 641–648. https://doi.org/10.1097/PAS.0000000000001461
19. Gotoh, O.; Sugiyama, Y.; Takazawa, Y.; Kato, K.; Tanaka, N.; Omatsu, K.; Takeshima, N.; Nomura, H.; Hasegawa, K.; Fujiwara, K.; Taki, M.; Matsumura, N.; Noda, T.; Mori, S. Clinically relevant molecular subtypes and genomic alteration –independent differentiation in gynecologic carcinosarcoma. *Nat. Commun.***2019**, *10*(1), 4965. https://doi.org/10.1038/s41467 –019 –12985 –x
20. Hollis, R.L.; Thomson, J.P.; Stanley, B.; Churchman, M.; Meynert, A.M.; Rye, T.; Bartos, C.; Iida, Y.; Croy, I.; Mackean, M.; Nussey, F.; Okamoto, A.; Semple, C.A.; Gourley, C.; Herrington, C.S. Molecular stratification of endometrioid ovarian carcinoma predicts clinical outcome. *Nat. Commun.***2020**, *11*(1), 4995. https://doi.org/10.1038/s41467 –020 –18819 –5
21. Davila, J.I.; Chanana, P.; Sarangi, V.; Fogarty, Z.C.; Weroha, S.J.; Guo, R.; Goode, E.L.; Huang, Y.; Wang, C. Frequent POLE –driven hypermutation in ovarian endometrioid cancer revealed by mutational signatures in RNA sequencing. *BMC Med. Genomics.* **2021**, *14*(1), 165. https://doi.org/10.1186/s12920 –021 –01017 –7
22. Casari, E.; Gobbini, E.; Gnugnoli, M.; Mangiagalli, M.; Clerici, M.; Longhese, M.P. Dpb4 promotes resection of DNA double –strand breaks and checkpoint activation by acting in two different protein complexes. *Nat. Commun.***2021**, *12*(1), 4750. https://doi.org/10.1038/s41467 –021 –25090 –9
23. Pursell, Z.F.; Isoz, I.; Lundström, E.B.; Johansson, E., Kunkel, T. A. Yeast DNA polymerase epsilon participates in leading –strand DNA replication. *Science* **2007**, *317*(5834), 127–130. https://doi.org/10.1126/science.1144067
24. Sabouri, N.; Johansson, E. Translesion synthesis of abasic sites by yeast DNA polymerase epsilon. *J. Biol. Chem.***2009**, *284*(46), 31555–31563. https://doi.org/10.1074/jbc.M109.043927
25. Herzig, J.K.; Bullinger, L.; Tasdogan, A.; Zimmermann, P.; Schlegel, M.; Teleanu, V.; Weber, D.; Rücker, F.G.; Paschka, P.; Dolnik, A.; Schneider, E.; Kuchenbauer, F.; Heidel, F.H.; Buske, C.; Döhner, H.; Döhner, K.; Gaidzik, V.I. Protein phosphatase 4 regulatory subunit 2 *(PPP4R2)* is recurrently deleted in acute myeloid leukemia and required for efficient DNA double strand break repair. *Oncotarget* **2017**, *8*(56), 95038–95053. https://doi.org/10.18632/oncotarget.21119
26. Xiang, S.; Koshland, D. Cohesin architecture and clustering in vivo. *eLife* **2021**, *10*, e62243. https://doi.org/10.7554/eLife.62243
27. Sarogni, P.; Pallotta, M.M.; Musio, A. Cornelia de Lange syndrome: from molecular diagnosis to therapeutic approach. *J. Med. Genet.***2020**, *57*(5), 289–295. https://doi.org/10.1136/jmedgenet –2019 –106277
28. Rahman, S.; Jones, M.J.; Jallepalli, P.V. Cohesin recruits the Esco1 acetyltransferase genome wide to repress transcription and promote cohesion in somatic cells. *Proc. Natl. Acad. Sci U. S. A.* **2015**, *112*(36), 11270–11275. https://doi.org/10.1073/pnas.1505323112

29. Ngo, G.; Grimstead, J.W.; Baird, D.M. UPF1 promotes the formation of R loops to stimulate DNA double –strand break repair. *Nat. Commun.***2021,** *12*(1), 3849. https://doi.org/10.1038/s41467 –021 –24201 –w
30. Azzalin C. M. UPF1: a leader at the end of chromosomes. *Nucleus* **2012**, *3*(1), 16–21. https://doi.org/10.4161/nucl.18929
31. Kim, M.; Kim, J.M. The role of USP1 autocleavage in DNA interstrand crosslink repair. *FEBS Lett.***2016**, *590*(3), 340–348. https://doi.org/10.1002/1873 –3468.12060
32. Lim, K.S.; Li, H., Roberts, E.A.; Gaudiano, E.F.; Clairmont, C.; Sambel, L.A.; Ponnienselvan, K.; Liu, J.C.; Yang, C.; Kozono, D.; Parmar, K.; Yusufzai, T.; Zheng, N.; D'Andrea, A.D. USP1 Is Required for Replication Fork Protection in BRCA1 –Deficient Tumors. *Mol. Cell* **2018**, *72*(6), 925–941.e4. https://doi.org/10.1016/j.molcel.2018.10.045
33. Cukras, S.; Lee, E.; Palumbo, E.; Benavidez, P.; Moldovan, G.L.; Kee, Y. The USP1 –UAF1 complex interacts with RAD51AP1 to promote homologous recombination repair. *Cell Cycle* **2016**, *15*(19), 2636–2646. https://doi.org/10.1080/15384101.2016.1209613
34. Audain, E.; Wilsdon, A.; Breckpot, J.; Izarzugaza, J.; Fitzgerald, T. W.; Kahlert, A.K.; Sifrim, A.; Wünnemann, F.; Perez –Riverol, Y.; Abdul –Khaliq, H.; Bak, M.; Bassett, A.S.; Benson, W.D.; Berger, F.; Daehnert, I.; Devriendt, K.; Dittrich, S.; Daubeney, P.E.; Garg, V.; Hackmann, K.; Hoff, K.; Hofmann, P.; Dombrowsky, G.; Pickardt, T.; Bauer, U.; Keavney, B.D.; Klaassen, S.; Kramer, H.H.; Marshall, C.R.; Milewicz, D.M.; Lemaire, S.; Coselli, J.S.; Mitchell, M.E.; Tomita –Mitchell, A.; Prakash, S.K.; Stamm, K.; Stewart, A.F.R.; Silversides, C.K.; Siebert, R.; Stiller, B.; Rosenfeld, J.A.; Vater, I.; Postma, A.V.; Caliebe, A.; Brook, J.D.; Andelfinger, G.; Hurles, M.E.; Thienpont, B.; Larsen, L.A.; Hitz, M. P. Integrative analysis of genomic variants reveals new associations of candidate haploinsufficient genes with congenital heart disease. *PLoS Genet.* **2021**, *17*(7), e1009679. https://doi.org/10.1371/journal.pgen.1009679
35. Wang, Y.; Zhu, L.; Guo, M.; Sun, G.; Zhou, K.; Pang, W.; Cao, D.; Tang, X.; Meng, X. Histone methyltransferase WHSC1 inhibits colorectal cancer cell apoptosis via targeting anti –apoptotic BCL2. *Cell Death Discov.* **2021**, *7*(1), 19. https://doi.org/10.1038/s41420 –021 –00402 –6
36. Kragelund, B.B.; Weterings, E.; Hartmann –Petersen, R.; Keijzers, G. The Ku70/80 ring in Non –Homologous End –Joining: easy to slip on, hard to remove. *Front. Biosci. (Landmark Ed.)* **2016**, *21*, 514–527. https://doi.org/10.2741/4406
37. Zhu, B.; Cheng, D.; Li, S.; Zhou, S.; Yang, Q. High Expression of XRCC6 Promotes Human Osteosarcoma Cell Proliferation through the β –Catenin/Wnt Signaling Pathway and Is Associated with Poor Prognosis. *Int. J. Mol. Sci.* **2016**, *17*(7), 1188. https://doi.org/10.3390/ijms17071188
38. Spagnolo, L.; Rivera –Calzada, A.; Pearl, L.H.; Llorca, O. Three –dimensional structure of the human DNA –PKcs/Ku70/Ku80 complex assembled on DNA and its implications for DNA DSB repair. *Mol. Cell* **2006**, *22*(4), 511–519. https://doi.org/10.1016/j.molcel.2006.04.013

39. Bellelli, R.; Belan, O.; Pye, V.E.; Clement, C.; Maslen, S.L.; Skehel, J.M.; Cherepanov, P.; Almouzni, G.; Boulton, S.J. POLE3 –POLE4 Is a Histone H3 –H4 Chaperone that Maintains Chromatin Integrity during DNA Replication. *Mol. Cell* **2018**, *72*(1), 112–126.e5. https://doi.org/10.1016/j.molcel.2018.08.043
40. Mak, V. C.; Li, X.; Rao, L.; Zhou, Y.; Tsao, S.W.; Cheung, L.W. p85β alters response to EGFR inhibitor in ovarian cancer through p38 MAPK –mediated regulation of DNA repair. *Neoplasia* **2021**, *23*(7), 718–730. https://doi.org/10.1016/j.neo.2021.05.009
41. Fan, X.J.; Wang, Y.L.; Zhao, W.W.; Bai, S.M.; Ma,Y.; Yin, X.K.; Feng, L.L.; Feng, W.X.; Wang, Y.N.; Liu, Q.; Hung, M.C.; Wan, X.B. NONO phase separation enhances DNA damage repair by accelerating nuclear EGFR –induced DNA –PK activation. *Am. J. Cancer Res.***2021**, 11(6) 2838–2852.
42. Liccardi, G.; Hartley, J.A.; Hochhauser, D. EGFR nuclear translocation modulates DNA repair following cisplatin and ionizing radiation treatment. *Cancer Res.* **2011**, *71*(3), 1103–1114. https://doi.org/10.1158/0008 –5472.CAN –10 –2384
43. Wang, Y. JimMY on the stage: Linking DNA damage with cell adhesion and motility. *Cell Adhes. Migr.* **2010**, *4*(2), 166–168. https://doi.org/10.4161/cam.4.2.11368
44. Coutts, A.S.; Weston, L.; La Thangue, N.B. Actin nucleation by a transcription co –factor that links cytoskeletal events with the p53 response. *Cell cycle* **2010**, *9*(8), 1511–1515. https://doi.org/10.4161/cc.9.8.11258
45. Zhang, R.; Ji, Z.; Yao, Y.; Zuo, W.; Yang, M.; Qu, Y.; Su, Y.; Ma, G.; Li, Y. Identification of hub genes in unstable atherosclerotic plaque by conjoint analysis of bioinformatics**.** *Life Sci.***2020**, *262*, 118517. https://doi.org/10.1016/j.lfs.2020.118517
46. Bomfim, M.M.; Andrade, G.M.; Del Collado, M.; Sangalli, J.R.; Fontes, P.K.; Nogueira, M.; Meirelles, F. V.; da Silveira, J.C.; Perecin, F. Antioxidant responses and deregulation of epigenetic writers and erasers link oxidative stress and DNA methylation in bovine blastocysts. *Mol.Reprod. Dev.* **2017**, *84*(12), 1296–1305. https://doi.org/10.1002/mrd.22929
47. Aguilera –Aguirre, L.; Hosoki, K.; Bacsi, A.; Radák, Z.; Wood, T.G.; Widen, S.G.; Sur, S.; Ameredes, B.T.; Saavedra –Molina, A.; Brasier, A.R.; Ba, X.; Boldogh, I. Whole transcriptome analysis reveals an 8 –oxoguanine DNA glycosylase –1 –driven DNA repair –dependent gene expression linked to essential biological processes. *Free Radic. Biol. Med.* **2015**, *81*, 107–118. https://doi.org/10.1016/j.freeradbiomed.2015.01.004
48. Yuzefovych, L.V.; Kahn, A.G.; Schuler, M.A.; Eide, L.; Arora, R.; Wilson, G.L.; Tan, M.; Rachek, L.I. Mitochondrial DNA Repair through OGG1 Activity Attenuates Breast Cancer Progression and Metastasis. *Cancer Res.* **2016**, *76*(1), 30–34. https://doi.org/10.1158/0008 –5472.CAN –15 –0692
49. Brasier, A.R.; Boldogh, I. Targeting inducible epigenetic reprogramming pathways in chronic airway remodeling. *Drugs Context.* **2019**, *8*, 2019 –8 –3. https://doi.org/10.7573/dic.2019 –8 –3
50. Hao, W.; Wang, J.; Zhang, Y.; Wang, C.; Xia, L.; Zhang, W.; Zafar, M.; Kang, J.Y.; Wang, R.; Ali Bohio, A.; Pan, L.; Zeng, X.; Wei, M.; Boldogh, I.; Ba, X. Enzymatically inactive OGG1 binds to DNA and steers base excision repair toward gene transcription. *FASEB J.***2020**, *34*(6), 7427–7441. https://doi.org/10.1096/fj.201902243R

51. Tumurkhuu, G.; Shimada, K.; Dagvadorj, J.; Crother, T.R.; Zhang, W.; Luthringer, D.; Gottlieb, R.A.; Chen, S.; Arditi, M. Ogg1 –Dependent DNA Repair Regulates NLRP3 Inflammasome and Prevents Atherosclerosis. *Circ. Res.***2016**, *119*(6), e76–e90. https://doi.org/10.1161/CIRCRESAHA.116.308362
52. Bilokapic, S.; Suskiewicz, M.J.; Ahel, I.; Halic, M. Bridging of DNA breaks activates PARP2 –HPF1 to modify chromatin. *Nature* **2020**, *585*(7826), 609–613. https://doi.org/10.1038/s41586 –020 –2725 –7
53. Schreiber, V.; Amé, J.C.; Dollé, P.; Schultz, I.; Rinaldi, B.; Fraulob, V.; Ménissier –de Murcia, J.; de Murcia, G. Poly(ADP –ribose) polymerase –2 (PARP –2) is required for efficient base excision DNA repair in association with PARP –1 and XRCC1. *J. Biol. Chem.***2002**, *277*(25), 23028–23036. https://doi.org/10.1074/jbc.M202390200
54. Suskiewicz, M.J.; Zobel, F.; Ogden, T.; Fontana, P.; Ariza, A.; Yang, J.C.; Zhu, K.; Bracken, L.; Hawthorne, W.J.; Ahel, D.; Neuhaus, D.; Ahel, I. HPF1 completes the PARP active site for DNA damage –induced ADP –ribosylation. *Nature* **2020**, *579*(7800), 598–602. https://doi.org/10.1038/s41586 –020 –2013 –6
55. Fouquin, A.; Guirouilh –Barbat, J.; Lopez, B.; Hall, J.; Amor –Guéret, M.; Pennaneach, V. PARP2 controls double –strand break repair pathway choice by limiting 53BP1 accumulation at DNA damage sites and promoting end –resection. *Nucleic Acids Res.* **2017**, 45(21), 12325–12339. https://doi.org/10.1093/nar/gkx881
56. Tong, Z.B.; Ai, H.S.; Li, J. B. The Mechanism of Chromatin Remodeler SMARCAD1/Fun30 in Response to DNA Damage. *Front. Cell. Dev. Biol.* **2020**, *8*, 560098. https://doi.org/10.3389/fcell.2020.560098
57. Bantele, S.; Pfander, B. Nucleosome Remodeling by Fun30^{SMARCAD1} in the DNA Damage Response. *Front. Mol. Biosci.* **2019**, *6*, 78. https://doi.org/10.3389/fmolb.2019.00078
58. de Castro, R.O.; Previato, L.; Goitea, V.; Felberg, A.; Guiraldelli, M.F.; Filiberti, A.; Pezza, R.J. The chromatin –remodeling subunit Baf200 promotes homology –directed DNA repair and regulates distinct chromatin –remodeling complexes. *J. Biol. Chem.* **2017**, *292*(20), 8459–8471. https://doi.org/10.1074/jbc.M117.778183
59. Oba, A.; Shimada, S.; Akiyama, Y.; Nishikawaji, T.; Mogushi, K.; Ito, H.; Matsumura, S.; Aihara, A.; Mitsunori, Y.; Ban, D.; Ochiai, T.; Kudo, A.; Asahara, H.; Kaida, A.; Miura, M.; Tanabe, M.; Tanaka, S. ARID2 modulates DNA damage response in human hepatocellular carcinoma cells. *J. Hepatol.* **2017**, *66*(5), 942–951. https://doi.org/10.1016/j.jhep.2016.12.026
60. Lu, H.; Guo, X.; Meng, X.; Liu, J.; Allen, C.; Wray, J.; Nickoloff, J.A.; Shen, Z. The BRCA2 –interacting protein BCCIP functions in RAD51 and BRCA2 focus formation and homologous recombinational repair. *Mol. Cell. Biol.* **2005**, *25*(5), 1949–1957. https://doi.org/10.1128/MCB.25.5.1949 –1957.2005
61. Liu, X.; Cao, L.; Ni, J.; Liu, N.; Zhao, X.; Wang, Y.; Zhu, L.; Wang, L.; Wang, J.; Yue, Y.; Cai, Y.; Jin, J. Differential BCCIP gene expression in primary human ovarian cancer, renal cell carcinoma and colorectal cancer tissues. *International journal of oncology* **2013**, *43*(6), 1925–1934. https://doi.org/10.3892/ijo.2013.2124

62. Bassermann, F.; Frescas, D.; Guardavaccaro, D.; Busino, L.; Peschiaroli, A.; Pagano, M. The Cdc14B –Cdh1 –Plk1 axis controls the G2 DNA –damage –response checkpoint. *Cell* **2008**, *134*(2), 256–267. https://doi.org/10.1016/j.cell.2008.05.043
63. Zhang, Y.M.; Meng, L.B.; Yu, S.J.; Ma, D.X. Identification of potential crucial genes in monocytes for atherosclerosis using bioinformatics analysis. *Int. J. Med. Res.* **2020**, *48*(4), 300060520909277. https://doi.org/10.1177/0300060520909277
64. Murphy, K.J.; Cutter, A.R.; Fang, H.; Postnikov, Y.V.; Bustin, M.; Hayes, J.J. HMGN1 and 2 remodel core and linker histone tail domains within chromatin. *Nucleic Acids Res.* **2017**, *45*(17), 9917–9930. https://doi.org/10.1093/nar/gkx579
65. Postnikov, Y.V.; Kurahashi, T.; Zhou, M.; Bustin, M. The nucleosome binding protein HMGN1 interacts with PCNA and facilitates its binding to chromatin. *Mol. Cell. Biol.* **2012**, *32*(10), 1844–1854. https://doi.org/10.1128/MCB.06429 –11
66. Subramanian, M.; Gonzalez, R.W.; Patil, H.; Ueda, T.; Lim, J.H.; Kraemer, K.H.; Bustin, M.; Bergel, M. The nucleosome –binding protein HMGN2 modulates global genome repair. *FEBS J.* **2009**, *276*(22), 6646–6657. https://doi.org/10.1111/j.1742 –4658.2009.07375.x
67. Hemphill, W.O.; Perrino, F.W. Measuring TREX1 and TREX2 exonuclease activities. *Methods Enzymol.* **2019**, *625*, 109–133. https://doi.org/10.1016/bs.mie.2019.05.004
68. Manils, J.; Gómez, D.; Salla –Martret, M.; Fischer, H.; Fye, J.M.; Marzo, E.; Marruecos, L.; Serrano, I.; Salgado, R.; Rodrigo, J.P.; Garcia –Pedrero, J.M.; Serafin, A.M.; Cañas, X.; Benito, C.; Toll, A., Forcales, S.V.; Perrino, F.W.; Eckhart, L.; Soler, C. Multifaceted role of TREX2 in the skin defense against UV –induced skin carcinogenesis. *Oncotarget* **2015**, *6*(26), 22375–22396. https://doi.org/10.18632/oncotarget.4296
69. Deegan, T.D.; Mukherjee, P.P.; Fujisawa, R.; Polo Rivera, C.; Labib, K. CMG helicase disassembly is controlled by replication fork DNA, replisome components and a ubiquitin threshold. *eLife* **2020**, *9*, e60371. https://doi.org/10.7554/eLife.60371
70. Cortez, D.; Glick, G.; Elledge, S.J. Minichromosome maintenance proteins are direct targets of the ATM and ATR checkpoint kinases *Proc. Natl. Acad. Sci U. S. A.***2004**, *101*(27), 10078–10083. https://doi.org/10.1073/pnas.0403410101
71. Oh, J.H.; Hyun, J.Y.; Chen, S.J.; Varshavsky, A. Five enzymes of the Arg/N –degron pathway form a targeting complex: The concept of superchanneling. *Proc. Natl. Acad. Sci U. S. A.* **2020**, May 19;117(20):10778 –10788. doi: 10.1073/pnas.2003043117. Epub 2020 May 4. PMID: 32366662; PMCID: PMC7245096.
72. Song, E.; Fan, P.; Huang, B.; Deng, H.B.; Cheung, B.M.; Félétou, M.; Vilaine, J.P.; Villeneuve, N.; Xu, A.; Vanhoutte, P. M.; Wang, Y. Deamidated lipocalin –2 induces endothelial dysfunction and hypertension in dietary obese mice. *J. Am. Heart Assoc.***2014**, *3*(2), e000837. https://doi.org/10.1161/JAHA.114.000837
73. Dutta, B.; Park, J.E.; Kumar, S.; Hao, P.; Gallart –Palau, X.; Serra, A.; Ren, Y.; Sorokin, V.; Lee, C.N.; Ho, H.H.; de Kleijn, D.; Sze, S.K. Monocyte adhesion to atherosclerotic matrix proteins is enhanced by Asn –Gly –Arg deamidation. *Sci. Rep.* **2017**, *7*(1), 5765. https://doi.org/10.1038/s41598 –017 –06202 –2
74. Li, Y.; Bolderson, E.; Kumar, R.; Muniandy, P.A.; Xue, Y.; Richard, D.J.; Seidman, M.; Pandita, T.K.; Khanna, K.K.; Wang, W. HSSB1 and hSSB2 form similar multiprotein

complexes that participate in DNA damage response. *J. Biol. Chem.***2009**, *284*(35), 23525–23531. https://doi.org/10.1074/jbc.C109.039586
75. Li, J.; Ma, X.; Banerjee, S.; Baruah, S.; Schnicker, N.J.; Roh, E.; Ma, W.; Liu, K.; Bode, A.M.; Dong, Z. Structural basis for multifunctional roles of human Ints3 C –terminal domain. *J. Biol. Chem.***2021**, *296*, 100112. https://doi.org/10.1074/jbc.RA120.016393
76. Çağlayan, M. Pol μ ribonucleotide insertion opposite 8 –oxodG facilitates the ligation of premutagenic DNA repair intermediate. *Sci. Rep.* **2020**, *10*(1), 940. https://doi.org/10.1038/s41598 –020 –57886 –y
77. Ghosh, D.; Raghavan, S.C. 20 years of DNA Polymerase μ, the polymerase that still surprises. *FEBS J.* **2021**, 10.1111/febs.15852. Advance online publication. https://doi.org/10.1111/febs.15852
78. Jurkiw, T.J.; Tumbale, P.P.; Schellenberg, M.J.; Cunningham –Rundles, C.; Williams, R.S.; O'Brien, P.J. LIG1 syndrome mutations remodel a cooperative network of ligand binding interactions to compromise ligation efficiency. *Nucleic Acids Res.* **2021**, *49*(3), 1619–1630. https://doi.org/10.1093/nar/gkaa1297
79. Bold, I.T.; Specht, A.K.; Droste, C.F.; Zielinski, A.; Meyer, F.; Clauditz, T.S.; Münscher, A.; Werner, S.; Rothkamm, K.; Petersen, C.; Borgmann, K. DNA Damage Response during Replication Correlates with CIN70 Score and Determines Survival in HNSCC Patients. *Cancers* **2021**, *13*(6), 1194. https://doi.org/10.3390/cancers13061194
80. Słabicki, M.; Theis, M., Krastev, D.B.; Samsonov, S.; Mundwiller, E.; Junqueira, M.; Paszkowski –Rogacz, M.; Teyra, J.; Heninger, A.K.; Poser, I.; Prieur, F.; Truchetto, J.; Confavreux, C.; Marelli, C.; Durr, A.; Camdessanche, J.P.; Brice, A.; Shevchenko, A.; Pisabarro, M.T.; Stevanin, G.; Buchholz, F. A genome –scale DNA repair RNAi screen identifies SPG48 as a novel gene associated with hereditary spastic paraplegia. *PLoS Biol.* **2010**, *8*(6), e1000408. https://doi.org/10.1371/journal.pbio.1000408
81. Hirst, J.; Barlow, L.D.; Francisco, G.C.; Sahlender, D.A.; Seaman, M.N.; Dacks, J.B.; Robinson, M.S. The fifth adaptor protein complex. *PLoS Biol.* **2011**, *9*(10), e1001170. https://doi.org/10.1371/journal.pbio.1001170
82. Ryu, S.; Teles, F.; Minopoli, G.; Russo, T.; Rosenfeld, M.G.; Suh, Y. An epigenomic role of Fe65 in the cellular response to DNA damage. *Mutat. Res.***2015**, *776*, 40–47. https://doi.org/10.1016/j.mrfmmm.2015.01.006
83. Stante, M.; Minopoli, G.; Passaro, F.; Raia, M.; Vecchio, L.D.; Russo, T. Fe65 is required for Tip60 –directed histone H4 acetylation at DNA strand breaks. *Proc. Natl. Acad. Sci U. S. A.* **2009**, *106*(13), 5093–5098. https://doi.org/10.1073/pnas.0810869106
84. Gao, Y.; Gan, H.; Lou, Z.; Zhang, Z. Asf1a resolves bivalent chromatin domains for the induction of lineage –specific genes during mouse embryonic stem cell differentiation. *Proc. Natl. Acad. Sci U. S. A.* **2018**, *115*(27), E6162–E6171. https://doi.org/10.1073/pnas.1801909115
85. Lee, K.Y.; Im, J.S.; Shibata, E.; Dutta, A. ASF1a Promotes Non –homologous End Joining Repair by Facilitating Phosphorylation of MDC1 by ATM at Double –Strand Breaks. *Mol. Cell* **2017**, *68*(1), 61–75.e5. https://doi.org/10.1016/j.molcel.2017.08.021

86. Wu, Y.; Li, X.; Yu, J.; Björkholm, M.; Xu, D. ASF1a inhibition induces p53 –dependent growth arrest and senescence of cancer cells. *Cell Death Dis.* **2019**, *10*(2), 76. https://doi.org/10.1038/s41419 –019 –1357 –z
87. Friedberg, E.C.; Wood, R.D. New insights into the combined Cockayne/xeroderma pigmentosum complex: human XPG protein can function in transcription factor stability. *Mol. Cell* **2007**, *26*(2), 162–164. https://doi.org/10.1016/j.molcel.2007.04.002
88. Durik, M.; Kavousi, M.; van der Pluijm, I.; Isaacs, A.; Cheng, C.; Verdonk, K.; Loot, A.E.; Oeseburg, H.; Bhaggoe, U.M.; Leijten, F.; van Veghel, R.; de Vries, R.; Rudez, G.; Brandt, R.; Ridwan, Y.R.; van Deel, E.D.; de Boer, M.; Tempel, D.; Fleming, I.; Mitchell, G.F.; Verwoert, G.C.; Tarasov, K.V.; Uitterlinden, A.G.; Hofman, A.; Duckers, H.J.; van Duijn, C.M.; Oostra, B.A.; Witteman, J.C.; Duncker, D.J.; Danser, A.H.; Hoeijmakers, J.H.; Roks, A.J. Nucleotide excision DNA repair is associated with age –related vascular dysfunction. *Circulation* **2012**, *126*(4), 468–478. https://doi.org/10.1161/CIRCULATIONAHA.112.104380
89. Ferri, D.; Orioli. D.; Botta, E. Heterogeneity and overlaps in nucleotide excision repair disorders. *Clin Genet.* **2020** Jan;97(1):12 –24. doi: 10.1111/cge.13545. Epub 2019 Apr 22. PMID: 30919937.
90. Ghosh, D.; Raghavan, S. C. Nonhomologous end joining: new accessory factors fine tune the machinery. *Trends Genet.***2021**, *37*(6), 582–599. https://doi.org/10.1016/j.tig.2021.03.001
91. Jia, Y.; Cheng, Z.; Bharath, S.R.; Sun, Q.; Su, N.; Huang, J.; Song, H. Crystal structure of the INTS3/INTS6 complex reveals the functional importance of INTS3 dimerization in DSB repair. *Cell discovery* **2021**, *7*(1), 66. https://doi.org/10.1038/s41421 –021 –00283 –0
92. Zhang, F.; Ma, T.; Yu, X. A core hSSB1 –INTS complex participates in the DNA damage response. *J. Cell. Sci.* **2013**, *126*(Pt 21), 4850–4855. https://doi.org/10.1242/jcs.132514
93. Hoffman, A.E.; Zheng, T.; Ba, Y.; Zhu, Y. The circadian gene NPAS2, a putative tumor suppressor, is involved in DNA damage response. *Molecular Cancer Res. : MCR* **2008**, *6*(9), 1461–1468. https://doi.org/10.1158/1541 –7786.MCR –07 –2094
94. Nernpermpisooth, N.; Qiu, S.; Mintz, J.D.; Suvitayavat, W.; Thirawarapan, S.; Rudic, D.R.; Fulton, D.J.; Stepp, D.W. Obesity alters the peripheral circadian clock in the aorta and microcirculation. *Microcirculation* **2015**, *22*(4), 257–266. https://doi.org/10.1111/micc.12192
95. Morales, C.; Ruiz –Torres, M.; Rodríguez –Acebes, S.; Lafarga, V.; Rodríguez –Corsino, M.; Megías, D.; Cisneros, D.A.; Peters, J.M.; Méndez, J.; Losada, A. PDS5 proteins are required for proper cohesin dynamics and participate in replication fork protection. *J. Biol. Chem.***2020**, *295*(1), 146–157. https://doi.org/10.1074/jbc.RA119.011099
96. Zhang, N.; Coutinho, L.E.; Pati, D. PDS5A and PDS5B in Cohesin Function and Human Disease. *Int. J. Mol. Sci.* **2021**, *22*(11), 5868. https://doi.org/10.3390/ijms22115868

97. Marchand, B.; Pitarresi, J.R.; Reichert, M.; Suzuki, K.; Laczkó, D.; Rustgi, A.K. PRRX1 isoforms cooperate with FOXM1 to regulate the DNA damage response in pancreatic cancer cells. *Oncogene* **2019**, *38*(22), 4325–4339. https://doi.org/10.1038/s41388 –019 –0725 –6
98. Bosada, F.M.; Rivaud, M.R.; Uhm, J.S.; Verheule, S.; van Duijvenboden, K.; Verkerk, A.O.; Christoffels, V. M.; Boukens, B.J. A Variant Noncoding Region Regulates *Prrx1* and Predisposes to Atrial Arrhythmias. *Circ. Res.***2021**, *129*(3), 420–434. https://doi.org/10.1161/CIRCRESAHA.121.319146
99. Higuchi, M.; Kato, T.; Yoshida, S.; Ueharu, H.; Nishimura, N.; Kato, Y. PRRX1 – and PRRX2 –positive mesenchymal stem/progenitor cells are involved in vasculogenesis during rat embryonic pituitary development. *Cell Tissue Res.* **2015**, *361*(2), 557–565. https://doi.org/10.1007/s00441 –015 –2128 –5
100. Wang, X.; Yang, R.; Wang, Q.; Wang, Y.; Ci, H.; Wu, S. Aberrant expression of vasculogenic mimicry, PRRX1, and CIP2A in clear cell renal cell carcinoma and its clinicopathological significance. *Medicine* **2019**, *98*(36), e17028. https://doi.org/10.1097/MD.0000000000017028
101. Chen, Z.; Chen, Y.; Li, Y.; Lian, W.; Zheng, K.; Zhang, Y.; Zhang, Y.; Lin, C.; Liu, C.; Sun, F.; Sun, X.; Wang, J.; Zhao, L.; Ke, Y. Prrx1 promotes stemness and angiogenesis via activating TGF –β/smad pathway and upregulating proangiogenic factors in glioma. *Cell Death Dis.* **2021**, *12*(6), 615. https://doi.org/10.1038/s41419 –021 –03882 –7
102. Bellelli, R.; Youds, J.; Borel, V.; Svendsen, J.; Pavicic –Kaltenbrunner, V.; Boulton, S.J. Synthetic Lethality between DNA Polymerase Epsilon and RTEL1 in Metazoan DNA Replication. *Cell Rep.* **2020**, *31*(8), 107675. https://doi.org/10.1016/j.celrep.2020.107675
103. Björkman, A.; Johansen, S. L.; Lin, L.; Schertzer, M.; Kanellis, D.C.; Katsori, A.M.; Christensen, S.T.; Luo, Y.; Andersen, J.S.; Elsässer, S.J.; Londono –Vallejo, A.; Bartek, J.; Schou, K.B. Human RTEL1 associates with Poldip3 to facilitate responses to replication stress and R –loop resolution. *Genes Dev.* **2020**, *34*(15 –16), 1065–1074. https://doi.org/10.1101/gad.330050.119
104. Mancini, M.; Petretto, E.; Kleinert, C.; Scavone, A.; De, T.; Cook, S.; Silhavy, J.; Zidek, V.; Pravenec, M.; d'Amati, G.; Camici, P.G. Mapping genetic determinants of coronary microvascular remodeling in the spontaneously hypertensive rat. *Basic Res. Cardiol.* **2013**, *108*(1), 316. https://doi.org/10.1007/s00395 –012 –0316 –y
105. Kimura, Y.; Izumiya, Y.; Araki, S.; Yamamura, S.; Hanatani, S.; Onoue, Y.; Ishida, T.; Arima, Y.; Nakamura, T.; Yamamoto, E.; Senokuchi, T.; Yoshizawa, T.; Sata, M., Kim –Mitsuyama, S.; Nakagata, N.; Bober, E.; Braun, T.; Kaikita, K.; Yamagata, K.; Tsujita, K. Sirt7 Deficiency Attenuates Neointimal Formation Following Vascular Injury by Modulating Vascular Smooth Muscle Cell Proliferation. *Circ. J.***2021**, 10.1253/circj.CJ –20 –0936. Advance online publication. https://doi.org/10.1253/circj.CJ –20 –0936
106. Li, L.; Shi, L.; Yang, S.; Yan, R.; Zhang, D.; Yang, J.; He, L.; Li, W.; Yi, X.; Sun, L.; Liang, J.; Cheng, Z.; Shi, L.; Shang, Y.; Yu, W. SIRT7 is a histone desuccinylase that

functionally links to chromatin compaction and genome stability. *Nat. Commun.***2016**, *7*, 12235. https://doi.org/10.1038/ncomms12235

107. Tang, M.; Tang, H.; Tu, B.; Zhu, W.G. SIRT7: a sentinel of genome stability. *Open Biol.* **2021**, *11*(6), 210047. https://doi.org/10.1098/rsob.210047

108. Zheng, J.; Chen, K.; Wang, H.; Chen, Z.; Xi, Y.; Yin, H.; Lai, K.; Liu, Y. SIRT7 Regulates the Vascular Smooth Muscle Cells Proliferation and Migration via Wnt/*β* –Catenin Signaling Pathway. *Biomed Res. Int.* **2018**, *2018*, 4769596. https://doi.org/10.1155/2018/4769596

109. Prakash, R.; Sandoval, T.; Morati, F.; Zagelbaum, J.A.; Lim, P.X.; White, T.; Taylor, B.; Wang, R.; Desclos, E.; Sullivan, M.R.; Rein, H.L.; Bernstein, K.A.; Krawczyk, P.M.; Gautier, J.; Modesti, M.; Vanoli, F.; Jasin, M. Distinct pathways of homologous recombination controlled by the SWS1 –SWSAP1 –SPIDR complex. *Nat. Commun.***2021**, *12*(1), 4255. https://doi.org/10.1038/s41467 –021 –24205 –6

110. Martino, J.; Brunette, G.J.; Barroso –González, J.; Moiseeva, T.N.; Smith, C.M.; Bakkenist, C.J.; O'Sullivan, R.J.; Bernstein, K.A. The human Shu complex functions with PDS5B and SPIDR to promote homologous recombination. *Nucleic Acids Res.* **2019**, *47*(19), 10151–10165. https://doi.org/10.1093/nar/gkz738

111. Wan, L.; Han, J.; Liu, T.; Dong, S.; Xie, F.; Chen, H.; Huang, J. Scaffolding protein SPIDR/KIAA0146 connects the Bloom syndrome helicase with homologous recombination repair. *Proc. Natl. Acad. Sci U. S. A.* **2013**, *110*(26), 10646–10651. https://doi.org/10.1073/pnas.1220921110

112. Thomas, A.; Pommier, Y. Targeting Topoisomerase I in the Era of Precision Medicine. *Clin. Cancer Res.* **2019**, *25*(22), 6581–6589. https://doi.org/10.1158/1078 –0432.CCR –19 –1089

113. Bizard, A.H.; Hickson, I.D. The many lives of type IA topoisomerases. *J. Biol. Chem.* **2020,** May 15;295(20):7138 –7153. doi: 10.1074/jbc.REV120.008286. Epub **2020,** Apr 10. PMID: 32277049; PMCID: PMC7242696.

114. Tsai, H.Z.; Lin, R.K.; Hsieh, T.S. Drosophila mitochondrial topoisomerase III alpha affects the aging process via maintenance of mitochondrial function and genome integrity. *J. Biomed. Sci.* **2016,** Apr 12;23:38. doi: 10.1186/s12929 –016 –0255 –2. PMID: 27067525; PMCID: PMC4828762.

115. Xia, X.; Hu, T.; He, J.; Xu, Q.; Yu, C.; Liu, X.; Shao, Z.; Liao, Y.; Huang, H.; Liu, N. USP10 deletion inhibits macrophage –derived foam cell formation and cellular –oxidized low density lipoprotein uptake by promoting the degradation of CD36. *Aging* **2020**, *12*(22), 22892–22905. https://doi.org/10.18632/aging.104003

116. Zhang, M.; Hu, C.; Tong, D.; Xiang, S.; Williams, K.; Bai, W.; Li, G.M.; Bepler, G.; Zhang, X. Ubiquitin –specific Peptidase 10 (USP10) Deubiquitinates and Stabilizes MutS Homolog 2 (MSH2) to Regulate Cellular Sensitivity to DNA Damage. *J. Biol. Chem.* **2016**, *291*(20), 10783–10791. https://doi.org/10.1074/jbc.M115.700047

117. Wang, Z.; Zhang, H.; Liu, J.; Cheruiyot, A.; Lee, J. H.; Ordog, T.; Lou, Z.; You, Z.; Zhang, Z. USP51 deubiquitylates H2AK13,15ub and regulates DNA damage response. *Genes Dev.* **2016**, *30*(8), 946–959. https://doi.org/10.1101/gad.271841.115

118. Walser, F.; Mulder, M.; Bragantini, B.; Burger, S.; Gubser, T.; Gatti, M.; Botuyan, M.V.; Villa, A.; Altmeyer, M.; Neri, D.; Ovaa, H.; Mer, G.; Penengo, L. Ubiquitin Phosphorylation at Thr12 Modulates the DNA Damage Response. *Mol. Cell* **2020**, *80*(3), 423–436.e9. https://doi.org/10.1016/j.molcel.2020.09.017
119. Schmitz, U.; Ishida, M.; Berk, B. C. Angiotensin II stimulates tyrosine phosphorylation of phospholipase C –gamma –associated proteins. Characterization of a c –Src –dependent 97 –kD protein in vascular smooth muscle cells. *Circ. Res.***1997**, *81*(4), 550–557. https://doi.org/10.1161/01.res.81.4.550
120. Tao, W.; Hong, Y.; He, H.; Han, Q.; Mao, M.; Hu, B.; Zhang, H.; Huang, X.; You, W.; Liang, X.; Zhang, Y.; Li, X. MicroRNA –199a –5p aggravates angiotensin II –induced vascular smooth muscle cell senescence by targeting Sirtuin –1 in abdominal aortic aneurysm. *J. Cell. Mol. Med.* **2021**, *25*(13), 6056–6069. Advance online publication. https://doi.org/10.1111/jcmm.16485
121. Swan, R.L.; Cowell, I.G.; Austin, C.A. A Role for VCP/p97 in the Processing of Drug –Stabilized TOP2 –DNA Covalent Complexes. *Mol. Pharmacol.* **2021**, *100*(1), 57–62. https://doi.org/10.1124/molpharm.121.000262
122. Kilgas, S.; Singh, A.N.; Paillas, S.; Then, C.K.; Torrecilla, I.; Nicholson, J.; Browning, L.; Vendrell, I.; Konietzny, R.; Kessler, B.M.; Kiltie, A.E.; Ramadan, K. p97/VCP inhibition causes excessive MRE11 –dependent DNA end resection promoting cell killing after ionizing radiation. *Cell Rep.* **2021**, *35*(8), 109153. https://doi.org/10.1016/j.celrep.2021.109153
123. Kim, H.; Dejsuphong, D.; Adelmant, G.; Ceccaldi, R.; Yang, K.; Marto, J.A.; D'Andrea, A.D. Transcriptional repressor ZBTB1 promotes chromatin remodeling and translesion DNA synthesis. *Mol. Cell* **2014**, *54*(1), 107–118. https://doi.org/10.1016/j.molcel.2014.02.017
124. Denton, K.; Mou, Y.; Xu, C.C.; Shah, D.; Chang, J.; Blackstone, C.; Li, X.J. Impaired mitochondrial dynamics underlie axonal defects in hereditary spastic paraplegias. *Hum. Mol. Genet.* **2018**, *27*(14), 2517–2530. https://doi.org/10.1093/hmg/ddy156
125. Hurst, V.; Shimada, K.; Gasser, S.M. Nuclear Actin and Actin –Binding Proteins in DNA Repair. *Trends Cell Biol.* **2019**, *29*(6), 462–476. https://doi.org/10.1016/j.tcb.2019.02.010
126. Lv, P.; Zhang, F.; Yin, Y.J.; Wang, Y.C.; Gao, M.; Xie, X.L.; Zhao, L.L.; Dong, L.H.; Lin, Y.L.; Shu, Y.N.; Zhang, D.D.; Liu, G.X.; Han, M. SM22α inhibits lamellipodium formation and migration via Ras –Arp2/3 signaling in synthetic VSMCs. *Am. J. Physiol. Cell Physiol.* **2016**, *311*(5), C758–C767. https://doi.org/10.1152/ajpcell.00033.2016
127. Nolen, B.J.; Tomasevic, N.; Russell, A.; Pierce, D.W.; Jia, Z.; McCormick, C.D.; Hartman, J.; Sakowicz, R.; Pollard, T.D. Characterization of two classes of small molecule inhibitors of Arp2/3 complex. *Nature* **2009**, *460*(7258), 1031–1034. https://doi.org/10.1038/nature08231

128. Collis, S.J.; Barber, L.J.; Clark, A.J.; Martin, J.S.; Ward, J.D.; Boulton, S.J. HCLK2 is essential for the mammalian S –phase checkpoint and impacts on Chk1 stability. *Nat. Cell Biol.* **2007**, *9*(4), 391–401. https://doi.org/10.1038/ncb1555
129. Iwai, K.; Yaguchi, M.; Nishimura, K.; Yamamoto, Y.; Tamura, T.; Nakata, D.; Dairiki, R.; Kawakita, Y.; Mizojiri, R.; Ito, Y.; Asano, M.; Maezaki, H.; Nakayama, Y., Kaishima, M.; Hayashi, K.; Teratani, M.; Miyakawa, S.; Iwatani, M., Miyamoto, M.; Klein, M. G.; Lane, W.; Snell, G.; Tjhen, R.; He, X.; Pulukuri, S.; Nomura, T. Anti – tumor efficacy of a novel CLK inhibitor via targeting RNA splicing and MYC – dependent vulnerability. *EMBO Mol. Med.* **2018**, *10*(6), e8289. https://doi.org/10.15252/emmm.201708289
130. Eisenreich, A.; Bogdanov, V.Y., Zakrzewicz, A.; Pries, A.; Antoniak, S.; Poller, W.; Schultheiss, H.P.; Rauch, U. Cdc2 –like kinases and DNA topoisomerase I regulate alternative splicing of tissue factor in human endothelial cells. *Circ. Res.***2009**, 104(5), 589–599. https://doi.org/10.1161/CIRCRESAHA.108.183905
131. Pan, L.; Yu, Y.; Yu, M.; Yao, S.; Mu, Q.; Luo, G.; Xu, N. Expression of flTF and asTF splice variants in various cell strains and tissues. *Mol. Med. Rep.* **2019**, *19*(3), 2077–2086. https://doi.org/10.3892/mmr.2019.9843
132. Amangyeld, T.; Shin, Y.K.; Lee, M.; Kwon, B.; Seo, Y.S. Human MUS81 –EME2 can cleave a variety of DNA structures including intact Holliday junction and nicked duplex. *Nucleic Acids Res.* **2014**, *42*(9), 5846–5862. https://doi.org/10.1093/nar/gku237
133. Falquet, B.; Rass, U. Structure –Specific Endonucleases and the Resolution of Chromosome Underreplication. *Genes* **2019**, *10*(3), 232. https://doi.org/10.3390/genes10030232
134. Roy A.L. Role of the multifunctional transcription factor TFII –I in DNA damage repair. *DNA repair* **2021**, *106*, 103175. Advance online publication. https://doi.org/10.1016/j.dnarep.2021.103175
135. Fattah, F.J.; Hara, K.; Fattah, K.R.; Yang, C.; Wu, N.; Warrington, R.; Chen, D.J.; Zhou, P.; Boothman, D.A.; Yu, H. The transcription factor TFII –I promotes DNA translesion synthesis and genomic stability. *PLoS Genet.* **2014**, *10*(6), e1004419. https://doi.org/10.1371/journal.pgen.1004419
136. Wu, W.; Sato, K.; Koike, A.; Nishikawa, H.; Koizumi, H.; Venkitaraman, A.R.; Ohta, T. HERC2 is an E3 ligase that targets BRCA1 for degradation. *Cancer Res.* **2010**, *70*(15), 6384–6392. https://doi.org/10.1158/0008 –5472.CAN –10 –1304
137. Kang, T.H.; Lindsey –Boltz, L.A.; Reardon, J.T.; Sancar, A. Circadian control of XPA and excision repair of cisplatin –DNA damage by cryptochrome and HERC2 ubiquitin ligase. *Proc. Natl. Acad. Sci U. S. A.* **2010**, *107*(11), 4890–4895. https://doi.org/10.1073/pnas.0915085107
138. Mathieu, N.A.; Levin, R.H.; Spratt, D.E. Exploring the Roles of HERC2 and the NEDD4L HECT E3 Ubiquitin Ligase Subfamily in p53 Signaling and the DNA Damage Response. *Front. Oncol.* **2021**, *11*, 659049. https://doi.org/10.3389/fonc.2021.659049
139. Duan, Y.; Huang, S.; Yang, J.; Niu, P.; Gong, Z.; Liu, X.; Xin, L.; Currie, R.W.; Wu, T. HspA1A facilitates DNA repair in human bronchial epithelial cells exposed to

Benzo[a]pyrene and interacts with casein kinase 2. *Cell Stress Chaperones* **2014**, *19*(2), 271–279. https://doi.org/10.1007/s12192 –013 –0454 –7
140. Dulin, E.; García –Barreno, P.; Guisasola, M.C. Genetic variations of HSPA1A, the heat shock protein levels, and risk of atherosclerosis. *Cell Stress Chaperones* **2012**, *17*(4), 507–516. https://doi.org/10.1007/s12192 –012 –0328 –4
141. McPherson, M.T.; Holub, A.S.; Husbands, A.Y.; Petreaca, R.C. Mutation Spectra of the MRN (MRE11, RAD50, NBS1/NBN) Break Sensor in Cancer Cells. *Cancers* **2020**, *12*(12), 3794. https://doi.org/10.3390/cancers12123794
142. Wang, Q.; Goldstein, M.; Alexander, P.; Wakeman, T.P.; Sun, T.; Feng, J.; Lou, Z.; Kastan, M.B.; Wang, X.F. Rad17 recruits the MRE11 –RAD50 –NBS1 complex to regulate the cellular response to DNA double –strand breaks. *EMBO J.* **2014**, *33*(8), 862–877. https://doi.org/10.1002/embj.201386064
143. Chen, L.; Cai, Y.; Jin, J.; Florens, L.; Swanson, S.K.; Washburn, M.P.; Conaway, J.W.; Conaway, R.C. Subunit organization of the human INO80 chromatin remodeling complex: an evolutionarily conserved core complex catalyzes ATP –dependent nucleosome remodeling. *J. Biol. Chem.***2011**, *286*(13), 11283–11289. https://doi.org/10.1074/jbc.M111.222505
144. Shan, C.M.; Bao, K.; Diedrich, J.; Chen, X.; Lu, C.; Yates, J.R. 3rd; Jia, S. The INO80 Complex Regulates Epigenetic Inheritance of Heterochromatin. *Cell Rep.* **2020**, *33*(13), 108561. https://doi.org/10.1016/j.celrep.2020.108561
145. Abe, T.; Ooka, M.; Kawasumi, R.; Miyata, K.; Takata, M.; Hirota, K.; Branzei, D. Warsaw breakage syndrome DDX11 helicase acts jointly with RAD17 in the repair of bulky lesions and replication through abasic sites. *Proc. Natl. Acad. Sci U. S. A.* **2018**, *115*(33), 8412–8417. https://doi.org/10.1073/pnas.1803110115
146. Tsuda, M.; Kitamasu, K.; Kumagai, C.; Sugiyama, K.; Nakano, T.; Ide, H. Tyrosyl –DNA phosphodiesterase 2 (TDP2) repairs topoisomerase 1 DNA –protein crosslinks and 3' –blocking lesions in the absence of tyrosyl –DNA phosphodiesterase 1 (TDP1). *DNA repair* **2020**, *91 –92*, 102849. https://doi.org/10.1016/j.dnarep.2020.102849
147. Schellenberg, M.J.; Appel, C.D.; Riccio, A.A.; Butler, L.R.; Krahn, J.M.; Liebermann, J.A.; Cortés –Ledesma, F.; Williams, R.S. Ubiquitin stimulated reversal of topoisomerase 2 DNA –protein crosslinks by TDP2. *Nucleic Acids Res.* **2020**, *48*(11), 6310–6325. https://doi.org/10.1093/nar/gkaa318
148. Huang, S.N.; Pommier, Y. Mammalian Tyrosyl –DNA Phosphodiesterases in the Context of Mitochondrial DNA Repair. *Int. J. Mol. Sci.* **2019**, *20*(12), 3015. https://doi.org/10.3390/ijms20123015
149. Feris, E.J.; Hinds, J.W.; Cole, M.D. Formation of a structurally –stable conformation by the intrinsically disordered MYC:TRRAP complex. *PloS One* **2019**, *14*(12), e0225784. https://doi.org/10.1371/journal.pone.0225784
150. Abbey, D.; Conlon, D.; Rainville, C.; Elwyn, S.; Quiroz –Figueroa, K.; Billheimer, J.; Schultz, D.C.; Hand, N.J.; Cherry, S.; Rader, D.J. Lipid droplet screen in human hepatocytes identifies TRRAP as a regulator of cellular triglyceride metabolism. *Clin. Transl. Sci.* **2021**, *14*(4), 1369–1379. https://doi.org/10.1111/cts.12988

151. Taskinen, M.R.; Packard, C.J.; Borén, J. Emerging Evidence that ApoC –III Inhibitors Provide Novel Options to Reduce the Residual CVD. *Curr. Atheroscler. Rep.* **2019**, *21*(8), 27. https://doi.org/10.1007/s11883 –019 –0791 –9
152. Jung, M.; Ramanagoudr –Bhojappa, R.; van Twest, S.; Rosti, R.O.; Murphy, V.; Tan, W.; Donovan, F.X.; Lach, F.P.; Kimble, D.C.; Jiang, C.S.; Vaughan, R.; Mehta, P.A.; Pierri, F.; Dufour, C.; Auerbach, A.D.; Deans, A.J.; Smogorzewska, A.; Chandrasekharappa, S.C. Association of clinical severity with FANCB variant type in Fanconi anemia. *Blood* **2020**, *135*(18), 1588–1602. https://doi.org/10.1182/blood.2019003249
153. Nomura, Y.; Adachi, N.; Koyama, H. Human Mus81 and FANCB independently contribute to repair of DNA damage during replication. *Genes Cells.* **2007**, *12*(10), 1111–1122. https://doi.org/10.1111/j.1365 –2443.2007.01124.x
154. Benedict, B.; van Bueren, M.A.; van Gemert, F.P.; Lieftink, C.; Guerrero Llobet, S.; van Vugt, M.A.; Beijersbergen, R.L.; Te Riele, H. The RECQL helicase prevents replication fork collapse during replication stress. *Life Sci. Alliance* **2020**, *3*(10), e202000668. https://doi.org/10.26508/lsa.202000668
155. Debnath, S.; Sharma, S. RECQ1 Helicase in Genomic Stability and Cancer. *Genes* **2020**, *11*(6), 622. https://doi.org/10.3390/genes11060622
156. Giovannini, S.; Weller, M.C.; Hanzlíková, H.; Shiota, T.; Takeda, S.; Jiricny, J. ATAD5 deficiency alters DNA damage metabolism and sensitizes cells to PARP inhibition. *Nucleic Acids Res.* **2020**, *48*(9), 4928–4939. https://doi.org/10.1093/nar/gkaa255
157. Shiomi, Y.; Nishitani, H. Control of Genome Integrity by RFC Complexes; Conductors of PCNA Loading onto and Unloading from Chromatin during DNA Replication. *Genes* **2017**, *8*(2), 52. https://doi.org/10.3390/genes8020052
158. Coggins, S.A.; Mahboubi, B.; Schinazi, R.F.; Kim, B. SAMHD1 Functions and Human Diseases. *Viruses* **2020**, *12*(4), 382. https://doi.org/10.3390/v12040382
159. Daddacha, W.; Koyen, A.E.; Bastien, A.J.; Head, P.E.; Dhere, V.R.; Nabeta, G.N.; Connolly, E.C.; Werner, E.; Madden, M.Z.; Daly, M.B.; Minten, E.V.; Whelan, D.R.; Schlafstein, A.J.; Zhang, H.; Anand, R.; Doronio, C.; Withers, A.E.; Shepard, C.; Sundaram, R.K.; Deng, X.; Dynan, W.S.; Wang, Y.; Bindra, R.S.; Cejka, P.; Rothenberg, E.; Doetsch, P.W.; Kim, B.; Yu., D.S. SAMHD1 Promotes DNA End Resection to Facilitate DNA Repair by Homologous Recombination. *Cell Rep.* **2017**, *20*(8), 1921–1935. https://doi.org/10.1016/j.celrep.2017.08.008
160. Akimova, E.; Gassner, F.J.; Schubert, M.; Rebhandl, S., Arzt, C., Rauscher, S.; Tober, V.; Zaborsky, N.; Greil, R.; Geisberger, R. SAMHD1 restrains aberrant nucleotide insertions at repair junctions generated by DNA end joining. *Nucleic Acids Res.* **2021**, *49*(5), 2598–2608. https://doi.org/10.1093/nar/gkab051
161. Li, W.; Xin, B.; Yan, J.; Wu, Y.; Hu, B.; Liu, L.; Wang, Y.; Ahn, J.; Skowronski, J.; Zhang, Z.; Wang, Y.; Wang, H. SAMHD1 Gene Mutations Are Associated with Cerebral Large –Artery Atherosclerosis. *Biomed Res. Int.* **2015**, *2015*, 739586. https://doi.org/10.1155/2015/739586

162. Brühl, J.; Trautwein, J.; Schäfer, A.; Linne, U.; Bouazoune, K. The DNA repair protein SHPRH is a nucleosome –stimulated ATPase and a nucleosome –E3 ubiquitin ligase. *Epigenet. Chromatin* **2019**, *12*(1), 52. https://doi.org/10.1186/s13072 –019 –0294 –5
163. Elserafy, M.; Abugable, A.A.; Atteya, R.; El –Khamisy, S.F. Rad5, HLTF, and SHPRH: A Fresh View of an Old Story. *Trends Genet.***2018**, *34*(8), 574–577. https://doi.org/10.1016/j.tig.2018.04.006
164. Seelinger, M.; Søgaard, C.K.; Otterlei, M. The Human RAD5 Homologs, HLTF and SHPRH, Have Separate Functions in DNA Damage Tolerance Dependent on The DNA Lesion Type. *Biomolecules* **2020**, *10*(3), 463. https://doi.org/10.3390/biom10030463
165. Lee, D.; Park, J.H.; Kim, S.; Lee, S.G.; Myung, K. SHPRH as a new player in ribosomal RNA transcription and its potential role in homeostasis of ribosomal DNA repeats. *Transcription* **2018**, *9*(3), 190–195. https://doi.org/10.1080/21541264.2017.1381795
166. Bae, N.S.; Baumann, P. A. RAP1/TRF2 complex inhibits nonhomologous end – joining at human telomeric DNA ends. *Mol. Cell* **2007**, *26*(3), 323–334. https://doi.org/10.1016/j.molcel.2007.03.023
167. Kotla, S.; Vu, H.T.; Ko, K.A.; Wang, Y.; Imanishi, M.; Heo, K.S.; Fujii, Y.; Thomas, T.N.; Gi, Y.J.; Mazhar, H.; Paez –Mayorga, J.; Shin, J.H.; Tao, Y.; Giancursio, C.J.; Medina, J.L.; Taunton, J.; Lusis, A.J.; Cooke, J.P.; Fujiwara, K.; Le, N.T.; Abe, J.I. Endothelial senescence is induced by phosphorylation and nuclear export of telomeric repeat binding factor 2 –interacting protein. *JCI Insight* **2019**, *4*(9), e124867. https://doi.org/10.1172/jci.insight.124867
168. Lebedeva, N.A.; Rechkunova, N.I.; Agama, K.; Pommier, Y.; Lavrik, O.I. Interaction of DNA topoisomerase 1 with DNA intermediates and proteins of base excision repair. *Biochemistry. Biokhimiia* **2009**, *74*(11), 1278–1284. https://doi.org/10.1134/s0006297909110157
169. Kim, N.; Jinks –Robertson, S. The Top1 paradox: Friend and foe of the eukaryotic genome. *DNA repair* **2017**, *56*, 33–41. https://doi.org/10.1016/j.dnarep.2017.06.005
170. Xu, Y.; Her, C. Inhibition of Topoisomerase (DNA) I (TOP1): DNA Damage Repair and Anticancer Therapy. *Biomolecules* **2015**, *5*(3), 1652–1670. https://doi.org/10.3390/biom5031652
171. Lee, K.Y.; Myung, K. PCNA modifications for regulation of post –replication repair pathways. *Mol. Cells* **2008**, *26*(1), 5–11.
172. Babatz, T.D.; Spear, E.D.; Xu, W.; Sun, O.L.; Nie, L.; Carpenter, E.P.; Michaelis, S. Site specificity determinants for prelamin A cleavage by the zinc metalloprotease ZMPSTE24. *J. Biol. Chem.* **2021**, *296*, 100165. https://doi.org/10.1074/jbc.RA120.015792

Table S6. Relationships between categorical characteristics of study subjects (gender, smoking status) and expression of genes selected from comparisons performed.

GENE NAME	GENDER	SMOKING STATUS	GENE NAME	GENDER	SMOKING STATUS
	P	*P*		*P*	*P*
ACTR2	0.627	0.445	*POLE*	0.799	0.979
AP5S1	1.000	0.114	*POLE3*	0.501	0.302
AP5Z1	0.105	0.254	*POLE4*	0.390	0.896
APBB1	0.835	**0.036**	***POLM***	**0.015**	1.000
ARID2	0.340	0.655	*PPP4R2*	0.729	0.937
ASF1A	0.295	0.445	*PRRX1*	0.167	0.618
ATM	0.871	0.278	*RAD17P1*	0.627	0.445
BCCIP	0.199	0.414	*RAD21*	0.835	0.158
CLK2	1.000	0.090	*RECQL*	0.253	0.142
DEK	0.472	0.328	*RFC1*	0.199	0.618
EGFR	0.982	0.090	*RTEL1*	0.799	0.384
EME2	0.295	0.511	*SAMHD1*	0.835	0.445
ERCC5	0.501	0.618	*SHPRH*	0.799	0.979
ERCC6L2	0.295	0.477	*SIRT7*	0.444	0.232
FANCB	0.390	0.694	*SMARCAD1*	0.627	0.733
FZR1	0.501	0.813	*SMC3*	0.417	0.813
GTF2I	0.594	0.618	*SPIDR*	0.835	0.302
HERC2	0.871	0.854	*TDP2*	0.127	0.414
HMGN1	0.390	0.618	*TERF2IP*	0.116	0.511
HSPA1A	0.627	0.937	*TOP1*	0.340	0.937
INIP	0.116	0.896	*TOP3A*	0.982	0.090
INTS3	0.594	0.581	*TREX2*	0.982	0.773
JMY	0.799	0.080	*TRRAP*	0.764	0.581
LIG1	0.253	0.232	*UBE2A*	0.562	0.356
MCM5	0.660	0.356	*UBE2V2*	0.501	0.854
MCM7	0.945	0.655	*UPF1*	0.764	0.694
MORF4L2	0.835	0.854	*USP1*	0.295	0.813
NBN	0.729	0.773	*USP10*	0.945	0.979
NFRKB	0.417	0.655	*USP51*	0.140	0.328
NIPBL	0.871	0.581	*UVSSA*	0.694	0.384
NPAS2	0.908	0.356	*VCP*	0.116	0.477
OGG1	0.472	0.477	*WHSC1*	0.835	0.854
PARP2	0.694	0.414	***XRCC6***	**0.023**	0.937
PDS5A	0.444	0.384	*ZBTB1*	0.105	0.212
PDS5B	0.660	0.979	*ZMPSTE24*	0.729	0.733
PNKP	0.694	0.813			

P – *p* value of statistical significance obtained from two –sided Mann–Whitney U adjusted by Benjamini – Hochberg False Discovery Rate.

Table S7. Correlation analysis between characteristics (age and BMI) of studied groups and expression of genes selected from all comparisons performed.

Gene symbol	AGE		BMI		Gene symbol	AGE		BMI	
	R	*p*	R	*p*		R	*p*	R	*p*
ACTR2	0.251	0.293	–0.149	0.554	*POLE*	0.174	0.480	–0.123	0.631
AP5S1	0.371	0.107	–0.245	0.306	*POLE3*	–0.110	0.667	–0.069	0.787
AP5Z1	0.215	0.373	–0.139	0.582	*POLE4*	0.580	**0.009**	0.450	**0.047**
APBB1	–0.590	0.007	0.467	**0.038**	*POLM*	–0.374	0.104	0.247	0.304
ARID2	–0.069	0.788	–0.052	0.840	*PPP4R2*	–0.118	0.643	–0.084	0.746
ASF1A	–0.423	0.063	0.283	0.232	*PRRX1*	–0.510	**0.023**	–0.395	0.084
ATM	–0.653	**0.002**	–0.519	0.021	*RAD17P1*	–0.077	0.766	–0.059	0.821
BCCIP	–0.034	0.894	0.035	0.892	*RAD21*	–0.390	0.089	0.270	0.256
CLK2	–0.059	0.818	–0.049	0.848	*RECQL*	0.118	0.643	0.077	0.766
DEK	–0.131	0.603	–0.085	0.742	*RFC1*	0.077	0.766	–0.054	0.834
EGFR	–0.357	0.122	–0.237	0.324	*RTEL1*	0.202	0.406	0.138	0.585
EME2	0.201	0.409	0.135	0.592	*SAMHD1*	0.226	0.349	0.148	0.555
ERCC5	–0.546	**0.014**	–0.413	0.070	*SHPRH*	–0.216	0.372	–0.141	0.577
ERCC6L2	–0.500	**0.026**	–0.363	0.116	*SIRT7*	0.499	**0.026**	0.355	0.125
FANCB	0.051	0.842	0.035	0.891	*SMARCAD1*	–0.424	0.062	0.306	0.192
FZR1	0.096	0.714	0.065	0.800	*SMC3*	–0.143	0.572	0.093	0.722
GTF2I	–0.056	0.827	0.048	0.852	*SPIDR*	0.381	0.097	–0.255	0.285
HERC2	0.194	0.430	0.129	0.611	*TDP2*	–0.219	0.365	0.146	0.561
HMGN1	–0.189	0.441	–0.125	0.624	*TERF2IP*	0.020	0.940	–0.008	0.977
HSPA1A	0.088	0.736	0.064	0.806	*TOP1*	0.335	0.149	–0.199	0.413
INIP	0.424	0.063	0.293	0.214	*TOP3A*	0.579	**0.009**	–0.443	0.052
INTS3	0.426	0.061	–0.321	0.169	*TREX2*	0.000	0.999	0.006	0.982
JMY	–0.490	**0.030**	–0.345	0.136	*TRRAP*	0.193	0.431	0.127	0.616
LIG1	–0.461	0.042	0.337	0.147	*UBE2A*	0.105	0.685	–0.065	0.800
MCM5	0.027	0.917	0.027	0.918	*UBE2V2*	0.148	0.556	–0.095	0.715
MCM7	–0.137	0.588	–0.089	0.734	*UPF1*	0.558	**0.012**	0.432	0.058
MORF4L2	–0.302	0.199	0.189	0.441	*USP1*	–0.384	0.094	0.258	0.279
NBN	–0.371	0.107	–0.239	0.320	*USP10*	0.357	0.122	0.233	0.336
NFRKB	0.163	0.511	0.119	0.640	*USP51*	–0.672	**0.002**	–0.533	**0.017**
NIPBL	–0.289	0.222	0.180	0.464	*UVSSA*	0.259	0.277	0.159	0.525
NPAS2	–0.456	**0.044**	–0.332	0.153	*VCP*	0.619	**0.004**	–0.469	**0.037**
OGG1	0.324	0.164	0.192	0.433	*WHSC1*	0.479	**0.034**	–0.343	0.138
PARP2	–0.411	0.072	0.272	0.252	*XRCC6*	0.085	0.745	–0.061	0.812
PDS5A	–0.278	0.242	–0.174	0.478	*ZBTB1*	–0.269	0.259	–0.166	0.503
PDS5B	–0.284	0.230	0.178	0.467	*ZMPSTE24*	0.114	0.654	0.070	0.786
PNKP	0.196	0.422	–0.132	0.602					

R – Spearman correlation coefficient, BMI – body mass index, *p* – *p* value of statistical significance adjusted by Benjamini – Hochberg FDR.

SUPPLEMENTARY TEXT

Current publication focuses on transcriptomic analysis of DNA repair and other interconnected processes in abdominal aortic aneurysm (AAA), chronic venous disease (CVD) and lower extremities arterial disease (LEAD). Analysis constitutes 519 gene whose expression account for DNA repair, nucleotide pool sanitization and translesion synthesis. Moreover, substantial number of genes determined as indicative in analysed diseases is related to various regulatory processes regarding DNA repair. In supplementary text further introductory comments on DNA damage repair, interstrand crosslinks repair, DNA damage tolerance and DNA damage prevention were placed (section 1). In section 2 discussion on genes with regulatory function in DNA damage repair, which were indicated in presented research as having potential for contribution to analysed vascular diseases is explored.

1. Brief description of DNA Damage Metabolism Pathways

1.2. DNA Damage Repair (DDR)

1.2.1. Direct and indirect repair of DNA base damage

In DDR we can point two main groups of DNA repair modes: direct repair of DNA base adducts and indirect repair by base or whole nucleotide removal.
First of mechanisms – direct reversal of adducted DNA bases consist of enzymatic reactions removing harmful chemical group(s) from the base without the need of excision and subsequent recreation of undamaged DNA strand. This applies to various alkylated adducts and is conducted by either transfer of alkyl group by 'suicidal protein' O^6–Methylguanine–DNA Methyltransferase (MGMT) [1] or by oxidative dealkylation by AlkB homologs [2]. Another mechanism is photochemical decomposition of UV–light induced pyrimidine dimers by photolyases. Those proteins are absent in humans [3].

Mismatch repair (MMR), base excision repair (BER) and nucleotide excision repair (NER) all represent the indirect repair mechanism.

MMR engages several proteins with both regulatory and core–enzymatic functions, including some components of the DNA replication system. MMR corrects mainly DNA bases mispaired during replication, which were able to escape the proofreading activity of DNA replicative polymerases. Moreover MMR machinery is able to detect and repair mismatches resulting from recombination, branching secondary DNA structures and chemically modified, bulky DNA adducts [4].

BER system consists of DNA glycosylases, removing damaged base and at least thirty other accessory proteins, some of them taking direct part in subsequent enzymatic reactions conducted on DNA (like AP endonuclease – APE1, polynucleotide kinase phosphatase – PNPK or DNA polymerase β) while others regulating repair functions of whole complex (like poly(ADP–ribose) polymerase 1 – PARP1). Oxidative lesions and alkylation adducts as

well as DNA bases hydrolysis products (abasic sites) and single – strand breaks could be classified as BER substrates [5

NER is a major DNA repair and a multi–step process of removal of various DNA helix–distorting lesions or structures, whether they are generated through environmental mutagens, irradiation or bulky chemical compounds. Global Genome NER and Transcription Coupled NER could be recognized [6,7].

1.2.2. Interstrand crosslinks (ICLs) repair

ICLs are highly cytotoxic lesions caused by covalent bonding of complementary DNA strands by substances like cancer chemotherapeutics, alcohol metabolites, cigarette smoke, acetaldehyde and malondialdehyde. Being an obstacle to replication and transcription they may induce mutations, chromosome breakage, chromosome missegregation and mitotic catastrophe. FA/BRCA repair pathway is dedicated to removal of such DNA damage. It engages 22 genes termed complementation groups FANCA-FANCW, which along with accessory proteins governs initial steps of crosslink repair. Final steps are completed in cooperation with Trans Lesion Synthesis (TLS) and Homologous Recombination (HR) [8,9].

1.2.3. DNA Double Strand Breaks (DSBs) Repair

Unlicensed double strands breaks of DNA caused by DNA damaging agents are undoubtedly the most toxic kind of damage, that can occur [10,11]. This kind of damage is proceeded by two major repair mechanisms: Homologous Recombination (HR) and Non–Homologous End Joining (NHEJ).

HR repairs a variety of DNA lesions, including DSBs, single–strand DNA gaps and interstrand crosslinks [11]. HR process may utilize couple subpathways one of which is synthesis–dependent strand annealing (SDSA), believed to be the predominant HR mode for DSB repair. Another one includes formation of a secondary DNA structure consisting of a four–way junction between the recombining DNA strands (Holliday Junction – HJ), later dissolved by BTR complex consisting of Bloom helicase (BLM), Topoisomerase 3A and other proteins. Both subpathways are believed to be error–free [10,12].

NHEJ is active during whole cell cycle, not to be constrained to only S and G_2 phases like HR. It is responsible for direct religation of almost all DSBs during whole cell cycle and remains the main repair system for DSBs. Non–homologous in name may be misleading, however NHEJ could utilize up to 4 bp of microhomology during repair. Action of this repair pathway requires multiprotein complexes with nuclease, kinase and ligase activity, as well as contribution of polymerases. DSBs of various characteristics like blunt ends, 5′ and 3′ overhangs, gaps, loops, bubbles, hairpins, 5′ end lacking a phosphate, 3′ end phosphates, 3′–phosphoglycolates, 3′–Topoisomerse 1 crosslinks may be processed by NHEJ machinery [13,14].

1.2.4.DNA Damage Tolerance (DDT)

Although not engaging enzymes directly acting on damaged DNA bases, DNA damage tolerance and prevention fulfill the picture of possible mechanisms assuring the genome stability, hence the term DNA damage metabolism instead solely DNA damage repair was used in the text and the title.

DNA replication process is under constant threat of aberrant function or even permanent stalling due to various DNA lesions, secondary DNA structures and rare and difficult to replicate sequences [15]. To overcome such obstacles organisms not only have evolved means of removal of such DNA alterations (NER and BER) but also an alternative system of DNA Damage Tolerance. It is composed of two different routes: Translesion synthesis (TLS) and Template Switching (TS), first of them being error–prone and the second one error–free [16]. Low fidelity and processivity polymerases are the main enzymes engaged in TLS. Those enzymes are specialized and unique, capable of supporting stalled replication forks and replicate through distorted DNA structures. This is possible due to their ability to accommodate altered DNA bases to their unexceptionally spacious active site. Since TLS polymerases do not possess proofreading activity, their action is error–prone and requires strict control [17]. In humans we can distinguish pol η, pol ι, pol κ, Rev1, (Y–family DNA polymerases) and pol ζ (B–family DNA polymerases), being the principle TLS polymerases [15].

TS uses newly synthesized sister chromatid as a template for lesion bypass. It is triggered by replication fork pausing what leads to disengagement of the lagging strand and invasion of an adjacent replication fork through the annealing of microhomologies [16, 18, 19]

1.2.5. DNA Damage Prevention (DDP)

The formation of damaged bases in DNA double helix is well recognized source of mutation. In addition, DNA precursor pool (2′–deoxyribonucleoside 5′–triphosphates) is also subjected to various chemical reactions. ROS predominantly cause formation of 8–hydroxy–2′–deoxyguanosine 5′–triphosphate (8–OH–dGTP) 2–hydroxy–2′–deoxyadenosine 5′–triphosphate (2–OH–dATP) and 8–hydroxy–2′–deoxyadenosine 5′–triphosphate (8–OH–dATP) [20]. Those may be incorporated into DNA strand by most of DNA polymerases [21]. In order to prevent such events enzymes of high selectivity conducts reactions of hydrolysis of DNA base derivatives triphosphates to corresponding monophosphates and pyrophosphate [20, 21]. In mammals three enzymes bearing such activities have been found: MTH1 (NUDT1) and MTH2 (NUDT15) hydrolysing 8–OH–dGTP, NUDT5 hydrolysing 8–oxo–7,8–dihydro–2′–deoxyguanosine 5′–diphosphate and MTH1 (NUDT1) hydrolysing 2–OH–dATP and 8–OH–dATP [20].

2 Supplementary comments and discussion

When describing DNA repair and related processes it is of the uttermost importance to be aware, that one can encounter numerous phenomena, where DNA repair machinery elements have also a common physiological function(s). Moreover, there are many genes possessing multiple tasks, engaged in course of many processes. That complicates the view of given network of interactions and requires a thorough analysis. All additional information regarding presented genes are placed in supplementary table S5.

2.1. *Cohesins could be engaged in pathological processes in AAA*

RAD21 encodes essential and evolutionarily conserved protein belonging to the cohesin family and constituting one of four (along with SMC1A, SMC3 and STAG) core cohesin complex subunits [21]. This protein is present in all eukaryotes, bearing various functions like involvement in sister chromatid cohesion, chromosome segregation, post–replicative DNA damage repair, and prevention of unlicensed recombination [22,23]. Although being the most frequently occurring differentially expressed gene through all comparisons, *RAD21* exhibits dissimilar pattern of expression with decrease in AAA vs control and CVD vs control groups and increase in LEAD vs AAA and LEAD vs CVD groups. Surprisingly, it corresponds in many details with expression of the second cohesin complex element (*SMC3*) [21] and cohesin–related genes like *NIPBL*– encoding cohesin loading factor [24], and *PDS5A/B* which provides cohesin complex maintenance [25]. Moreover products of those two genes are also responsible for fine regulation of the cohesin complex. Those similarities are especially prominent when it comes to decrease in expression of cohesin genes in AAA vs control group. This could be indicative for different modes of action against DNA damage depending on disease, thus being a good distinctive feature, useful in diagnostics and potential treatment.

2.2. *Expression of DNA Double Strand Break Repair genes is altered in vascular diseases.*

Due to their detrimental effects and toxicity, double strand breaks require rapid detection, safeguarding and prompt repair. This forces mutual action of many proteins. Repair process needs not solely direct enzymatic activities processing damaged DNA, but it is also highly regulated by accessory proteins [26,27].

Three genes encoding products possessing various enzymatic activities needed in HR were shown to be differentially expressed and characteristic for multiple comparisons, namely: *PNKP, POLE, UPF1*. PNPK is a polynucleotide kinase 3'–phosphatase engaged in processing of free DNA ends [28] enabling ligation of DNA strand breaks in both nuclear and mitochondrial DNA [29]. POLE is a catalytic subunit of polymerase epsilon complex, engaged in sensing and resection of DNA in DSBs, leading –strand DNA replication in yeast [30,31]. UPF1 is a DNA/RNA helicase functioning also in resection of DNA in DSBs [32]. Interestingly, these genes (*PNKP, POLE, UPF1*) have strikingly similar pattern of expression being upregulated in AAA and CVD groups in comparison with controls, suggesting their functional dependency. On contrary to this observation *PNKP* has decreased expression in only LEAD vs AAA and LEAD vs CVD while *POLE* and *UPF1* expression remains unchanged. REQL is a helicase, preventing replication fork collapse during replication stress, what is needed for genomic stability [33,34]. On contrary to *PNKP, POLE* and *UPF1*, *REQL* has been found as upregulated only in LEAD vs CVD.

Second group of genes engaged in DSB repair is composed of genes encoding proteins engaged mainly in DNA binding with less consistent expression changes through compared groups. It includes: *XRCC6 (Ku70)*, *EGFR*, *INIP (SSBIP1)* and *INTS3*. Primary functions of Ku proteins are: recognition of free DNA ends, binding to them with high affinity, safeguarding them from degradation and subsequent recruitment of other factors needed for DSB repair [35,36]. EGFR is mainly known to be a signal transducer regulating not only cell growth, but also DNA repair [37,38], with ability of nuclear migration and direct DNA binding facilitating DSB repair induced by ionizing radiation [39]. SOSS1 is a single–stranded DNA (ssDNA)–binding protein complex composed of Integrator subunit 3 (INTS3), single–stranded DNA–binding protein 1(SSB1), and SSB–interacting protein 1 SSBIP1 (INIP). INTS3 has scaffolding function stabilizing this complex. Those proteins play a critical role in efficient HR–dependent repair of DSBs and ATM–dependent signaling pathways [40,41]. Decreased expression was observed in case of *XRCC6* (CVD vs Control) and *EGFR* (AAA vs control and CVD vs control), whereas increased expression was characteristic for: *XRCC6* and *INIP* in LEAD vs CVD as well as in pooled group LEAD and AAA vs CVD. *INTS* had elevated expression exclusively in AAA vs Control group comparison. It is tempting to assume, that revealed dependencies in expression changes could be indicative for diverse regulation of the same process in various diseases, creating the possibility of targeted therapies and diagnosis.

Third group of genes consists of genes responsible for regulation of DSB repair process. It includes: *PARP2, SMARCAD1, ARID2 (Baf200)* and *USP1* (in set of genes common for at least two comparisons) and *AP5S1, APBB1 (FE65), ASF1A, SIRT7, SPIDR, USP51, VCP (p97)* (unique for AAA vs Control group comparison) and *SAMHD1* exclusive for LEAD vs CVD comparison.

PARP proteins modify histones and many other substrates with mono– and poly(ADP–ribose) what is important for subsequent chromatin decompaction and additional repair proteins recruitment [42,43]. PARP2 is predominantly activated by DNA gaps, flaps and recombination intermediates [44] and readily recruited to DSB [42]. Moreover, it is known that PARP–2 is a component of a functional BER complex *in vivo* [45]. Decreased expression of this gene was characteristic only for AAA vs control and CVD vs control comparisons.

USP1 is a deubiquitinase removing ubiquitin from FANCD2 resulting in inactivation of the FA pathway [46] and promotes homologous recombination repair [47]. It also removes monoubiquitin modification of PCNA, depleting activity of the TLS DNA damage tolerance pathway [48]. Expression of this gene was decreased in AAA vs control and CVD vs control and in 'all three disease vs control' group. Decrease in expression of USP1 may lead to prevalence of monoubiquitinated forms of FANCD2 and PCNA leading to aberrant action of both Fanconi Anemia pathway and TLS [49], thus aggraviating DNA damage capabilities in AAA and CVD patients.

SMARCAD1 [50,51] and ARID2 [52,53] are responsible for chromatin remodelling in DNA damage response including DSB repair, what will be discussed further in the text (section 3.3.).

AAA vs Control group comparison was the richest in uniquely differentially expressed genes (eighteen positions). Among them eight genes were ascribed to regulation of DSBs repair process on the basis of cited literature (*AP5S1, APBB1, ASF1A, SIRT7, SPIDR, USP51, VCP*).

AP5S1 has been reported to have DNA helicase function, and has been associated with a protein complex, which is required for DNA double–strand break homologous recombinational repair [54]. APBB1 (FE65) modulates the DSBs repair pathway by regulating the acetylation of histone H4 at DNA strand breaks that induces recruitment of Tip60 to DNA damage sites [55]. Similar histon–modyfing activity possess ASF1A, being a histon H3 demethylase [56]. ASF1A inhibition induces p53–dependent growth arrest and senescence of cancer cells [57]. Decrease in matching genes expression may be responsible for impaired strand break repair and inducing senescence in patients' cells, potentially aggravating course of the AAA. Correspondingly, SIRT7 is a histone H3 desuccinilase (at lysine 122) and deacetylase (at lysine 18) promoting genomic stability and DNA repair via site–specific deacetylation of a damage–associated histone mark, H3K18Ac [58,59]. Enzymatic specifities of SIRT7, potential substrates and epigenetic activity not only do ameliorate many effects of stress and

senescence but also increase in activity of this gene points to the increased DSB repair [60] suggesting an alleviating action in AAA patients.

SPIDR encodes a scaffold protein, important element of SWS1–SWSAP1–SPIDR [61] and Shu (in concert with PDS5B) [62] complexes, whose one of functions is promotion of homologous recombination. Elevated expression of *SPIDR* in concordance with downregulation of PDS5B may indicate fine tuning of regulation of homologous recombination in AAA patients, allowing to distinguishing them from other conditions subjects.

USP51 encodes deubiquitinase engaged in removal of ubiquitin from histone H2A (lysine 13 and 15). Depletion of USP1 impairs cell viability and repair efficiency. It also facilitates the formation of H2AK15ub and 53BP1 recruitment, promoting NHEJ pathway of DSB repair [63,64]. This suggests, that in AAA patients a crosstalk between NHEJ and HR could be present due to a decrease in expression of *USP51*.

VCP (p97) is a protein interacting with MRE11–RAD50–NBS1 (MRN) complex regulating its function. Depletion of VCP leads to defective DNA repair and radiosensitivity [65]. It has been suggested that it is required in proteasomal degradation of topoisomerase 2–DNA covalent complexes [66]. Interestingly, VCP has also important function in angiotensin II signal transduction in Vascular Smooth Muscle Cells (VSMCs) [67]. Angiotensin II may induce senescence by promoting ROS generation in VSMCs from AAA tissue [68]. This could negatively contribute to general state of AAA patients enroled to this research.

SAMHD1 encodes enzyme with dNTP triphosphohydrolase activity, balancing the deoxynucleotide pool throughout the cell cycle [69]. This protein has a second function: facilitating DNA DSBs repair by homologous recombination through promotion of DNA ends resection [70]. Mutated gene was found in Aicardi–Goutières syndrome – condition mimicking congenital virus infection with presentations of inflammatory responses similar to systemic lupus erythromatosus [71]. Its expression is upregulated in LEAD vs CVD patients, suggesting not only distinct regulation of homologous recombination but also be one of potential elements inducing inflammation in LEAD and CVD.

There were other genes present in sets common for at least two comparisons (*BCCIP*, *LIG1*) and unique for only one comparison (*RTEL1*, *TOP3A*, *EME2*, *FANCB*) contributing to homologous recombination, according to cited literature.

BCCIP is an interactor of RAD51 and BRCA2 important in HR [72], while LIG1 is the main replication ligase taking part in rejoining of DNA strands as the last step of many DNA repair pathways [73]. Both genes possess unpatterned expression with *LIG1* underexpression in AAA vs CVD and pooled LEAD and AAA vs CVD groups and *BCCIP* underexpressed in CVD vs control and overexpressed in LEAD vs CVD groups.

Both *RTEL1* and *TOP3A* are overexpressed in AAA patients. RTEL1 is a regulator of HR implicated in meiotic cross–over control as an antirecombinase and in DNA repair in a worm *Caenorhabditis elegans*. It has a DNA helicase activity. [74,75]. Topoisomerase 3 A (encoded by *TOP3A* gene) works in concert with other proteins governing many aspects of genome maintenance, including DNA replication, DNA repair, telomere maintenance, and meiosis [76], but in context of this research the most important function is probably its ability to resolve secondary DNA structures occuring in HR, like Holliday Junctions [77]. Increased expression of those genes suggests pronounced presence of HR in AAA patients.

Both *EME2* and *FANCB* are overexpressed, but indicative separately for CVD and LEAD vs AAA, respectively. EME2 associated with MUS81 complex acts like a DNA structure–specific nuclease resolving Holliday junctions and other substrates occuring during HR repair [78,79]. Conversely, despite FANCB contribution to HR repair of DNA damage [80 it acts independently of MUS81 complex [81]. This distinctive feature implicates different regulatory pathways of HR in CVD, LEAD and AAA what could be utilized for discriminative diagnosis and treatment of CVD, AAA and LEAD.

The composition of transcriptional patterns points to homologous repair as the main DSBs repair mechanism predominant in AAA, but occuring also in both LEAD and CVD. Moreover, work by Miner *et al.* identified loci though to be specifically associated with not only thoracic aortic wall defects and abdominal aortic aneurysms, but also DSBs repair [82].

To further support engagement of homologous repair of DSB in presented diseases, non–homologous end joining–related genes (*POLM, ASF1A, ERCC6L2, USP51*) transcription was examined.

POL M (POL iota, Pol mu) encoded by *POLM* gene has an important role in DNA repair, being engaged in NHEJ and interacting with the other proteins of the NHEJ machinery [83]. It was confirmed in eukaryotic cells model, that ASF1A knockout reduces NHEJ and increases HR repair [84].

Recent studies suggest ERCC6L2 of having function as HR antagonist and loss of it restores HR in BRCA1–deficient cells [85]. USP51 is a histone H2A deubiquitinase whose depletion results in impairment of DNA damage response [63] while its knockdown decreases HR and NHEJ efficiency in LM2–DRR cells [86]. All NHEJ–related gene transcription was decreased suggesting shift to the HR as main mechanism of DNA DSBs in AAA patients.

Interestingly, genes identified by current analysis were engeged more in different modes of DSBs repair regulation than in specific enzymatic activities. This allows to speculate, that the regulation of basic processes of DNA repair could be characteristic for given vascular disease, reflecting different onset, progression and specific presentations, while the main enzymatic activities proceeding HR remain unaltered. This could be a prerequisite for targeted treatment and/or discriminative diagnosis. Even though counterintuitive may it seem, elevated activity of HR may promote excess in DNA damage due to rise in free DNA ends resection thus causing increase in DNA damage intermediates, known to cause DNA damage when urepaired [87,88]. It is in line with the fact, that intensified DNA damage may aggravate presentations of vascular diseases.

2.3. Chromatin Remodelling could be the factor shaping and regulating DNA damage responses in vascular diseases.

DNA in Eukaryotes tightly interacts with many specialised proteins, like histones, building a highly organised entity called chromatin. On one hand enabling packaging of whole DNA into the nucleus, this sophisticated structure requires also pronounced changes during life of the cell in order to meet the requirements of DNA replication, transcription, RNA maturation and DNA damage repair [89]. It should be remodelled to facilitate the recruitment and action of proteins and other factors governing those processes. Complex and diverse chromatin organization facilitates some processes like DSBs repair but may also pose an impediment for detection and repair of DNA lesions [90]. Thus changes in chromatin structure and nucleosome modifications are prerequisite to the fine–tuning in the course of various metabolic activities.

Current NGS analysis did detect some of differentially expressed genes, whose products are needed in various chromatin remodelling processes. In group of genes ascribed to more than one comparison those were: *ATM, POLE* and *POLE4, WHSC1 (NDS2), SMARCAD1, ARID2 (Baf200), HMGN1.*

ATM is a serine/threonine kinase, stimulated in response to DNA damage [91]. It is the node factor in a vast biochemical network of reciprocal interactions signalling the presence of DNA damage and controlling the repair process. Its function is especially prominent in DNA double–strand break response and p53 signalling [92]. Despite important function of ATM, *ATM* has ambigous expression pattern being decreased in LEAD and AAA patients when compared to control group and in

comparisons regarding LEAD vs CVD and pooled comparison LEAD and AAA vs CVD, suggesting that its impaired expression may be indicative for arterial diseases, but not for venous ones, additionally signalling potential decrease in DNA repair capacity in those patients.

DNA polymerase ε (Polε) is a complex consisting of four subunits with the major task of DNA leading strand synthesis in eukaryotes where POLE1(POLE catalytic subunit) possess polymerase and exonuclease activities and POLE2 structurally links Polε to the CMG complex [93]. Recently, it was discovered that two of subunits (POLE3 and POLE4) are histone H3–H4 chaperones maintaining chromatin integrity during DNA replication [94]. All three subunits' genes have different pattern of expresion across analyzed comparisons. *POLE* was upregulated in AAA and CVD and in pooled disease group in comparison with controls, while *POLE4* was upregulated in LEAD vs CVD and AAA vs CVD comparsion as well as in LEAD and AAA vs CVD pooled groups comparison. Upregulated expression of *POLE3* occurred in LEAD vs AAA. This may indicate, that expression of different subunits of Polε could be regulated in distinct way in various vascular conditions.

WHSC1 (NDS2) is a histone methyltransferase dimethylating lysine 36 on the H3 histone (H3K36me2), which activates transcription [95]. This protein facilitates alveolar macrophage pyroptosis through inflammosome activation [96]. Moreover it promotes cell proliferation and migration in colon and hepatic cancers [97, 98]. Consequently, WHSC1 may have stimulating effect on vascular wall remodelling by enhancing inflammation and increasing cells motility in AAA and CVD patients.

SMARCAD1 encodes protein which is an actin–dependent regulator of chromatin, implemented in homologous recombination, active during end–resection on double strand breaks. It is also a substrate of ATM kinase [50,99].

ARID2 is a defining subunit of PBAF (polybromo–associated BAF) complex belonging to the Swi/Snf family of chromatin remodelling complexes. It contributes to DNA damage–induced nucleosome remodelling especially in vicinity of DNA DSBs [52]. It is required in mouse embryonic cardiomyocyte proliferation as well as in lineage conversion of venous cells into arterial endothelial cells during coronary development [100]. Disregulated expression of *ARID2* may thus be an element of endothelial cell pathology in CVD (enhanced expression) while reduced expression may be one of elements establishing differences between LEAD vs CVD.

HMGN1 belongs to the family of proteins interacting with histones in 147–bp nucleosome core particle in purely structure–dependent manner [101]. This protein is coacting in repair of ultraviolet light and ionizing irradiation induced DNA damage, having contribution to Global Genome Repair, Transcription Coupled repair and priming chromatin for NER [102,103]. It is also a substrate for ATM kinase [91]. *HMGN1* was downregulated in CVD and was indicated as downregulated in LEAD, CVD and AAA vs control group comparison what bespeaks for *HMGN1* disregulation as a disease correlated factor in CVD and other analyzed diseases.

ACTR2 (ARP2) in concert with ARP3 was proposed to cluster DNA damage site into large foci in euchromatin and migrate heterochromatin DSBs to sites in nucleus, where homologous recombination is carried out. Movement is enabled by assembling of nuclear actin filaments [104,105]. Interestingly, chromatin remodelling activity of many chromatin/histone remodelling complexes (like SWI/SNF (or BAF), INO80, SRCAP/SWR1, TIP60/NuA4) is dependent on actin metabolism [105]. Upregulation of this gene was present in LEAD in comparison with CVD group of patients.

NFRKB is a subunit of INO80 ATP–dependent nucleosome/chromatin remodelling complex. Action of this complex may regulate transcription or DNA repair [106]. Although TRRAP shares many similarities with Phosphoinositide 3–Kinase–related kinase, it is devoid of any kinase activity. Despite that it is speculated to possess scaffolding function instead, connecting transcription factors and

chromatin modifying complexes [107] what is important in many chromatin–related processes like remodelling, transcription and DNA repair. Indeed, it was found in the MRN complex (MRE11–RAD50–NBS1) involved in DNA DSB repair [108]. Both *NFRKB* and *TRRAP* gene were upregulated exclusively in CVD patients. Dysregulation of *APBB1 (FE65), ASF1A, ERCC6L2, SIRT7, USP51* was characteristic for AAA patients and has been described in section 3.2.

Differential expression of genes related to chromatin remodelling clearly indicates important contribution of this process to regulation of DNA damage in presented vascular diseases. What is interesting, it could be assumed, that, similarly to DNA DSBs repair, the regulation of various chromatin–related processes is important factor to distinguish between vascular diseases. Those differences could have a tremendous impact on disease course and presentation.

2.4. *Specific gene expression changes may be indicative for altered oxidative DNA damage responses in AAA, CVD and LEAD.*

Reactive Oxygen Species (ROS) generated during metabolism have many adverse effects including not only oxidative DNA damage, genomic instability, carcinogenesis and cellular ageing but also oxidative modifications of other cell constituents like proteins and lipids [109,110]. It is widely accepted, that pro–inflammatory signalling activation, expression of specific cytokines/chemokines and increased oxidative stress triggering oxidative modification of Low Density Lipoproteins and endothelial cells activation are all main factors leading to atherosclerosis [150]. ROS are capable to influence endothelial cells by promoting apoptosis and necrosis what subsequently leads to thrombosis and atherosclerotic plaque rupture. Oxidative stress is both the crucial hallmark of cardiovascular diseases and their causative factor [111]. Analysis of indicative genes, revealed in presented research, confirmed and broadened the knowledge of oxidative stress in AAA, CVD and LEAD.

MRGX is a transcription factor belonging to mortality factor 4 (MORF4)–related gene family [112]. It associates with TRRAP/TIP60 histone acyltransferase complex [113]. It was indicated as upregulated in bovine embryos subjected to oxidative stress [114], suggesting its role in defence against ROS. Decrease of *MORF4L2 (MRGX)* transcription was observed in AAA and CVD patients what may indicate impaired response to elevated oxidative stress.

ERCC5 (XPG) encodes a protein which is one of multiple proteins that are indispensable for NER of DNA lesions in mammalian cells [115]. It is a single stranded DNA–specific endonuclease cleaving at 3′ end of the DNA lesion. Moreover it has many non–enzymatic and regulatory functions like direct interaction with TFIIH what forms XPG–TFIIH complex acting as a transcription elongation factor for RNA Polymerase II. XPG in concert with XPF is persumed to be involved in chromatin looping. Moreover XPG interacts *in vitro* and stimulates the NTH1 glycosylase involved in BER of oxidative DNA base damage. Finally it participates in HR [116]. This gene was downregulated in AAA patients and it may be concluded, that it is a sign of pathology, because dysfunction of NER was correlated with age –related vascular dysfunction [117].

OGG1 is the main glycosylase removing the most abundand DNA lesion resulting from oxidation: 8–oxoG. But its function is not restrained to merely DNA repair, as it has pleiotropic functions in both cellular signalling and epigenetic–like pathways [118 - 120]. Although this protein has been implicated to have important modulatory effect restricting inflammatory responses and apoptosis in macrophages preventing atherosclerosis [121], it also may promote inflammation by its ability to bind to DNA and upregulate pro–inflammatory gene expression [120]. OGG1 was upregulated in AAA and CVD patients suggesting ambiguous effect: on one hand preventing adverse

effects caused by elevated ROS on the other hand stimulating inflammation by its non–enzymatic action.

In the context of oxidative stress ERCC6L2 could be also mentioned. ERCC6L2 knockdown induced intracellular ROS in eukaryotic cell lines [122] suggesting that along with downregulation of *ERCC6L2* there could be increase in ROS production in AAA patients.

ATM was described in Chromatin remodelling section,while *PNKP, XRCC6 (Ku60), VCP (p97)* in DSBs repair section. Analysis of transcriptional patterns of genes presented in this research is supported by findings of other groups, that oxidative stress is the prominent feature of many vascular diseases, including those in current assessment.

2.5. Ubiquitination and Deubiquitination could be a prominent mechanism regulating gene activities in vascular diseases.

One of potent mechanisms of protein function regulation is ubiquitination. Chemically, this process could be characterised as a covalent binding of ubiquitin protein to various other proteins. It is conducted by a cascade of reactions catalysed by three types of enzymes: Ub–activating enzyme (E1), Ub–conjugating enzyme (E2), and Ub–protein ligase (E3). Apart from being the key element in proteasomal degradation of targeted proteins, ubiquitination is now known to also control their function by monoubiquitination and different polyubiquitination modes [123]. Deubiquitinating enzymes (DUBs) are proteases acting in dual manner either removing ubiquitin from target proteins or remodelling ubiquitin chains on target proteins [123]. Both ubiquitination and deubiquitination may regulate various processes associated with DNA, including DNA repair [124,125].

UBE2A (RAD6A) encodes enzyme whose function is a monoubiquitination of PCNA and taking part in DNA damage response to ionizing radiation [126,127]. Interestingly it is also engaged in a process of deamidation of proteins by regulating human Nt–Asn–specific Nt–amidase (NTAN1), Nt–Gln–specific Nt–amidase (NTAQ1) and arginyltransferase (ATE1) [128]. Deamidation of lipocalin –2 has been correlated with endothelial dysfunction and hypertension in dietary obese mice [129] and with enhancement of monocyte adhesion to atherosclerotic matrix proteins when deamidated aminoacids occurred in specific protein motifs [130]. Upregulation of *UBE2A* may suggest involvement of deamidation process, that could be characteristic for LEAD in comparison with AAA and CVD.

USP10 and USP51 are deubiquitinases with increased expression in AAA patients. USP10 deubiquitinates and stabilizes MutS Homolog 2 (MSH2) which is the key DNA mismatch repair protein [131]. USP51 is a histone deubiquitinase and has already been commented in section 2.2.

ZBTB1 is a pertinent upstream regulator of TLS. It regulates activity of RAD18, monoubiquitinating Proliferating Cell Nuclear Antigen (PCNA) at DNA damage sites what in turn recruits TLS polymerase, such as Pol κ or Pol η [132]. Decreased expression of ZBTB1 may be indicartive for rerouting of TLS pathways into DNA repair processes in AAA patients.

HERC2 is an E3 ligase managing BRCA1 and XPA degradation, thus negatively regulating HR and NER, respectively [133,134]. Upregulation of this gene is present in CVD patients, suggesting that ubiquitination may be the process responsible, at least to some extent, for characteristic disease presentations.

SHPRH belongs to the SWI/SNF family of AT–Pases [135] presenting enzymatic preference for nucleosomes with extranucleosomal DNA. It has also a nucleosome–E3 ubiquitin ligase activity [136]. It contributes to DNA damage tolerance (DDT) or post–replication repair (PRR) pathways and is important for avoiding of DNA strand breaks [136,137]. It is the only gene, whose expression was

lowered in LEAD patients in comparison with CVD ones. It may suggest that it is an element of disregulation of DNA DSBs repair, differentiating LEAD and CVD.

UBE2V2 (MMS2) is a ubiquitin–conjugating enzyme, acting with UBE2N in dimer to form K63–linked ubiquitin chains on protein substrates [138]. Moreover it is a DNA damage response regulator polyubiquitinating monoubiquitinated PCNA, thus promoting error–free damage avoidance pathway [139]. Interestingly, it is a crucial element of crosstalk between Redox and Ubiquitin regulation, capable of detecting ROS and coordinating DNA damage responses [140]. Increased expression of this gene may be thus indicative for regulation of oxidative stress response in LEAD patients when compared to CVD ones.

Presented results shows connection between DNA damage responses and ubiquitin metabolism, showing possible different modes of regulation of DNA damage. Moreover those seem to be specific for presented diseases, having potential value in both specific diagnosis and treatment.

2.6. *Analysis reveals known genes, being involved in vascular diseases initiation and progression.*

Cardiovascular diseases have long been described as 'multifactorial' [141]. Multifactorial diseases involve plethora of genetic and environmental factors contributing to overall disease outcome. Due to this fact, the genetic component of the disease is either not known or its effects are blurred. Even though, some genes indeed has already been correlated with various aspects of vascular diseases. Current Next Generation Sequencing analysis reveals a significant amount of those genes, whose deregulation was seen in many vascular disease–related experiments and assessments. Their contribution varies from regulation of vascular specific process to commitment to a specific risk factor or cardiovascular system defects. Transcriptional pattern is also variable ranging from five comparison occurrences (*RAD21*) to exclusiveness for only one comparison.

RAD21 has established role in signal transduction from vascular endothelial growth factor (VEGF) receptors, playing important role in atherogenesis [142].

DEK encodes an ubiquitous protein associated with chromatin during the entire cell cycle. It preferably binds to supercoiled and four–way junction DNA [143]. The protein promotes also skeletal muscle stem cell activation via intron removal during the quiescence exit [144]. It could be speculated, that similar mechanism is engaged in vascular smooth muscle cells activation, a process common for many vascular conditions [145].

WHSC1 is a candidate gene, whose haploinsufficiency was correlated with congenital heart disease [146].

JMY is a multi–role transcription factor affecting cell adhesion and coordinates cell adhesion and motility with p53 action in response to DNA damage by actin nucleation [147].

MORF4L2 (MRGX) decreased levels may promote unstable plaque through telomere dysfunction, what is a symptom of cellular damage and senescence [148]. *MORF4L2* analysis may thus improve the diagnosis, therapy and help to monitor condition of unstable plaque patients.

OGG1 activity may prevent inflammation and atherosclerosis through downregulating excessive inflammasome activation in mice [121]. This gene was correlated with other cardiovascular risk factors. It turned out, that levels of gluconeogenesis, thus hyperglycemia were elevated under fasting conditions in mice lacking OGG1 [149]. Moreover mitochondrial form of OGG1 was engaged in reduction of bodyweight and reduction of adipose tissue [150]. In the light of those facts, elevated expression of OGG1 in AAA and CVD may be ambiguous: on one hand being a factor that alleviates disease, on the other signaling excess of ROS presence and oxidative DNA damage.

FZR1 (CDH1) – is an adaptor protein for anaphase promoting complex/cyclosome (APC/C) E3 ubiquitin–protein ligase complex, contributing to cell cycle regulation and impacting the choice of DNA damage repair mechanism [151]. Proteasomal degradation of FZR1 reduces proliferation of VSMCs [152]. *FZR1* gene was identified by bioinformatics analysis to be potentially important in monocytes originating from PBMCs of atherosclerotic patients [153] In comparisons analysed in

current research *FZR1* expression was distinguishing CVD patients from controls and LEAD from CVD group, by upregulation and downregulation, respectively.

TREX2 is a keratinocyte–specific non–processive 3′–5′ deoxyribonuclease that is involved in genome maintenance by editing 3′–ends in multiple processes like DNA replication, recombination and various DNA repair pathways like error–free post–replication repair. It is engaged in regulation of keratinocyte apoptosis, thus contributing to skin inflammation, immune responses and preventing cancerogenesis induced by chemical or UV–B radiation. [154]. Activity of overexpressed protein may interfere with NHEJ DSBs repair disrupting exact DNA strand 3′–end rejoining [155]. Worth mentioning is the fact, that this exonuclease is strongly upregulated in human psoriasis, a hyperproliferative and inflammatory skin disease. Pathological cell proliferation and differentiation, disregulated immune responses, and angiogenesis are the major factors that underpin its pathogenesis [156] and importantly, the same mechanisms are engaged in vascular diseases. *TREX2* gene is pronouncedly upregulated in CVD patients, but downregulated in the group of arterial diseases patients (LEAD and AAA) when compared to CVD, suggesting distinct regulation of vascular changes in those diseases.

Gene sets correlated with both vascular–related process/term and DNA repair, unique for one comparison only had following composition: in AAA patients (AAA vs control group) there were six genes with differential expression: *NPAS2 (MOP4), PRRX1, RTEL1, SIRT7, USP10, VCP (p97)*; in LEAD vs CVD group there were three such genes: *ACTR2 (ARP2), SAMHD1, TERF2IP (RAP1);* in CVD patients (CVD vs control group) there were two characteristic genes: *HSPA1A, TRRAP;* and only one appeared in LEAD vs AAA comparison (*CLK2*).

- AAA vs control group: *NPAS2 (MOP4), PRRX1, RTEL1, SIRT7, USP10, VCP (p97)*

NPAS2 (MOP4) was correlated with DNA damage repair, where reduced *NPAS2* expression decreased DNA repair capacity [157]. It is also an element of the clock system, which is pronouncedly expressed in human vascular smooth muscle cells and in the murine vasculature. *NPAS2* cycles in the vasculature *in vivo*, and *in vitro* in vascular smooth muscle cells [158] Mice with inactive *NPAS2* had significant longer time to venous occlusion in femoral artery photochemical injury model [159]. Its decreased expression was noted in Crohn Disease– condition of digestive tract with autoimmune and inflammatory component [160]. This gene has lowered expression in AAA patients in comparison with control group. Interestingly HERC2 (mentioned earlier in the section 3.5.) cooperates with another clock protein (cryptochrome) in the regulation of NER, limiting the lifetime of XPA in a circadian manner [134] Expression of this gene is upregulated in CVD patients. Disruption of the molecular clock is a recognized risk factor for various health problems. It may also have profound repercussions in vascular diseases, due to many pathogenic processes involved during atherogenesis being indeed worsened when the clock is altered [161]. Moreover, it may give some interesting therapeutic opportunities like application of chronotherapy. This approach takes into account the daily oscillation of various metabolic processes governing uptake, transport and activation of medicines in order to apply different treatment regimens for the best outcome [134].

PRRX1 limits DNA damage in pancreatic tumor cells by regulating genes involved in DNA damage repair through interaction with FOXM1 (Forkhead Box M1) [162]. It is also known to contribute to vasculogenesis in different tissue and organism context– during rat embryonic pituitary development [163], in clear cell renal cell carcinoma [164] and in glioma [165]. Pathologic vasculogenesis is one of hallmarks of different vascular diseases, and PRRX1 disregulation may be useful in future AAA diagnosis and/or therapy.

RTEL1 was one of the most prominent candidate genes for arteriolar remodelling of coronary microvasculature in the spontaneously hypertensive rats [166]. Additionally, genetic variants of RTEL1 may have protective action against coronary heart disease in humans [167]. High expression levels of this gene may thus facilitate the vascular wall remodelling in AAA patients.

Evidence showed, that *SIRT7* has important role in vascular smooth muscle cell physiology. Its absence reduce neointimal formation following vascular injury by shaping vascular smooth muscle

cells proliferation and migration through Wnt/β –Catenin signaling pathway [168,169]. This mechanism could aggravate state of disease in AAA patients, especially, that high expression of this gene was observed in current assessment.

USP10 has been linked to foam cell formation by deubiquitinating and thus stabilizing CD36– the most significant scavenger receptor uptaking lipids in monocytes and macrophages. This gene was upregulated in AAA patients. Not surprisingly there are many examples of correlation between lipid metabolism and aneurysm. Lipid accumulation and oxidated lipids levels were associated with degeneration and rupture of the intracranial aneurysm [170]. Foam cells derived from VSMCs and subsequent accumulation of lipids were shown to promote intracranial aneurysms wall rupture [171]. Moreover, genetic polymorphisms in some genes responsible for lipid metabolism elements and deregulated cholesterol efflux were shown to be risk and susceptibility factors for AAA [172,173].

Due to angiotensin II (Ang II) signal transduction in VSMCs, **VCP** has an impact on blood pressure [67] The role of Ang II as a blood pressure elevating agent is well established. This molecule is engaged also in different processes, acting through angiotensin type 1 receptor Ang II is able to induce adverse effects like vasoconstriction, rise in aldosterone levels, sodium and fluid retention, inflammation and tissue injury, oxidative stress and fibrosis [174]. Levels of expression of angiotensin type 1 receptor and angiotensin type 2 receptor were unchanged in all groups of patients, not exceeding the threshold level of genes with mean number of reads higher than one (data not shown). Elevated levels of VCP expression may then facilitate stability of angiotensin type 1 receptor and enhance Ang II effects in AAA patients.

- LEAD vs CVD group: *ACTR2 (ARP2), SAMHD1, TERF2IP (RAP1)*

ACTR2 (ARP2) besides facilitating DNA repair has a role in cell motility. Neointima formation after vascular injury requires migration of VSMCs. ARP2/3 complex activates cytoplasmic actin polymerization and is involved in lamellipodia formation during VSMCs migration [175]. Although pathologic cell motility is important element of all presented diseases, upregulation of ACTR2 may be the element of cell motility mechanism, distinguishing LEAD from CVD.

SAMHD1 mutations are a common feature in Aicardi–Goutieres syndrome and other SAMHD1–related diseases being associated with cerebral vasculopathy [176-178]. One can speculate about engagement of SAMHD1 in vascular pathology, due to presumed contribution to maintenance of vessel homeostasis in LEAD but not in CVD patients.

TERF2IP (RAP1) in complex with TRF2 is required in protection of 3′ telomeric ends from illegitimate NHEJ [179]. In endothelial cells its phosphorylated form has another function. During simulated disturbed flow it is exported from the nucleus and contributes to cells' metabolism in a negative manner, inducing activation, senescence and apoptosis [180]. Its elevated expression suggest presence of before mentioned mechanisms in LEAD distinguishing this condition from CVD.

- CVD vs control group: *HSPA1A (HSP70), TRRAP*

HSPA1A is a molecular chaperone, controlling protein folding, translocation and degradation. Body of evidence suggests a tremendous contribution of this protein in regulation and facilitation of DNA repair pathways [181]. HSPA1A has anti–inflammatory properties and its decreased expression increases damage dealt by vascular risk factors involved in endothelial dysfunction [182]. Moreover, there is a considerable level of various polymorphisms in regulatory region of HSPA1A gene, exhibiting a tendency towards increased vascular risk in carriers [183]All in all, decreased expression of *HSPA1A* may be an element of decreased tolerance for stresses present in the course of CVD, compromising defense mechanisms, including DNA repair.

TRRAP is responsible for formation of lipid droplets consisting of triglicerides in HuH–7 cells [184] and positively regulates cholesterol metabolism and intake through coactivation of Liver X receptor alpha (LXRα) and Farnesoid X receptor (FXR) in HepG2 cells [185]. It is tempting to speculate, that elevated expression of TRRAP in CVD patients leads to aberrant function of LXRα, leading to symptoms of CVD in manner similar as in atherosclerosis [186]. It could also contribute to coagulation process through promoting abnormal FXR behaviour through increase of platelet

function, reduction of fibrinogen protein complex function and hepatic antithrombin III expression, like by the similar mechanism as observed in mice [187].

- LEAD vs AAA comparison (*CLK2*) and LEAD vs CVD comparison (*TOP1*)

CLK2 in worm *Caenorhabditis elegans* is an element of biological clock, physically interacts with S–phase checkpoint proteins– ATR, ATRIP, claspin and Chk1 and has critical role in S–phase checkpoint and DNA damage regulation [188]. Interestingly CLK2 in concert with DNA Topoisomerse I (TOP1) regulate alternative splicing of tissue factor (TF) mRNA in human endothelial cells what modulates its active forms expression. Tissue factor is the primary initiator of the coagulation cascade, appearing in two splicing variants– membrane –bound full length form (flTF) and soluble one (asHTF) in blood. The soluble one participates in thrombogenesis. CLK2 and TOP1 together are able to modulate physiological activities of TF in LEAD patients, albeit distinct from AAA and CVD [189].

2.7. *Miscellaneous genes revealed in current research and connected to AAA, CVD and LEAD (UVSSA, PPP4R2; RAD17P1, TDP2, RFC1; ZMPSTE24 (STE24))*

UVSSA gene product is required for transcription–coupled nucleotide excision repair [190], being an important interactor of transcription factor II H complex (TFIIH), [191] which regulates RNA polymerase II [192]. This may suggest a putative mechanism of indirect regulation of different genes by UVSSA–TFIIH interaction. Elevated expression of this gene was noticed in AAA and CVD patients, whereas low expression was a distinguishing characteristic between LEAD and both AAA and CVD. This opens a possibility for *UVSSA* to be a new diagnostic marker and/or therapeutic target in vascular diseases.

PPP4R2 is indispensable factor for DNA repair in human leukemia cells, engaged in human myelopoiesis. It is implicated in DNA repair of normal murine HSPC by regulating dephosphorylation of DNA damage repair signalling proteins [193]. This is another example, where regulation of a biological process may be important feature of the disease. Decrease of this gene expression may be a feature of AAA whereas increase could be indicative for differentiation of LEAD from AAA and CVD.

RAD17P1 is a pseudogene of *RAD17*. Its expression was decreased in CVD patients. Today it is known that pseudogenes have functional potential and are biologically relevant [194-196]. This interesting instance may point to alternative regulation modes in vascular diseases, especially of some aspects of CVD, but *RAD17P1* way of action still remains elusive. One of possible explanations is expression of non–coding RNAs, interfering with RAD17 function itself, thus its involvement in HR, histone remodeling, ATM activation, participation in DNA end resections, apurinic–apirimidinic sites repair and TLS [197,198].

TDP2 is a protein involved in DNA–protein crosslink repair. It is able to repair 3′–blocking lesions caused by DNA– topoisomerase 1 crosslinking, [199] and 5′ blocking lesions, when DNA topoisomerase 2 is engaged [200]. This could indicate some extent of compromising the ability to repair this characteristic type of DNA damage.

RCF1 is the largest subunit of heteropentameric replicator factor C complex with various functions ranging from maintaining the processivity of DNA polymerases to loading and unloading PCNA on DNA during replication and damage repair [201]. Upregulated expression of RCF1 may constitute an element of response towards DNA oxidative damage [202] in LEAD patients, distinguishing them from CVD ones.

ZMPSTE24 (STE24) is a protease providing cleavage of prelamin A, a precursor of lamin A– an important element of nuclear scaffold. Either mutations in prelamin–A gene or in ZMPSTE24 are causative for Hutchinson–Gilford progeria syndrome (HGPS) – an exceptionally rare autosomal disorder of accelerated aging [203]. HGPS patients are characterised by premature aging associated with accelerated atherosclerotic process causing coronary and carotid artery disease leading to death typically in the early teens. Interestingly, HGPS patients– derived endothelial cells have many

senescent features like increased LDL uptake, low levels of nitric oxide and impaired vessel formation [204]. Messner *et al*. associated chronic inflammation with increased expression of ZMPSTE24 and lamin A/C mRNA. Both markers also positively correlate with increased expression of the premature aging marker progerin which may be linked to cardiovascular aging [205]. Elevated expression of *ZMPSTE24* could then be attributed to mechanism increasing inflammation and senescence of vessels in LEAD but not in CVD.

List of Abbreviations:

2–OH–dATP	2–hydroxy–2′–deoxyadenosine 5′–triphosphate
8–OH–dATP	8–hydroxy–2′–deoxyadenosine 5′–triphosphate
8–OH–dGTP	8–hydroxy–2′–deoxyguanosine 5′–triphosphate
53BP1	Tumor Protein P53 Binding Protein 1
AAA	Abdominal aortic aneurysm
ARP3	Actin Related Protein 3
BAF200	BAF Nuclear Assembly Factor 1
BER	Base Excision Repair
BLM	Bloom helicase
BTR complex	Bloom helicase, topoisomerase 3, and the RMI1 and RMI2 scaffolding proteins
CMG complex	Human Replicative Helicase
CVD	Chronic Venous Disease
DDP	DNA Damage Prevention
DDR	DNA Damage Repair
DDT	DNA Damage Tolerance
dNTP	deoxynucleoside triphophate
DSBs	double strand breaks
FA/BRCA repair pathway	Fanconi Anemia/ Breast And Ovarian Cancer Susceptibility Protein
H3K18Ac	Lysine 18 acetylated in Histone H3
H2AK15ub	Lysine 15 ubiquitinated in histone H2A
HJ	Holliday Junction
HR	Homologous Recombination
HSPC	Hematopoietic Stem and Progenitor Cells
ICLs	Interstrand crosslinks
INO80	INO80 Complex ATPase Subunit
LEAD	Lower Extremities Arterial Disease
LXRα	Liver X receptor alpha
MA plot	Bland–Altman plot for visual representation of genomic data
MGMT	O^6–Methylguanine–DNA Methyltransferase
MMR	Mismatch repair
MUS81	MUS81 Structure-Specific Endonuclease Subunit
NER	nucleotide excision repair
NHEJ	Non–Homologous End Joining
p53	Tumor Protein P53
pol η	DNA polymerase eta

pol ι	DNA polymerase iota
POL iota	DNA polymerase iota
pol κ	DNA polymerase kappa
pol ζ	DNA polymerase zeta
Rev1	REV1 DNA Directed Polymerase
ROS	Reactive oxygen species
TIP60/NuA4	Lysine Acetyltransferase 5/ NuA4 histone acetyltransferase complex
TLS	Trans Lesion Synthesis
TS	Template Switching
SDSA	synthesis–dependent strand annealing
Shu complex	heterotetramer of Psy3, Csm2, Shu1, Shu2
SMC3	Structural Maintenance Of Chromosomes 3
SOSS1	Single Stranded DNA Binding Protein 1
SRCAP/SWR1	Snf2 Related CREBBP Activator Protein
STAG	stromal antigen
Swi/Snf complex	SWItch/Sucrose Non-Fermentable complex
SWS1–SWSAP1–SPIDR	Zinc Finger SWIM-Type Containing 7- SWIM-Type Zinc Finger 7 Associated Protein 1- Scaffold Protein Involved In DNA Repair
UV	ultraviolet
UVE-PLS	Uninformative Variable Elimination by Partial Least Squares
Wnt/*β* –Catenin	Wingless-Type MMTV Integration Site Family/ *β* –Catenin
VEGFA	Vascular Endothelial Growth Factor A
VSMCs	Vascular Smooth Muscle Cells

Supplementary text references:

1. Yu, W.; Zhang, L.; Wei, Q.; Shao, A. O^6–Methylguanine–DNA Methyltransferase (MGMT): Challenges and New Opportunities in Glioma Chemotherapy. *Front. Oncol.* **2020**, *9*, 1547. https://doi.org/10.3389/fonc.2019.01547
2. Ougland, R.; Rognes, T.; Klungland, A.; Larsen, E. Non–homologous functions of the AlkB homologs. *J. Mol. Cell Biol.* **2015**, *7*(6), 494–504. https://doi.org/10.1093/jmcb/mjv029
3. Vechtomova, Y. L.; Telegina, T. A.; Kritsky, M. S. Evolution of Proteins of the DNA Photolyase/Cryptochrome Family. *Biochemistry.* **2020**, *85* (Suppl 1), S131–S153. https://doi.org/10.1134/S0006297920140072
4. Reyes, G. X.; Schmidt, T. T.; Kolodner, R. D.; Hombauer, H. New insights into the mechanism of DNA mismatch repair. *Chromosoma* **2015**, *124*(4), 443–462. https://doi.org/10.1007/s00412–015–0514–0
5. Grundy, G. J.; Parsons, J. L. Base excision repair and its implications to cancer therapy. *Essays Biochem.* **2020**, *64*(5), 831–843. https://doi.org/10.1042/EBC20200013
6. Spivak G. Nucleotide excision repair in humans. *DNA repair* **2015**, *36*, 13–18. https://doi.org/10.1016/j.dnarep.2015.09.003
7. Kusakabe, M.; Onishi, Y.; Tada, H.; Kurihara, F.; Kusao, K.; Furukawa, M.; Iwai, S.; Yokoi, M.; Sakai, W.; Sugasawa, K. Mechanism and regulation of DNA damage recognition in nucleotide excision repair. *Genes Environ.* **2019**, *41*, 2. https://doi.org/10.1186/s41021–019–0119–6
8. Moreno, O. M.; Paredes, A. C.; Suarez–Obando, F.; Rojas, A. An update on Fanconi anemia: Clinical, cytogenetic and molecular approaches (Review). *Biomed. Rep.* **2021**, *15*(3), 74. https://doi.org/10.3892/br.2021.1450

9. Lopez–Martinez, D.; Liang, C. C.; Cohn, M. A. Cellular response to DNA interstrand crosslinks: the Fanconi anemia pathway. *Cell. Mol. Life Sci.* **2016**, *73*(16), 3097–3114. https://doi.org/10.1007/s00018–016–2218–x
10. Ackerson, S.M.; Romney, C.; Schuck, P.L.; Stewart, J.A. NHEJ To Join or Not to Join: Decision Points Along the Pathway to Double–Strand Break Repair vs. Chromosome End Protection. *Front. Cell Dev. Biol.* **2021**, *9*, 708763. https://doi.org/10.3389/fcell.2021.708763
11. Krejci, L.; Altmannova, V.; Spirek, M.; Zhao, X. Homologous recombination and its regulation. *Nucleic Acids Res.* **2012**, *40*(13), 5795–5818. https://doi.org/10.1093/nar/gks270
12. Elbakry, A.; Löbrich, M. Homologous Recombination Subpathways: A Tangle to Resolve. *Front. Genet.* **2021**, *12*, 723847. https://doi.org/10.3389/fgene.2021.723847
13. Pannunzio, N.R.; Watanabe, G.; Lieber, M.R. Nonhomologous DNA end–joining for repair of DNA double–strand breaks. *J. Biol. Chem.* **2018**, *293*(27), 10512–10523. https://doi.org/10.1074/jbc.TM117.000374
14. Chang, H.; Pannunzio, N.R.; Adachi, N.; Lieber, M.R. Non–homologous DNA end joining and alternative pathways to double–strand break repair. *Nat. Rev. Mol. Cell. Bio*. **2017**, *18*(8), 495–506. https://doi.org/10.1038/nrm.2017.48
15. Zhao, L.; Washington, M.T. Translesion Synthesis: Insights into the Selection and Switching of DNA Polymerases. *Genes* **2017**, *8*(1), 24. https://doi.org/10.3390/genes8010024
16. Ma, X.; Tang, T.S.; Guo, C. Regulation of translesion DNA synthesis in mammalian cells. *Environ. Mol. Mutagen.* **2020**, *61*(7), 680–692. https://doi.org/10.1002/em.22359
17. Maiorano, D.; El Etri, J.; Franchet, C.; Hoffmann, J.S. Translesion Synthesis or Repair by Specialized DNA Polymerases Limits Excessive Genomic Instability upon Replication Stress. *Int. J. Mol. Sci.* **2021**, *22*(8), 3924. https://doi.org/10.3390/ijms22083924
18. So, A.; Le Guen, T.; Lopez, B.S.; Guirouilh–Barbat, J. Genomic rearrangements induced by unscheduled DNA double strand breaks in somatic mammalian cells. *FEBS J.* **2017**, *284*(15), 2324–2344. https://doi.org/10.1111/febs.14053
19. Gao, Y.; Mutter–Rottmayer, E.; Zlatanou, A.; Vaziri, C.; Yang, Y. Mechanisms of Post–Replication DNA Repair. *Genes* **2017**, *8*(2), 64. https://doi.org/10.3390/genes8020064
20. Kamiya H. Mutagenicity of oxidized DNA precursors in living cells: Roles of nucleotide pool sanitization and DNA repair enzymes, and translesion synthesis DNA polymerases. *Mutation Res.* **2010**, *703*(1), 32–36. https://doi.org/10.1016/j.mrgentox.2010.06.003
21. Sarogni, P.; Pallotta, M. M.; Musio, A. Cornelia de Lange syndrome: from molecular diagnosis to therapeutic approach. *J. Med. Genet.* **2020**, *57*(5), 289–295. https://doi.org/10.1136/jmedgenet-2019-106277
22. Bauerschmidt, C.; Arrichiello, C.; Burdak-Rothkamm, S.; Woodcock, M.; Hill, M.A.; Stevens, D.L.; Rothkamm, K. Cohesin promotes the repair of ionizing radiation-induced DNA double-strand breaks in replicated chromatin. *Nucleic Acids Res.* **2010**, *38*(2), 477–487. https://doi.org/10.1093/nar/gkp976
23. Cheng, H.; Zhang, N.; Pati, D. Cohesin subunit RAD21: From biology to disease. *Gene* **2020**, *758*, 144966. https://doi.org/10.1016/j.gene.2020.144966
24. Garcia, P.; Fernandez-Hernandez, R.; Cuadrado, A.; Coca, I.; Gomez, A.; Maqueda, M.; Latorre-Pellicer, A.; Puisac, B.; Ramos, F.J.; Sandoval, J.; Esteller, M.; Mosquera, J.L.; Rodriguez, J.; Pié, J.; Losada, A.; Queralt, E. Disruption of NIPBL/Scc2 in Cornelia de Lange Syndrome provokes cohesin genome-wide redistribution with an impact in the transcriptome. *Nat. Commun.* **2021**, *12*(1), 4551. https://doi.org/10.1038/s41467-021-24808-z
25. Schierding, W.; Horsfield, J. A.; O'Sullivan, J. M. Low tolerance for transcriptional variation at cohesin genes is accompanied by functional links to disease-relevant pathways. *J. Med. Genet.* **2021**, *58*(8), 534–542. https://doi.org/10.1136/jmedgenet-2020-107095
26. Shrivastav, M.; De Haro, L.P.; & Nickoloff, J.A. Regulation of DNA double-strand break repair pathway choice. *Cell Res.* **2008**, *18*(1), 134–147. https://doi.org/10.1038/cr.2007.111

27. Marini, F.; Rawal, C.C.; Liberi, G.; Pellicioli, A. Regulation of DNA Double Strand Breaks Processing: Focus on Barriers. *Front. Mol. Biosci.* **2019**, *6*, 55. https://doi.org/10.3389/fmolb.2019.00055
28. Jilani, A.; Ramotar, D.; Slack, C.; Ong, C.; Yang, X.M.; Scherer, S.W.; Lasko, D.D. Molecular cloning of the human gene, PNKP, encoding a polynucleotide kinase 3' –phosphatase and evidence for its role in repair of DNA strand breaks caused by oxidative damage. *J. Biol Chem.* **1999**, *274*(34), 24176–24186. https://doi.org/10.1074/jbc.274.34.2417
29. Aceytuno, R.D.; Piett, C.G.; Havali –Shahriari, Z., Edwards, R.A.; Rey, M.; Ye, R.; Javed, F.; Fang, S.; Mani, R.; Weinfeld, M.; Hammel, M.; Tainer, J.A.; Schriemer, D.C.; Lees –Miller, S.P.; Glover, J. Structural and functional characterization of the PNKP –XRCC4 –LigIV DNA repair complex. *Nucleic Acids Res.* **2017**, *45*(10), 6238–6251. https://doi.org/10.1093/nar/gkx275
30. Casari, E.; Gobbini, E.; Gnugnoli, M.; Mangiagalli, M.; Clerici, M.; Longhese, M.P. Dpb4 promotes resection of DNA double –strand breaks and checkpoint activation by acting in two different protein complexes. *Nat. Commun.***2021**, *12*(1), 4750. https://doi.org/10.1038/s41467 –021 –25090 –9
31. Pursell, Z.F.; Isoz, I.; Lundström, E.B.; Johansson, E., Kunkel, T. A. Yeast DNA polymerase epsilon participates in leading –strand DNA replication. *Science* **2007**, *317*(5834), 127–130. https://doi.org/10.1126/science.1144067
32. Ngo, G.; Grimstead, J.W.; Baird, D.M. UPF1 promotes the formation of R loops to stimulate DNA double –strand break repair. *Nat. Commun.***2021,** *12*(1), 3849. https://doi.org/10.1038/s41467 –021 –24201 –w
33. Benedict, B.; van Bueren, M.A.; van Gemert, F.P.; Lieftink, C.; Guerrero Llobet, S.; van Vugt, M.A.; Beijersbergen, R.L.; Te Riele, H. The RECQL helicase prevents replication fork collapse during replication stress. *Life Sci. Alliance* **2020**, *3*(10), e202000668. https://doi.org/10.26508/lsa.202000668
34. Debnath, S.; Sharma, S. RECQ1 Helicase in Genomic Stability and Cancer. *Genes* **2020**, *11*(6), 622. https://doi.org/10.3390/genes11060622
35. Spagnolo, L.; Rivera –Calzada, A.; Pearl, L.H.; Llorca, O. Three –dimensional structure of the human DNA –PKcs/Ku70/Ku80 complex assembled on DNA and its implications for DNA DSB repair. *Mol. Cell* **2006**, *22*(4), 511–519. https://doi.org/10.1016/j.molcel.2006.04.013
36. Kragelund, B.B.; Weterings, E.; Hartmann –Petersen, R.; Keijzers, G. The Ku70/80 ring in Non –Homologous End –Joining: easy to slip on, hard to remove. *Front. Biosci. (Landmark Ed.)* **2016**, *21*, 514–527. https://doi.org/10.2741/4406
37. Mak, V. C.; Li, X.; Rao, L.; Zhou, Y.; Tsao, S.W.; Cheung, L.W. p85β alters response to EGFR inhibitor in ovarian cancer through p38 MAPK –mediated regulation of DNA repair. *Neoplasia* **2021**, *23*(7), 718–730. https://doi.org/10.1016/j.neo.2021.05.009
38. Fan, X.J.; Wang, Y.L.; Zhao, W.W.; Bai, S.M.; Ma,Y.; Yin, X.K.; Feng, L.L.; Feng, W.X.; Wang, Y.N.; Liu, Q.; Hung, M.C.; Wan, X.B. NONO phase separation enhances DNA damage repair by accelerating nuclear EGFR –induced DNA –PK activation. *Am. J. Cancer Res.***2021**, 11(6) 2838–2852.
39. Liccardi, G.; Hartley, J.A.; Hochhauser, D. EGFR nuclear translocation modulates DNA repair following cisplatin and ionizing radiation treatment. *Cancer Res.* **2011**, *71*(3), 1103–1114. https://doi.org/10.1158/0008 –5472.CAN –10 –2384
40. Li, J.; Ma, X.; Banerjee, S.; Baruah, S.; Schnicker, N.J.; Roh, E.; Ma, W.; Liu, K.; Bode, A.M.; Dong, Z. Structural basis for multifunctional roles of human Ints3 C –terminal domain. *J. Biol. Chem.***2021**, *296*, 100112. https://doi.org/10.1074/jbc.RA120.016393
41. Jia, Y.; Cheng, Z.; Bharath, S.R.; Sun, Q.; Su, N.; Huang, J.; Song, H. Crystal structure of the INTS3/INTS6 complex reveals the functional importance of INTS3 dimerization in DSB repair. *Cell discovery* **2021**, *7*(1), 66. https://doi.org/10.1038/s41421-021-00283-0

42. Bilokapic, S.; Suskiewicz, M.J.; Ahel, I.; Halic, M. Bridging of DNA breaks activates PARP2 –HPF1 to modify chromatin. *Nature* **2020**, 585(7826), 609–613. https://doi.org/10.1038/s41586 –020 –2725 –7
43. Suskiewicz, M.J.; Zobel, F.; Ogden, T.; Fontana, P.; Ariza, A.; Yang, J.C.; Zhu, K.; Bracken, L.; Hawthorne, W.J.; Ahel, D.; Neuhaus, D.; Ahel, I. HPF1 completes the PARP active site for DNA damage –induced ADP –ribosylation. *Nature* **2020**, 579(7800), 598–602. https://doi.org/10.1038/s41586 –020 –2013 –6
44. Fouquin, A.; Guirouilh –Barbat, J.; Lopez, B.; Hall, J.; Amor –Guéret, M.; Pennaneach, V. PARP2 controls double –strand break repair pathway choice by limiting 53BP1 accumulation at DNA damage sites and promoting end –resection. *Nucleic Acids Res.* **2017**, 45(21), 12325–12339. https://doi.org/10.1093/nar/gkx881
45. Schreiber, V.; Amé, J.C.; Dollé, P.; Schultz, I.; Rinaldi, B.; Fraulob, V.; Ménissier-de Murcia, J.; de Murcia, G. Poly(ADP-ribose) polymerase-2 (PARP-2) is required for efficient base excision DNA repair in association with PARP-1 and XRCC1. *J. Biol. Chem.* **2002**, *277*(25), 23028–23036. https://doi.org/10.1074/jbc.M202390200
46. Kim, M.; Kim, J.M. The role of USP1 autocleavage in DNA interstrand crosslink repair. *FEBS Lett.***2016**, *590*(3), 340–348. https://doi.org/10.1002/1873 –3468.12060
47. Cukras, S.; Lee, E.; Palumbo, E.; Benavidez, P.; Moldovan, G.L.; Kee, Y. The USP1 –UAF1 complex interacts with RAD51AP1 to promote homologous recombination repair. *Cell Cycle* **2016**, *15*(19), 2636–2646. https://doi.org/10.1080/15384101.2016.1209613
48. Bienko, M.; Green, C.M.; Sabbioneda, S.; Crosetto, N.; Matic, I.; Hibbert, R.G.; Begovic, T.; Niimi, A.; Mann, M.; Lehmann, A.R.; Dikic, I. Regulation of translesion synthesis DNA polymerase eta by monoubiquitination. *Mol. Cell* **2010**, *37*(3), 396–407. https://doi.org/10.1016/j.molcel.2009.12.039
49. Yang, K.; Moldovan, G.L.; Vinciguerra, P.; Murai, J.; Takeda, S.; D'Andrea, A.D. (2011). Regulation of the Fanconi anemia pathway by a SUMO-like delivery network. *Genes Dev.*, *25*(17), 1847–1858. https://doi.org/10.1101/gad.17020911
50. Tong, Z.B.; Ai, H.S.; Li, J. B. The Mechanism of Chromatin Remodeler SMARCAD1/Fun30 in Response to DNA Damage. *Front. Cell. Dev. Biol.* **2020**, *8*, 560098. https://doi.org/10.3389/fcell.2020.560098
51. Bantele, S.; Pfander, B. Nucleosome Remodeling by Fun30^{SMARCAD1} in the DNA Damage Response. *Front. Mol. Biosci.* **2019**, *6*, 78. https://doi.org/10.3389/fmolb.2019.00078
52. de Castro, R.O.; Previato, L.; Goitea, V.; Felberg, A.; Guiraldelli, M.F.; Filiberti, A.; Pezza, R.J. The chromatin –remodeling subunit Baf200 promotes homology –directed DNA repair and regulates distinct chromatin –remodeling complexes. *J. Biol. Chem.* **2017**, *292*(20), 8459–8471. https://doi.org/10.1074/jbc.M117.778183
53. Oba, A.; Shimada, S.; Akiyama, Y.; Nishikawaji, T.; Mogushi, K.; Ito, H.; Matsumura, S.; Aihara, A.; Mitsunori, Y.; Ban, D.; Ochiai, T.; Kudo, A.; Asahara, H.; Kaida, A.; Miura, M.; Tanabe, M.; Tanaka, S. ARID2 modulates DNA damage response in human hepatocellular carcinoma cells. *J. Hepatol.* **2017**, *66*(5), 942–951. https://doi.org/10.1016/j.jhep.2016.12.026
54. Słabicki, M.; Theis, M., Krastev, D.B.; Samsonov, S.; Mundwiller, E.; Junqueira, M.; Paszkowski –Rogacz, M.; Teyra, J.; Heninger, A.K.; Poser, I.; Prieur, F.; Truchetto, J.; Confavreux, C.; Marelli, C.; Durr, A.; Camdessanche, J.P.; Brice, A.; Shevchenko, A.; Pisabarro, M.T.; Stevanin, G.; Buchholz, F. A genome –scale DNA repair RNAi screen identifies SPG48 as a novel gene associated with hereditary spastic paraplegia. *PLoS Biol.* **2010**, *8*(6), e1000408. https://doi.org/10.1371/journal.pbio.1000408
55. Stante, M.; Minopoli, G.; Passaro, F.; Raia, M.; Vecchio, L.D.; Russo, T. Fe65 is required for Tip60 –directed histone H4 acetylation at DNA strand breaks. *Proc. Natl. Acad. Sci. U. S. A.* **2009**, 106(13), 5093–5098. https://doi.org/10.1073/pnas.0810869106

56. Gao, Y.; Gan, H.; Lou, Z.; Zhang, Z. Asf1a resolves bivalent chromatin domains for the induction of lineage –specific genes during mouse embryonic stem cell differentiation. *Proc. Natl. Acad. Sci U. S. A.* **2018**, *115*(27), E6162–E6171. https://doi.org/10.1073/pnas.1801909115
57. Wu, Y.; Li, X.; Yu, J.; Björkholm, M.; Xu, D. ASF1a inhibition induces p53 –dependent growth arrest and senescence of cancer cells. *Cell Death Dis.* **2019**, *10*(2), 76. https://doi.org/10.1038/s41419 –019 –1357 –z
58. Li, L.; Shi, L.; Yang, S.; Yan, R.; Zhang, D.; Yang, J.; He, L.; Li, W.; Yi, X.; Sun, L.; Liang, J.; Cheng, Z.; Shi, L.; Shang, Y.; Yu, W. SIRT7 is a histone desuccinylase that functionally links to chromatin compaction and genome stability. *Nat. Commun.***2016**, *7*, 12235. https://doi.org/10.1038/ncomms12235
59. Paredes, S.; Chua, K.F. SIRT7 clears the way for DNA repair. *EMBO J.* **2016**, *35*(14), 1483–1485. https://doi.org/10.15252/embj.201694904
60. Vazquez, B.N.; Thackray, J.K.; Simonet, N.G.; Kane-Goldsmith, N.; Martinez-Redondo, P.; Nguyen, T.; Bunting, S.; Vaquero, A.; Tischfield, J.A.; Serrano, L. SIRT7 promotes genome integrity and modulates non-homologous end joining DNA repair. *EMBO J.* **2016**, *35*(14), 1488–1503. https://doi.org/10.15252/embj.201593499
61. Prakash, R.; Sandoval, T.; Morati, F.; Zagelbaum, J.A.; Lim, P.X.; White, T.; Taylor, B.; Wang, R.; Desclos, E.; Sullivan, M.R.; Rein, H.L.; Bernstein, K.A.; Krawczyk, P.M.; Gautier, J.; Modesti, M.; Vanoli, F.; Jasin, M. Distinct pathways of homologous recombination controlled by the SWS1 –SWSAP1 –SPIDR complex. *Nat. Commun.***2021**, *12*(1), 4255. https://doi.org/10.1038/s41467 –021 –24205 –6
62. Martino, J.; Brunette, G.J.; Barroso –González, J.; Moiseeva, T.N.; Smith, C.M.; Bakkenist, C.J.; O'Sullivan, R.J.; Bernstein, K.A. The human Shu complex functions with PDS5B and SPIDR to promote homologous recombination. *Nucleic Acids Res.* **2019**, *47*(19), 10151–10165. https://doi.org/10.1093/nar/gkz738
63. Wang, Z.; Zhang, H.; Liu, J.; Cheruiyot, A.; Lee, J. H.; Ordog, T.; Lou, Z.; You, Z.; Zhang, Z. USP51 deubiquitylates H2AK13,15ub and regulates DNA damage response. *Genes Dev.* **2016**, *30*(8), 946–959. https://doi.org/10.1101/gad.271841.115
64. Walser, F.; Mulder, M.; Bragantini, B.; Burger, S.; Gubser, T.; Gatti, M.; Botuyan, M.V.; Villa, A.; Altmeyer, M.; Neri, D.; Ovaa, H.; Mer, G.; Penengo, L. Ubiquitin Phosphorylation at Thr12 Modulates the DNA Damage Response. *Mol. Cell* **2020**, *80*(3), 423–436.e9. https://doi.org/10.1016/j.molcel.2020.09.017
65. Kilgas, S.; Singh, A.N.; Paillas, S.; Then, C.K.; Torrecilla, I.; Nicholson, J.; Browning, L.; Vendrell, I.; Konietzny, R.; Kessler, B.M.; Kiltie, A.E.; Ramadan, K. p97/VCP inhibition causes excessive MRE11 –dependent DNA end resection promoting cell killing after ionizing radiation. *Cell Rep.* **2021**, *35*(8), 109153. https://doi.org/10.1016/j.celrep.2021.109153
66. Swan, R.L.; Cowell, I.G.; Austin, C.A. A Role for VCP/p97 in the Processing of Drug –Stabilized TOP2 –DNA Covalent Complexes. *Mol. Pharmacol.* **2021**, *100*(1), 57–62. https://doi.org/10.1124/molpharm.121.000262
67. Schmitz, U.; Ishida, M.; Berk, B. C. Angiotensin II stimulates tyrosine phosphorylation of phospholipase C –gamma –associated proteins. Characterization of a c –Src –dependent 97 –kD protein in vascular smooth muscle cells. *Circ. Res.***1997**, *81*(4), 550–557. https://doi.org/10.1161/01.res.81.4.550
68. Tao, W.; Hong, Y.; He, H.; Han, Q.; Mao, M.; Hu, B.; Zhang, H.; Huang, X.; You, W.; Liang, X.; Zhang, Y.; Li, X. MicroRNA –199a –5p aggravates angiotensin II –induced vascular smooth muscle cell senescence by targeting Sirtuin –1 in abdominal aortic aneurysm. *J. Cell. Mol. Med.* **2021**, *25*(13), 6056–6069. Advance online publication. https://doi.org/10.1111/jcmm.16485
69. Coggins, S.A.; Mahboubi, B.; Schinazi, R.F.; Kim, B. SAMHD1 Functions and Human Diseases. *Viruses* **2020**, *12*(4), 382. https://doi.org/10.3390/v12040382
70. Daddacha, W.; Koyen, A.E.; Bastien, A.J.; Head, P.E.; Dhere, V.R.; Nabeta, G.N.; Connolly, E.C.; Werner, E.; Madden, M.Z.; Daly, M.B.; Minten, E.V.; Whelan, D.R.; Schlafstein, A.J.;

Zhang, H.; Anand, R.; Doronio, C.; Withers, A.E.; Shepard, C.; Sundaram, R.K.; Deng, X.; Dynan, W.S.; Wang, Y.; Bindra, R.S.; Cejka, P.; Rothenberg, E.; Doetsch, P.W.; Kim, B.; Yu., D.S. SAMHD1 Promotes DNA End Resection to Facilitate DNA Repair by Homologous Recombination. *Cell Rep.* **2017**, *20*(8), 1921–1935. https://doi.org/10.1016/j.celrep.2017.08.008

71. Rice, G.I.; Bond, J.; Asipu, A.; Brunette, R.L.; Manfield, I. W.; Carr, I.M.; Fuller, J.C.; Jackson, R.M.; Lamb, T.; Briggs, T.A.; Ali, M.; Gornall, H.; Couthard, L.R.; Aeby, A.; Attard-Montalto, S.P.; Bertini, E.; Bodemer, C.; Brockmann, K.; Brueton, L.A.; Corry, P.C.; Desguerre, I.; Fazzi E.; Cazorla, A.G.; Gener, B.; Hamel, B.C.; Heiberg, A.; Hunter, M.; van der Knaap, M.S.; Kumar, R.; Lagae, L.; Landrieu, P.G.; Lourenco, C.M.; Marom, D.; McDermott, M.F.; van der Merwe, W.; Orcesi, S.; Prendiville, J.S.; Rasmussen, M.; Shalev, S.A.; Soler, D.M.; Shinawi, M.; Spiegel, R.; Tan, T.Y.; Vanderver, A.; Wakeling, E.L.; Wassmer, E.; Whittaker, E.; Lebon, P.; Stetson, D.B.; Bonthron, D.T.; Crow, Y.J. Mutations involved in Aicardi-Goutières syndrome implicate SAMHD1 as regulator of the innate immune response. *Nat. Genet.* **2009,** *41*(7), 829–832. https://doi.org/10.1038/ng.373
72. Lu, H.; Guo, X.; Meng, X.; Liu, J.; Allen, C.; Wray, J.; Nickoloff, J.A.; Shen, Z. The BRCA2 –interacting protein BCCIP functions in RAD51 and BRCA2 focus formation and homologous recombinational repair. *Mol. Cell. Biol.* **2005**, *25*(5), 1949–1957. https://doi.org/10.1128/MCB.25.5.1949 –1957.2005
73. Jurkiw, T.J.; Tumbale, P.P.; Schellenberg, M.J.; Cunningham –Rundles, C.; Williams, R.S.; O'Brien, P.J. LIG1 syndrome mutations remodel a cooperative network of ligand binding interactions to compromise ligation efficiency. *Nucleic Acids Res.* **2021**, *49*(3), 1619–1630. https://doi.org/10.1093/nar/gkaa1297
74. Bellelli, R.; Youds, J.; Borel, V.; Svendsen, J.; Pavicic –Kaltenbrunner, V.; Boulton, S.J. Synthetic Lethality between DNA Polymerase Epsilon and RTEL1 in Metazoan DNA Replication. *Cell Rep.* **2020**, *31*(8), 107675. https://doi.org/10.1016/j.celrep.2020.107675
75. Björkman, A.; Johansen, S. L.; Lin, L.; Schertzer, M.; Kanellis, D.C.; Katsori, A.M.; Christensen, S.T.; Luo, Y.; Andersen, J.S.; Elsässer, S.J.; Londono –Vallejo, A.; Bartek, J.; Schou, K.B. Human RTEL1 associates with Poldip3 to facilitate responses to replication stress and R –loop resolution. *Genes Dev.* **2020**, *34*(15 –16), 1065–1074. https://doi.org/10.1101/gad.330050.119
76. Bizard, A.H.; Hickson, I.D. The many lives of type IA topoisomerases. *J. Biol. Chem.* **2020,** May 15;295(20):7138 –7153. doi: 10.1074/jbc.REV120.008286. Epub **2020,** Apr 10. PMID: 32277049; PMCID: PMC7242696.
77. Wyatt, H.D.; Sarbajna, S.; Matos, J.; West, S.C. Coordinated actions of SLX1-SLX4 and MUS81-EME1 for Holliday junction resolution in human cells. *Mol. Cell* **2013**, *52*(2), 234–247. https://doi.org/10.1016/j.molcel.2013.08.035
78. Amangyeld, T.; Shin, Y.K.; Lee, M.; Kwon, B.; Seo, Y.S. Human MUS81 –EME2 can cleave a variety of DNA structures including intact Holliday junction and nicked duplex. *Nucleic Acids Res.* **2014**, *42*(9), 5846–5862. https://doi.org/10.1093/nar/gku237
79. Falquet, B.; Rass, U. Structure –Specific Endonucleases and the Resolution of Chromosome Underreplication. *Genes* **2019**, *10*(3), 232. https://doi.org/10.3390/genes10030232
80. Kessous, R.; Octeau, D.; Klein, K.; Tonin, P.N.; Greenwood, C.; Pelmus, M.; Laskov, I.; Kogan, L.; Salvador, S.; Lau, S.; Yasmeen, A.; Gotlieb, W.H. Distinct homologous recombination gene expression profiles after neoadjuvant chemotherapy associated with clinical outcome in patients with ovarian cancer. *Gynecol. Oncol.* **2018**, *148*(3), 553–558. https://doi.org/10.1016/j.ygyno.2018.01.017
81. Nomura, Y.; Adachi, N.; Koyama, H. Human Mus81 and FANCB independently contribute to repair of DNA damage during replication. *Genes Cells.* **2007**, *12*(10), 1111–1122. https://doi.org/10.1111/j.1365 –2443.2007.01124.x
82. Miner, G.H.; Renton, A.E.; Taubenfeld, E.; Tadros, R.O.; Marcora, E.; Lookstein, R.A.; Faries, P.L.; Marin, M.L. Whole genome sequencing identifies loci specifically associated with

thoracic aortic wall defects and abdominal aortic aneurysms in patients with European ancestry. *JVS Vasc. Sci.* **2020**, *1*, 233–245. https://doi.org/10.1016/j.jvssci.2020.09.001
83. Ghosh, D.; Raghavan, S.C. 20 years of DNA Polymerase μ, the polymerase that still surprises. *FEBS J.* **2021**, 10.1111/febs.15852. Advance online publication. https://doi.org/10.1111/febs.15852
84. Lee, K.Y.; Im, J.S.; Shibata, E.; Dutta, A. ASF1a Promotes Non –homologous End Joining Repair by Facilitating Phosphorylation of MDC1 by ATM at Double –Strand Breaks. *Mol. Cell* **2017**, *68*(1), 61–75.e5. https://doi.org/10.1016/j.molcel.2017.08.021
85. Francica, P.; Mutlu, M.; Blomen, V.A.; Oliveira, C.; Nowicka, Z.; Trenner, A.; Gerhards, N.M.; Bouwman, P.; Stickel, E.; Hekkelman, M.L.; Lingg, L.; Klebic, I.; van de Ven, M.; de Korte-Grimmerink, R.; Howald, D.; Jonkers, J.; Sartori, A.A.; Fendler, W.; Chapman, J.R.; Brummelkamp, T.; Rottenberg, S. Functional Radiogenetic Profiling Implicates ERCC6L2 in Non-homologous End Joining. *Cell Rep.* **2020**, *32*(8), 108068. https://doi.org/10.1016/j.celrep.2020.108068
86. Hang, Q.; Zeng, L.; Wang, L.; Nie, L.; Yao, F.; Teng, H.; Deng, Y.; Yap, S.; Sun, Y.; Frank, S.J.; Chen, J.; Ma, L. Non-canonical function of DGCR8 in DNA double-strand break repair signaling and tumor radioresistance. *Nat. Commun.* **2021**, *12*(1), 4033. https://doi.org/10.1038/s41467-021-24298-z
87. Aquila, L.; Atanassov, B.S. Regulation of Histone Ubiquitination in Response to DNA Double Strand Breaks. *Cells* **2020**, *9*(7), 1699. https://doi.org/10.3390/cells9071699
88. Keyamura, K.; Arai, K.; Hishida, T. Srs2 and Mus81-Mms4 Prevent Accumulation of Toxic Inter-Homolog Recombination Intermediates. *PLoS Genet.* **2016**, *12*(7), e1006136. https://doi.org/10.1371/journal.pgen.1006136 PLoS. 2016 Jul 7;12(7):e1006136. doi: 10.1371/journal.pgen.1006136. eCollection 2016 Jul.
89. Ye, B.; Hou, N.; Xiao, L.; Xu, Y.; Xu, H.; Li, F. Dynamic monitoring of oxidative DNA double-strand break and repair in cardiomyocytes. *Cardiovasc. Pathol.* **2016**, *25*(2), 93–100. https://doi.org/10.1016/j.carpath.2015.10.010
90. Nair, N.; Shoaib, M.; Sørensen, C.S. Chromatin Dynamics in Genome Stability: Roles in Suppressing Endogenous DNA Damage and Facilitating DNA Repair. *Int. J. Mol. Sci.* **2017**, *18*(7), 1486. https://doi.org/10.3390/ijms18071486
91. Boohaker, R. J.; Xu, B. The versatile functions of ATM kinase. *Biomed J.* **2014**, *37*(1), 3–9. https://doi.org/10.4103/2319 –4170.125655
92. Berger, N.D.; Stanley, F.; Moore, S.; Goodarzi, A.A. ATM –dependent pathways of chromatin remodelling and oxidative DNA damage responses. *Philos. Trans. R. Soc. Lond. B Biol. Sci.* **2017**, *372*(1731), 20160283. https://doi.org/10.1098/rstb.2016.0283
93. Bellelli, R.; Borel, V.; Logan, C.; Svendsen, J.; Cox, D.E.; Nye, E.; Metcalfe, K.; O'Connell, S.M.; Stamp, G.; Flynn, H.R.; Snijders, A.P.; Lassailly, F.; Jackson, A.; Boulton, S.J. Polε Instability Drives Replication Stress, Abnormal Development, and Tumorigenesis. *Mol. Cell* **2018**, *70*(4), 707–721.e7. https://doi.org/10.1016/j.molcel.2018.04.008
94. Bellelli, R.; Belan, O.; Pye, V.E.; Clement, C.; Maslen, S.L.; Skehel, J.M.; Cherepanov, P.; Almouzni, G.; Boulton, S.J. POLE3-POLE4 Is a Histone H3-H4 Chaperone that Maintains Chromatin Integrity during DNA Replication. *Mol. Cell* **2018**, *72*(1), 112–126.e5. https://doi.org/10.1016/j.molcel.2018.08.043
95. Wang, Y.; Zhu, L.; Guo, M.; Sun, G.; Zhou, K.; Pang, W.; Cao, D.; Tang, X.; Meng, X. Histone methyltransferase WHSC1 inhibits colorectal cancer cell apoptosis via targeting anti – apoptotic BCL2. *Cell Death Discov.* **2021**, *7*(1), 19. https://doi.org/10.1038/s41420 –021 –00402 –6
96. Liu, C.; Cai, B.; Li, D.; Yao, Y. Wolf-Hirschhorn syndrome candidate 1 facilitates alveolar macrophage pyroptosis in sepsis-induced acute lung injury through NEK7-mediated NLRP3 inflammasome activation. *Innate Immun.* **2021**, *27*(6), 437–447. https://doi.org/10.1177/17534259211035426

97. Liu, H.H.; Lee, C.H.; Hsieh, Y.C.; Hsu, D.W.; Cho, E.C. Multiple myeloma driving factor WHSC1 is a transcription target of oncogene HMGA2 that facilitates colon cancer proliferation and metastasis. *Biochem. Biophys. Res. Commun.* **2021**, *567*, 183–189. https://doi.org/10.1016/j.bbrc.2021.06.034
98. Dai, J.; Jiang, L.; Qiu, L.; Shao, Y.; Shi, P.; Li, J. WHSC1 Promotes Cell Proliferation, Migration, and Invasion in Hepatocellular Carcinoma by Activating mTORC1 Signaling. *Onco. Targets Ther.* **2020,** *13*, 7033–7044. https://doi.org/10.2147/OTT.S248570
99. Costelloe, T.; Louge, R.; Tomimatsu, N.; Mukherjee, B.; Martini, E.; Khadaroo, B.; Dubois, K.; Wiegant, W.W.; Thierry, A.; Burma, S.; van Attikum, H.; Llorente, B. The yeast Fun30 and human SMARCAD1 chromatin remodellers promote DNA end resection. *Nature* **2012**, *489*(7417), 581–584. https://doi.org/10.1038/nature11353
100. He, L.; Tian, X.; Zhang, H.; Hu, T.; Huang, X.; Zhang, L.; Wang, Z.; Zhou, B. (2014). BAF200 is required for heart morphogenesis and coronary artery development. *PloS one*, *9*(10), e109493. https://doi.org/10.1371/journal.pone.0109493
101. Murphy, K.J.; Cutter, A.R.; Fang, H.; Postnikov, Y.V.; Bustin, M.; Hayes, J.J. HMGN1 and 2 remodel core and linker histone tail domains within chromatin. *Nucleic Acids Res.* **2017**, *45*(17), 9917–9930. https://doi.org/10.1093/nar/gkx579
102. Subramanian, M.; Gonzalez, R.W.; Patil, H.; Ueda, T.; Lim, J.H.; Kraemer, K.H.; Bustin, M.; Bergel, M. The nucleosome –binding protein HMGN2 modulates global genome repair. *FEBS J.* **2009**, *276*(22), 6646–6657. https://doi.org/10.1111/j.1742 –4658.2009.07375.x
103. Gerlitz G. HMGNs, DNA repair and cancer. *Biochim. Biophys. Acta* **2010**, *1799*(1-2), 80–85. https://doi.org/10.1016/j.bbagrm.2009.10.007
104. Caridi, C.P.; D'Agostino, C.; Ryu, T.; Zapotoczny, G.; Delabaere, L.; Li, X.; Khodaverdian, V.Y.; Amaral, N.; Lin, E.; Rau, A.R.; Chiolo, I. Nuclear F-actin and myosins drive relocalization of heterochromatic breaks. *Nature* **2018**, *559*(7712), 54–60. https://doi.org/10.1038/s41586-018-0242-8
105. Hurst, V.; Shimada, K.; Gasser, S.M. Nuclear Actin and Actin –Binding Proteins in DNA Repair. *Trends Cell Biol.* **2019**, *29*(6), 462–476. https://doi.org/10.1016/j.tcb.2019.02.010
106. Yao, T.; Song, L.; Jin, J.; Cai, Y.; Takahashi, H.; Swanson, S.K.; Washburn, M.P.; Florens, L.; Conaway, R.C.; Cohen, R.E.; Conaway, J.W. Distinct modes of regulation of the Uch37 deubiquitinating enzyme in the proteasome and in the Ino80 chromatin-remodeling complex. *Mol. Cell* **2008**, 31(6), 909–917. https://doi.org/10.1016/j.molcel.2008.08.027
107. Feris, E.J.; Hinds, J.W.; Cole, M.D. Formation of a structurally –stable conformation by the intrinsically disordered MYC:TRRAP complex. *PloS One* **2019**, *14*(12), e0225784. https://doi.org/10.1371/journal.pone.0225784
108. Murr, R.; Vaissière, T.; Sawan, C.; Shukla, V.; Herceg, Z. Orchestration of chromatin-based processes: mind the TRRAP. *Oncogene* **2007**, *26*(37), 5358–5372. https://doi.org/10.1038/sj.onc.1210605
109. Woodbine, L.; Brunton, H.; Goodarzi, A.A.; Shibata, A.; Jeggo, P.A. Endogenously induced DNA double strand breaks arise in heterochromatic DNA regions and require ataxia telangiectasia mutated and Artemis for their repair. *Nucleic Acids Res.* **2011**, 39(16), 6986–6997. https://doi.org/10.1093/nar/gkr331
110. Senoner, T.; Dichtl, W. Oxidative Stress in Cardiovascular Diseases: Still a Therapeutic Target?. *Nutrients* **2019**, 11(9), 2090. https://doi.org/10.3390/nu11092090
111. Cervantes Gracia, K.; Llanas-Cornejo, D.; Husi, H. CVD and Oxidative Stress. *J. Clin. Med.* **2017**, *6*(2), 22. https://doi.org/10.3390/jcm6020022
112. Tominaga, K.; Leung, J.K.; Rookard, P.; Echigo, J.; Smith, J. R.; & Pereira-Smith, O. M. MRGX is a novel transcriptional regulator that exhibits activation or repression of the B-myb promoter in a cell type-dependent manner. *J Biol. Chem.* **2003**, *278*(49), 49618–49624. https://doi.org/10.1074/jbc.M309192200

113. Cai, Y.; Jin, J.; Tomomori-Sato, C.; Sato, S.; Sorokina, I.; Parmely, T.J.; Conaway, R.C.; Conaway, J.W. Identification of new subunits of the multiprotein mammalian TRRAP/TIP60-containing histone acetyltransferase complex. *J Biol. Chem.* **2003**, *278*(44), 42733–42736. https://doi.org/10.1074/jbc.C300389200
114. Bomfim, M.M.; Andrade, G.M.; Del Collado, M.; Sangalli, J.R.; Fontes, P.K.; Nogueira, M.; Meirelles, F. V.; da Silveira, J.C.; Perecin, F. Antioxidant responses and deregulation of epigenetic writers and erasers link oxidative stress and DNA methylation in bovine blastocysts. *Mol.Reprod. Dev.* **2017**, *84*(12), 1296–1305. https://doi.org/10.1002/mrd.22929
115. Friedberg, E.C.; Wood, R.D. New insights into the combined Cockayne/xeroderma pigmentosum complex: human XPG protein can function in transcription factor stability. *Mol. Cell* **2007**, *26*(2), 162–164. https://doi.org/10.1016/j.molcel.2007.04.002
116. Ferri, D.; Orioli. D.; Botta, E. Heterogeneity and overlaps in nucleotide excision repair disorders. *Clin Genet.* **2020** Jan;97(1):12 –24. doi: 10.1111/cge.13545. Epub 2019 Apr 22. PMID: 30919937.
117. Durik, M.; Kavousi, M.; van der Pluijm, I.; Isaacs, A.; Cheng, C.; Verdonk, K.; Loot, A.E.; Oeseburg, H.; Bhaggoe, U.M.; Leijten, F.; van Veghel, R.; de Vries, R.; Rudez, G.; Brandt, R.; Ridwan, Y.R.; van Deel, E.D.; de Boer, M.; Tempel, D.; Fleming, I.; Mitchell, G.F.; Verwoert, G.C.; Tarasov, K.V.; Uitterlinden, A.G.; Hofman, A.; Duckers, H.J.; van Duijn, C.M.; Oostra, B.A.; Witteman, J.C.; Duncker, D.J.; Danser, A.H.; Hoeijmakers, J.H.; Roks, A.J. Nucleotide excision DNA repair is associated with age –related vascular dysfunction. *Circulation* **2012**, *126*(4), 468–478. https://doi.org/10.1161/CIRCULATIONAHA.112.104380
118. Aguilera –Aguirre, L.; Hosoki, K.; Bacsi, A.; Radák, Z.; Wood, T.G.; Widen, S.G.; Sur, S.; Ameredes, B.T.; Saavedra –Molina, A.; Brasier, A.R.; Ba, X.; Boldogh, I. Whole transcriptome analysis reveals an 8 –oxoguanine DNA glycosylase –1 –driven DNA repair –dependent gene expression linked to essential biological processes. *Free Radic. Biol. Med.* **2015**, *81*, 107–118. https://doi.org/10.1016/j.freeradbiomed.2015.01.004
119. Brasier, A.R.; Boldogh, I. Targeting inducible epigenetic reprogramming pathways in chronic airway remodeling. *Drugs Context.* **2019**, *8*, 2019 –8 –3. https://doi.org/10.7573/dic.2019 –8 –3
120. Hao, W.; Wang, J.; Zhang, Y.; Wang, C.; Xia, L.; Zhang, W.; Zafar, M.; Kang, J.Y.; Wang, R.; Ali Bohio, A.; Pan, L.; Zeng, X.; Wei, M.; Boldogh, I.; Ba, X. Enzymatically inactive OGG1 binds to DNA and steers base excision repair toward gene transcription. *FASEB J.***2020**, *34*(6), 7427–7441. https://doi.org/10.1096/fj.201902243R
121. Tumurkhuu, G.; Shimada, K.; Dagvadorj, J.; Crother, T.R.; Zhang, W.; Luthringer, D.; Gottlieb, R.A.; Chen, S.; Arditi, M. Ogg1 –Dependent DNA Repair Regulates NLRP3 Inflammasome and Prevents Atherosclerosis. *Circ. Res.***2016**, *119*(6), e76–e90. https://doi.org/10.1161/CIRCRESAHA.116.308362
122. Tummala, H.; Kirwan, M.; Walne, A.J.; Hossain, U.; Jackson, N.; Pondarre, C.; Plagnol, V.; Vulliamy, T.; Dokal, I. ERCC6L2 mutations link a distinct bone-marrow-failure syndrome to DNA repair and mitochondrial function. *Am. J. Hum. Genet.* **2014,** *94*(2), 246–256. https://doi.org/10.1016/j.ajhg.2014.01.007
123. Jin, J.O.; Puranik, N.; Bui, Q.T.; Yadav, D.; Lee, P.C. The Ubiquitin System: An Emerging Therapeutic Target for Lung Cancer. *Int. J. Mol. Sci.* **2021,** *22*(17), 9629. https://doi.org/10.3390/ijms22179629
124. Brinkmann, K.; Schell, M.; Hoppe, T.; Kashkar, H. Regulation of the DNA damage response by ubiquitin conjugation. *Front. Genet.* **2015,** *6*, 98. https://doi.org/10.3389/fgene.2015.00098
125. He, M.; Zhou, Z.; Shah, A.A.; Zou, H.; Tao, J.; Chen, Q.; Wan, Y. The emerging role of deubiquitinating enzymes in genomic integrity, diseases, and therapeutics. *Cell Biosci*. **2016,** *6*, 62. https://doi.org/10.1186/s13578-016-0127-1
126. Masuda, Y.; Piao, J.; Kamiya, K. DNA replication-coupled PCNA mono-ubiquitination and polymerase switching in a human in vitro system. *J. Mol. Biol*. **2010,** *396*(3), 487–500. https://doi.org/10.1016/j.jmb.2010.01.003

127. Liu, C.; Wang, D.; Wu, J.; Keller, J.; Ma, T.; Yu, X. RNF168 forms a functional complex with RAD6 during the DNA damage response. *J. Cell Sci.* **2013,** *126*(Pt 9), 2042–2051. https://doi.org/10.1242/jcs.122945
128. Oh, J.H.; Hyun, J.Y.; Chen, S.J.; Varshavsky, A. Five enzymes of the Arg/N –degron pathway form a targeting complex: The concept of superchanneling. *Proc. Natl. Acad. Sci U. S. A.* **2020**, May 19;117(20):10778 –10788. doi: 10.1073/pnas.2003043117. Epub 2020 May 4. PMID: 32366662; PMCID: PMC7245096.
129. Song, E.; Fan, P.; Huang, B.; Deng, H.B.; Cheung, B.M.; Félétou, M.; Vilaine, J.P.; Villeneuve, N.; Xu, A.; Vanhoutte, P. M.; Wang, Y. Deamidated lipocalin –2 induces endothelial dysfunction and hypertension in dietary obese mice. *J. Am. Heart Assoc.* **2014**, *3*(2), e000837. https://doi.org/10.1161/JAHA.114.000837
130. Dutta, B.; Park, J.E.; Kumar, S.; Hao, P.; Gallart –Palau, X.; Serra, A.; Ren, Y.; Sorokin, V.; Lee, C.N.; Ho, H.H.; de Kleijn, D.; Sze, S.K. Monocyte adhesion to atherosclerotic matrix proteins is enhanced by Asn –Gly –Arg deamidation. *Sci. Rep.* **2017**, *7*(1), 5765. https://doi.org/10.1038/s41598 –017 –06202 –2
131. Zhang, M.; Hu, C.; Tong, D.; Xiang, S.; Williams, K.; Bai, W.; Li, G.M.; Bepler, G.; Zhang, X. Ubiquitin –specific Peptidase 10 (USP10) Deubiquitinates and Stabilizes MutS Homolog 2 (MSH2) to Regulate Cellular Sensitivity to DNA Damage. *J. Biol. Chem.* **2016**, *291*(20), 10783–10791. https://doi.org/10.1074/jbc.M115.700047
132. Kim, H.; Dejsuphong, D.; Adelmant, G.; Ceccaldi, R.; Yang, K.; Marto, J.A.; D'Andrea, A.D. Transcriptional repressor ZBTB1 promotes chromatin remodeling and translesion DNA synthesis. *Mol. Cell* **2014**, *54*(1), 107–118. https://doi.org/10.1016/j.molcel.2014.02.017
133. Wu, W.; Sato, K.; Koike, A.; Nishikawa, H.; Koizumi, H.; Venkitaraman, A.R.; Ohta, T. HERC2 is an E3 ligase that targets BRCA1 for degradation. *Cancer Res.* **2010**, *70*(15), 6384–6392. https://doi.org/10.1158/0008 –5472.CAN –10 –1304
134. Kang, T.H.; Lindsey –Boltz, L.A.; Reardon, J.T.; Sancar, A. Circadian control of XPA and excision repair of cisplatin –DNA damage by cryptochrome and HERC2 ubiquitin ligase. *Proc. Natl. Acad. Sci U. S. A.* **2010**, *107*(11), 4890–4895. https://doi.org/10.1073/pnas.0915085107
135. Sood, R.; Makalowska, I.; Galdzicki, M.; Hu, P.; Eddings, E.; Robbins, C.M.; Moses, T.; Namkoong, J.; Chen, S.; Trent, J.M. Cloning and characterization of a novel gene, SHPRH, encoding a conserved putative protein with SNF2/helicase and PHD-finger domains from the 6q24 region. *Genomics* **2003**, *82*(2), 153–161. https://doi.org/10.1016/s0888-7543(03)00121-6
136. Brühl, J.; Trautwein, J.; Schäfer, A.; Linne, U.; Bouazoune, K. The DNA repair protein SHPRH is a nucleosome –stimulated ATPase and a nucleosome –E3 ubiquitin ligase. *Epigenet. Chromatin* **2019**, *12*(1), 52. https://doi.org/10.1186/s13072 –019 –0294 –5
137. Seelinger, M.; Søgaard, C.K.; Otterlei, M. The Human RAD5 Homologs, HLTF and SHPRH, Have Separate Functions in DNA Damage Tolerance Dependent on The DNA Lesion Type. *Biomolecules* **2020**, *10*(3), 463. https://doi.org/10.3390/biom10030463
138. Kiss, L.; Zeng, J.; Dickson, C.F.; Mallery, D.L.; Yang, J.C.; McLaughlin, S.H.; Boland, A.; Neuhaus, D.; James, L.C. A tri-ionic anchor mechanism drives Ube2N-specific recruitment and K63-chain ubiquitination in TRIM ligases. *Nat. Commun.* **2019,** *10*(1), 4502. https://doi.org/10.1038/s41467-019-12388-y
139. Lee, K.Y.; Myung, K. PCNA modifications for regulation of post –replication repair pathways. *Mol. Cells* **2008**, *26*(1), 5–11.
140. Zhao, Y.; Long, M.; Wang, Y.; Zhang, S.; Aye, Y. Ube2V2 Is a Rosetta Stone Bridging Redox and Ubiquitin Codes, Coordinating DNA Damage Responses. *ACS Cent. Sci.* **2018,** *4*(2), 246–259. https://doi.org/10.1021/acscentsci.7b00556
141. Schwartz, S.M.; Schwartz, H.T.; Horvath, S.; Schadt, E.; Lee, S.I. A systematic approach to multifactorial cardiovascular disease: causal analysis. *ATVB* **2012**, *32*(12), 2821–2835. https://doi.org/10.1161/ATVBAHA.112.300123

142. Matsumoto, T.; Mugishima, H. Signal transduction via vascular endothelial growth factor (VEGF) receptors and their roles in atherogenesis. J. Atheroscler. Thromb. 2006, 13(3), 130–135. https://doi.org/10.5551/jat.13.130
143. Brázda, V.; Laister, R.C.; Jagelská, E.B.; Arrowsmith, C. Cruciform structures are a common DNA feature important for regulating biological processes. *BMC Mol Biol.* **2011**, *12*, 33. https://doi.org/10.1186/1471 –2199 –12 –33
144. Yue, L.; Wan, R.; Luan, S.; Zeng, W.; Cheung, T.H. Dek Modulates Global Intron Retention during Muscle Stem Cells Quiescence. *Exit. Dev. Cell* **2020**, 53(6), 661–676.e6. https://doi.org/10.1016/j.devcel.2020.05.006
145. Cecchettini, A.; Rocchiccioli, S.; Boccardi, C.; Citti, L. Vascular smooth-muscle-cell activation: proteomics point of view. In *International review of cell and molecular biology,*1st ed.; Jeon, K. Eds.; Academic Press: San Diego, USA; Waltham, USA; London, UK; Amsterdam, The Netherlands **2011**, Volume 288, pp. 43–99. https://doi.org/10.1016/B978-0-12-386041-5.00002-9
146. Audain, E.; Wilsdon, A.; Breckpot, J.; Izarzugaza, J.; Fitzgerald, T. W.; Kahlert, A.K.; Sifrim, A.; Wünnemann, F.; Perez –Riverol, Y.; Abdul –Khaliq, H.; Bak, M.; Bassett, A.S.; Benson, W.D.; Berger, F.; Daehnert, I.; Devriendt, K.; Dittrich, S.; Daubeney, P.E.; Garg, V.; Hackmann, K.; Hoff, K.; Hofmann, P.; Dombrowsky, G.; Pickardt, T.; Bauer, U.; Keavney, B.D.; Klaassen, S.; Kramer, H.H.; Marshall, C.R.; Milewicz, D.M.; Lemaire, S.; Coselli, J.S.; Mitchell, M.E.; Tomita –Mitchell, A.; Prakash, S.K.; Stamm, K.; Stewart, A.F.R.; Silversides, C.K.; Siebert, R.; Stiller, B.; Rosenfeld, J.A.; Vater, I.; Postma, A.V.; Caliebe, A.; Brook, J.D.; Andelfinger, G.; Hurles, M.E.; Thienpont, B.; Larsen, L.A.; Hitz, M. P. Integrative analysis of genomic variants reveals new associations of candidate haploinsufficient genes with congenital heart disease. *PLoS Genet.* **2021**, *17*(7), e1009679. https://doi.org/10.1371/journal.pgen.1009679
147. Coutts, A.S.; Weston, L.; La Thangue, N.B. Actin nucleation by a transcription co –factor that links cytoskeletal events with the p53 response. *Cell cycle* **2010**, *9*(8), 1511–1515. https://doi.org/10.4161/cc.9.8.11258
148. Zhang, R.; Ji, Z.; Yao, Y.; Zuo, W.; Yang, M.; Qu, Y.; Su, Y.; Ma, G.; Li, Y. Identification of hub genes in unstable atherosclerotic plaque by conjoint analysis of bioinformatics**.** *Life Sci.***2020**, *262*, 118517. https://doi.org/10.1016/j.lfs.2020.118517
149. Scheffler, K.; Rachek, L.; You, P.; Rowe, A.D.; Wang, W.; Kuśnierczyk, A.; Kittelsen, L.; Bjørås, M.; Eide, L. 8-oxoguanine DNA glycosylase (Ogg1) controls hepatic gluconeogenesis. *DNA repair* **2018**, *61*, 56–62. https://doi.org/10.1016/j.dnarep.2017.11.008
150. Komakula, S.; Tumova, J.; Kumaraswamy, D.; Burchat, N.; Vartanian, V.; Ye, H.; Dobrzyn, A.; Lloyd, R.S.; Sampath, H. The DNA Repair Protein OGG1 Protects Against Obesity by Altering Mitochondrial Energetics in White Adipose Tissue. *Sci. Rep*. **2018,** *8*(1), 14886. https://doi.org/10.1038/s41598-018-33151-1
151. Ha, K.; Ma, C.; Lin, H.; Tang, L.; Lian, Z.; Zhao, F.; Li, J.M.; Zhen, B.; Pei, H.; Han, S.; Malumbres, M.; Jin, J.; Chen, H.; Zhao, Y.; Zhu, Q.; Zhang, P. The anaphase promoting complex impacts repair choice by protecting ubiquitin signalling at DNA damage sites. *Nat. Commun.* **2017,** *8*, 15751. https://doi.org/10.1038/ncomms15751
152. Jeong, K.; Murphy, J.M.; Erin Ahn, E.Y.; Steve Lim, S.T. FAK in the nucleus prevents VSMC proliferation by promoting p27 and p21 expression via Skp2 degradation. *Nat. Cardiovasc. Res*. **2021,** cvab132. Advance online publication. https://doi.org/10.1093/cvr/cvab132
153. Zhang, Y.M.; Meng, L.B.; Yu, S.J.; Ma, D.X. Identification of potential crucial genes in monocytes for atherosclerosis using bioinformatics analysis. *Int. J. Med. Res.* **2020**, *48*(4), 300060520909277. https://doi.org/10.1177/0300060520909277
154. Manils, J.; Gómez, D.; Salla –Martret, M.; Fischer, H.; Fye, J.M.; Marzo, E.; Marruecos, L.; Serrano, I.; Salgado, R.; Rodrigo, J.P.; Garcia –Pedrero, J.M.; Serafin, A.M.; Cañas, X.; Benito, C.; Toll, A., Forcales, S.V.; Perrino, F.W.; Eckhart, L.; Soler, C. Multifaceted role of TREX2 in the skin defense against UV –induced skin carcinogenesis. *Oncotarget* **2015**, *6*(26), 22375–22396. https://doi.org/10.18632/oncotarget.4296

155. Certo, M.T.; Gwiazda, K.S.; Kuhar, R.; Sather, B.; Curinga, G.; Mandt, T.; Brault, M.; Lambert, A.R.; Baxter, S.K.; Jacoby, K.; Ryu, B.Y.; Kiem, H.P.; Gouble, A.; Paques, F.; Rawlings, D.J.; Scharenberg, A.M. Coupling endonucleases with DNA end-processing enzymes to drive gene disruption. *Nat. Methods* **2012,** *9*(10), 973–975. https://doi.org/10.1038/nmeth.2177
156. Manils, J.; Casas, E.; Viña –Vilaseca, A.; López –Cano, M.; Díez –Villanueva, A.; Gómez, D.; Marruecos, L.; Ferran, M.; Benito, C.; Perrino, F.W.; Vavouri, T.; de Anta, J.M.; Ciruela, F.; Soler, C. The Exonuclease Trex2 Shapes Psoriatic Phenotype. *J. Invest. Dermatol.* **2016**, *136*(12), 2345–2355. https://doi.org/10.1016/j.jid.2016.05.122
157. Hoffman, A.E.; Zheng, T.; Ba, Y.; Zhu, Y. The circadian gene NPAS2, a putative tumor suppressor, is involved in DNA damage response. *Molecular Cancer Res.: MCR* **2008**, *6*(9), 1461–1468. https://doi.org/10.1158/1541 –7786.MCR –07 –2094
158. McNamara, P.; Seo, S.B.; Rudic, R.D.; Sehgal, A.; Chakravarti, D.; FitzGerald, G.A. Regulation of CLOCK and MOP4 by nuclear hormone receptors in the vasculature: a humoral mechanism to reset a peripheral clock. *Cell* **2001,** *105*(7), 877–889. https://doi.org/10.1016/s0092-8674(01)00401-9
159. Westgate, E.J.; Cheng, Y.; Reilly, D.F.; Price, T.S.; Walisser, J.A.; Bradfield, C.A.; FitzGerald, G.A. Genetic components of the circadian clock regulate thrombogenesis in vivo. *Circulation* **2008,** *117*(16), 2087–2095. https://doi.org/10.1161/CIRCULATIONAHA.107.739227
160. Palmieri, O.; Mazzoccoli, G.; Bossa, F.; Maglietta, R.; Palumbo, O.; Ancona, N.; Corritore, G.; Latiano, T.; Martino, G.; Rubino, R.; Biscaglia, G.; Scimeca, D.; Carella, M.; Annese, V.; Andriulli, A.; Latiano, A. Systematic analysis of circadian genes using genome-wide cDNA microarrays in the inflammatory bowel disease transcriptome. *Chronobiol. Int*. **2015** *32*(7), 903–916. https://doi.org/10.3109/07420528.2015.1050726
161. Pourcet, B.; Duez, H. Nuclear Receptors and Clock Components in Cardiovascular Diseases. *Int. J. Mol. Sci.* **2021,** *22*(18), 9721. https://doi.org/10.3390/ijms22189721
162. Marchand, B.; Pitarresi, J.R.; Reichert, M.; Suzuki, K.; Laczkó, D.; Rustgi, A.K. PRRX1 isoforms cooperate with FOXM1 to regulate the DNA damage response in pancreatic cancer cells. *Oncogene* **2019**, *38*(22), 4325–4339. https://doi.org/10.1038/s41388 –019 –0725 –6
163. Higuchi, M.; Kato, T.; Yoshida, S.; Ueharu, H.; Nishimura, N.; Kato, Y. PRRX1 – and PRRX2 – positive mesenchymal stem/progenitor cells are involved in vasculogenesis during rat embryonic pituitary development. *Cell Tissue Res.* **2015**, *361*(2), 557–565. https://doi.org/10.1007/s00441 –015 –2128 –5
164. Wang, X.; Yang, R.; Wang, Q.; Wang, Y.; Ci, H.; Wu, S. Aberrant expression of vasculogenic mimicry, PRRX1, and CIP2A in clear cell renal cell carcinoma and its clinicopathological significance. *Medicine* **2019**, *98*(36), e17028. https://doi.org/10.1097/MD.0000000000017028
165. Chen, Z.; Chen, Y.; Li, Y.; Lian, W.; Zheng, K.; Zhang, Y.; Zhang, Y.; Lin, C.; Liu, C.; Sun, F.; Sun, X.; Wang, J.; Zhao, L.; Ke, Y. Prrx1 promotes stemness and angiogenesis via activating TGF –β/smad pathway and upregulating proangiogenic factors in glioma. *Cell Death Dis.* **2021**, *12*(6), 615. https://doi.org/10.1038/s41419 –021 –03882 –7
166. Mancini, M.; Petretto, E.; Kleinert, C.; Scavone, A.; De, T.; Cook, S.; Silhavy, J.; Zidek, V.; Pravenec, M.; d'Amati, G.; Camici, P.G. Mapping genetic determinants of coronary microvascular remodeling in the spontaneously hypertensive rat. *Basic Res. Cardiol.* **2013**, *108*(1), 316. https://doi.org/10.1007/s00395 –012 –0316 –y
167. Lu, S.; Zhong, J.; Wu, M.; Huang, K.; Zhou, Y.; Zhong, Z.; Li, Q.; Zhou, H. Genetic analysis of the relation of telomere length-related gene (RTEL1) and coronary heart disease risk. *Mol. Genet. Genomic Med.* **2019,** *7*(3), e550. https://doi.org/10.1002/mgg3.550
168. Kimura, Y.; Izumiya, Y.; Araki, S.; Yamamura, S.; Hanatani, S.; Onoue, Y.; Ishida, T.; Arima, Y.; Nakamura, T.; Yamamoto, E.; Senokuchi, T.; Yoshizawa, T.; Sata, M., Kim –Mitsuyama, S.; Nakagata, N.; Bober, E.; Braun, T.; Kaikita, K.; Yamagata, K.; Tsujita, K. Sirt7 Deficiency Attenuates Neointimal Formation Following Vascular Injury by Modulating Vascular Smooth

Muscle Cell Proliferation. *Circ. J.***2021**, 10.1253/circj.CJ –20 –0936. Advance online publication. https://doi.org/10.1253/circj.CJ –20 –0936

169. Zheng, J.; Chen, K.; Wang, H.; Chen, Z.; Xi, Y.; Yin, H.; Lai, K.; Liu, Y. SIRT7 Regulates the Vascular Smooth Muscle Cells Proliferation and Migration via Wnt/β –Catenin Signaling Pathway. *Biomed Res. Int.* **2018**, *2018*, 4769596. https://doi.org/10.1155/2018/4769596
170. Frösen, J.; Tulamo, R.; Heikura, T.; Sammalkorpi, S.; Niemelä, M.; Hernesniemi, J.; Levonen, A.L.; Hörkkö, S.; Ylä-Herttuala, S. Lipid accumulation, lipid oxidation, and low plasma levels of acquired antibodies against oxidized lipids associate with degeneration and rupture of the intracranial aneurysm wall. *Acta Neuropathol. Commun.*, **2013,** *1*, 71. https://doi.org/10.1186/2051-5960-1-71
171. Ollikainen, E.; Tulamo, R.; Lehti, S.; Lee-Rueckert, M.; Hernesniemi, J.; Niemelä, M.; Ylä-Herttuala, S.; Kovanen, P.T.; Frösen, J. Smooth Muscle Cell Foam Cell Formation, Apolipoproteins, and ABCA1 in Intracranial Aneurysms: Implications for Lipid Accumulation as a Promoter of Aneurysm Wall Rupture. *J. Neuropathol. Exp. Neurol.* **2016,** *75*(7), 689–699. https://doi.org/10.1093/jnen/nlw041
172. Galora, S.; Saracini, C.; Palombella, A.M.; Pratesi, G.; Pulli, R.; Pratesi, C.; Abbate, R.; Giusti, B. (2013). Low-density lipoprotein receptor-related protein 5 gene polymorphisms and genetic susceptibility to abdominal aortic aneurysm. *J. Vasc. Surg. 58*(4), 1062–8.e1. https://doi.org/10.1016/j.jvs.2012.11.092
173. Mourmoura, E.; Vasilaki, A.; Giannoukas, A.; Michalodimitrakis, E.; Pavlidis, P.; Tsezou, A. Evidence of deregulated cholesterol efflux in abdominal aortic aneurysm. *Acta Histochem.* **2016,** 118(2), 97–108. https://doi.org/10.1016/j.acthis.2015.11.012
174. Benigni, A.; Cassis, P.; Remuzzi, G. Angiotensin II revisited: new roles in inflammation, immunology and aging. *EMBO Mol. Med.* **2010,** *2*(7), 247–257. https://doi.org/10.1002/emmm.201000080
175. Lv, P.; Zhang, F.; Yin, Y.J.; Wang, Y.C.; Gao, M.; Xie, X.L.; Zhao, L.L.; Dong, L.H.; Lin, Y.L.; Shu, Y.N.; Zhang, D.D.; Liu, G.X.; Han, M. SM22α inhibits lamellipodium formation and migration via Ras –Arp2/3 signaling in synthetic VSMCs. *Am. J. Physiol. Cell Physiol.* **2016**, *311*(5), C758–C767. https://doi.org/10.1152/ajpcell.00033.2016
176. Li, W.; Xin, B.; Yan, J.; Wu, Y.; Hu, B.; Liu, L.; Wang, Y.; Ahn, J.; Skowronski, J.; Zhang, Z.; Wang, Y.; Wang, H. SAMHD1 Gene Mutations Are Associated with Cerebral Large-Artery Atherosclerosis. *Biomed Res. Int*, **2015**, 739586. https://doi.org/10.1155/2015/739586
177. Wu, C.C.; Peng, S.S.; Lee, W.T. Intracerebral large artery disease in Aicardi-Goutières syndrome with TREX1 mutation: a case report. *Neurol. Sci*, **2020,** *41*(11), 3353–3356. https://doi.org/10.1007/s10072-020-04516-0
178. Xin, B.; Jones, S.; Puffenberger, E. G.; Hinze, C.; Bright, A.; Tan, H.; Zhou, A.; Wu, G.; Vargus-Adams, J.; Agamanolis, D.; Wang, H. Homozygous mutation in SAMHD1 gene causes cerebral vasculopathy and early onset stroke. *Proc. Natl. Acad. Sci. U.S.A.* **2011,** 108(13), 5372–5377. https://doi.org/10.1073/pnas.1014265108
179. Bae, N.S.; Baumann, P. A RAP1/TRF2 complex inhibits nonhomologous end –joining at human telomeric DNA ends. *Mol. Cell* **2007**, *26*(3), 323–334. https://doi.org/10.1016/j.molcel.2007.03.023
180. Kotla, S.; Vu, H.T.; Ko, K.A.; Wang, Y.; Imanishi, M.; Heo, K.S.; Fujii, Y.; Thomas, T.N.; Gi, Y.J.; Mazhar, H.; Paez –Mayorga, J.; Shin, J.H.; Tao, Y.; Giancursio, C.J.; Medina, J.L.; Taunton, J.; Lusis, A.J.; Cooke, J.P.; Fujiwara, K.; Le, N.T.; Abe, J.I. Endothelial senescence is induced by phosphorylation and nuclear export of telomeric repeat binding factor 2 –interacting protein. *JCI Insight* **2019**, *4*(9), e124867. https://doi.org/10.1172/jci.insight.124867
181. Duan, Y.; Huang, S.; Yang, J.; Niu, P.; Gong, Z.; Liu, X.; Xin, L.; Currie, R.W.; Wu, T. HspA1A facilitates DNA repair in human bronchial epithelial cells exposed to Benzo[a]pyrene and interacts with casein kinase 2. *Cell Stress Chaperones* **2014**, *19*(2), 271–279. https://doi.org/10.1007/s12192 –013 –0454 –7

182. Dulin, E.; García-Barreno, P.; Guisasola, M.C. Extracellular heat shock protein 70 (HSPA1A) and classical vascular risk factors in a general population. *Cell Stress Chaperones* **2010,** *15*(6), 929–937. https://doi.org/10.1007/s12192-010-0201-2
183. Dulin, E.; García-Barreno, P.; Guisasola, M.C. Genetic variations of HSPA1A, the heat shock protein levels, and risk of atherosclerosis. *Cell Stress Chaperones*, **2012,** *17*(4), 507–516. https://doi.org/10.1007/s12192-012-0328-4
184. Abbey, D.; Conlon, D.; Rainville, C.; Elwyn, S.; Quiroz –Figueroa, K.; Billheimer, J.; Schultz, D.C.; Hand, N.J.; Cherry, S.; Rader, D.J. Lipid droplet screen in human hepatocytes identifies TRRAP as a regulator of cellular triglyceride metabolism. *Clin. Transl. Sci.* **2021**, *14*(4), 1369–1379. https://doi.org/10.1111/cts.12988
185. Unno, A.; Takada, I.; Takezawa, S.; Oishi, H.; Baba, A.; Shimizu, T.; Tokita, A.; Yanagisawa, J.; Kato, S. TRRAP as a hepatic coactivator of LXR and FXR function. *Biochem. Biophys. Res. Commun.* **2005,** *327*(3), 933–938. https://doi.org/10.1016/j.bbrc.2004.12.095
186. Zhang, D.D.; Song, Y.; Kong, P.; Xu, X.; Gao, Y.K.; Dou, Y.Q.; Weng, L.; Wang, X.W.; Lin, Y.L.; Zhang, F.; Zhang, H.; Han, M. Smooth muscle 22 alpha protein inhibits VSMC foam cell formation by supporting normal LXRα signaling, ameliorating atherosclerosis. *Cell Death Dis.* **2021,** *12*(11), 982. https://doi.org/10.1038/s41419-021-04239-w
187. Luan, Z.L.; Wei, Y.Y.; Wang, Y.C.; Ming, W.H.; Zhang, H.B.; Wang, B.; Cui, X.H.; Li, Y.Y.; Guan, Y.F.; Zhang, X.Y. Farnesoid X receptor (FXR) inhibits coagulation process via inducing hepatic antithrombin III expression in mice. *Sheng li xue bao* : [*Acta physiologica Sinica*], **2021,** *73*(5), 795–804.
188. Collis, S.J.; Barber, L.J.; Clark, A.J.; Martin, J.S.; Ward, J.D.; Boulton, S.J. HCLK2 is essential for the mammalian S-phase checkpoint and impacts on Chk1 stability. *Nat. Cell Biol.* **2007,** *9*(4), 391–401. https://doi.org/10.1038/ncb1555
189. Eisenreich, A.; Bogdanov, V.Y.; Zakrzewicz, A.; Pries, A.; Antoniak, S.; Poller, W.; Schultheiss, H.P.; Rauch, U. Cdc2-like kinases and DNA topoisomerase I regulate alternative splicing of tissue factor in human endothelial cells. *Circ. Res.* **2009,** *104*(5), 589–599. https://doi.org/10.1161/CIRCRESAHA.108.183905
190. Nakazawa, Y.; Hara, Y.; Oka, Y.; Komine, O.; van den Heuvel, D.; Guo, C.; Daigaku, Y.; Isono, M.; He, Y.; Shimada, M.; Kato, K.; Jia, N.; Hashimoto, S.; Kotani, Y.; Miyoshi, Y.; Tanaka, M.; Sobue, A.; Mitsutake, N.; Suganami, T.; Masuda, A.; Ohno K.; Nakada S.; Mashimo T.; Yamanaka K.; Luijsterburg M.S.; Ogi.; T. Ubiquitination of DNA Damage-Stalled RNAPII Promotes Transcription-Coupled Repair.*Cell* **2020**, *180*(6), 1228–1244.e24. https://doi.org/10.1016/j.cell.2020.02.010
191. Sarasin A. UVSSA and USP7: new players regulating transcription-coupled nucleotide excision repair in human cells. *Genome Med.* **2012**, *4*(5), 44. https://doi.org/10.1186/gm343
192. Rimel, J.K.; Taatjes, D.J. The essential and multifunctional TFIIH complex. *Protein Sci.* **2018**, *27*(6), 1018–1037. https://doi.org/10.1002/pro.3424
193. Herzig, J.K.; Bullinger, L.; Tasdogan, A.; Zimmermann, P.; Schlegel, M.; Teleanu, V.; Weber, D.; Rücker, F.G.; Paschka, P.; Dolnik, A.; Schneider, E.; Kuchenbauer, F.; Heidel, F.H.; Buske, C.; Döhner, H.; Döhner, K.; Gaidzik, V.I. Protein phosphatase 4 regulatory subunit 2 *(PPP4R2)* is recurrently deleted in acute myeloid leukemia and required for efficient DNA double strand break repair. *Oncotarget* **2017**, *8*(56), 95038–95053. https://doi.org/10.18632/oncotarget.21119
194. Guo, X.; Lin, M.; Rockowitz, S.; Lachman, H.M.; Zheng, D. Characterization of human pseudogene-derived non-coding RNAs for functional potential. *PloS one* **2014,** *9*(4), e93972. https://doi.org/10.1371/journal.pone.0093972
195. Kovalenko, T.F.; Patrushev, L.I. Pseudogenes as Functionally Significant Elements of the Genome. *Biochemistry. Biokhimiia* **2018,** *83*(11), 1332–1349. https://doi.org/10.1134/S0006297918110044

196. Zalewski, D.P.; Ruszel, K.P.; Stępniewski, A.; Gałkowski, D.; Bogucki, J.; Kołodziej, P.; Szymańska, J.; Płachno, B.J.; Zubilewicz, T.; Feldo, M.; Kocki, J.; Bogucka–Kocka, A. Identification of Transcriptomic Differences between Lower Extremities Arterial Disease, Abdominal Aortic Aneurysm and Chronic Venous Disease in Peripheral Blood Mononuclear Cells Specimens. *Int. J. Mol. Sci* **2021,** *22*(6), 3200. https://doi.org/10.3390/ijms22063200
197. Wang, Q.; Goldstein, M.; Alexander, P.; Wakeman, T.P.; Sun, T.; Feng, J.; Lou, Z.; Kastan, M.B.; Wang, X.F. Rad17 recruits the MRE11 –RAD50 –NBS1 complex to regulate the cellular response to DNA double –strand breaks. *EMBO J.* **2014**, *33*(8), 862–877. https://doi.org/10.1002/embj.201386064
198. Abe, T.; Ooka, M.; Kawasumi, R.; Miyata, K.; Takata, M.; Hirota, K.; Branzei, D. Warsaw breakage syndrome DDX11 helicase acts jointly with RAD17 in the repair of bulky lesions and replication through abasic sites. *Proc. Natl. Acad. Sci U. S. A.* **2018**, *115*(33), 8412–8417. https://doi.org/10.1073/pnas.1803110115
199. Tsuda, M.; Kitamasu, K.; Kumagai, C.; Sugiyama, K.; Nakano, T.; Ide, H. Tyrosyl –DNA phosphodiesterase 2 (TDP2) repairs topoisomerase 1 DNA –protein crosslinks and 3' –blocking lesions in the absence of tyrosyl –DNA phosphodiesterase 1 (TDP1). *DNA repair* **2020**, *91 –92*, 102849. https://doi.org/10.1016/j.dnarep.2020.102849
200. Schellenberg, M.J.; Appel, C.D.; Riccio, A.A.; Butler, L.R.; Krahn, J.M.; Liebermann, J.A.; Cortés –Ledesma, F.; Williams, R.S. Ubiquitin stimulated reversal of topoisomerase 2 DNA –protein crosslinks by TDP2. *Nucleic Acids Res.* **2020**, *48*(11), 6310–6325. https://doi.org/10.1093/nar/gkaa318
201. Shiomi, Y.; Nishitani, H. Control of Genome Integrity by RFC Complexes; Conductors of PCNA Loading onto and Unloading from Chromatin during DNA Replication. *Genes* **2017**, *8*(2), 52. https://doi.org/10.3390/genes8020052
202. Giovannini, S.; Weller, M.C.; Hanzlíková, H.; Shiota, T.; Takeda, S.; Jiricny, J. ATAD5 deficiency alters DNA damage metabolism and sensitizes cells to PARP inhibition. *Nucleic Acids Res.* **2020**, *48*(9), 4928–4939. https://doi.org/10.1093/nar/gkaa255
203. Babatz, T.D.; Spear, E.D.; Xu, W.; Sun, O.L.; Nie, L.; Carpenter, E.P.; Michaelis, S. Site specificity determinants for prelamin A cleavage by the zinc metalloprotease ZMPSTE24. *J. Biol. Chem.* **2021**, *296*, 100165. https://doi.org/10.1074/jbc.RA120.015792
204. Matrone, G.; Thandavarayan, R.A.; Walther, B.K.; Meng, S.; Mojiri, A.; Cooke, J. P. Dysfunction of iPSC-derived endothelial cells in human Hutchinson-Gilford progeria syndrome. *Cell cycle* **2019,** *18*(19), 2495–2508. https://doi.org/10.1080/15384101.2019.1651587
205. Messner, M.; Ghadge, S.K.; Maurer, T.; Graber, M.; Staggl, S.; Christine Maier, S.; Pölzl, G.; Zaruba, M.M. ZMPSTE24 Is Associated with Elevated Inflammation and Progerin mRNA. *Cells* **2020**, *9*(9), 1981. https://doi.org/10.3390/cells9091981
